# Supplementary material for: Visible-Light-Induced Decarboxylation of Dioxazolones to Phosphinimidic Amides and Ureas
Source: Molecules. 2022 Jun 7;27(12):3648. doi: 10.3390/molecules27123648 (PMC9229220; doi:10.3390/molecules27123648)
Supplement: Supplementary file 1 [file molecules-27-03648-s001.zip › molecules-1734945-supplementary.pdf]

Supporting Information

Visible-light-induced decarboxylation of dioxazolones to phosphinimidic amides and ureas

Jie Pan [1], Hao-Cong Li [1], Kai Sun [1,2,*], Shi Tang [3], and Bing Yu [1,*]

1 Green Catalysis Center, College of Chemistry, Zhengzhou University, Zhengzhou 450001, China.
2 College of Chemistry & Materials Engineering, Huaihua University, Huaihua 418008, China.
3 College of Chemistry and Chemical Engineering, Jishou University, Jishou 416000, China
* Correspondence: sunkaichem@zzu.edu.cn (K.S.); bingyu@zzu.edu.cn (B.Y.).

Table of Contents

1. **General information......S2**
2. **Experimental procedures S4**
3. **Procedure for optimization studies......S10**
4. **Characterization of compounds......S12**
5. **NMR copies of products S28**

1. General information

All nuclear magnetic resonance (NMR) spectra were recorded on a Bruker Avance 400 MHz in $CDCl_3$ at room temperature (20 ± 3 °C), by using tetramethylsilane as the internal standard. High-resolution mass spectra (HRMS) were conducted on a 3000-mass spectrometer, using Waters Q-Tof MS/MS system with the ESI technique.

Photochemical reactions were carried out under visible light irradiation by a blue LED at 25 °C. RLH-18 8-position Photo Reaction System manufactured by Beijing Roger Tech Ltd. was used in this system. Eight 10 W blue LEDs were equipped in this photochemical reactor (Figure S1). The wavelength for blue LED is 430 nm, peak width at half-height is 18.4 nm (Figure S2). The distance from the light source to the irradiation vessel was approximately 15 mm.

Most of the starting materials used in this study are commercial and were purchased in the available from Shanghai Aladdin Biochemical Technology Co., Ltd.; Saen Chemical Technology (Shanghai) Co., Ltd.; Shanghai Bide Pharmaceutical Technology Co., Ltd.; Henan Alpha Chemical Co., Ltd. and Tianjin Comeo Chemical Reagent Co., Ltd. used as received, without further purifications. All dioxazolones [59] were prepared as previously described.

Table S1. The commercial sources of all starting materials

Starting materials	Commercial sources
Triphenylphosphine	Tianjin Comeo Chemical Reagent Co., Ltd.
Tris(4-methylphenyl)phosphine	Shanghai Bide Pharmaceutical Technology Co., Ltd.
Tris(4-methoxyphenyl)phosphine	Shanghai Bide Pharmaceutical Technology Co., Ltd.
Tri(4-chlorophenyl)phosphine	Shanghai Bide Pharmaceutical Technology Co., Ltd.
Tris(4-fluorophenyl)phosphane	Shanghai Bide Pharmaceutical Technology Co., Ltd.
Tris(3-methoxyphenyl)phosphane	Shanghai Bide Pharmaceutical Technology Co., Ltd.
Diphenyl(4-tolyl)phosphinen	Saen Chemical Technology (Shanghai) Co., Ltd.
2,2'-Bis(diphenylphosphino)-1,1'-binaphthalene	Henan Alpha Chemical Co., Ltd.
Diisopropylamine	Saen Chemical Technology (Shanghai) Co., Ltd.
Diethylamine	Shanghai Aladdin Biochemical Technology Co., Ltd.
Dipropylamine	Shanghai Aladdin Biochemical Technology Co., Ltd.
Dibutylamine	Shanghai Aladdin Biochemical Technology Co., Ltd.
N-benzylisopropylamine	Henan Alpha Chemical Co., Ltd.
N-ethylpropan-1-amine	Saen Chemical Technology (Shanghai) Co., Ltd.
N-ethylcyclohexylamine	Saen Chemical Technology (Shanghai) Co., Ltd.
N-ethylbenzylamine	Saen Chemical Technology (Shanghai) Co., Ltd.
1,2,3,4-Tetrahydroisoquinoline	Shanghai Bide Pharmaceutical Technology Co., Ltd.
Aniline	Saen Chemical Technology (Shanghai) Co., Ltd.
Cyclohexylamine	Saen Chemical Technology (Shanghai) Co., Ltd.
Benzylamine	Saen Chemical Technology (Shanghai) Co., Ltd.
Dibenzoylmethane	Saen Chemical Technology (Shanghai) Co., Ltd.
Benzohydroxamic acid	Shanghai Bide Pharmaceutical Technology Co., Ltd.
N,N'-Carbonyldiimidazole	Shanghai Bide Pharmaceutical Technology Co., Ltd.
4-*tert*-butylbenzoyl chloride	Shanghai Aladdin Biochemical Technology Co., Ltd.
4-cyanobenzoyl chloride	Shanghai Aladdin Biochemical Technology Co., Ltd.
3-chlorobenzoyl chloride	Shanghai Aladdin Biochemical Technology Co., Ltd.
3-methoxybenzoyl chloride	Shanghai Aladdin Biochemical Technology Co., Ltd.
3-(trifluoromethyl)benzoyl chloride	Shanghai Aladdin Biochemical Technology Co., Ltd.
3-fluorobenzoyl chloride	Shanghai Aladdin Biochemical Technology Co., Ltd.

2-methoxybenzoyl chloride	Shanghai Aladdin Biochemical Technology Co., Ltd.
2-chlorobenzoyl chloride	Shanghai Aladdin Biochemical Technology Co., Ltd.
2-fluorobenzoyl chloride	Shanghai Aladdin Biochemical Technology Co., Ltd.
4-methoxybenzoyl chloride	Saen Chemical Technology (Shanghai) Co., Ltd.
4-(trifluoromethyl)benzoyl chloride	Saen Chemical Technology (Shanghai) Co., Ltd.
4-(trifluoromethyl)benzoyl chloride	Saen Chemical Technology (Shanghai) Co., Ltd.
4-chlorobenzoyl chloride	Saen Chemical Technology (Shanghai) Co., Ltd.
4-(chloromethyl)benzoyl chloride	Saen Chemical Technology (Shanghai) Co., Ltd.
3-bromobenzoyl chloride	Saen Chemical Technology (Shanghai) Co., Ltd.
2-bromobenzoyl chloride	Saen Chemical Technology (Shanghai) Co., Ltd.
2-furoyl chloride	Saen Chemical Technology (Shanghai) Co., Ltd.
2-thiophenecarbonyl chloride	Shanghai Bide Pharmaceutical Technology Co., Ltd.
4-fluorobenzoyl chloride	Henan Alpha Chemical Co., Ltd.

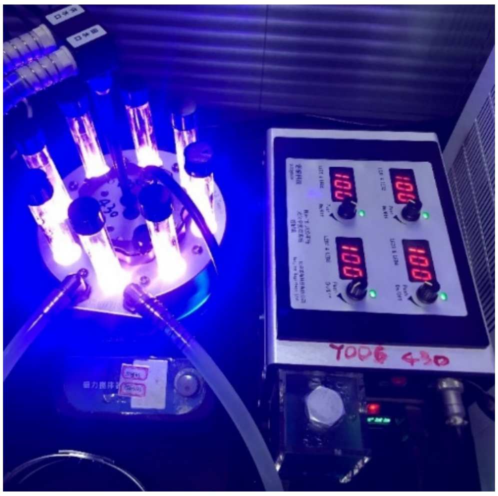

Figure S1 The reaction apparatus.

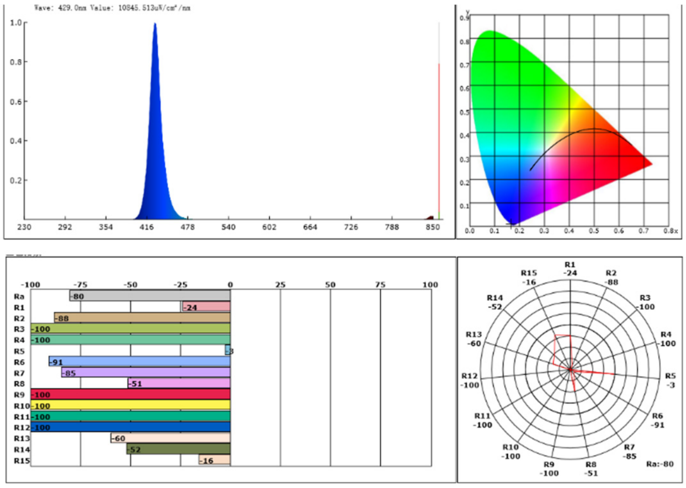

Figure S2 The spectrum of the blue LED.

2. Experimental procedures

2.1 General procedure for the preparation of dioxazolone

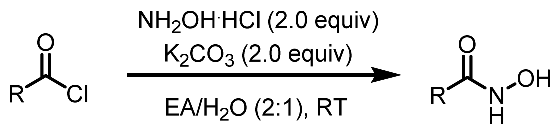

Following a modified procedure, hydroxylamine hydrochloride (20.0 mmol), ethyl acetate (60 mL), H_2O (40 mL) and K_2CO_3 (20.0 mmol) were added to a 250 mL flask at 0 °C. Then acyl chloride (10.0 mmol) dissolved in 20 mL ethyl acetate was added to the resulting mixture dropwise. The solution was warmed up to room temperature and stirred overnight. After that, the reaction mixture was extracted with ethyl acetate, washed with water and brine and dried over anhydrous Na_2SO_4. The solvent was evaporated under the reduced pressure to afford the desired products for the next step without further purification.

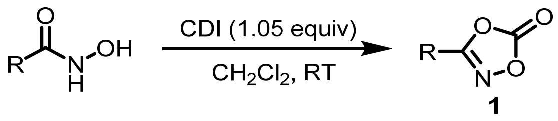

To a stirred solution of hydroxamic acid (10.0 mmol) in CH_2Cl_2 (50 mL) in a 250 mL flask was added 1,1'-carbonyldiimidazole (CDI 1.70 g, 10.5 mmol) at room temperature. After stirring for 20-30 min, the reaction mixture was quenched with 1 M HCl (50 mL) and extracted with CH_2Cl_2, dried over Na_2SO_4, and concentrated under reduced pressure to give the desired dioxazolones **1**.

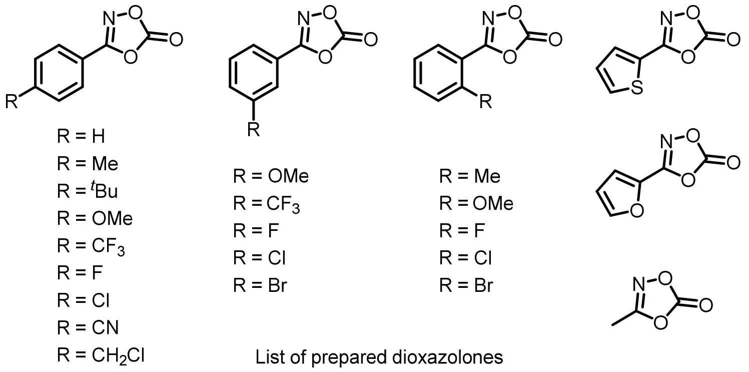

List of prepared dioxazolones

2.2 General experimental procedures for the synthesis of 3.

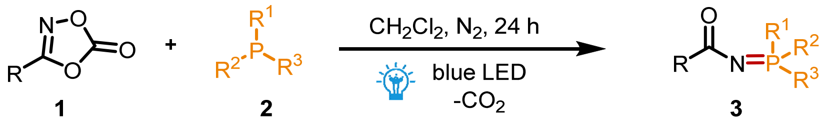

In a 25 mL reaction tube, dioxazolones **1** (0.2 mmol, 1.0 equiv), organic phosphine substrate **2** (0.2 mmol, 1.0 equiv) in 1 mL CH_2Cl_2 was allowed to stir with irradiation of 10 W blue LED under N_2 atmosphere at room temperature for 24 h. After the reaction, the solvent was evaporated under vacuum, and the residue was purified by column chromatography on silica gel to afford the desired products **3**.

2.3 General experimental procedures for the synthesis of 5.

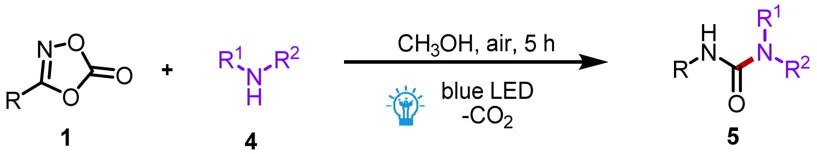

In a 25 mL reaction tube, dioxazolones **1** (0.2 mmol, 1.0 equiv), amines **4** (0.4 mmol, 2.0 equiv) in 1 mL CH_3OH

was allowed to stir with irradiation of 10 W blue LED at room temperature for 5 h. After the reaction, the solvent was evaporated under vacuum, and the residue was purified by column chromatography on silica gel to afford the desired products **5**.

2.4 General experimental procedures for 7 and gram-scale synthesis of 3a.

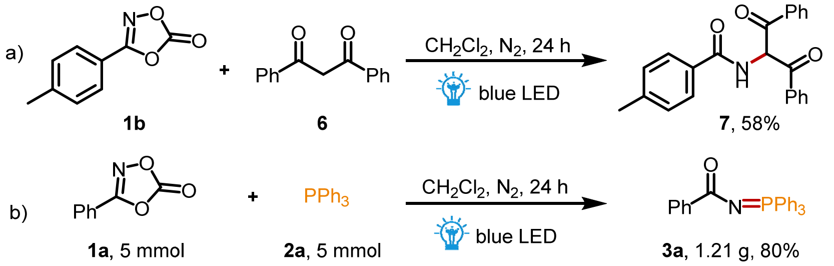

In a 25 mL reaction tube, 3-(*p*-tolyl)-1,4,2-dioxazol-5-one **1b** (0.2 mmol, 1.0 equiv), 1,3-diphenylpropane-1,3-dione **6** (0.3 mmol, 1.5 equiv) in 2 mL CH_2Cl_2 was allowed to stir with irradiation of 10 W blue LED under N_2 atmosphere at room temperature for 24 h. After the reaction, the solvent was evaporated under vacuum, and the residue was purified by column chromatography on silica gel to afford the desired product **7**.

In a 50 mL flask, 3-phenyl-1,4,2-dioxazol-5-one **1a** (5.0 mmol, 0.8 g), triphenylphosphine **2a** (5.0 mmol, 1.3 g) were dissolved in CH_2Cl_2 (20 mL), and then the reaction flask was stirred under N_2 atmosphere at room temperature for 24 h with the irradiation of 10 W blue LED. After the reaction, the solvent was evaporated under vacuum, and the residue was purified by column chromatography on silica gel to afford the desired product **3a**.

2.5 Control experiments

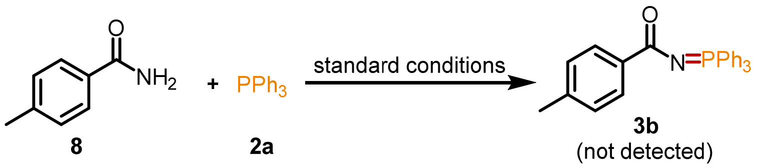

In a 25 mL reaction tube, 4-methylbenzamide **8** (0.2 mmol, 1.0 equiv), triphenylphosphine **2a** (0.2 mmol, 1.0 equiv) in 2 mL CH_2Cl_2 was allowed to stir with irradiation of 10 W blue LED under N_2 atmosphere at room temperature for 24 h. After the reaction was completed, the mixture was quenched with brine and washed with CH_2Cl_2. There is no corresponding product formation.

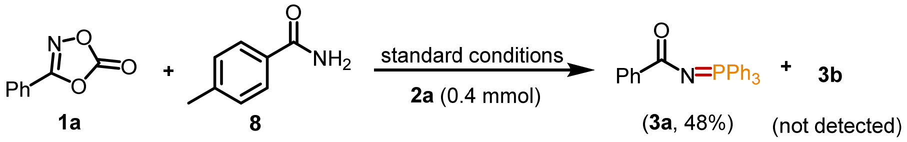

In a 25 mL reaction tube, 4-methylbenzamide **8** (0.2 mmol, 1.0 equiv), 3-phenyl-1,4,2-dioxazol-5-one **1a** (0.2 mmol, 1.0 equiv), triphenylphosphine **2a** (0.4 mmol, 2.0 equiv) in 3 mL CH_2Cl_2 was allowed to stir with irradiation of 10 W blue LED under N_2 atmosphere at room temperature for 24 h. After the reaction, the solvent was evaporated under vacuum, and the residue was purified by column chromatography on silica gel to afford the desired product **3a**, but the corresponding product **3b** was not obtained.

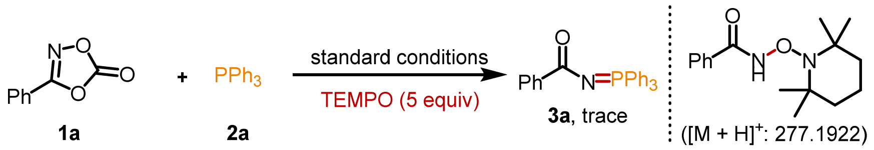

In a 25 mL reaction tube, 3-phenyl-1,4,2-dioxazol-5-one **1a** (0.2 mmol, 1.0 equiv), triphenylphosphine **2a** (0.2 mmol, 1.0 equiv), (2,2,6,6-tetramethyl-1-piperidinyloxy) (TEMPO, 5.0 equiv) in 2 mL CH_2Cl_2 was allowed to stir with irradiation of 10 W blue LED under N_2 atmosphere at room temperature for 24 h. After the reaction, the yield

of **3a** was detected by column chromatography on silica gel.

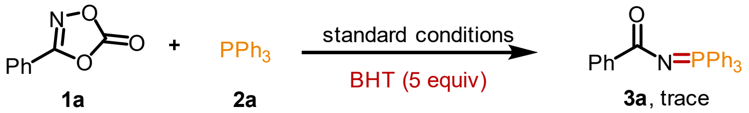

1a **2a** **3a**, trace

In a 25 mL reaction tube, 3-phenyl-1,4,2-dioxazol-5-one **1a** (0.2 mmol, 1.0 equiv), triphenylphosphine **2a** (0.2 mmol, 1.0 equiv), 2,6-di-*tert*-butyl-4-methylphenol (BHT, 5.0 equiv) in 2 mL CH_2Cl_2 was allowed to stir with irradiation of 10 W blue LED under N_2 atmosphere at room temperature for 24 h. After the reaction, the yield of **3a** was detected by column chromatography on silica gel.

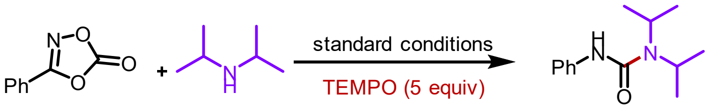

1a **4a** **5a**, 29%

In a 25 mL reaction tube, 3-phenyl-1,4,2-dioxazol-5-one **1a** (0.2 mmol, 1.0 equiv), diisopropylamine **4a** (0.4 mmol, 2.0 equiv), (2,2,6,6-tetramethyl-1-piperidinyloxy) (TEMPO, 5.0 equiv) in 1 mL CH_3OH was allowed to stir with irradiation of 10 W blue LED at room temperature for 5 h. After the reaction, the solvent was evaporated under vacuum, and the residue was purified by column chromatography on silica gel to afford the desired product **5a**.

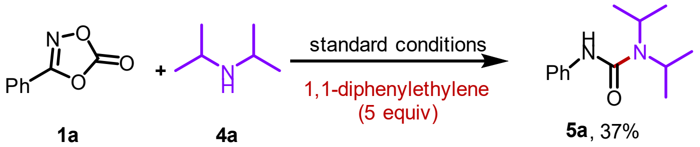

1a **4a** **5a**, 37%

In a 25 mL reaction tube, 3-phenyl-1,4,2-dioxazol-5-one **1a** (0.2 mmol, 1.0 equiv), diisopropylamine **4a** (0.4 mmol, 2.0 equiv), 2,6-di-*tert*-butyl-4-methylphenol (BHT, 5.0 equiv) in 1 mL CH_3OH was allowed to stir with irradiation of 10 W blue LED at room temperature for 5 h. After the reaction, the solvent was evaporated under vacuum, and the residue was purified by column chromatography on silica gel to afford the desired product **5a**.

2.6 HRMS analysis of model reaction solution

To understand the reaction mechanism more deeply, we employed high-resolution mass spectrometry (HRMS) to analyze the reaction solution of the model reaction. The m/z 277.1922 and 577.2448 correspond to the molecular ion $[C_{16}H_{25}N_2O_2]^+$ and $[C_{34}H_{40}N_2O_2P]^+$ as shown in Figure S3.

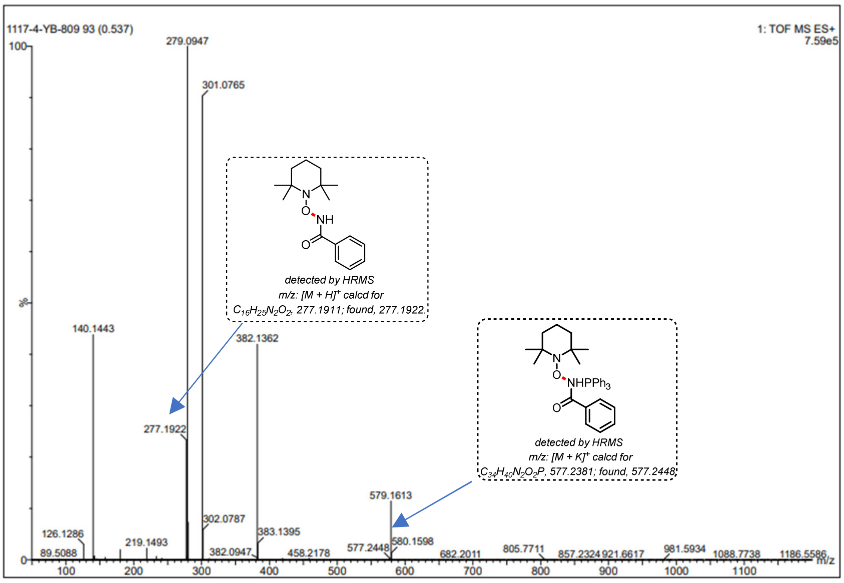

Figure S3 HRMS spectrum analysis of reaction in the presence of TEMPO.

2.7 Sensitivity assessment [a]

A set of experiments that employed **1a** and **2a** were performed to evaluate the sensitivity of the transformation. Compared with the standard conditions, the changes of concentration, temperature, oxygen level, water level, light intensity, and scale were measured. The yield was measured by ^{31}P NMR and the yield deviation was calculated. Among them, light intensity and oxygen level are important parameters of the reaction, which is moderately sensitive to water. Other parameters, such as concentration and temperature, can be regarded as random errors, indicating that the strategy has good repeatability.

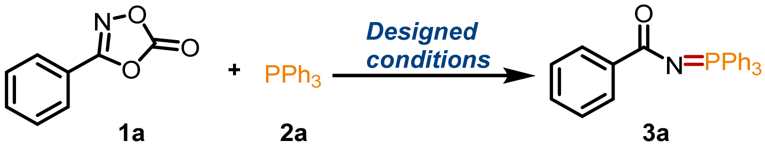

1a **2a** **3a**

Parameter	Variation		Description	Yield[b]	Deviation
Concentration (*c*)	High *c*	*c* + 10% *c*	0.9 mL CH_2Cl_2	80%	3%
	Low *c*	*c* - 10% *c*	1.1 mL CH_2Cl_2	76%	-3%
H_2O level	High H_2O	+ H_2O	10 μL H_2O in 1mL CH_2Cl_2	37%	-53%
O_2 level	High O_2	O_2 balloon	O_2 balloon instead of N_2	trace	-100%
Temperature (*T*)	High *T*	*T* + 10 °C	35 °C	81%	4%
	Low *T*	*T* - 10 °C	15 °C	73%	-7%
Light intensity (*W*)	Low *W*	*W*/16	0.6 W	trace	-100%
Scale	Big scale	50 n	5 mmol of **1a**	80%	3%

[a] Reaction condition: **1a** (0.1 mmol), **2a** (0.1 mmol) in solvent (1 mL) at room temperature for 24 h under the irradiation of 10 W 430 nm blue LED. [b] The average yield of three parallel reactions; yield is determined by ^{31}P NMR.

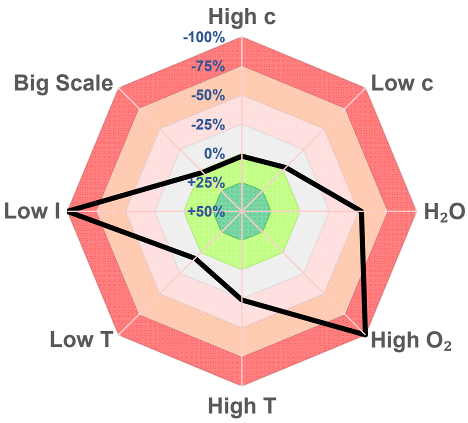

Figure S4 Sensitivity assessment.

2.8 Calculation for E-factor and EcoScale score

Table S2. E-factor of synthesis of phosphinimidic amides system

Item	Reactant 1	Reactant 2	Product
	**1a**	**2a**	**3a**
Mmol	0.2 mmol	0.2 mmol	0.16 mmol
MW	163.03	262.09	381.13
Mass	32.61 mg	52.42 mg	61.74 mg

$$\mathbf{E-factor} = \frac{\Sigma \text{MW of stoichiometric reactants} - \Sigma \text{MW of desired products}}{\Sigma \text{MW of desired products}}$$

$$= \frac{32.61 + 52.42 - 61.74}{61.74}$$

$= 0.38$

Table S3. Penalty points for the synthesis of phosphinimidic amides system

EcoScale penalty points	Factor	Penalty
1.yield	81%	9.5
2.price	3-phenyl-1,4,2-dioxazol-5-one	0
	triphenylphosphane	0
	CH_2Cl_2	0
3.safety	3-phenyl-1,4,2-dioxazol-5-one	0
	triphenylphosphane	0
	CH_2Cl_2	0
4.technical setup	(Inert) gas atmosphere	1
5.temperature/time heating,	Room temperature, < 24 h	1
6. workup and purification	Classical chromatography	10
total penalty points		***21.5***
EcoScale score		***78.5***

Table S4. E-factor of synthesis of ureas system

Item	Reactant 1	Reactant 2	Product
	**1a**	**2a**	**3a**
Mmol	0.2 mmol	0.4 mmol	0.18 mmol
MW	163.03	101.12	220.16
Mass	32.61 mg	40.45 mg	40.07 mg

$$\mathbf{E-factor} = \frac{\Sigma \text{MW of stoichiometric reactants} - \Sigma \text{MW of desired products}}{\Sigma \text{MW of desired products}}$$

$$= \frac{32.61 + 40.45 - 40.07}{40.07}$$

$= 0.82$

Table S5. Penalty points for the synthesis of ureas system

EcoScale penalty points	Factor	Penalty
1.yield	91%	4.5
2.price	3-phenyl-1,4,2-dioxazol-5-one	0
	Diisopropylamine	0
	CH_3OH	0
3.safety	3-phenyl-1,4,2-dioxazol-5-one	0
	Diisopropylamine	0
	CH_3OH	0
4.technical setup	Common setup	0
5.temperature/time heating,	Room temperature, < 24 h	1
6. workup and purification	Classical chromatography	10
total penalty points		***15.5***
EcoScale score		***84.5***

2.9 Determination of trace metallic iron (ICP-MS)

The ICP-MS experiment have been performed to confirm the presence of trace iron, as shown in Figure S5. Number 1: 3-phenyl-1,4,2-dioxazol-5-one **1a** (0.1 mmol) and triphenylphosphine **2a** (0.1 mmol) were reacted under standard conditions. Then, after the reaction, the acid solution was diluted; Number 2: 3-Phenyl-1,4,2-dioxazol-5-one **1a** (0.1 mmol) and diisopropylamine **4a** (0.2 mmol) were reacted under standard conditions. Then, after the reaction, the acid solution was diluted; Number 3: 3-Phenyl-1,4,2-dioxazol-5-one **1a**, diluted acid solution; Number 4: Triphenylphosphine **2a**, the acid solution was diluted; Number 5: Diisopropylamine **4a**, the acid solution was diluted. ICP-MS: The Agilent 7800 measures the iron content of the sample and the results are shown. In Figure S5, it can be seen that the elemental iron content of the sample 1-5 were C_{X1} = (26577.5+26584.0)/2 ug/L≈ 27 ppm, C_{X2} = (2965.2+2965.9)/2 ug/L≈ 3 ppm; C_{X3} = (122970.9+122451.0)/2 ug/L≈ 123 ppm; C_{X4} = (421226.2+418572.5)/2 ug/L≈ 420 ppm; C_{X5} = (857.9+857.9)/2 ug/L≈ 0.9 ppm, which proves that there is iron in the reaction system.

Test items	Fe							
Instrument model	ICP-MS:Agilent 7800							
Instrument parameters	Pump Rate	29r/min						
	Nebulizer Flow	0.86L/min						
	Auxiliary Gas	0.7L/min						
	Sample Flush Time	40s						
	RF Power	1300w						
Data calculation instructions	$Cx(mg/kg)=\frac{C_0(mg/L)\cdot f\cdot V_0(mL)\cdot 10^{-3}}{m(g)\cdot 10^{-3}}=\frac{C_1(mg/L)\cdot V_0(mL)\cdot 10^{-3}}{m(g)\cdot 10^{-3}}$ (1)							
	$W(\%)=\frac{C_x(mg/kg)}{10^6}\cdot 100\%$ (2)							
	m_0: when analyzing the sample, the mass of the sample taken, the data is recorded by the analytical balance							
	V_0, after the sample is digested, the volume of constant volume							
	f: dilution factor							
	Co: the concentration of the elements in the test solution, in mg/L, the data is obtained from the instrument test							
	C_1: the element concentration of the sample digestion solution stock solution, in mg/L, C1(mg/L)=Co(mg/L)*f							
	C_x: the final test result of the tested element, in mg/kg. Calculated from Equation 1							
	W(%): the final test result of the test element, expressed in percentage form, is calculated by the above formula 2							
	number 1: Take 0.1mL of sample and digest it to 50 mL, dilute and measure; number 2: Take 0.2 mL of sample and digest it to 10 mL, dilute and measure; number 5: Take 2 mL of sample and digest it to 10 mL, dilute and measure							
number	compound	m_0 (g)	V_0 (mL)	test element	C_0 (ug/L)	f	Digestion solution original solution element concentration C_1 (ug/L)	Sample element content C_x (ug/L)
1	phosphinimidic amide	/	/	Fe	53.155	500	26577.5	26577.5
1	phosphinimidic amide	/	/	Fe	53.168	500	26584.0	26584.0
2	ureas	/	/	Fe	59.303	50	2965.2	2965.2
2	ureas	/	/	Fe	59.317	50	2965.9	2965.9
3	3-phenyl-1,4,2-dioxazol-5-one (1a)	0.0515	25	Fe	253.320	1	253.3	122970.9
3	3-phenyl-1,4,2-dioxazol-5-one (1a)	0.0515	25	Fe	252.249	1	252.2	122451.0
4	triphenylphosphine (2a)	0.0683	25	Fe	115.079	10	1150.8	421226.2
4	triphenylphosphine (2a)	0.0683	25	Fe	114.354	10	1143.5	418572.5
5	diisopropylamine (4a)	/	/	Fe	171.577	5	857.9	857.9
5	diisopropylamine (4a)	/	/	Fe	171.584	5	857.9	857.9

Figure S5 ICP-MS trace iron determination experiment.

2.10 Adding additional iron catalyst experiments

When additional iron catalyst $FeCl_3$ (5 mol%) was added to the model reaction under standard conditions, the reaction time was shortened and the yield increased, indicating that the addition of additional iron catalyst can promote the reaction.

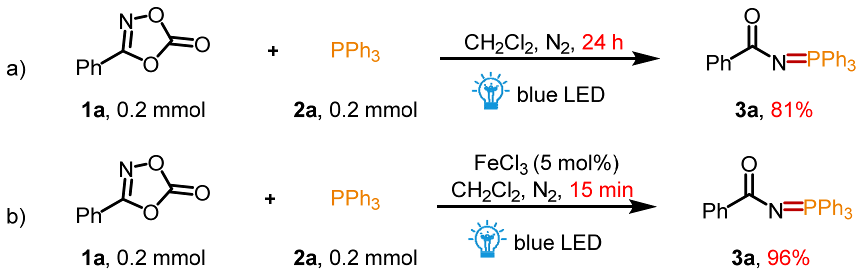

Scheme R1 Adding additional iron catalyst experiments.

3. Procedure for optimization studies

Table S6. Optimization for imidation of organophosphorus compounds.

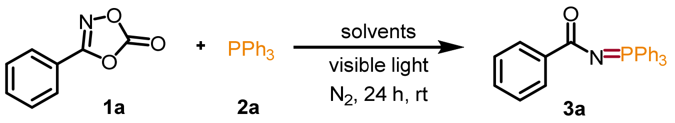

Entry	Solvent	Wavelength	Yield (%)
1	DCE	430 nm	11
2	1,4-dioxane	430 nm	23
3	CH_3OH	430 nm	14
4	Acetone	430 nm	23
5	DMF	430 nm	22
6	CH_3CN	430 nm	26
7	THF	430 nm	0
8	CH_2Cl_2	430 nm	81
9	CH_2Cl_2	460 nm	22
10	CH_2Cl_2	390 nm	63
11[b]	CH_2Cl_2	Green LED	0
12[c]	CH_2Cl_2	White LED	0
13[d]	CH_2Cl_2	430 nm	61
14[e]	CH_2Cl_2	430 nm	67
15[f]	CH_2Cl_2	--	0
16[g]	CH_2Cl_2	430 nm	<5

[a] Reaction condition: **1a** (0.1 mmol), **2a** (0.1 mmol) in solvent (1 mL) at room temperature for 24 h under the irradiation of 10 W 430 nm blue LED; yield is determined by ^{31}P NMR. N.D. = not detected. [b] Green LED (10 W). [c] White LED (10 W). [d] Experiment performed in the dark. [e] **1a** (0.2 mmol), [f] **2a** (0.2 mmol), [g] Air.

Table S7. Optimization for imidation of secondary amine compounds

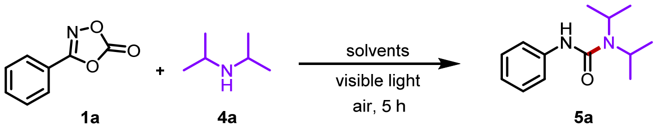

Entry	Solvent	Wavelength	Yield (%)
1	CH_2Cl_2	430 nm	76
2	DCE	430 nm	90
**3**	**CH_3OH**	**430 nm**	**96 (91)**
4	Toluene	430 nm	23
5	DMF	430 nm	25
6	CH_3CN	430 nm	63
7	THF	430 nm	0
8	1,4-dioxane	430 nm	31
9	CH_3OH	460 nm	87
10	CH_3OH	390 nm	86
11[b]	CH_3OH	--	68
12[c]	CH_3OH	430 nm	85
13[d]	CH_3OH	430 nm	95
14[e]	CH_3OH	430 nm	91
15[f]	CH_3OH	430 nm	84
16[g]	CH_3OH	430 nm	88

[a] Reaction condition: **1a** (0.1 mmol), **2a** (0.2 mmol) in solvent (1 mL) at room temperature for 5 h under the irradiation of 10W 430 nm blue LED. [b] Experiment performed in the dark. [c] **1a** (0.2 mmol), [d] **2a** (0.4 mmol), [e] N_2, [f] 3 h, [g] 7 h.

4. Characterization of compounds

N-(triphenyl-λ5-phosphanylidene)benzamide (3a)

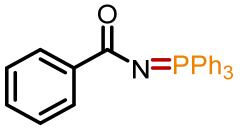

Purified by flash chromatography (silica gel, petroleum ether/ethyl acetate from 10:1 to 5:1, v/v) to give the desired product: white solid (59.7 mg, 78%). ^{1}H NMR (400 MHz, Chloroform-*d*) δ 8.45 – 8.38 (m, 2H), 7.94 – 7.85 (m, 6H), 7.62 – 7.55 (m, 3H), 7.54 – 7.41 (m, 9H); ^{13}C NMR (101 MHz, Chloroform-*d*) δ 176.3 (d, $J_{C\text{-}P}$ = 8.0 Hz), 138.7 (d, $J_{C\text{-}P}$ = 20.6 Hz), 133.2 (d, $J_{C\text{-}P}$ = 10.0 Hz), 132.3 (d, $J_{C\text{-}P}$ = 2.9 Hz), 130.7, 129.6 (d, $J_{C\text{-}P}$ = 2.6 Hz), 128.7 (d, $J_{C\text{-}P}$ = 12.4 Hz), 128.4 (d, $J_{C\text{-}P}$ = 99.7 Hz), 127.7; ^{31}P NMR (162 MHz, Chloroform-*d*) δ 20.71. The ^{1}H NMR values were in agreement with literature values.[29]

4-methyl-N-(triphenyl-λ5-phosphanylidene)benzamide (3b)

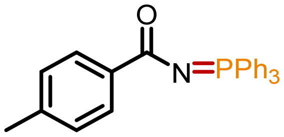

Purified by flash chromatography (silica gel, petroleum ether/ethyl acetate from 10:1 to 8:1, v/v) to give the desired product: white solid (69.0 mg, 87%). ^{1}H NMR (400 MHz, Chloroform-*d*) δ 8.30 (d, *J* = 8.2 Hz, 2H), 7.92 – 7.85 (m, 6H), 7.62 – 7.55 (m, 3H), 7.53 – 7.47 (m, 6H), 7.24 (d, *J* = 7.9 Hz, 2H), 2.42 (s, 3H); ^{13}C NMR (101 MHz, Chloroform-*d*) δ 176.5 (d, $J_{C\text{-}P}$ = 8.1 Hz), 140.9, 135.9 (d, $J_{C\text{-}P}$ = 20.5 Hz), 133.2 (d, $J_{C\text{-}P}$ = 9.7 Hz), 132.2 (d, $J_{C\text{-}P}$ = 2.9 Hz), 129.6 (d, $J_{C\text{-}P}$ = 2.6 Hz), 128.7 (d, $J_{C\text{-}P}$ = 12.1 Hz), 128.5 (d, $J_{C\text{-}P}$ = 99.6 Hz), 128.4, 21.6; ^{31}P NMR (162 MHz, Chloroform-*d*) δ 20.47. The ^{1}H NMR values were in agreement with literature values.[29]

4-(tert-butyl)-N-(triphenyl-λ5-phosphanylidene)benzamide (3c)

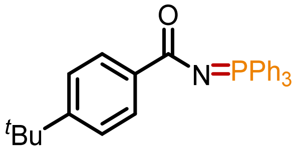

Purified by flash chromatography (silica gel, petroleum ether/ethyl acetate from 10:1 to 5:1, v/v) to give the desired product: white solid (55.0 mg, 63%). ^{1}H NMR (400 MHz, Chloroform-*d*) δ 8.33 (d, *J* = 8.5 Hz, 2H), 7.92 – 7.84 (m, 6H), 7.61 – 7.55 (m, 3H), 7.53 – 7.45 (m, 8H), 1.38 (s, 9H); ^{13}C NMR (101 MHz, Chloroform-*d*) δ 176.4 (d, $J_{C\text{-}P}$ = 8.1 Hz), 153.9, 136.0 (d, $J_{C\text{-}P}$ = 20.5 Hz), 133.2 (d, $J_{C\text{-}P}$ = 10.1 Hz), 132.2 (d, $J_{C\text{-}P}$ = 2.9 Hz), 129.4 (d, $J_{C\text{-}P}$ = 2.6 Hz), 128.7 (d, $J_{C\text{-}P}$ = 12.4 Hz), 128.5 (d, $J_{C\text{-}P}$ = 99.3 Hz), 124.6, 34.9, 31.4; ^{31}P NMR (162 MHz, Chloroform-*d*) δ 20.24. The ^{1}H NMR values were in agreement with literature values.[29]

4-methoxy-N-(triphenyl-λ5-phosphanylidene)benzamide (3d)

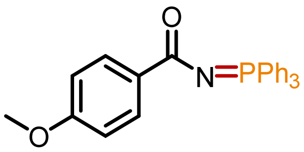

Purified by flash chromatography (silica gel, petroleum ether/ethyl acetate from 8:1 to 6:1, v/v) to give the desired product: white solid (79 mg, 96%). ^{1}H NMR (400 MHz, Chloroform-*d*) δ 8.35 (d, J = 8.8 Hz, 2H), 7.91 – 7.84 (m, 6H), 7.60 – 7.54 (m, 3H), 7.52 – 7.46 (m, 6H), 6.94 (d, J = 8.8 Hz, 2H), 3.85 (s, 3H); ^{13}C NMR (101 MHz, Chloroform-*d*) δ 176.1 (d, $J_{C\text{-}P}$ = 7.8 Hz), 161.8, 133.2 (d, $J_{C\text{-}P}$ = 9.9 Hz), 132.2 (d, $J_{C\text{-}P}$ = 2.9 Hz), 131.5, 131.4 (d, $J_{C\text{-}P}$ = 2.5 Hz), 128.7 (d, $J_{C\text{-}P}$ = 12.2 Hz), 128.5 (d, $J_{C\text{-}P}$ = 99.6 Hz), 112.8, 55.3; ^{31}P NMR (162 MHz, Chloroform-*d*) δ 20.30. The ^{1}H NMR values were in agreement with literature values.[29]

4-(trifluoromethyl)-N-(triphenyl-λ^5-phosphanylidene)benzamide (3e)

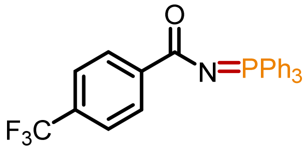

Purified by flash chromatography (silica gel, petroleum ether/ethyl acetate from 15:1 to 10:1, v/v) to give the desired product: white solid (87.6 mg, 94%). ^{1}H NMR (400 MHz, Chloroform-*d*) δ 8.49 (d, J = 8.1 Hz, 2H), 7.91 – 7.84 (m, 6H), 7.69 (d, J = 8.1 Hz, 2H), 7.64 – 7.57 (m, 3H), 7.56 – 7.49 (m, 6H); ^{13}C NMR (101 MHz, Chloroform-*d*) δ 174.8 (d, $J_{C\text{-}P}$ = 7.7 Hz), 142.0 (d, $J_{C\text{-}P}$ = 21.0 Hz), 133.2 (d, $J_{C\text{-}P}$ = 10.3 Hz), 132.5 (d, $J_{C\text{-}P}$ = 2.9 Hz), 132.2 (q, $J_{C\text{-}F}$ = 32.0 Hz), 129.8 (d, $J_{C\text{-}P}$ = 2.5 Hz), 128.8 (d, $J_{C\text{-}P}$ = 12.4 Hz), 127.9 (d, $J_{C\text{-}P}$ = 99.7 Hz), 124.7 (q, $J_{C\text{-}F}$ = 3.8 Hz), 124.3 (q, $J_{C\text{-}F}$ = 272.4 Hz); ^{31}P NMR (162 MHz, Chloroform-*d*) δ 21.40; ^{19}F NMR (376 MHz, Chloroform-*d*) δ -62.47. The ^{1}H NMR values were in agreement with literature values.[29]

4-fluoro-N-(triphenyl-λ^5-phosphanylidene)benzamide (3f)

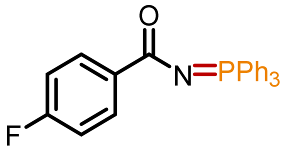

Purified by flash chromatography (silica gel, petroleum ether/ethyl acetate from 5:1 to 4:1, v/v) to give the desired product: white solid (57.5 mg, 73%). ^{1}H NMR (400 MHz, Chloroform-*d*) δ 8.38 (dd, J = 8.7, 5.9 Hz, 2H), 7.90 – 7.82 (m, 6H), 7.61 – 7.56 (m, 3H), 7.55 – 7.46 (m, 6H), 7.08 (t, J = 8.8 Hz, 2H); ^{13}C NMR (101 MHz, Chloroform-*d*) δ 175.3 (d, $J_{C\text{-}P}$ = 8.0 Hz), 164.7 (d, $J_{C\text{-}F}$ = 249.4 Hz), 134.9 (d, $J_{C\text{-}P}$ = 20.1 Hz), 133.2 (d, $J_{C\text{-}P}$ = 10.1 Hz), 132.3 (d, $J_{C\text{-}P}$ = 2.9 Hz), 131.8 (dd, $J_{C\text{-}F}$ = 8.9, $J_{C\text{-}P}$ = 2.3 Hz), 128.7 (d, $J_{C\text{-}P}$ = 12.3 Hz), 128.2 (d, $J_{C\text{-}P}$ = 99.7 Hz), 114.4 (d, $J_{C\text{-}F}$ = 21.4 Hz); ^{31}P NMR (162 MHz, Chloroform-*d*) δ 20.87; ^{19}F NMR (376 MHz, Chloroform-*d*) δ -110.66. The ^{1}H NMR values were in agreement with literature values.[29]

4-chloro-N-(triphenyl-λ^5-phosphanylidene)benzamide (3g)

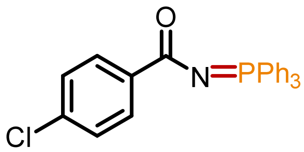

Purified by flash chromatography (silica gel, petroleum ether/ethyl acetate from 5:1 to 4:1, v/v) to give the desired product: white solid (81.8 mg, 98%). ^{1}H NMR (400 MHz, Chloroform-*d*) δ 8.33 (d, J = 8.5 Hz, 2H), 7.91 – 7.82 (m, 6H), 7.62 – 7.55 (m, 3H), 7.54 – 7.48 (m, 6H), 7.39 (d, J = 8.5 Hz, 2H); ^{13}C NMR (101 MHz, Chloroform-*d*) δ 175.2 (d, $J_{C\text{-}P}$ = 7.9 Hz), 137.2 (d, $J_{C\text{-}P}$ = 21.2 Hz), 136.8, 133.2 (d, $J_{C\text{-}P}$ = 9.7 Hz), 132.4 (d, $J_{C\text{-}P}$ = 2.9 Hz), 131.1 (d, $J_{C\text{-}P}$ = 2.3 Hz), 128.8 (d, $J_{C\text{-}P}$ = 12.4 Hz), 128.1 (d, $J_{C\text{-}P}$ = 99.7 Hz), 127.8; ^{31}P NMR (162 MHz, Chloroform-*d*) δ 21.05. The ^{1}H NMR values were in agreement with literature values.[29]

4-cyano-N-(triphenyl-λ^5-phosphaneylidene)benzamide (3h)

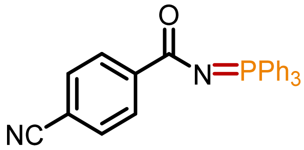

Purified by flash chromatography (silica gel, petroleum ether/ethyl acetate from 4:1 to 3:1, v/v) to give the desired product: white solid (57.6 mg, 71%), mp 185.5 – 187.1 °C. ^{1}H NMR (400 MHz, Chloroform-*d*) δ 8.44 (d, J = 8.1 Hz, 2H), 7.89 – 7.80 (m, 6H), 7.70 (d, J = 8.3 Hz, 2H), 7.64 – 7.57 (m, 3H), 7.56 – 7.49 (m, 6H); ^{13}C NMR (101 MHz, Chloroform-*d*) δ 174.2 (d, $J_{C\text{-}P}$ = 8.1 Hz), 142.8 (d, $J_{C\text{-}P}$ = 21.3 Hz), 133.1 (d, $J_{C\text{-}P}$ = 9.7 Hz), 132.6 (d, $J_{C\text{-}P}$ = 3.0 Hz), 131.6, 130.0 (d, $J_{C\text{-}P}$ = 2.3 Hz), 128.9 (d, $J_{C\text{-}P}$ = 12.4 Hz), 127.7 (d, $J_{C\text{-}P}$ = 99.8 Hz), 119.1, 113.8; ^{31}P NMR (162 MHz, Chloroform-*d*) δ 21.79. HRMS (ESI-TOF) *m/z*: $[M + H]^+$ calcd for $C_{26}H_{20}N_2OP$, 407.1308; found, 407.1309.

3-methoxy-N-(triphenyl-λ^5-phosphanylidene)benzamide (3i)

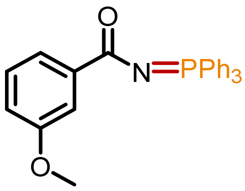

Purified by flash chromatography (silica gel, petroleum ether/ethyl acetate from 5:1 to 4:1, v/v) to give the desired product: white solid (62.4 mg, 76%). ^{1}H NMR (400 MHz, Chloroform-*d*) δ 8.05 (d, J = 7.6 Hz, 1H), 7.93 – 7.83 (m, 7H), 7.61 – 7.55 (m, 3H), 7.54 – 7.47 (m, 6H), 7.35 (t, J = 7.9 Hz, 1H), 7.06 – 7.01 (m, 1H), 3.88 (s, 3H); ^{13}C NMR (101 MHz, Chloroform-*d*) δ 176.1 (d, $J_{C\text{-}P}$ = 8.1 Hz), 159.3, 140.2 (d, $J_{C\text{-}P}$ = 20.5 Hz), 133.2 (d, $J_{C\text{-}P}$ = 9.7 Hz), 132.3 (d, $J_{C\text{-}P}$ = 2.9 Hz), 128.7 (d, $J_{C\text{-}P}$ = 12.5 Hz), 128.3 (d, $J_{C\text{-}P}$ = 99.2 Hz), 122.3 (d, $J_{C\text{-}P}$ = 2.6 Hz), 117.4, 113.8 (d, $J_{C\text{-}P}$ = 2.9 Hz), 55.4; ^{31}P NMR (162 MHz, Chloroform-*d*) δ 20.70. The ^{1}H NMR values were in agreement with literature values.[29]

3-(trifluoromethyl)-N-(triphenyl-λ^5-phosphanylidene)benzamide (3j)

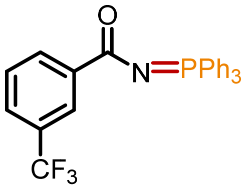

Purified by flash chromatography (silica gel, petroleum ether/ethyl acetate from 6:1 to 5:1, v/v) to give the desired product: white solid (65.1 mg, 73%). ^{1}H NMR (400 MHz, Chloroform-*d*) δ 8.67 (s, 1H), 8.57 (d, *J* = 7.7 Hz, 1H), 7.92 – 7.84 (m, 6H), 7.72 (d, *J* = 8.1 Hz, 1H), 7.64 – 7.58 (m, 3H), 7.57 – 7.50 (m, 7H); ^{13}C NMR (101 MHz, Chloroform-*d*) δ 174.7 (d, $J_{C\text{-}P}$ = 7.7 Hz), 139.5 (d, $J_{C\text{-}P}$ = 21.2 Hz), 133.2 (d, $J_{C\text{-}P}$ = 10.2 Hz), 132.8, 132.4 (d, $J_{C\text{-}P}$ = 3.0 Hz), 130.1 (q, $J_{C\text{-}F}$ = 32.2 Hz), 128.8 (d, $J_{C\text{-}P}$ = 12.4 Hz), 128.2, 128.0 (d, $J_{C\text{-}P}$ = 99.8 Hz), 127.1 (q, $J_{C\text{-}F}$ = 3.8 Hz), 126.5 (q, $J_{C\text{-}F}$ = 3.5 Hz), 123.0 (q, $J_{C\text{-}F}$ = 272.2 Hz); ^{31}P NMR (162 MHz, Chloroform-*d*) δ 21.62; ^{19}F NMR (376 MHz, Chloroform-*d*) δ -62.33. The ^{1}H NMR values were in agreement with literature values.[29]

3-fluoro-N-(triphenyl-λ^{5}-phosphanylidene)benzamide (3k)

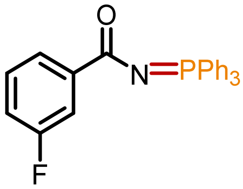

Purified by flash chromatography (silica gel, petroleum ether/ethyl acetate from 5:1 to 4:1, v/v) to give the desired product: white solid (48.7 mg, 61%), mp 154.6 – 156.5 °C. ^{1}H NMR (400 MHz, Chloroform-*d*) δ 8.14 (d, *J* = 7.7 Hz, 1H), 8.10 – 8.05 (m, 1H), 7.91 – 7.82 (m, 6H), 7.63 – 7.56 (m, 3H), 7.55 – 7.48 (m, 6H), 7.42 – 7.34 (m, 1H), 7.19 – 7.13 (m, 1H); ^{13}C NMR (101 MHz, Chloroform-*d*) δ 175.0 (d, $J_{C\text{-}P}$ = 7.4 Hz), 162.6 (d, $J_{C\text{-}F}$ = 244.8 Hz), 141.2 (d, $J_{C\text{-}F}$ = 21.3 Hz), 133.2 (d, $J_{C\text{-}P}$ = 10.0 Hz), 132.4 (d, $J_{C\text{-}F}$ = 2.9 Hz), 129.1 (d, $J_{C\text{-}P}$ = 7.7 Hz), 128.8 (d, $J_{C\text{-}P}$ = 12.4 Hz), 128.1 (d, $J_{C\text{-}P}$ = 99.8 Hz), 125.1 (d, $J_{C\text{-}P}$ = 2.8 Hz), 117.5 (d, $J_{C\text{-}P}$ = 21.6 Hz), 116.4 (dd, $J_{C\text{-}F}$ = 22.2, $J_{C\text{-}P}$ = 2.6 Hz); ^{31}P NMR (162 MHz, Chloroform-*d*) δ 21.13; ^{19}F NMR (376 MHz, Chloroform-*d*) δ -114.38. HRMS (ESI-TOF) *m/z*: $[M + H]^{+}$ calcd for $C_{25}H_{20}FNOP$, 400.1261; found, 400.1261.

3-chloro-N-(triphenyl-λ^{5}-phosphanylidene)benzamide (3l)

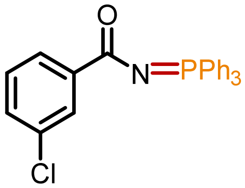

Purified by flash chromatography (silica gel, petroleum ether/ethyl acetate from 10:1 to 8:1, v/v) to give the desired product: colorless liquid (56.7 mg, 64%). ^{1}H NMR (400 MHz, Chloroform-*d*) δ 8.40 – 8.36 (m, 1H), 8.23 (d, *J* = 7.8 Hz, 1H), 7.90 – 7.82 (m, 6H), 7.63 – 7.57 (m, 3H), 7.55 – 7.48 (m, 6H), 7.46 – 7.41 (m, 1H), 7.37 – 7.32 (m, 1H); ^{13}C NMR (101 MHz, Chloroform-*d*) δ 174.9 (d, $J_{C\text{-}P}$ = 8.0 Hz), 140.6 (d, $J_{C\text{-}P}$ = 21.2 Hz), 133.7, 133.2 (d, $J_{C\text{-}P}$ = 10.2 Hz), 132.4 (d, $J_{C\text{-}P}$ = 2.6 Hz), 130.6, 129.8 (d, $J_{C\text{-}P}$ = 2.7 Hz), 129.0, 128.8 (d, $J_{C\text{-}P}$ = 12.0 Hz), 128.0 (d, $J_{C\text{-}P}$ = 99.8 Hz), 127.6 (d, $J_{C\text{-}P}$ = 2.2 Hz); ^{31}P NMR (162 MHz, Chloroform-*d*) δ 21.40. HRMS (ESI-TOF) *m/z*: $[M + H]^{+}$ calcd for $C_{25}H_{20}ClNOP$, 416.0966; found, 416.0963.

2-fluoro-N-(triphenyl-λ^5-phosphanylidene)benzamide (3m)

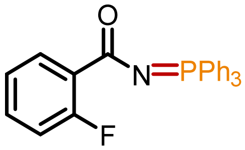

Purified by flash chromatography (silica gel, petroleum ether/ethyl acetate from 6:1 to 5:1, v/v) to give the desired product: white solid (37.5 mg, 47%). ^{1}H NMR (400 MHz, Chloroform-*d*) δ 8.22 – 8.16 (m, 1H), 7.93 – 7.84 (m, 6H), 7.61 – 7.55 (m, 3H), 7.53 – 7.47 (m, 6H), 7.41 – 7.34 (m, 1H), 7.18 – 7.06 (m, 2H); ^{13}C NMR (101 MHz, Chloroform-*d*) δ 174.1 (dd, J_{C-P} = 7.9 Hz, J_{C-F} = 3.6 Hz), 161.9 (d, J_{C-F} = 255.2 Hz), 133.2 (d, J_{C-P} = 10.1 Hz), 132.4 (dd, J_{C-P} = 2.3 Hz, J_{C-F} = 2.2 Hz), 132.3 (d, J_{C-P} = 2.9 Hz), 131.7 (d, J_{C-F} = 8.8 Hz), 128.7 (d, J_{C-P} = 12.4 Hz), 128.0 (d, J_{C-P} = 99.3 Hz), 127.5 (dd, J_{C-P} = 21.4 Hz, J_{C-F} = 9.9 Hz), 123.3 (d, J_{C-F} = 3.8 Hz), 116.5 (d, J_{C-F} = 23.4 Hz); ^{31}P NMR (162 MHz, Chloroform-*d*) δ 20.39; ^{19}F NMR (376 MHz, Chloroform-*d*) δ -111.60. The ^{1}H NMR values were in agreement with literature values.[29]

2-chloro-N-(triphenyl-λ^5-phosphanylidene)benzamide (3n)

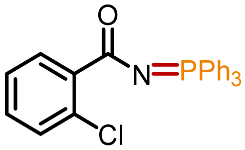

Purified by flash chromatography (silica gel, petroleum ether/ethyl acetate from 8:1 to 6:1, v/v) to give the desired product: white solid (35.0 mg, 42%), mp 196.6 – 197.8 °C. ^{1}H NMR (400 MHz, Chloroform-*d*) δ 8.43 – 8.36 (m, 2H), 7.92 – 7.84 (m, 6H), 7.62 – 7.56 (m, 3H), 7.54 – 7.48 (m, 6H), 7.46 – 7.41 (m, 2H); ^{13}C NMR (101 MHz, Chloroform-*d*) δ 176.4 (d, J_{C-P} = 8.6 Hz), 138.6 (d, J_{C-P} = 20.6 Hz), 133.2 (d, J_{C-P} = 9.7 Hz), 132.3 (d, J_{C-P} = 2.9 Hz), 130.7, 129.6 (d, J_{C-P} = 2.3 Hz), 128.7 (d, J_{C-P} = 12.3 Hz), 128.4 (d, J_{C-P} = 99.5 Hz), 127.7; ^{31}P NMR (162 MHz, Chloroform-*d*) δ 20.72. HRMS (ESI-TOF) *m/z*: $[M + H]^+$ calcd for $C_{25}H_{20}ClNOP$, 416.0966; found, 416.0969.

N-(triphenyl-λ^5-phosphanylidene)thiophene-2-carboxamide (3o)

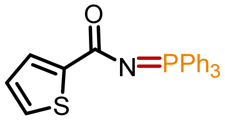

Purified by flash chromatography (silica gel, petroleum ether/ethyl acetate from 5:1 to 3:1, v/v) to give the desired product: brown solid (33.3 mg, 43%). ^{1}H NMR (400 MHz, Chloroform-*d*) δ 7.89 – 7.82 (m, 6H), 7.80 (dd, *J* = 3.6, 1.2 Hz, 1H), 7.62 – 7.56 (m, 3H), 7.53 – 7.47 (m, 6H), 7.40 (dd, *J* = 5.0, 1.2 Hz, 1H), 7.10 – 7.01 (m, 1H); ^{13}C NMR (101 MHz, Chloroform-*d*) δ 171.1 (d, J_{C-P} = 7.0 Hz), 145.2 (d, J_{C-P} = 24.3 Hz), 133.2 (d, J_{C-P} = 9.7 Hz), 132.3 (d, J_{C-P} = 2.9 Hz), 130.2 (d, J_{C-P} = 2.8 Hz), 129.6, 128.7 (d, J_{C-P} = 12.4 Hz), 128.1 (d, J_{C-P} = 99.7 Hz), 127.3; ^{31}P NMR (162 MHz, Chloroform-*d*) δ 19.47. The ^{1}H NMR values were in agreement with literature values.[29]

N-(triphenyl-λ^5-phosphanylidene)furan-2-carboxamide (3p)

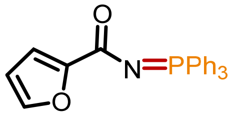

Purified by flash chromatography (silica gel, petroleum ether/ethyl acetate from 4:1 to 3:1, v/v) to give the desired product: white solid (37.2 mg, 50%). ^{1}H NMR (400 MHz, Chloroform-*d*) δ 7.87 – 7.79 (m, 6H), 7.59 – 7.53 (m, 3H), 7.51 – 7.45 (m, 7H), 7.17 (d, J = 3.3 Hz, 1H), 6.44 (dd, J = 3.2, 1.7 Hz, 1H); ^{13}C NMR (101 MHz, Chloroform-*d*) δ 168.0 (d, J_{C-P} = 6.7 Hz), 152.8 (d, J_{C-P} = 26.0 Hz), 144.0, 133.2 (d, J_{C-P} = 10.1 Hz), 132.4, 128.7 (d, J_{C-P} = 12.4 Hz), 127.9 (d, J_{C-P} = 100.2 Hz), 114.3, 111.3; ^{31}P NMR (162 MHz, Chloroform-*d*) δ 21.86. The ^{1}H NMR values were in agreement with literature values.[29]

N-(tri-p-tolyl-λ⁵-phosphanylidene)benzamide (3q)

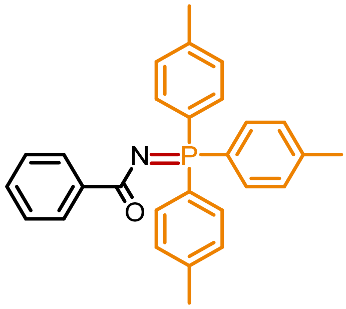

Purified by flash chromatography (silica gel, petroleum ether/ethyl acetate from 8:1 to 6:1, v/v) to give the desired product: white solid (77.1 mg, 91%). ^{1}H NMR (400 MHz, Chloroform-*d*) δ 8.42 (d, J = 7.8 Hz, 2H), 7.79 (dd, J = 12.2, 7.7 Hz, 6H), 7.48 – 7.41 (m, 3H), 7.32 (dd, J = 8.3, 2.8 Hz, 6H), 2.43 (s, 9H); ^{13}C NMR (101 MHz, Chloroform-*d*) δ 176.2 (d, J_{C-P} = 8.0 Hz), 142.7 (d, J_{C-P} = 2.9 Hz), 138.9 (d, J_{C-P} = 20.8 Hz), 133.2 (d, J_{C-P} = 10.3 Hz), 130.6, 129.6 (d, J_{C-P} = 2.3 Hz), 129.4 (d, J_{C-P} = 12.9 Hz), 127.6, 125.4 (d, J_{C-P} = 102.0 Hz), 21.7; ^{31}P NMR (162 MHz, Chloroform-*d*) δ 20.63. The ^{1}H NMR values were in agreement with literature values.[29]

N-(tri-p-methoxyphenyl-λ⁵-phosphanylidene)benzamide (3r)

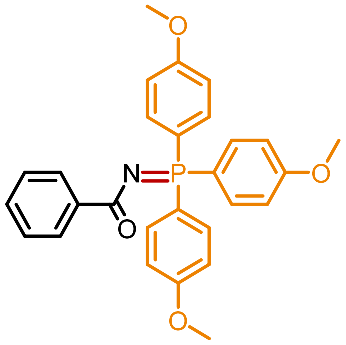

Purified by flash chromatography (silica gel, petroleum ether/ethyl acetate from 5:1 to 4:1, v/v) to give the desired product: white solid (56.7 mg, 60%). ^{1}H NMR (400 MHz, Chloroform-*d*) δ 8.40 – 8.34 (m, 2H), 7.78 (dd, J = 11.7, 8.8 Hz, 6H), 7.46 – 7.38 (m, 3H), 7.00 (dd, J = 8.9, 2.3 Hz, 6H), 3.85 (s, 9H); ^{13}C NMR (101 MHz, Chloroform-*d*) δ 176.1 (d, J_{C-P} = 7.4 Hz), 162.6 (d, J_{C-P} = 2.9 Hz), 139.0 (d, J_{C-P} = 20.4 Hz), 135.0 (d, J_{C-P} = 11.1 Hz), 130.5, 129.5 (d, J_{C-P} = 2.3 Hz), 127.6, 119.9 (d, J_{C-P} = 106.5 Hz), 114.3 (d, J_{C-P} = 13.3 Hz), 55.4; ^{31}P NMR (162 MHz, Chloroform-*d*) δ

19.68. The ^{1}H NMR values were in agreement with literature values.[29]

N-(tris(4-fluorophenyl)-λ⁵-phosphanylidene)benzamide (3s)

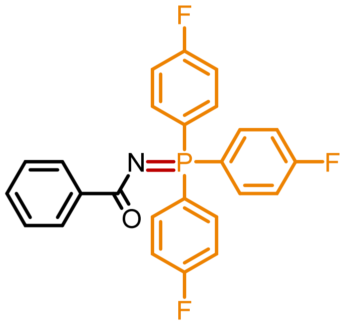

Purified by flash chromatography (silica gel, petroleum ether/ethyl acetate from 15:1 to 10:1, v/v) to give the desired product: white solid (44.2 mg, 51%). ^{1}H NMR (400 MHz, Chloroform-*d*) δ 8.34 – 8.29 (m, 2H), 7.90 – 7.81 (m, 6H), 7.50 – 7.41 (m, 3H), 7.26 – 7.19 (m, 6H); ^{13}C NMR (101 MHz, Chloroform-*d*) δ 176.5 (d, $J_{C\text{-}P}$ = 8.1 Hz), 165.4 (dd, $J_{C\text{-}F}$ = 255.1 Hz, $J_{C\text{-}P}$ = 3.2 Hz), 138.1 (d, $J_{C\text{-}P}$ = 20.6 Hz), 135.6 (dd, $J_{C\text{-}F}$ = 11.7 Hz, $J_{C\text{-}P}$ = 8.8 Hz), 131.0, 129.5 (d, $J_{C\text{-}P}$ = 2.7 Hz), 127.8, 123.9 (dd, $J_{C\text{-}P}$ = 103.9 Hz, $J_{C\text{-}F}$ = 3.3 Hz), 116.4 (dd, $J_{C\text{-}F}$ = 21.6 Hz, $J_{C\text{-}P}$ = 13.6 Hz); ^{31}P NMR (162 MHz, Chloroform-*d*) δ 18.98; ^{19}F NMR (376 MHz, Chloroform-*d*) δ -105.30. The ^{1}H NMR values were in agreement with literature values.[29]

N-(tris(4-chlorophenyl)-λ⁵-phosphanylidene)benzamide (3t)

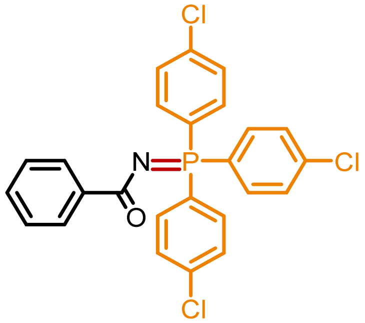

Purified by flash chromatography (silica gel, petroleum ether/ethyl acetate from 20:1 to 15:1, v/v) to give the desired product: white solid (80.5 mg, 83%). ^{1}H NMR (400 MHz, Chloroform-*d*) δ 8.36 – 8.30 (m, 2H), 7.79 (dd, *J* = 12.0, 8.4 Hz, 6H), 7.54 – 7.47 (m, 7H), 7.43 (dd, *J* = 8.1, 6.2 Hz, 2H); ^{13}C NMR (101 MHz, Chloroform-*d*) δ 176.7 (d, $J_{C\text{-}P}$ = 8.0 Hz), 139.5 (d, $J_{C\text{-}P}$ = 3.6 Hz), 137.9 (d, $J_{C\text{-}P}$ = 20.5 Hz), 134.4 (d, $J_{C\text{-}P}$ = 11.0 Hz), 131.1, 129.5 (d, $J_{C\text{-}P}$ = 2.5 Hz), 129.4 (d, $J_{C\text{-}P}$ = 12.8 Hz), 127.8, 126.2 (d, $J_{C\text{-}P}$ = 102.0 Hz); ^{31}P NMR (162 MHz, Chloroform-*d*) δ 19.49. The ^{1}H NMR values were in agreement with literature values.[29]

N-(tris(3-methoxyphenyl)-λ⁵-phosphaneylidene)benzamide (3u)

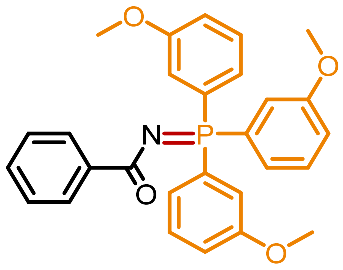

Purified by flash chromatography (silica gel, petroleum ether/ethyl acetate from 5:1 to 4:1, v/v) to give the desired product: white solid (48.5 mg, 52%), mp 146.3 – 147.7 °C. ^{1}H NMR (400 MHz, Chloroform-*d*) δ 8.41 – 8.36 (m, 2H), 7.52 – 7.47 (m, 3H), 7.46 – 7.34 (m, 9H), 7.12 – 7.07 (m, 3H), 3.79 (s, 9H); ^{13}C NMR (101 MHz, Chloroform-*d*) δ 176.2 (d, J_{C-P} = 7.9 Hz), 159.6 (d, J_{C-P} = 15.4 Hz), 138.7 (d, J_{C-P} = 20.6 Hz), 130.7, 129.9 (d, J_{C-P} = 14.6 Hz), 129.6 (d, J_{C-P} = 99.1 Hz), 129.5 (d, J_{C-P} = 2.4 Hz), 127.7, 125.4 (d, J_{C-P} = 9.7 Hz), 118.4 (d, J_{C-P} = 11.0 Hz), 118.1 (d, J_{C-P} = 2.9 Hz), 55.4; ^{31}P NMR (162 MHz, Chloroform-*d*) δ 21.38. HRMS (ESI-TOF) *m/z*: [M + H]$^+$ calcd for $C_{28}H_{27}NO_4P$, 472.1672; found, 472.1677.

N-(diphenyl(p-tolyl)-λ^5-phosphanylidene)benzamide (3v)

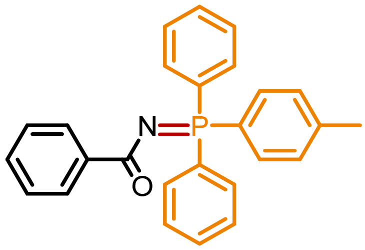

Purified by flash chromatography (silica gel, petroleum ether/ethyl acetate from 15:1 to 10:1, v/v) to give the desired product: white solid (71.1 mg, 90%). ^{1}H NMR (400 MHz, Chloroform-*d*) δ 8.45 – 8.37 (m, 2H), 7.93 – 7.85 (m, 4H), 7.81 – 7.74 (m, 2H), 7.61 – 7.55 (m, 2H), 7.53 – 7.42 (m, 7H), 7.32 (dd, *J* = 8.3, 2.9 Hz, 2H), 2.43 (s, 3H); ^{13}C NMR (101 MHz, Chloroform-*d*) δ 176.3 (d, J_{C-P} = 8.0 Hz), 142.9 (d, J_{C-P} = 2.9 Hz), 138.7 (d, J_{C-P} = 20.6 Hz), 133.3 (d, J_{C-P} = 10.5 Hz), 133.2 (d, J_{C-P} = 9.5 Hz), 132.2 (d, J_{C-P} = 3.0 Hz), 130.7, 129.6, 129.5 (d, J_{C-P} = 10.0 Hz), 128.7 (d, J_{C-P} = 12.3 Hz), 128.6 (d, J_{C-P} = 99.8 Hz), 127.7, 124.8 (d, J_{C-P} = 101.3 Hz), 21.7; ^{31}P NMR (162 MHz, Chloroform-*d*) δ 20.67. The ^{1}H NMR values were in agreement with literature values.[29]

N,N'-([1,1'-binaphthalene]-2,2'-diylbis(diphenyl-λ^5-phosphaneylylidene))dibenzamide (3w)

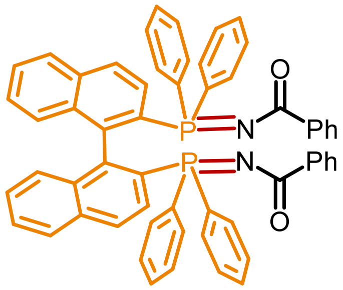

Purified by flash chromatography (silica gel, petroleum ether/ethyl acetate from 5:1 to 3:1, v/v) to

give the desired product: white solid (87.7 mg, 51%), mp 184.5 – 185.8 °C. ^{1}H NMR (400 MHz, Chloroform-*d*) δ 8.18 (d, *J* = 7.1 Hz, 4H), 7.78 – 7.69 (m, 6H), 7.57 – 7.46 (m, 7H), 7.44 – 7.29 (m, 13H), 7.28 – 7.21 (m, 3H), 7.20 – 7.07 (m, 7H), 6.41 (dd, *J* = 8.3, 5.0 Hz, 2H); ^{13}C NMR (101 MHz, Chloroform-*d*) δ 175.9 (d, J_{C-P} = 8.0 Hz), 159.6 (d, J_{C-P} = 2.0 Hz), 138.8 (d, J_{C-P} = 21.2 Hz), 135.1 (d, J_{C-P} = 7.1 Hz), 134.1 (d, J_{C-P} = 2.6 Hz), 133.3 (d, J_{C-P} = 10.5 Hz), 132.9 (d, J_{C-P} = 10.3 Hz), 132.1 (d, J_{C-P} = 3.0 Hz), 131.6 (d, J_{C-P} = 2.4 Hz), 130.4, 129.4 (d, J_{C-P} = 2.7 Hz), 128.5 (d, J_{C-P} = 12.5 Hz), 128.2 (d, J_{C-P} = 12.6 Hz), 127.8 (d, J_{C-P} = 104.7 Hz), 127.50, 127.45, 123.9 (d, J_{C-P} = 11.4 Hz), 121.0 (d, J_{C-P} = 6.8 Hz), 119.2 (d, J_{C-P} = 100.4 Hz); ^{31}P NMR (162 MHz, Chloroform-*d*) δ 19.95. HRMS (ESI-TOF) *m/z*: [M + H]$^+$ calcd for $C_{58}H_{43}N_2O_2P_2$, 861.2794; found, 861.2791.

N-(1,3-dioxo-1,3-diphenylpropan-2-yl)-4-methylbenzamide (7)

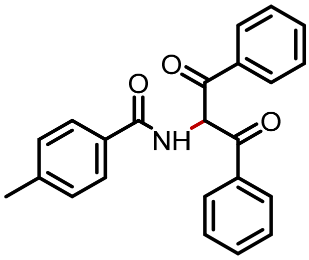

Purified by flash chromatography (silica gel, petroleum ether/ethyl acetate from 10:1 to 5:1, v/v) to give the desired product: white solid (41.4 mg, 58%). ^{1}H NMR (400 MHz, Chloroform-*d*) δ 8.15 – 8.09 (m, 4H), 7.79 (d, *J* = 8.2 Hz, 2H), 7.75 (d, *J* = 7.9 Hz, 1H), 7.63 – 7.58 (m, 2H), 7.50 – 7.45 (m, 4H), 7.26 (dd, *J* = 8.1, 2.9 Hz, 3H), 2.41 (s, 3H); ^{13}C NMR (101 MHz, Chloroform-*d*) δ 193.4, 166.8, 142.8, 134.6, 134.3, 130.4, 129.4, 129.2, 129.0, 127.4, 60.7, 21.5. The ^{1}H NMR values were in agreement with literature values.[29]

1,1-diisopropyl-3-phenylurea (5a)

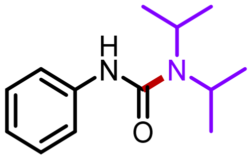

Purified by flash chromatography (silica gel, petroleum ether/ethyl acetate from 20:1 to 10:1, v/v) to give the desired product: white solid (40.1 mg, 91%). ^{1}H NMR (400 MHz, Chloroform-*d*) δ 7.39 (dd, *J* = 8.4, 1.3 Hz, 2H), 7.31 – 7.26 (m, 2H), 7.06 – 6.99 (m, 1H), 6.26 (s, 1H), 4.04 – 3.95 (m, 2H), 1.34 (d, *J* = 7.0 Hz, 12H); ^{13}C NMR (101 MHz, Chloroform-*d*) δ 154.6, 139.4, 128.8, 122.6, 119.7, 45.5, 21.5. The ^{1}H NMR values were in agreement with literature values.[34]

1,1-diisopropyl-3-(p-tolyl)urea (5b)

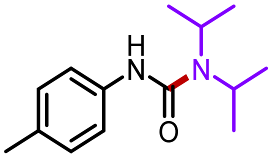

Purified by flash chromatography (silica gel, petroleum ether/ethyl acetate from 20:1 to 15:1, v/v) to give the desired product: white solid (44.9 mg, 96%). ^{1}H NMR (400 MHz, Chloroform-*d*) δ 7.27 (d, *J* = 8.3 Hz, 2H), 7.09 (d, *J* = 8.4 Hz, 2H), 6.18 (s, 1H), 4.04 – 3.94 (m, 2H), 2.30 (s, 3H), 1.33

(d, J = 6.9 Hz, 12H); ^{13}C NMR (101 MHz, Chloroform-*d*) δ 154.8, 136.8, 132.1, 129.3, 119.9, 45.4, 21.5, 20.7. The ^{1}H NMR values were in agreement with literature values.[61]

3-(4-(tert-butyl)phenyl)-1,1-diisopropylurea (5c)

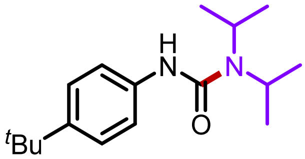

Purified by flash chromatography (silica gel, petroleum ether/ethyl acetate from 15:1 to 10:1, v/v) to give the desired product: white solid (49.1 mg, 89%), mp 115.1 – 116.7 °C. ^{1}H NMR (400 MHz, Chloroform-*d*) δ 7.33 – 7.29 (m, 4H), 6.17 (s, 1H), 4.05 – 3.96 (m, 2H), 1.34 (d, J = 6.9 Hz, 12H), 1.31 (s, 9H); ^{13}C NMR (101 MHz, Chloroform-*d*) δ 154.9, 145.6, 136.7, 125.7, 119.7, 45.4, 34.2, 31.4, 21.6. HRMS (ESI-TOF) *m/z*: $[M + H]^+$ calcd for $C_{17}H_{29}N_2O$, 277.2274; found, 277.2284.

1,1-diisopropyl-3-(4-methoxyphenyl)urea (5d)

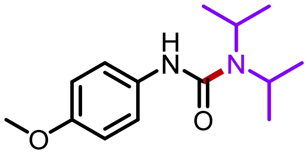

Purified by flash chromatography (silica gel, petroleum ether/ethyl acetate from 15:1 to 10:1, v/v) to give the desired product: white solid (49.0 mg, 98%). ^{1}H NMR (400 MHz, Chloroform-*d*) δ 7.27 (d, J = 8.9 Hz, 2H), 6.83 (d, J = 9.0 Hz, 2H), 6.13 (s, 1H), 4.01 – 3.92 (m, 2H), 3.77 (s, 3H), 1.32 (d, J = 6.9 Hz, 12H); ^{13}C NMR (101 MHz, Chloroform-*d*) δ 155.5, 155.1, 132.5, 122.0, 114.1, 55.5, 45.4, 21.5. The ^{1}H NMR values were in agreement with literature values.[60]

1,1-diisopropyl-3-(4-(trifluoromethyl)phenyl)urea (5e)

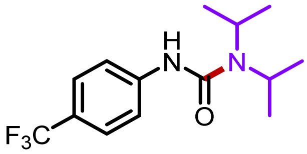

Purified by flash chromatography (silica gel, petroleum ether/ethyl acetate from 15:1 to 10:1, v/v) to give the desired product: white solid (44.2 mg, 79%), mp 151.9 – 153.1 °C. ^{1}H NMR (400 MHz, Chloroform-*d*) δ 7.57 – 7.46 (m, 4H), 6.43 (s, 1H), 4.07 – 3.92 (m, 2H), 1.35 (d, J = 6.9 Hz, 12H); ^{13}C NMR (101 MHz, Chloroform-*d*) δ 154.0, 142.6, 126.1 (q, $J_{C\text{-}F}$ = 3.8 Hz), 124.4 (q, $J_{C\text{-}F}$ = 271.1 Hz), 124.2 (q, $J_{C\text{-}F}$ = 32.7 Hz), 118.9, 45.7, 21.5; ^{19}F NMR (376 MHz, Chloroform-*d*) δ -61.81; HRMS (ESI-TOF) *m/z*: $[M + H]^+$ calcd for $C_{14}H_{20}F_3N_2O$, 289.1522; found, 289.1534.

3-(4-fluorophenyl)-1,1-diisopropylurea (5f)

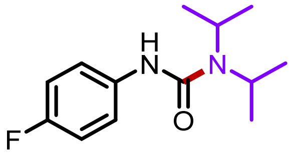

Purified by flash chromatography (silica gel, petroleum ether/ethyl acetate from 15:1 to 10:1, v/v) to give the desired product: white solid (36.7 mg, 77%), mp 134.5 – 135.7 °C. ^{1}H NMR (400 MHz,

Chloroform-*d*) δ 7.35 – 7.27 (m, 2H), 6.94 (t, J = 8.6 Hz, 2H), 6.31 (s, 1H), 4.03 – 3.86 (m, 2H), 1.30 (d, J = 6.9 Hz, 12H); ^{13}C NMR (101 MHz, Chloroform-*d*) δ 158.6 (d, $J_{C\text{-}F}$ = 241.2 Hz), 154.8, 135.4 (d, $J_{C\text{-}F}$ = 2.9 Hz), 121.8 (d, $J_{C\text{-}F}$ = 7.9 Hz), 115.2 (d, $J_{C\text{-}F}$ = 22.1 Hz), 45.6, 21.4; ^{19}F NMR (376 MHz, Chloroform-*d*) δ -120.90; HRMS (ESI-TOF) *m/z*: [M + H]$^+$ calcd for $C_{13}H_{20}FN_2O$, 239.1554; found, 239.1567.

3-(4-chlorophenyl)-1,1-diisopropylurea (5g)

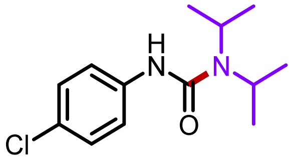

Purified by flash chromatography (silica gel, petroleum ether/ethyl acetate from 15:1 to 10:1, v/v) to give the desired product: white solid (49.8 mg, 98%). ^{1}H NMR (400 MHz, Chloroform-*d*) δ 7.32 (d, J = 8.9 Hz, 2H), 7.22 (d, J = 8.8 Hz, 2H), 6.29 (s, 1H), 4.03 – 3.90 (m, 2H), 1.32 (d, J = 6.9 Hz, 12H).; ^{13}C NMR (101 MHz, Chloroform-*d*) δ 154.4, 138.0, 128.7, 127.4, 121.0, 45.6, 21.5. The ^{1}H NMR values were in agreement with literature values.[62]

1,1-diisopropyl-3-(3-methoxyphenyl)urea (5h)

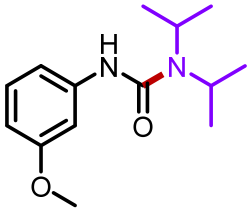

Purified by flash chromatography (silica gel, petroleum ether/ethyl acetate from 8:1 to 6:1, v/v) to give the desired product: white solid (43.6 mg, 87%). ^{1}H NMR (400 MHz, Chloroform-*d*) δ 7.20 – 7.14 (m, 2H), 6.85 (dd, J = 8.0, 1.2 Hz, 1H), 6.57 (dd, J = 8.2, 1.7 Hz, 1H), 6.27 (s, 1H), 4.05 – 3.95 (m, 2H), 3.81 (s, 3H), 1.33 (d, J = 6.9 Hz, 12H).; ^{13}C NMR (101 MHz, Chloroform-*d*) δ 160.2, 154.5, 140.7, 129.4, 111.7, 108.6, 105.1, 55.3, 45.4, 21.5. The ^{1}H NMR values were in agreement with literature values.[60]

1,1-diisopropyl-3-(3-(trifluoromethyl)phenyl)urea (5i)

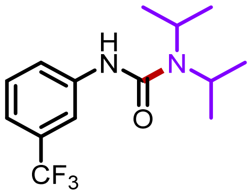

Purified by flash chromatography (silica gel, petroleum ether/ethyl acetate from 8:1 to 5:1, v/v) to give the desired product: white solid (21.3 mg, 37%), mp 151.2 – 152.7 °C. ^{1}H NMR (400 MHz, Chloroform-*d*) δ 7.67 (s, 1H), 7.62 – 7.55 (m, 1H), 7.39 (t, J = 7.9 Hz, 1H), 7.29 – 7.25 (m, 1H), 6.37 (s, 1H), 4.07 – 3.94 (m, 2H), 1.36 (d, J = 6.9 Hz, 12H).; ^{13}C NMR (101 MHz, Chloroform-*d*) δ 154.2, 139.9, 131.2 (q, $J_{C\text{-}F}$ = 271.6 Hz), 129.3, 122.7, 119.1 (q, $J_{C\text{-}F}$ = 3.7 Hz), 116.1 (q, $J_{C\text{-}F}$ = 4.0 Hz), 45.7, 21.5; ^{19}F NMR (376 MHz, Chloroform-*d*) δ -62.64. HRMS (ESI-TOF) *m/z*: [M + H]$^+$

calcd for $C_{14}H_{20}F_3N_2O$, 289.1522; found, 289.1536.

3-(3-fluorophenyl)-1,1-diisopropylurea (5j)

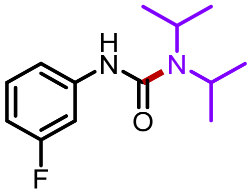

Purified by flash chromatography (silica gel, petroleum ether/ethyl acetate from 8:1 to 6:1, v/v) to give the desired product: white solid (23.9 mg, 50%), mp 118.5 – 119.8 °C. ^{1}H NMR (400 MHz, Chloroform-*d*) δ 7.40 – 7.32 (m, 1H), 7.23 – 7.14 (m, 1H), 7.00 (dd, J = 8.3, 2.1 Hz, 1H), 6.72 – 6.64 (m, 1H), 6.39 (s, 1H), 4.03 – 3.89 (m, 2H), 1.32 (d, J = 6.6 Hz, 12H); ^{13}C NMR (101 MHz, Chloroform-*d*) δ 163.2 (d, $J_{C\text{-}F}$ = 243.5 Hz), 154.2, 141.1 (d, $J_{C\text{-}F}$ = 11.2 Hz), 129.7 (d, $J_{C\text{-}F}$ = 9.6 Hz), 114.7 (d, $J_{C\text{-}F}$ = 2.9 Hz), 109.0 (d, $J_{C\text{-}F}$ = 21.3 Hz), 106.9 (d, $J_{C\text{-}F}$ = 26.4 Hz), 45.6, 21.5; ^{19}F NMR (376 MHz, Chloroform-*d*) δ -112.35. HRMS (ESI-TOF) *m/z*: $[M + H]^+$ calcd for $C_{13}H_{20}FN_2O$, 239.1554; found, 239.1564.

1,1-diisopropyl-3-(o-tolyl)urea (5k)

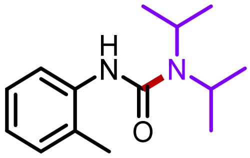

Purified by flash chromatography (silica gel, petroleum ether/ethyl acetate from 10:1 to 8:1, v/v) to give the desired product: white solid (43.0 mg, 92%), mp 136.8 – 138.7 °C. ^{1}H NMR (400 MHz, Chloroform-*d*) δ 7.77 (dd, J = 8.1, 1.2 Hz, 1H), 7.23 – 7.14 (m, 2H), 7.04 – 6.96 (m, 1H), 6.07 (s, 1H), 4.11 – 3.99 (m, 2H), 2.28 (s, 3H), 1.36 (d, J = 6.9 Hz, 12H); ^{13}C NMR (101 MHz, Chloroform-*d*) δ 154.8, 137.6, 130.3, 127.6, 126.7, 123.2, 122.3, 45.4, 21.5, 18.3. HRMS (ESI-TOF) *m/z*: $[M + H]^+$ calcd for $C_{14}H_{23}N_2O$, 235.1805; found, 235.1816.

3-(2-fluorophenyl)-1,1-diisopropylurea (5l)

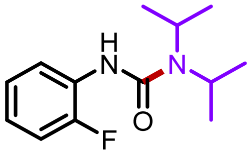

Purified by flash chromatography (silica gel, petroleum ether/ethyl acetate from 10:1 to 8:1, v/v) to give the desired product: white solid (40.0 mg, 84%), mp 109.1 – 110.7 °C. ^{1}H NMR (400 MHz, Chloroform-*d*) δ 8.24 – 8.16 (m, 1H), 7.13 – 7.02 (m, 2H), 6.99 – 6.90 (m, 1H), 6.58 (s, 1H), 4.12 – 4.03 (m, 2H), 1.35 (d, J = 6.9 Hz, 12H); ^{13}C NMR (101 MHz, Chloroform-*d*) δ 154.1, 152.3 (d, $J_{C\text{-}F}$ = 239.8 Hz), 128.0 (d, $J_{C\text{-}F}$ = 9.5 Hz), 124.5 (d, $J_{C\text{-}F}$ = 3.6 Hz), 122.0 (d, $J_{C\text{-}F}$ = 7.5 Hz), 121.1, 114.3 (d, $J_{C\text{-}F}$ = 19.2 Hz), 45.3, 21.4; ^{19}F NMR (376 MHz, Chloroform-*d*) δ -133.39. HRMS (ESI-TOF) *m/z*: $[M + H]^+$ calcd for $C_{13}H_{20}FN_2O$, 239.1554; found, 239.1564.

3-(2-chlorophenyl)-1,1-diisopropylurea (5m)

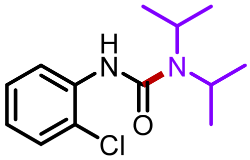

Purified by flash chromatography (silica gel, petroleum ether/ethyl acetate from 8:1 to 6:1, v/v) to give the desired product: white solid (49.6 mg, 95%). ^{1}H NMR (400 MHz, Chloroform-*d*) δ 7.41 – 7.37 (m, 2H), 7.29 (d, *J* = 8.6 Hz, 1H), 7.06 – 6.98 (m, 1H), 6.27 (s, 1H), 4.05 – 3.95 (m, 2H), 1.34 (d, *J* = 6.9 Hz, 12H); ^{13}C NMR (101 MHz, Chloroform-*d*) δ 154.6, 139.4, 128.8, 122.6, 119.7, 45.5, 21.5. The ^{1}H NMR values were in agreement with literature values.[62]

1,1-diethyl-3-phenylurea (5n)

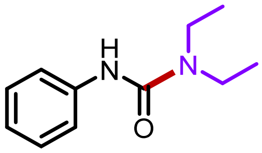

Purified by flash chromatography (silica gel, petroleum ether/ethyl acetate from 6:1 to 5:1, v/v) to give the desired product: white solid (36.8 mg, 96%). ^{1}H NMR (400 MHz, Chloroform-*d*) δ 7.43 – 7.39 (m, 2H), 7.31 – 7.25 (m, 2H), 7.02 (t, *J* = 7.3 Hz, 1H), 6.40 (s, 1H), 3.41 – 3.35 (m, 4H), 1.22 (t, *J* = 7.1 Hz, 6H); ^{13}C NMR (101 MHz, Chloroform-*d*) δ 154.7, 139.4, 128.8, 122.8, 119.9, 41.6, 13.9. The ^{1}H NMR values were in agreement with literature values.[63]

3-phenyl-1,1-dipropylurea (5o)

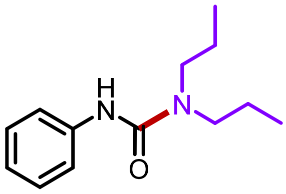

Purified by flash chromatography (silica gel, petroleum ether/ethyl acetate from 8:1 to 6:1, v/v) to give the desired product: white solid (35 mg, 80%). ^{1}H NMR (400 MHz, Chloroform-*d*) δ 7.43 – 7.37 (m, 2H), 7.31 – 7.25 (m, 2H), 7.06 – 6.97 (m, 1H), 6.41 (s, 1H), 3.31 – 3.24 (m, 4H), 1.71 – 1.61 (m, 4H), 0.96 (t, *J* = 7.4 Hz, 6H); ^{13}C NMR (101 MHz, Chloroform-*d*) δ 155.0, 139.4, 128.8, 122.7, 119.8, 49.4, 21.9, 11.4. The ^{1}H NMR values were in agreement with literature values.[34]

1,1-dibutyl-3-phenylurea (5p)

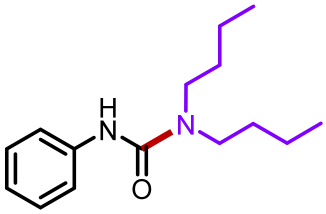

Purified by flash chromatography (silica gel, petroleum ether/ethyl acetate from 8:1 to 6:1, v/v) to give the desired product: white solid (41.6 mg, 84%). ^{1}H NMR (400 MHz, Chloroform-*d*) δ 7.44 – 7.38 (m, 2H), 7.30 – 7.25 (m, 2H), 7.05 – 6.99 (m, 1H), 6.38 (s, 1H), 3.34 – 3.28 (m, 4H), 1.65 – 1.58 (m, 4H), 1.43 – 1.34 (m, 4H), 0.98 (t, *J* = 7.3 Hz, 6H); ^{13}C NMR (101 MHz, Chloroform-*d*) δ

155.0, 139.4, 128.8, 122.7, 119.7, 47.5, 30.8, 20.2, 13.9. The ^{1}H NMR values were in agreement with literature values.[34]

1-ethyl-3-phenyl-1-propylurea (5q)

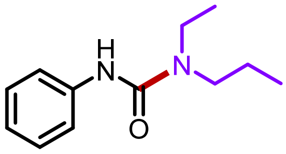

Purified by flash chromatography (silica gel, petroleum ether/ethyl acetate from 10:1 to 8:1, v/v) to give the desired product: colorless liquid (34.0 mg, 83%), mp 56.3 – 57.6 °C. ^{1}H NMR (400 MHz, Chloroform-*d*) δ 7.43 – 7.37 (m, 2H), 7.31 – 7.25 (m, 2H), 7.02 (t, *J* = 7.4 Hz, 1H), 6.39 (s, 1H), 3.42 – 3.34 (m, 2H), 3.30 – 3.24 (m, 2H), 1.71 – 1.61 (m, 2H), 1.22 (t, *J* = 7.1 Hz, 3H), 0.96 (t, *J* = 7.4 Hz, 3H); ^{13}C NMR (101 MHz, Chloroform-*d*) δ 154.9, 139.4, 128.8, 122.7, 119.8, 48.8, 42.1, 22.0, 13.8, 11.4; HRMS (ESI-TOF) *m/z*: $[M + H]^+$ calcd for $C_{12}H_{19}N_2O$, 207.1492; found, 207.1499.

1-cyclohexyl-1-ethyl-3-phenylurea (5r)

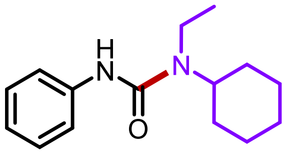

Purified by flash chromatography (silica gel, petroleum ether/ethyl acetate from 10:1 to 8:1, v/v) to give the desired product: white solid (44.0 mg, 89%), mp 123.5 – 124.7 °C. ^{1}H NMR (400 MHz, Chloroform-*d*) δ 7.44 – 7.39 (m, 2H), 7.31 – 7.25 (m, 2H), 7.01 (t, *J* = 7.3 Hz, 1H), 6.41 (s, 1H), 4.14 – 4.03 (m, 1H), 3.33 – 3.25 (m, 2H), 1.85 – 1.76 (m, 4H), 1.72 – 1.64 (m, 1H), 1.47 – 1.33 (m, 4H), 1.25 (t, *J* = 7.2 Hz, 3H), 1.18 – 1.06 (m, 1H); ^{13}C NMR (101 MHz, Chloroform-*d*) δ 154.8, 139.5, 128.8, 122.7, 119.9, 54.8, 36.9, 31.5, 26.0, 25.6, 16.1; HRMS (ESI-TOF) *m/z*: $[M + H]^+$ calcd for $C_{15}H_{23}N_2O$, 247.1805; found, 247.1815.

1,1-dicyclohexyl-3-phenylurea (5s)

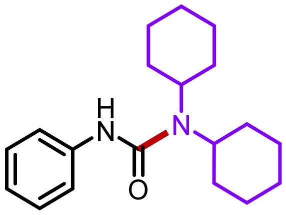

Purified by flash chromatography (silica gel, petroleum ether/ethyl acetate from 10:1 to 8:1, v/v) to give the desired product: white solid (54.0 mg, 90%). ^{1}H NMR (400 MHz, Chloroform-*d*) δ 7.40 – 7.36 (m, 2H), 7.31 – 7.25 (m, 2H), 7.04 – 6.98 (m, 1H), 6.32 (s, 1H), 3.55 – 3.45 (m, 2H), 1.89 – 1.81 (m, 6H), 1.80 – 1.75 (m, 6H), 1.69 (d, *J* = 13.0 Hz, 2H), 1.42 – 1.31 (m, 4H), 1.22 – 1.11 (m, 2H); ^{13}C NMR (101 MHz, Chloroform-*d*) δ 154.9, 139.4, 128.8, 122.5, 119.7, 55.5, 31.9, 26.4, 25.6. The ^{1}H NMR values were in agreement with literature values.[63]

N-phenyl-3,4-dihydroisoquinoline-2(1H)-carboxamide (5t)

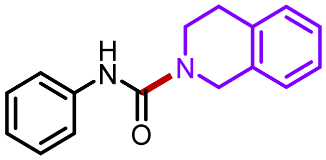

Purified by flash chromatography (silica gel, petroleum ether/ethyl acetate from 10:1 to 8:1, v/v) to give the desired product: white solid (45.1 mg, 89%). ^{1}H NMR (400 MHz, Chloroform-*d*) δ 7.46 – 7.42 (m, 2H), 7.32 – 7.27 (m, 2H), 7.25 – 7.17 (m, 3H), 7.15 – 7.10 (m, 1H), 7.05 (t, *J* = 7.4 Hz, 1H), 6.75 (s, 1H), 4.67 (s, 2H), 3.72 (t, *J* = 5.9 Hz, 2H), 2.91 (t, *J* = 5.9 Hz, 2H); ^{13}C NMR (101 MHz, Chloroform-*d*) δ 155.2, 139.2, 135.0, 133.3, 128.9, 128.4, 126.8, 126.5, 126.4, 123.1, 120.3, 45.8, 41.6, 29.0. The ^{1}H NMR values were in agreement with literature values.[34]

1-benzyl-1-ethyl-3-phenylurea (5u)

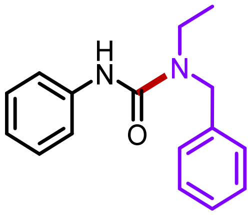

Purified by flash chromatography (silica gel, petroleum ether/ethyl acetate from 5:1 to 4:1, v/v) to give the desired product: colorless liquid (42.8 mg, 84%). ^{1}H NMR (400 MHz, Chloroform-*d*) δ 7.42 – 7.38 (m, 2H), 7.37 – 7.30 (m, 5H), 7.29 – 7.24 (m, 2H), 7.06 – 7.00 (m, 1H), 6.40 (s, 1H), 4.59 (s, 2H), 3.52 – 3.45 (m, 2H), 1.24 (t, *J* = 7.1 Hz, 3H); ^{13}C NMR (101 MHz, Chloroform-*d*) δ 155.4, 139.2, 137.7, 129.0, 128.8, 127.7, 127.1, 122.9, 119.9, 50.3, 42.5, 13.5. HRMS (ESI-TOF) *m/z*: $[M + H]^+$ calcd for $C_{16}H_{19}N_2O$, 255.1492; found, 255.1500.

1-benzyl-1-isopropyl-3-phenylurea (5v)

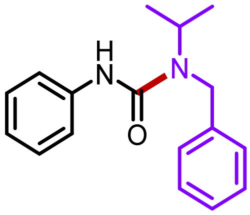

Purified by flash chromatography (silica gel, petroleum ether/ethyl acetate from 10:1 to 8:1, v/v) to give the desired product: white solid (48.8 mg, 91%), mp 108.8 – 109.9 °C. ^{1}H NMR (400 MHz, Chloroform-*d*) δ 7.45 – 7.37 (m, 4H), 7.37 – 7.32 (m, 1H), 7.25 – 7.19 (m, 4H), 7.02 – 6.96 (m, 1H), 6.36 (s, 1H), 4.86 – 4.73 (m, 1H), 4.47 (s, 2H), 1.24 (d, *J* = 6.8 Hz, 6H); ^{13}C NMR (101 MHz, Chloroform-*d*) δ 155.8, 139.3, 138.2, 129.2, 128.7, 127.8, 126.4, 122.8, 119.8, 46.4, 45.5, 20.8. HRMS (ESI-TOF) *m/z*: $[M + H]^+$ calcd for $C_{17}H_{21}N_2O$, 269.1648; found, 269.1657.

1,3-diphenylurea (5w)

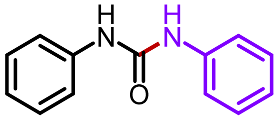

Purified by flash chromatography (silica gel, petroleum ether/ethyl acetate from 20:1 to 10:1, v/v)

to give the desired product: white solid (22.5 mg, 52%). ^{1}H NMR (400 MHz, DMSO-d_6) δ 8.7 (s, 2H), 7.5 (dd, J = 8.6, 1.2 Hz, 4H), 7.3 – 7.2 (m, 4H), 7.0 – 6.9 (m, 2H); ^{13}C NMR (101 MHz, DMSO-d_6) δ 153.0, 140.2, 129.2, 122.3, 118.6. The ^{1}H NMR values were in agreement with literature values.[32]

5. NMR copies of products

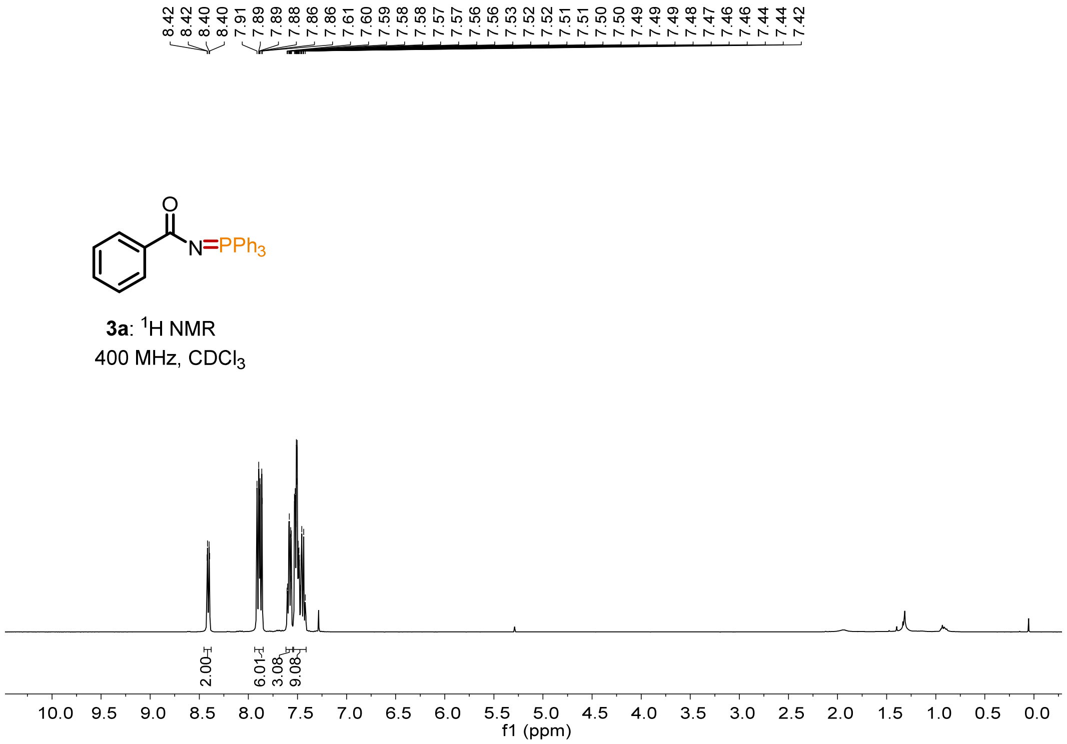

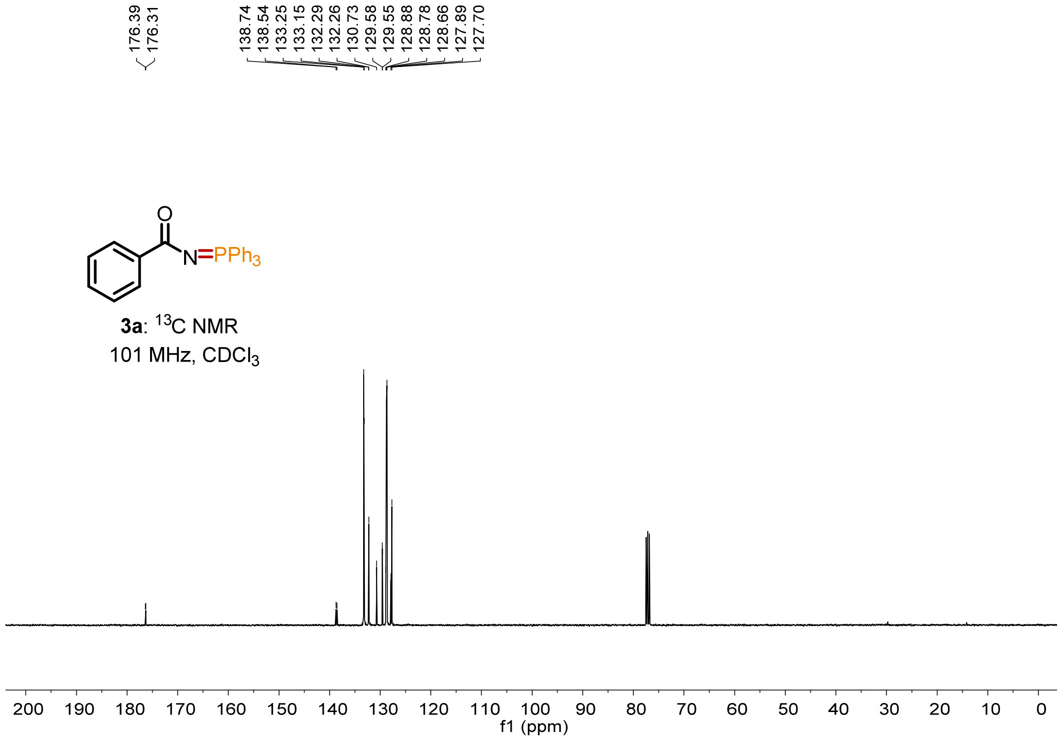

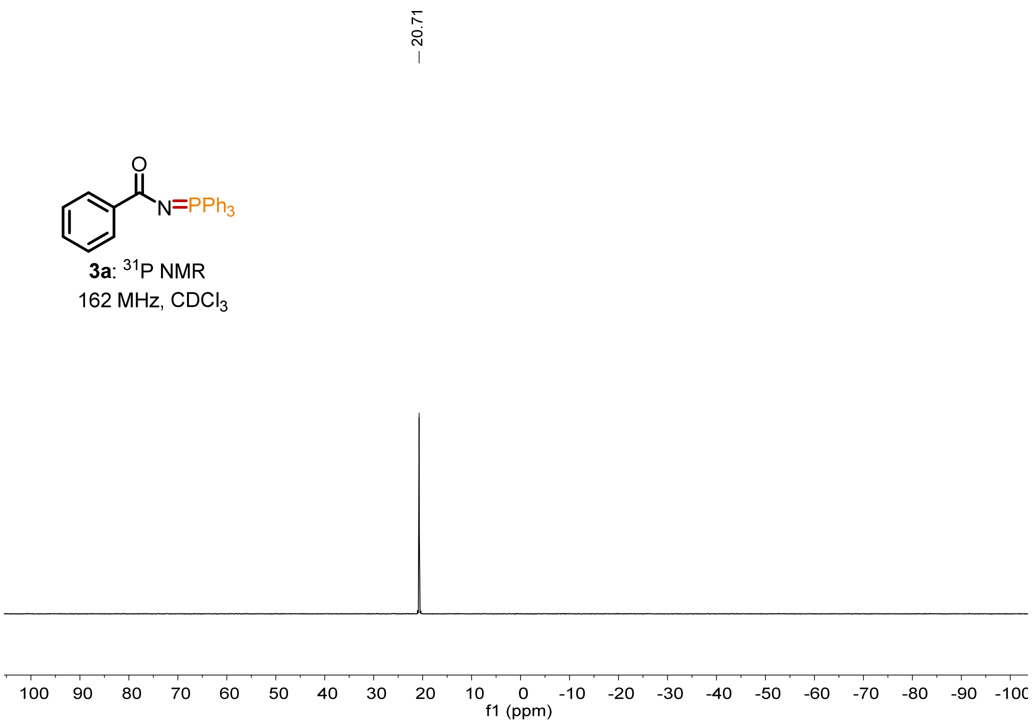

20.71
O
N=PPh3
3a: 31P NMR
162 MHz, CDCl3
100 90 80 70 60 50 40 30 20 10 0 -10 -20 -30 -40 -50 -60 -70 -80 -90 -100
f1 (ppm)

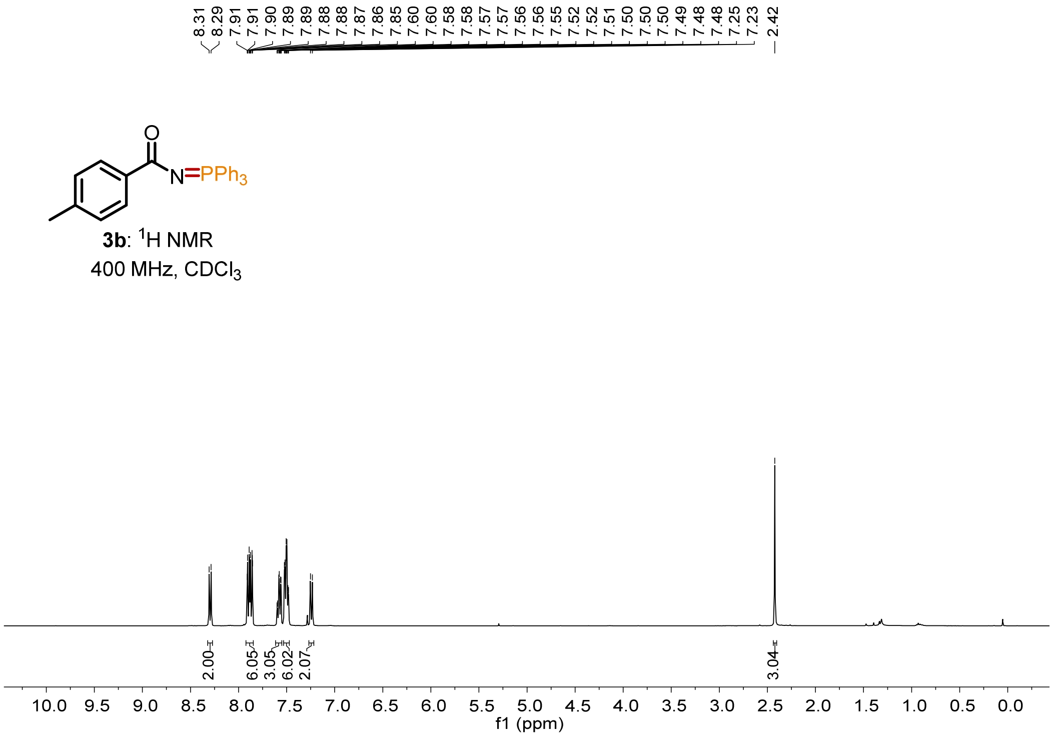

8.31 8.29 7.91 7.91 7.90 7.89 7.89 7.88 7.88 7.87 7.86 7.85 7.60 7.60 7.58 7.58 7.57 7.57 7.56 7.56 7.55 7.52 7.52 7.51 7.50 7.50 7.50 7.49 7.48 7.48 7.25 7.23 2.42
O
N=PPh3
3b: 1H NMR
400 MHz, CDCl3
2.00 6.05 3.05 6.02 2.07 3.04
10.0 9.5 9.0 8.5 8.0 7.5 7.0 6.5 6.0 5.5 5.0 4.5 4.0 3.5 3.0 2.5 2.0 1.5 1.0 0.5 0.0
f1 (ppm)

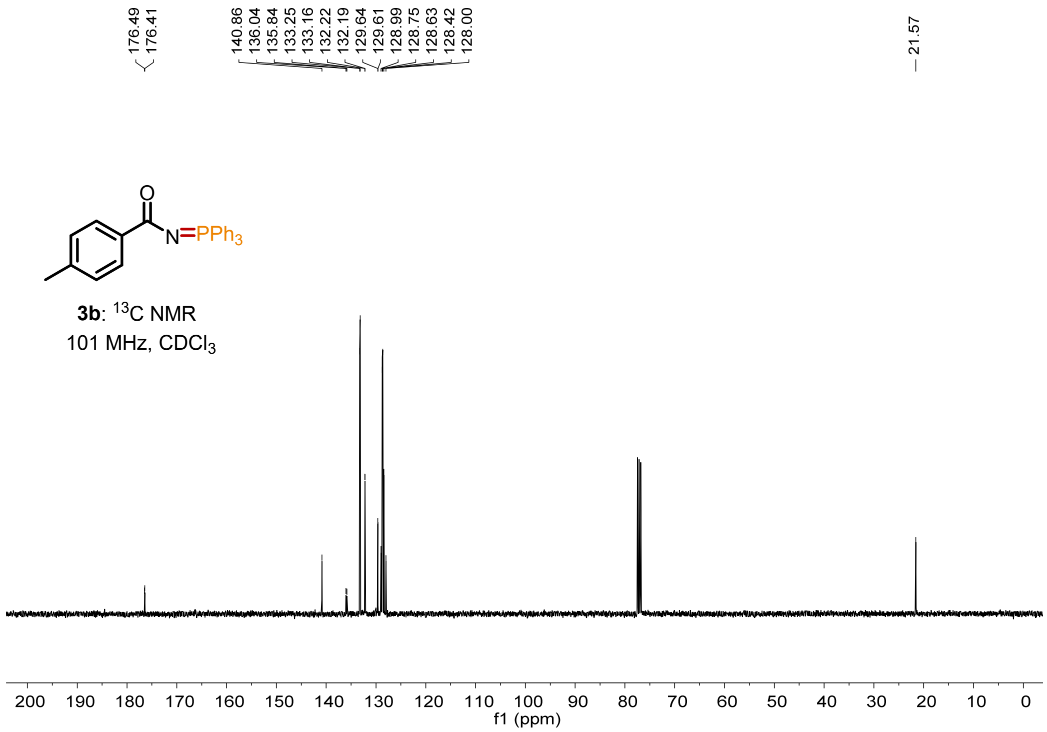
176.49
176.41
140.86
136.04
135.84
133.25
133.16
132.22
132.19
129.64
129.61
128.99
128.75
128.63
128.42
128.00
21.57
O
N=PPh3
3b: 13C NMR
101 MHz, CDCl3
200 190 180 170 160 150 140 130 120 110 100 90 80 70 60 50 40 30 20 10 0
f1 (ppm)

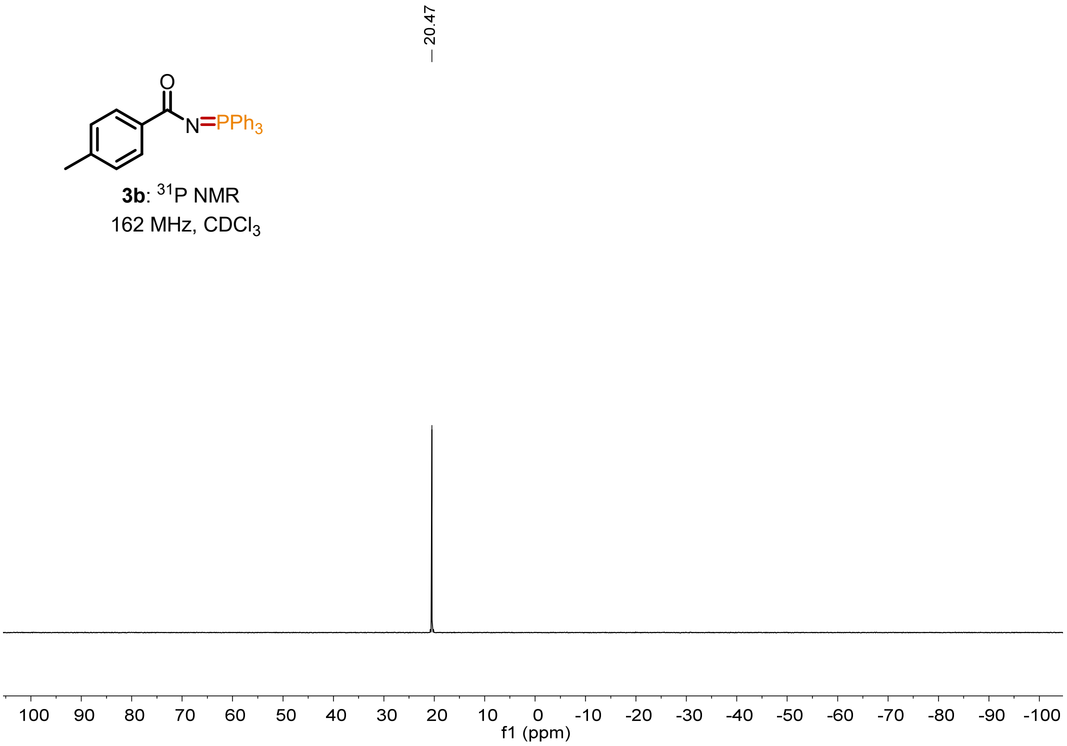
20.47
O
N=PPh3
3b: 31P NMR
162 MHz, CDCl3
100 90 80 70 60 50 40 30 20 10 0 -10 -20 -30 -40 -50 -60 -70 -80 -90 -100
f1 (ppm)

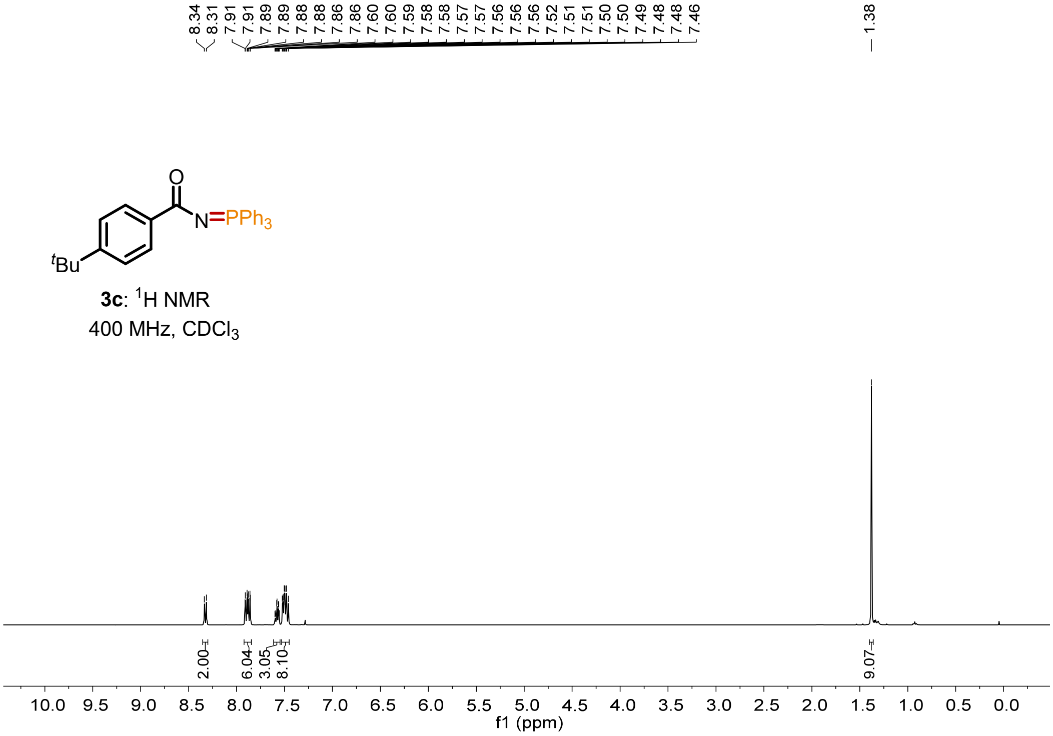
8.34
8.31
7.91
7.91
7.89
7.89
7.88
7.88
7.86
7.60
7.60
7.59
7.58
7.58
7.57
7.57
7.56
7.56
7.56
7.52
7.51
7.51
7.50
7.50
7.49
7.48
7.48
7.46
1.38
N=PPh3
tBu
3c: 1H NMR
400 MHz, CDCl3
2.00
6.04
3.05
8.10
9.07
10.0 9.5 9.0 8.5 8.0 7.5 7.0 6.5 6.0 5.5 5.0 4.5 4.0 3.5 3.0 2.5 2.0 1.5 1.0 0.5 0.0
f1 (ppm)

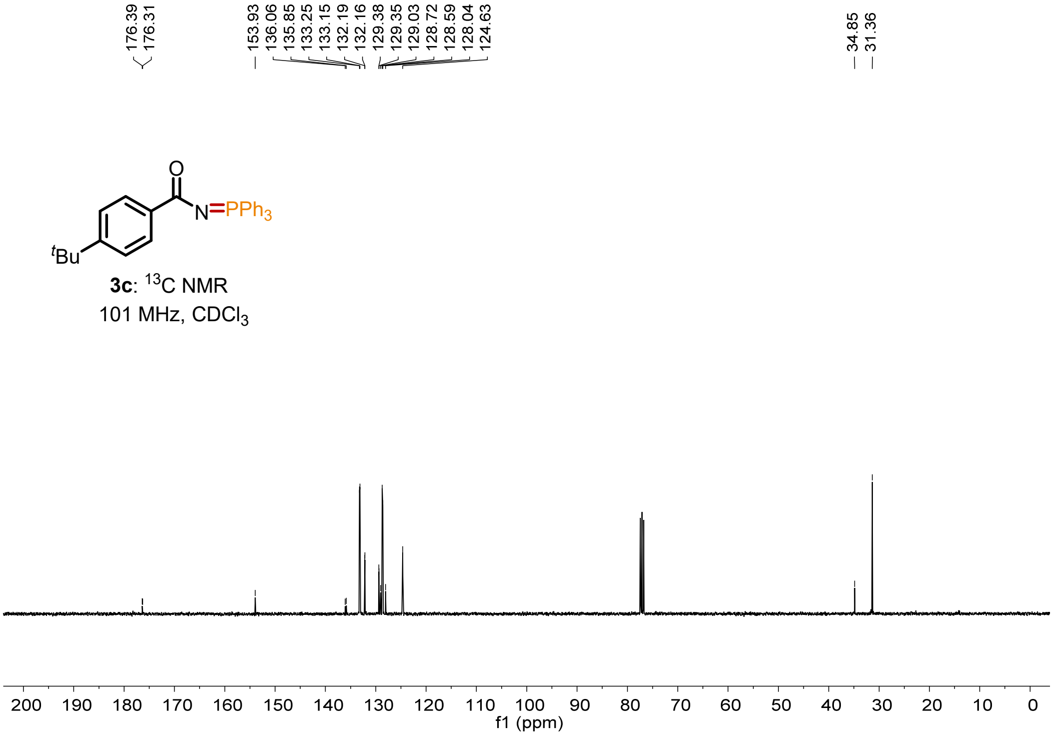
176.39
176.31
153.93
136.06
135.85
133.25
133.15
132.19
132.16
129.38
129.35
129.03
128.72
128.59
128.04
124.63
34.85
31.36
N=PPh3
tBu
3c: 13C NMR
101 MHz, CDCl3
200 190 180 170 160 150 140 130 120 110 100 90 80 70 60 50 40 30 20 10 0
f1 (ppm)

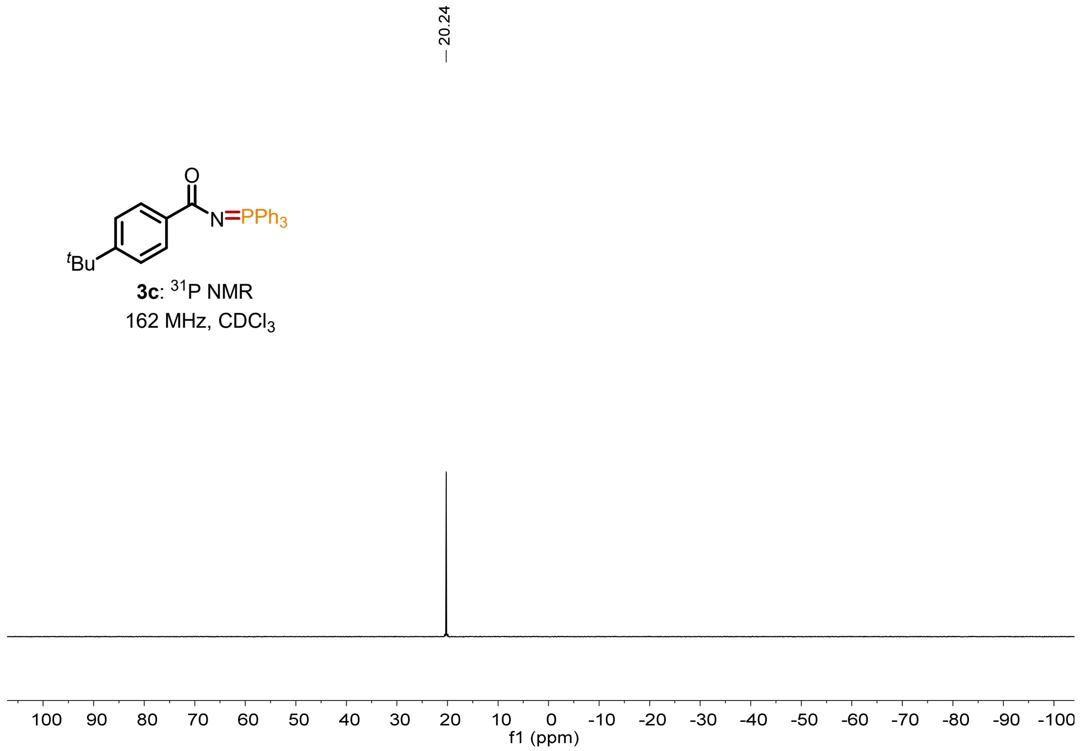
N=PPh3
tBu
3c: 31P NMR
162 MHz, CDCl3
f1 (ppm)

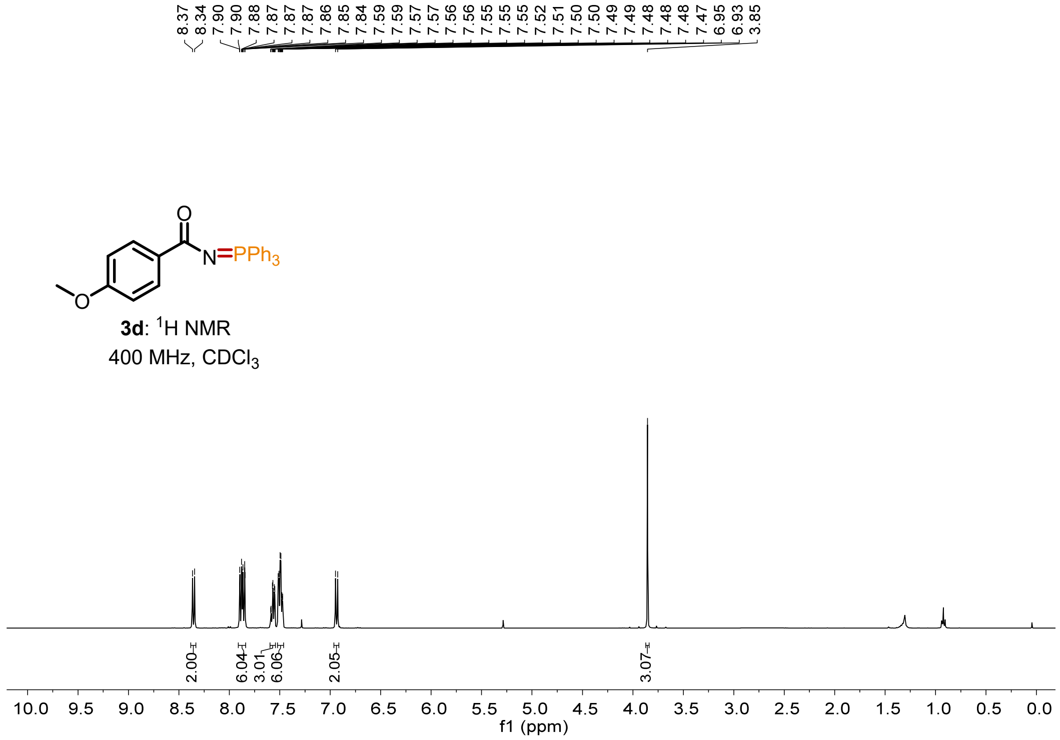
N=PPh3
3d: 1H NMR
400 MHz, CDCl3
f1 (ppm)

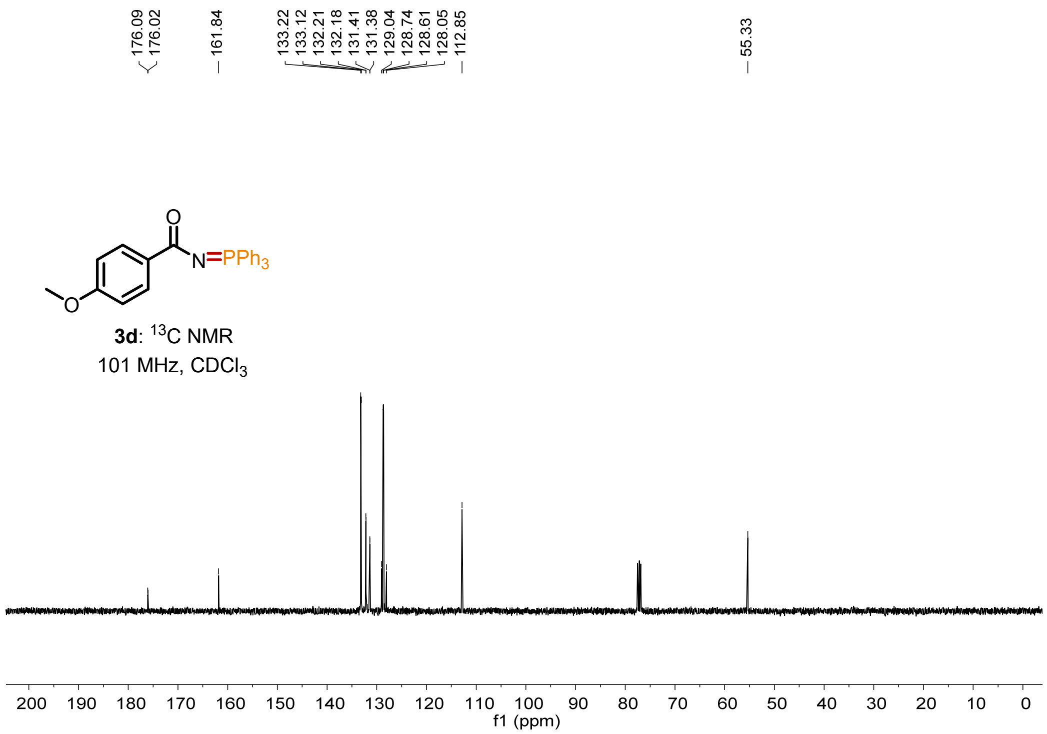

3d: ^{13}C NMR
101 MHz, $CDCl_3$

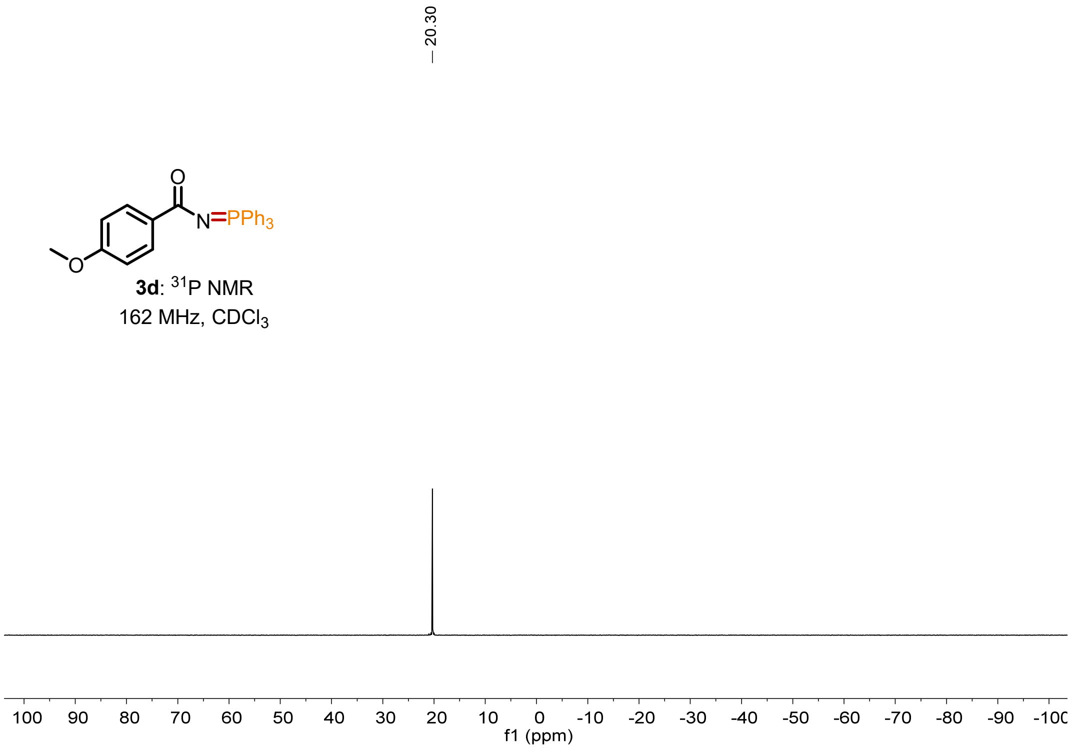

3d: ^{31}P NMR
162 MHz, $CDCl_3$

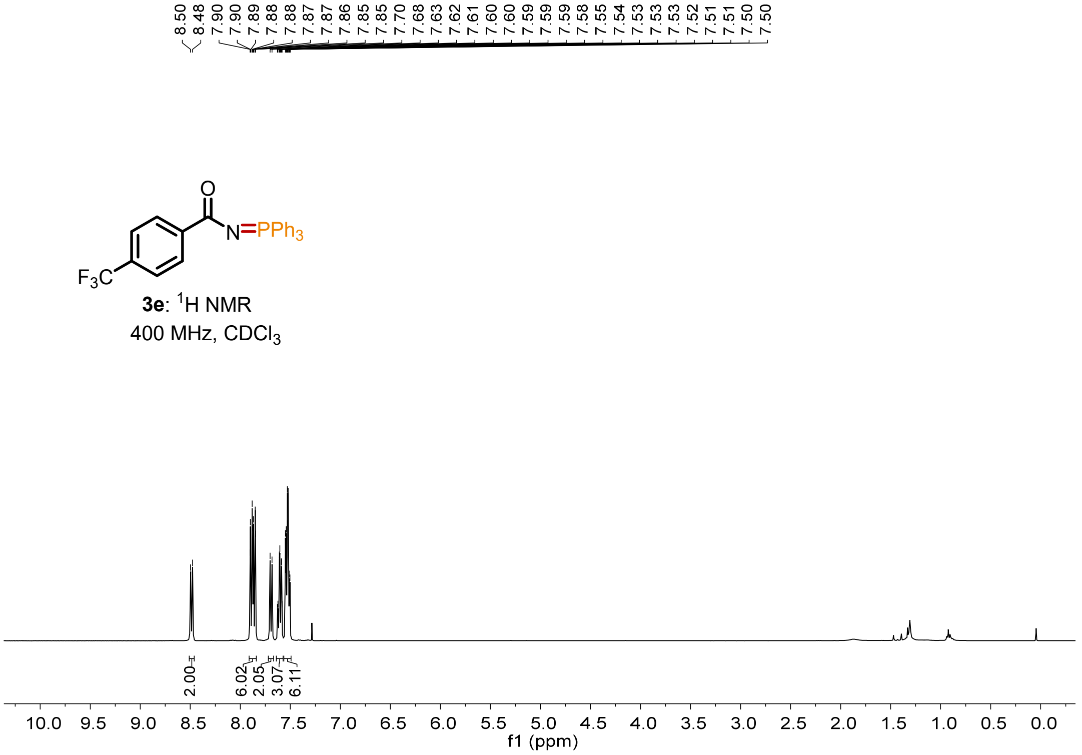

3e: ^{1}H NMR
400 MHz, $CDCl_3$

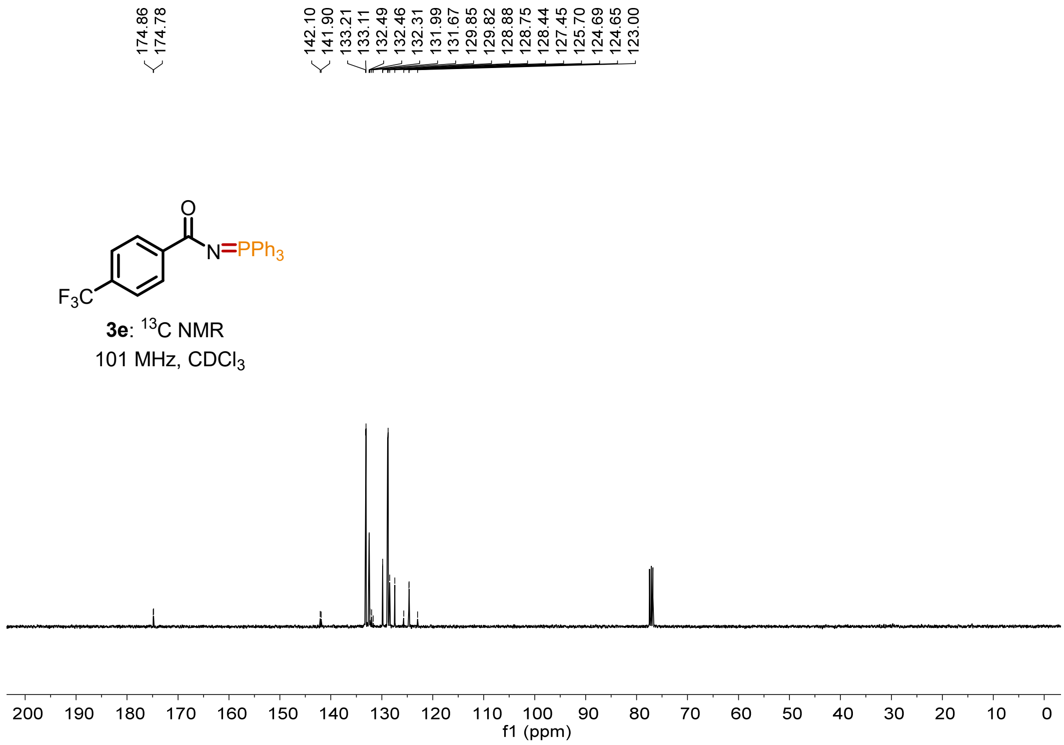

3e: ^{13}C NMR
101 MHz, $CDCl_3$

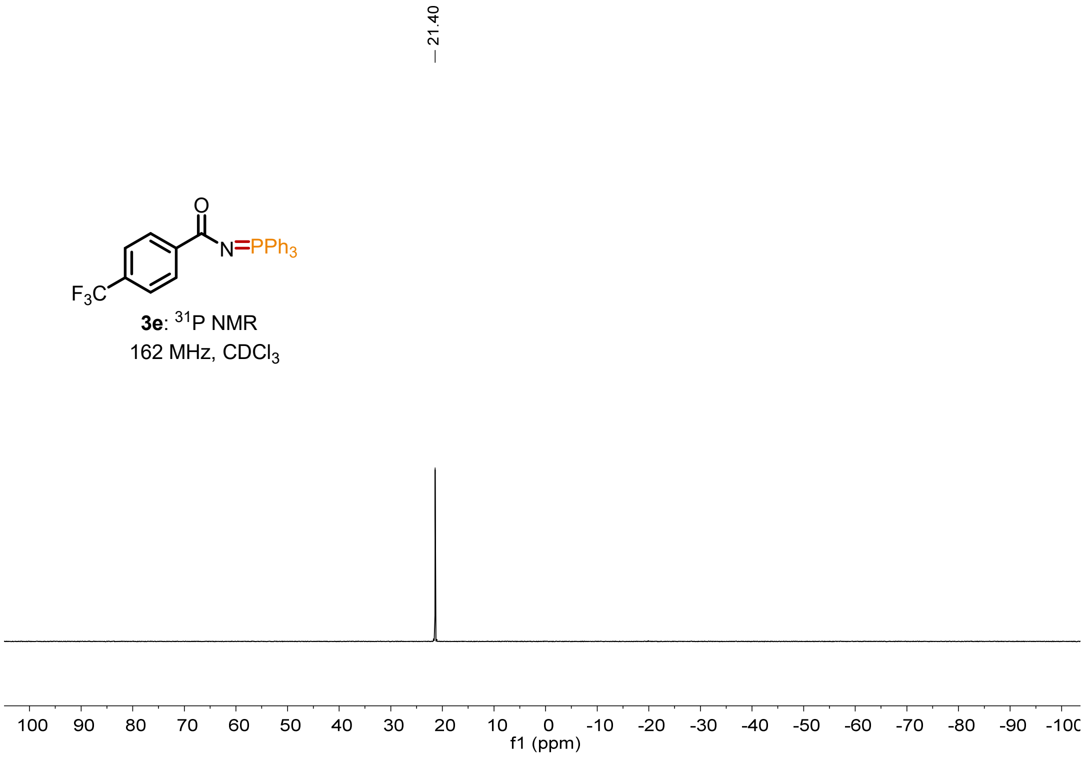
21.40
N=PPh3
F3C
O
3e: 31P NMR
162 MHz, CDCl3
100 90 80 70 60 50 40 30 20 10 0 -10 -20 -30 -40 -50 -60 -70 -80 -90 -100
f1 (ppm)

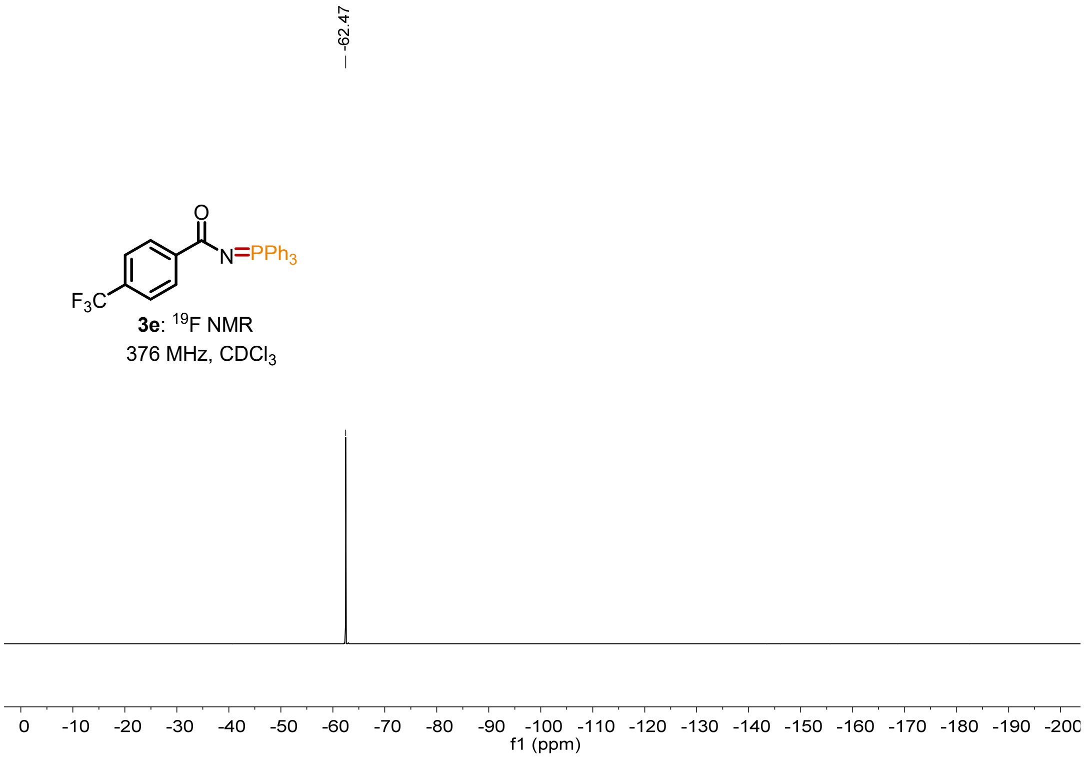
-62.47
N=PPh3
F3C
O
3e: 19F NMR
376 MHz, CDCl3
0 -10 -20 -30 -40 -50 -60 -70 -80 -90 -100 -110 -120 -130 -140 -150 -160 -170 -180 -190 -200
f1 (ppm)

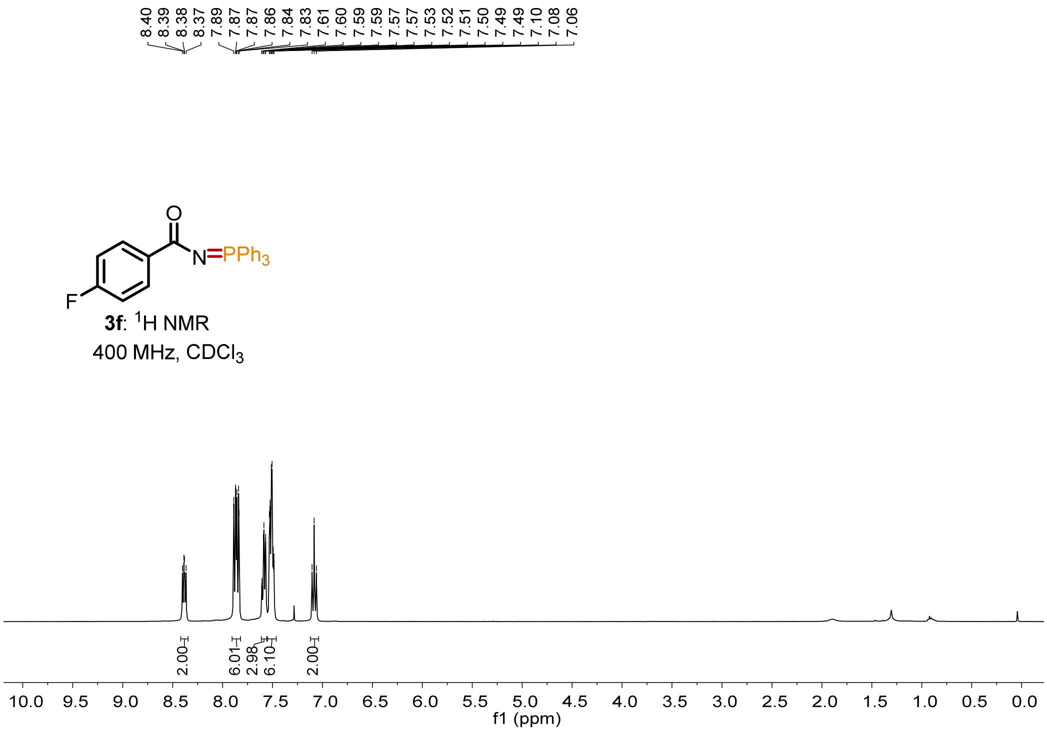
8.40
8.39
8.38
8.37
7.89
7.87
7.87
7.86
7.84
7.83
7.61
7.60
7.59
7.59
7.57
7.57
7.53
7.52
7.51
7.50
7.49
7.10
7.08
7.06
O
N=PPh3
F
3f: 1H NMR
400 MHz, CDCl3
2.00
6.01
2.98
6.10
2.00
10.0 9.5 9.0 8.5 8.0 7.5 7.0 6.5 6.0 5.5 5.0 4.5 4.0 3.5 3.0 2.5 2.0 1.5 1.0 0.5 0.0
f1 (ppm)

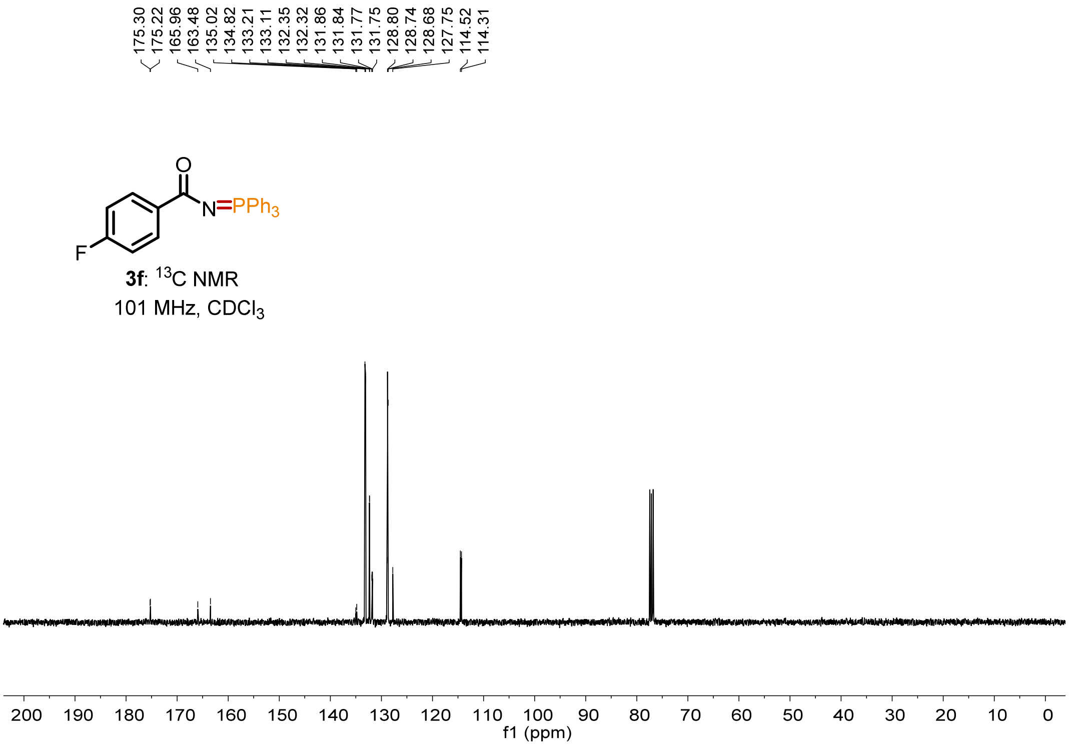
175.30
175.22
165.96
163.48
135.02
134.82
133.21
133.11
132.35
132.32
131.86
131.84
131.77
131.75
128.80
128.74
128.68
127.75
114.52
114.31
O
N=PPh3
F
3f: 13C NMR
101 MHz, CDCl3
200 190 180 170 160 150 140 130 120 110 100 90 80 70 60 50 40 30 20 10 0
f1 (ppm)

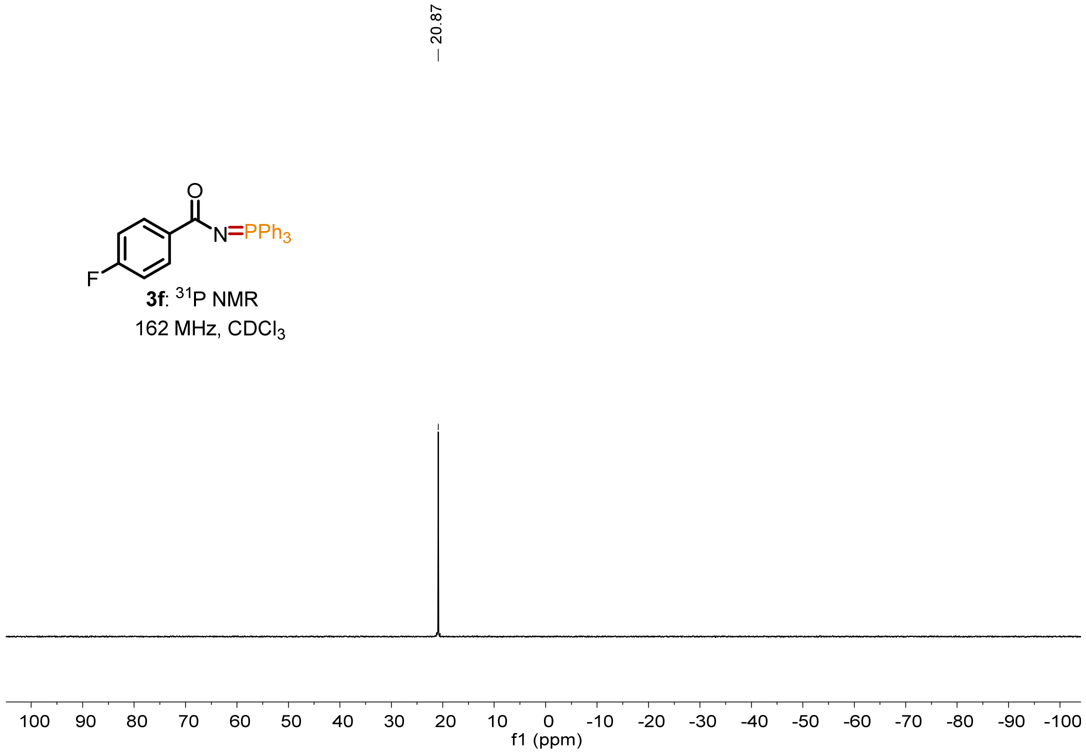
20.87
N=PPh3
O
F
3f: 31P NMR
162 MHz, CDCl3
100 90 80 70 60 50 40 30 20 10 0 -10 -20 -30 -40 -50 -60 -70 -80 -90 -100
f1 (ppm)

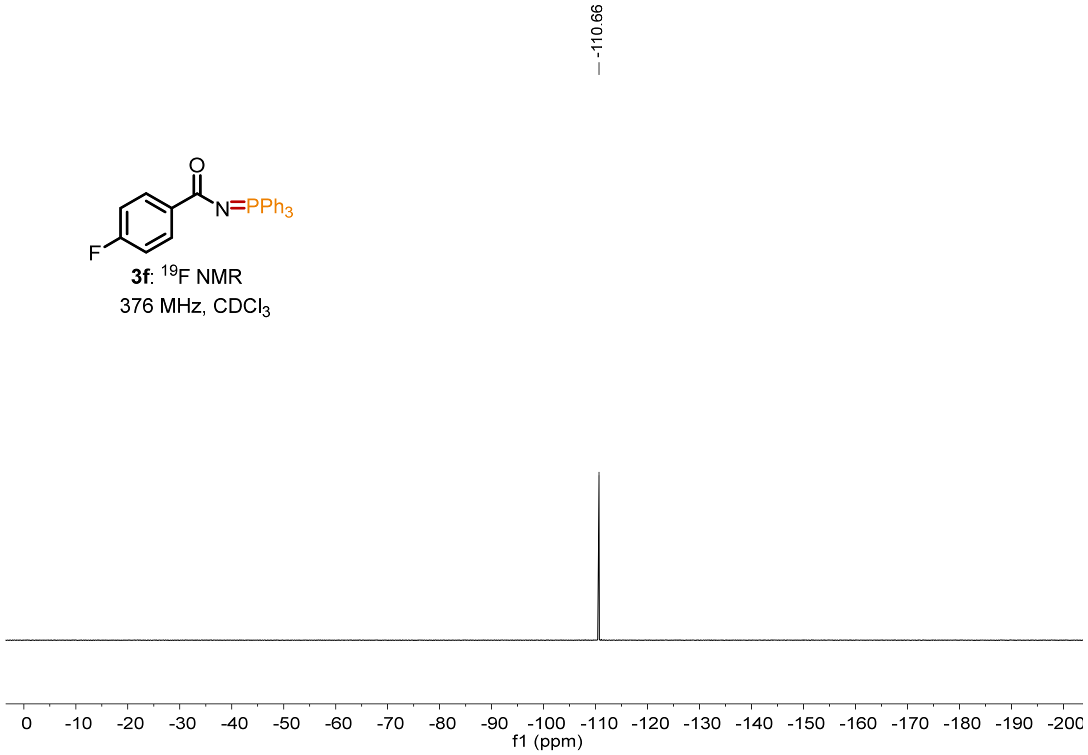
-110.66
N=PPh3
O
F
3f: 19F NMR
376 MHz, CDCl3
0 -10 -20 -30 -40 -50 -60 -70 -80 -90 -100 -110 -120 -130 -140 -150 -160 -170 -180 -190 -200
f1 (ppm)

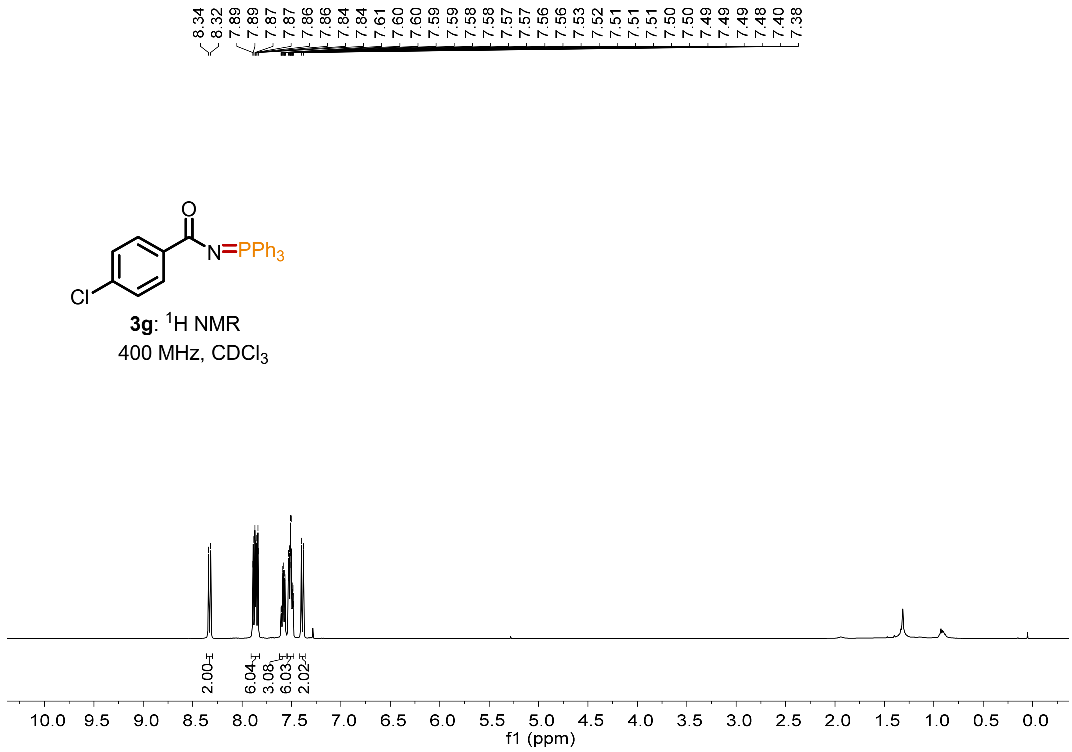

3g: ^{1}H NMR
400 MHz, $CDCl_3$

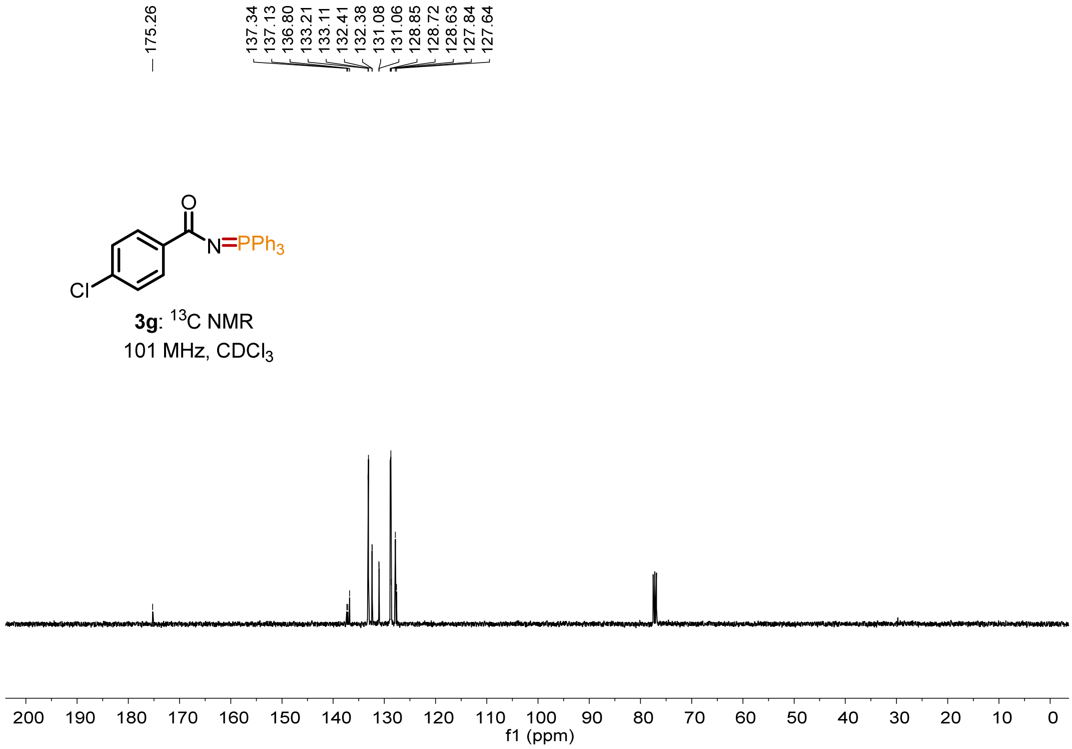

3g: ^{13}C NMR
101 MHz, $CDCl_3$

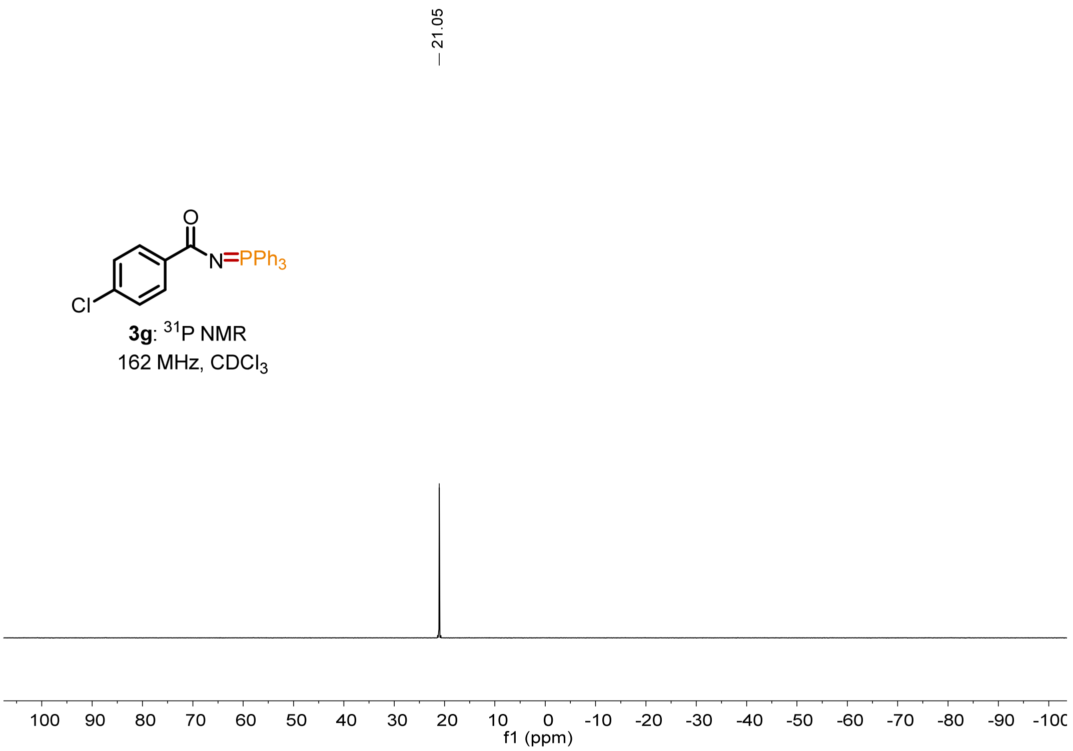
21.05
O
N=PPh3
Cl
3g: 31P NMR
162 MHz, CDCl3
100 90 80 70 60 50 40 30 20 10 0 -10 -20 -30 -40 -50 -60 -70 -80 -90 -100
f1 (ppm)

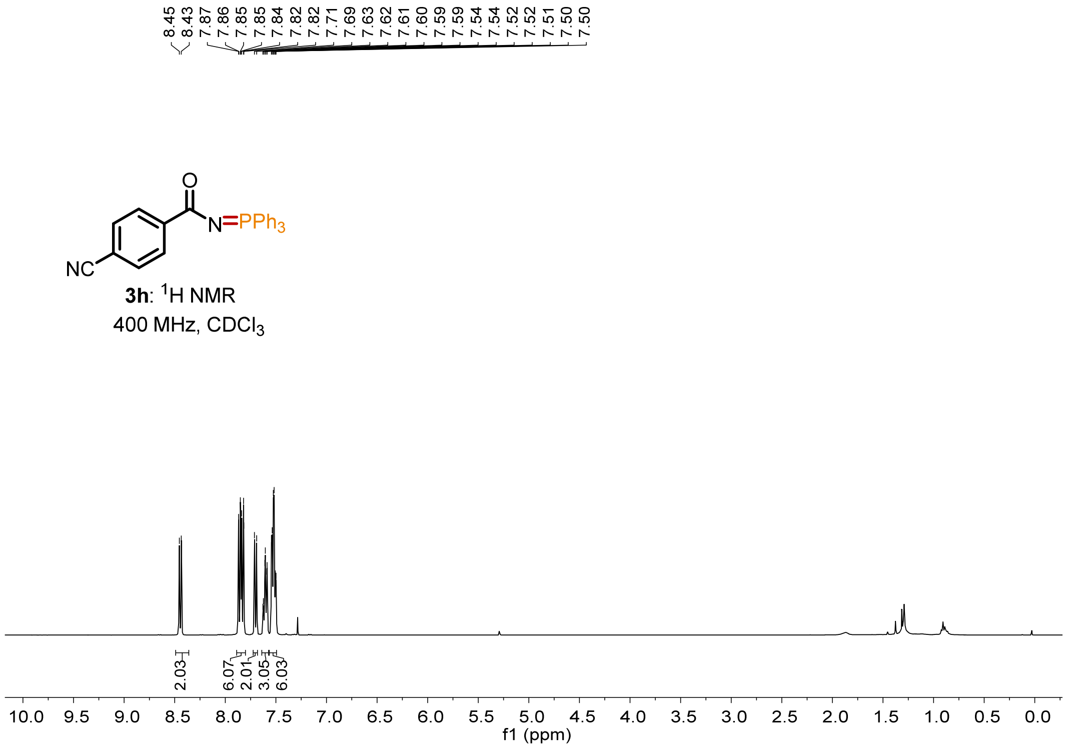
8.45 8.43 7.87 7.86 7.85 7.85 7.84 7.82 7.82 7.71 7.69 7.63 7.62 7.61 7.60 7.59 7.59 7.54 7.54 7.52 7.52 7.51 7.50 7.50
O
N=PPh3
NC
3h: 1H NMR
400 MHz, CDCl3
2.03
6.07 2.01 3.05 6.03
10.0 9.5 9.0 8.5 8.0 7.5 7.0 6.5 6.0 5.5 5.0 4.5 4.0 3.5 3.0 2.5 2.0 1.5 1.0 0.5 0.0
f1 (ppm)

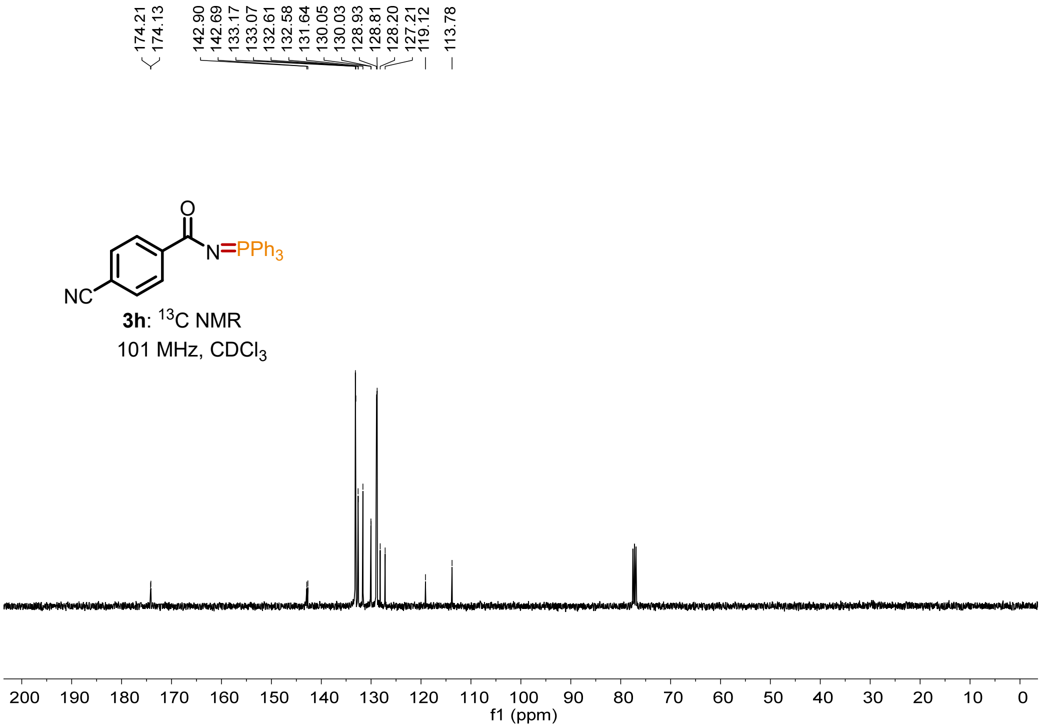
174.21
174.13
142.90
142.69
133.17
133.07
132.61
132.58
131.64
130.05
130.03
128.93
128.81
128.20
127.21
119.12
113.78
O
N=PPh3
NC
3h: 13C NMR
101 MHz, CDCl3
200 190 180 170 160 150 140 130 120 110 100 90 80 70 60 50 40 30 20 10 0
f1 (ppm)

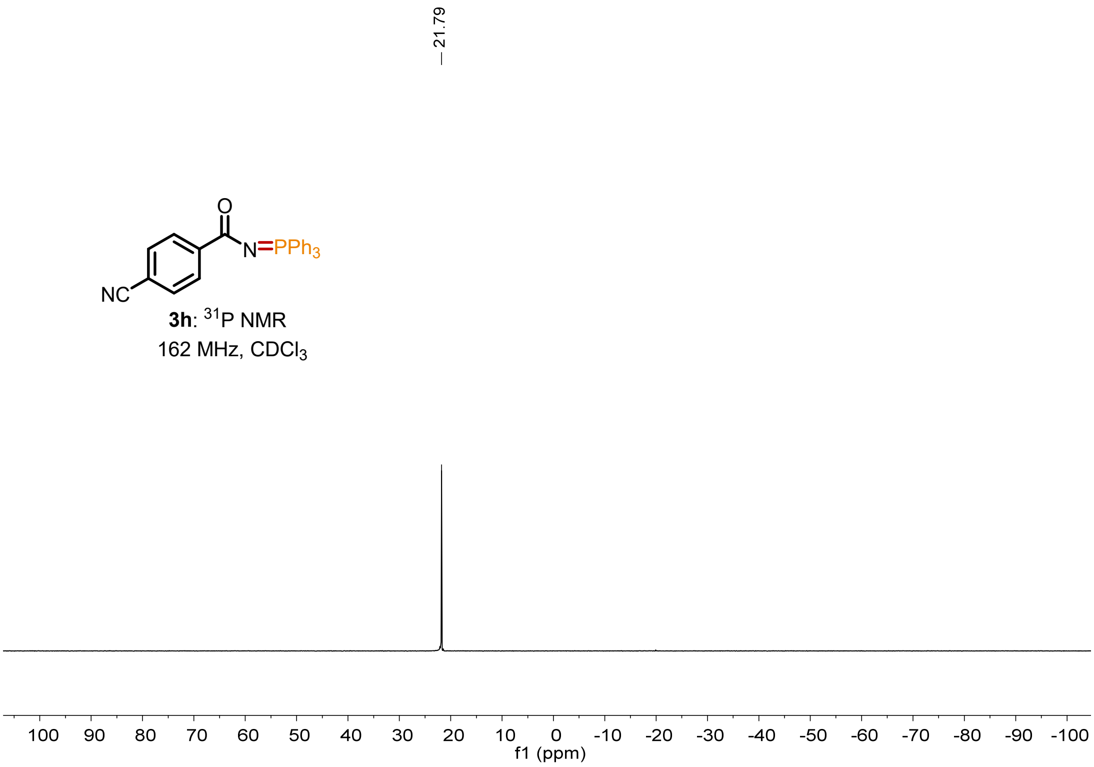
21.79
O
N=PPh3
NC
3h: 31P NMR
162 MHz, CDCl3
100 90 80 70 60 50 40 30 20 10 0 -10 -20 -30 -40 -50 -60 -70 -80 -90 -100
f1 (ppm)

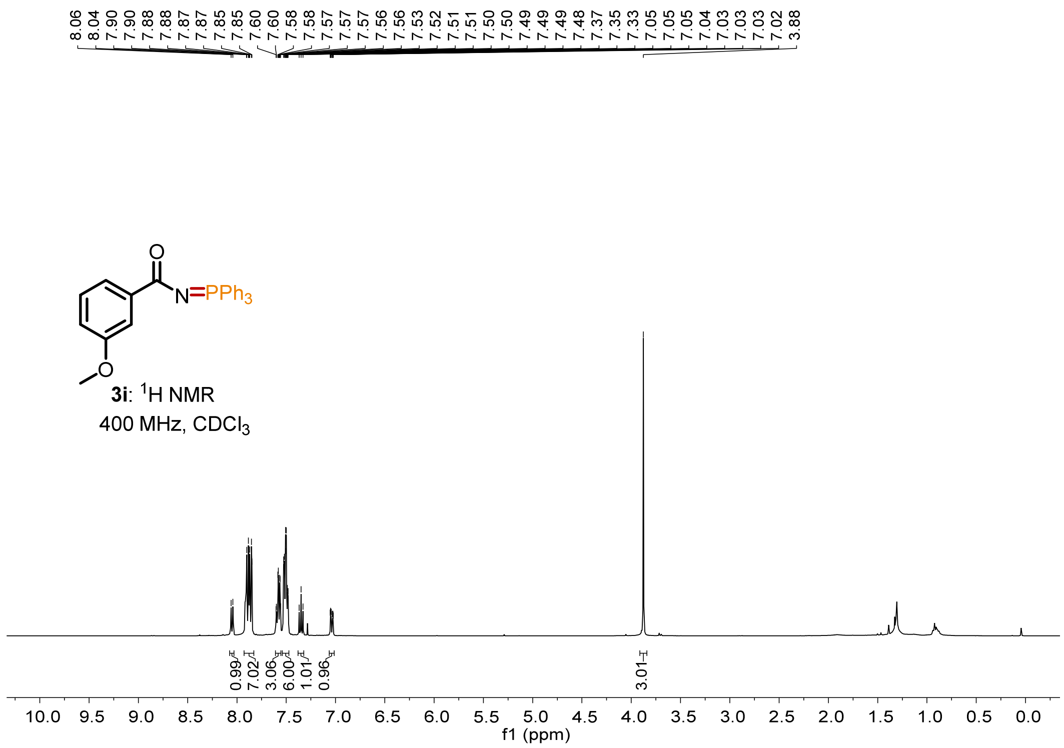

N=PPh3
3i: 1H NMR
400 MHz, CDCl3
8.06 8.04 7.90 7.90 7.88 7.88 7.87 7.87 7.85 7.85 7.60 7.60 7.58 7.58 7.57 7.57 7.56 7.56 7.53 7.52 7.51 7.51 7.50 7.50 7.49 7.49 7.48 7.37 7.35 7.33 7.05 7.05 7.05 7.04 7.03 7.03 7.03 7.02 3.88
0.99 7.02 3.06 6.00 1.01 0.96 3.01
10.0 9.5 9.0 8.5 8.0 7.5 7.0 6.5 6.0 5.5 5.0 4.5 4.0 3.5 3.0 2.5 2.0 1.5 1.0 0.5 0.0
f1 (ppm)

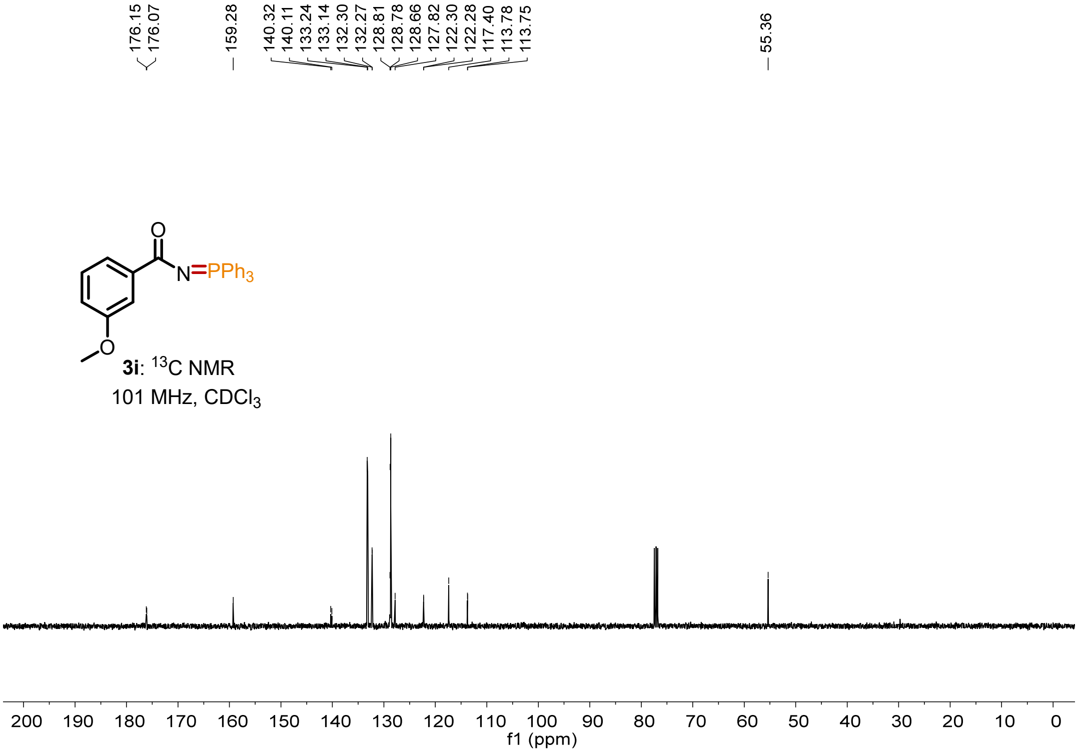

176.15 176.07 159.28 140.32 140.11 133.24 133.14 132.30 132.27 128.81 128.78 128.66 127.82 122.30 122.28 117.40 113.78 113.75 55.36
N=PPh3
3i: 13C NMR
101 MHz, CDCl3
200 190 180 170 160 150 140 130 120 110 100 90 80 70 60 50 40 30 20 10 0
f1 (ppm)

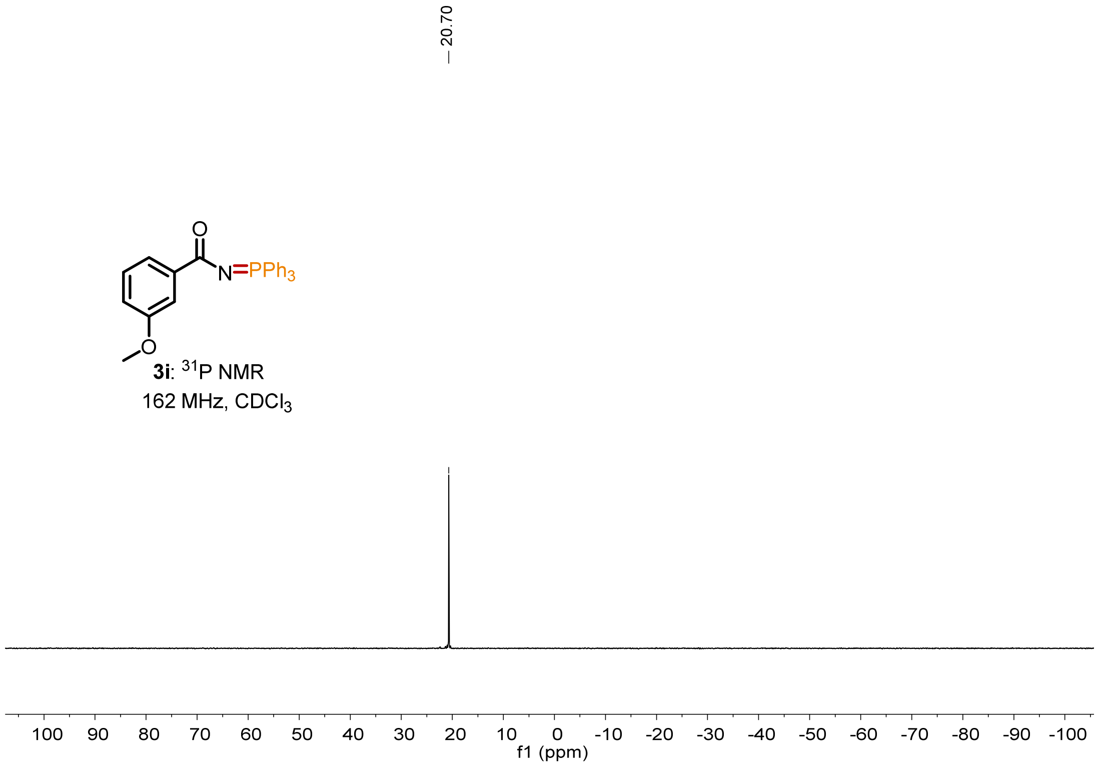
20.70
O
N=PPh3
O
3i: 31P NMR
162 MHz, CDCl3
f1 (ppm)

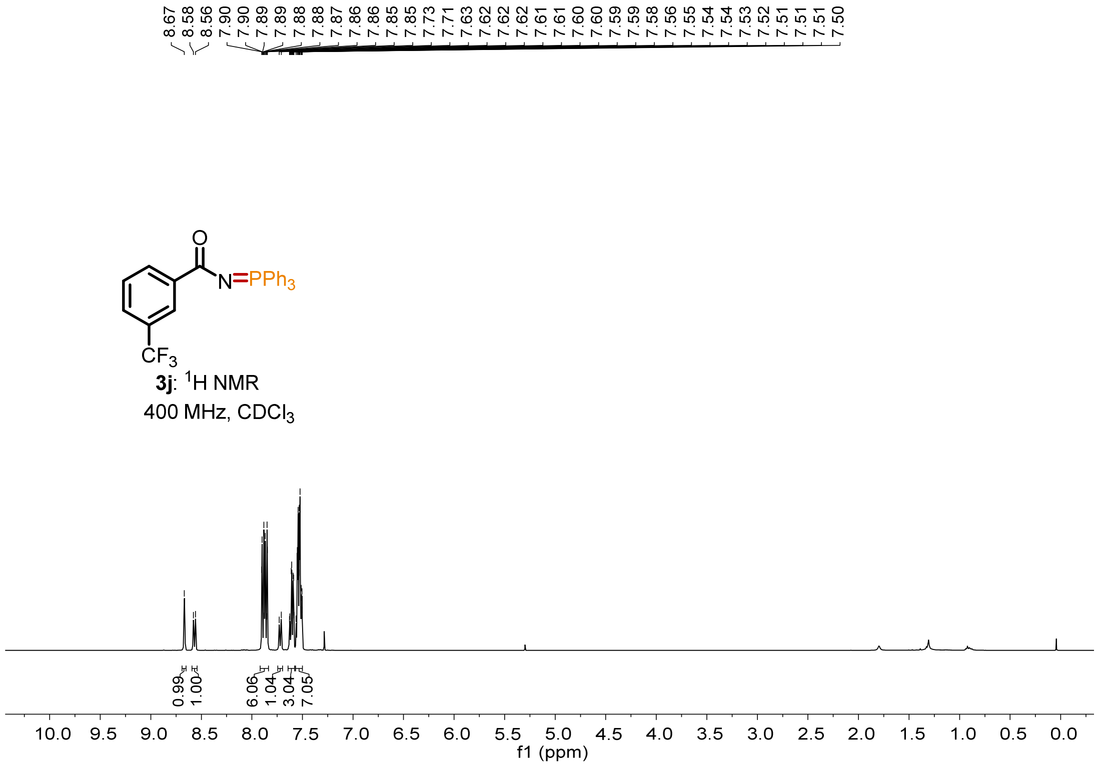
8.67
8.58
8.56
7.90
7.90
7.89
7.89
7.88
7.88
7.87
7.86
7.86
7.85
7.85
7.73
7.71
7.63
7.62
7.62
7.62
7.61
7.61
7.60
7.60
7.59
7.59
7.58
7.56
7.55
7.54
7.54
7.53
7.52
7.51
7.51
7.51
7.50
O
N=PPh3
CF3
3j: 1H NMR
400 MHz, CDCl3
0.99
1.00
6.06
1.04
3.04
7.05
f1 (ppm)

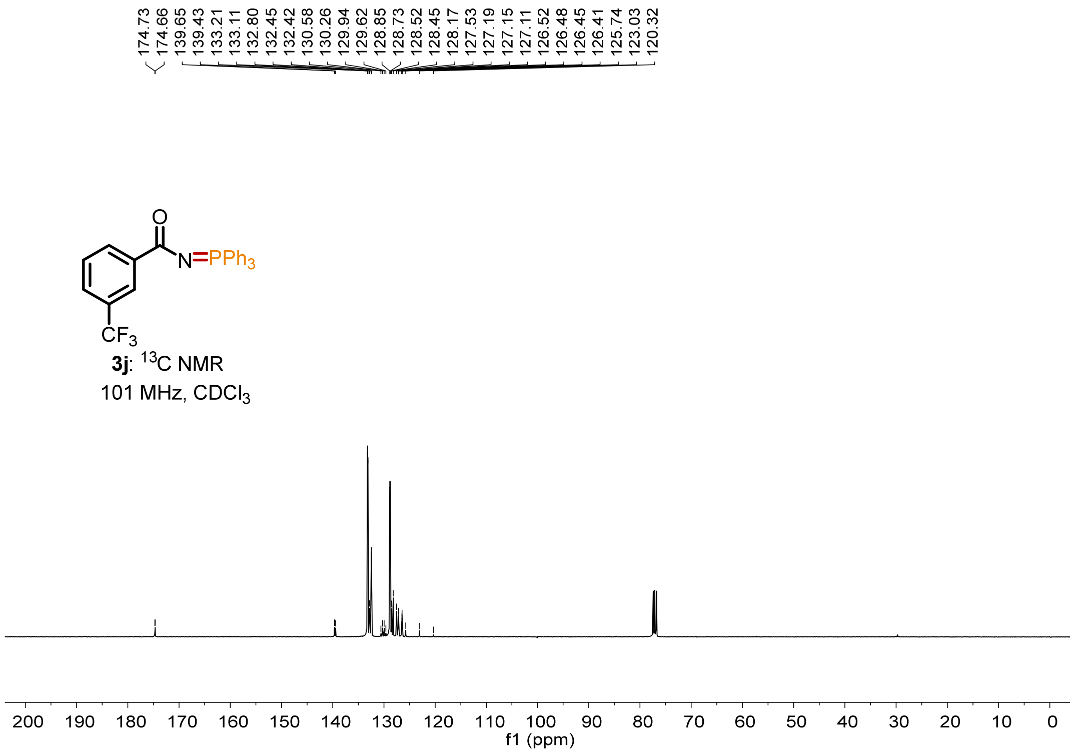
174.73
174.66
139.65
139.43
133.21
133.11
132.80
132.45
132.42
130.58
130.26
129.94
129.62
128.85
128.73
128.52
128.45
128.17
127.53
127.19
127.15
127.11
126.52
126.48
126.45
126.41
125.74
123.03
120.32
N=PPh3
CF3
3j: 13C NMR
101 MHz, CDCl3
f1 (ppm)

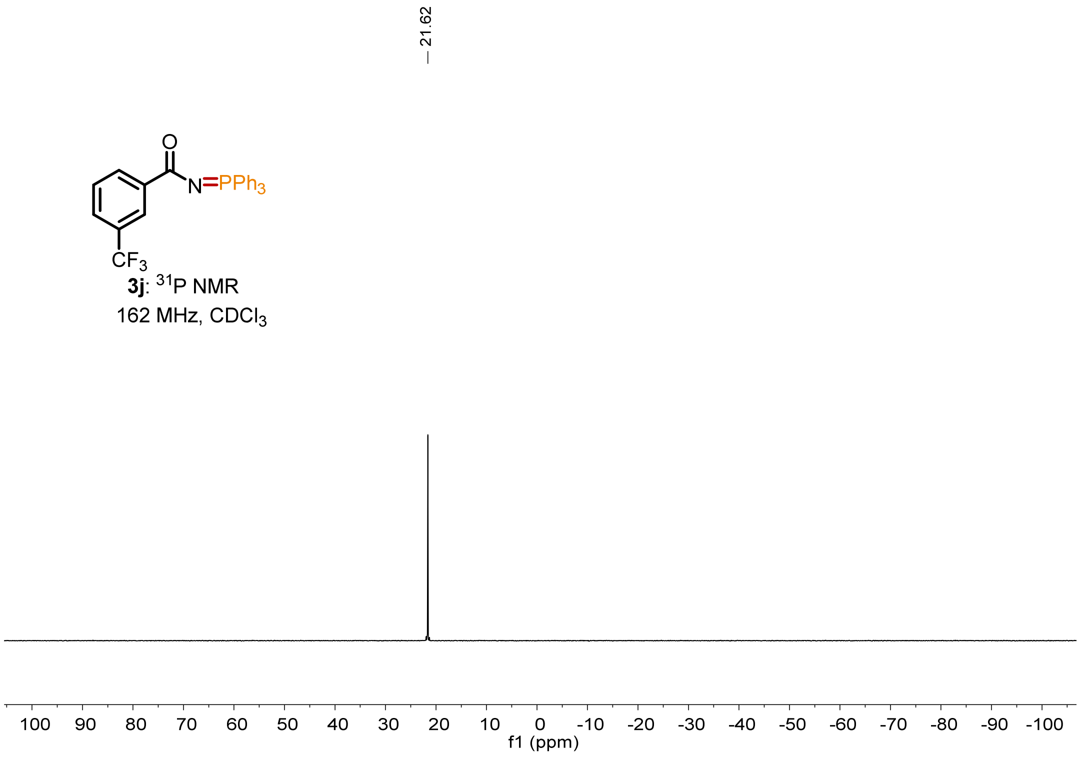
21.62
N=PPh3
CF3
3j: 31P NMR
162 MHz, CDCl3
f1 (ppm)

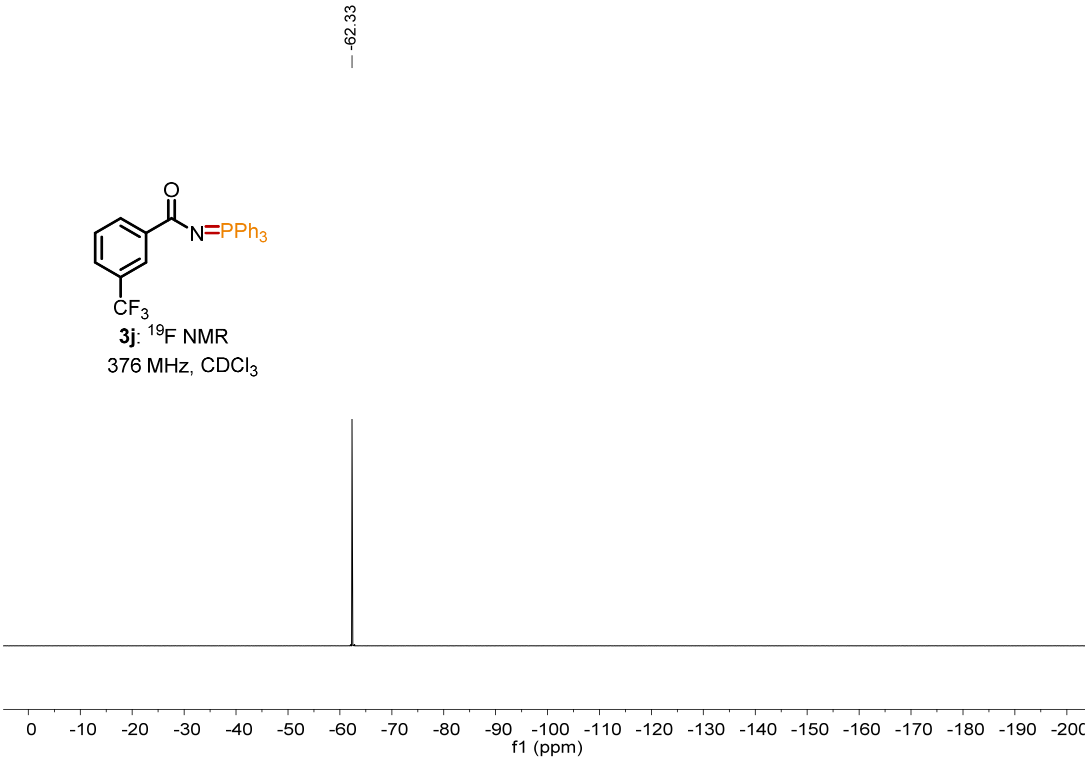

3j: ^{19}F NMR
376 MHz, $CDCl_3$

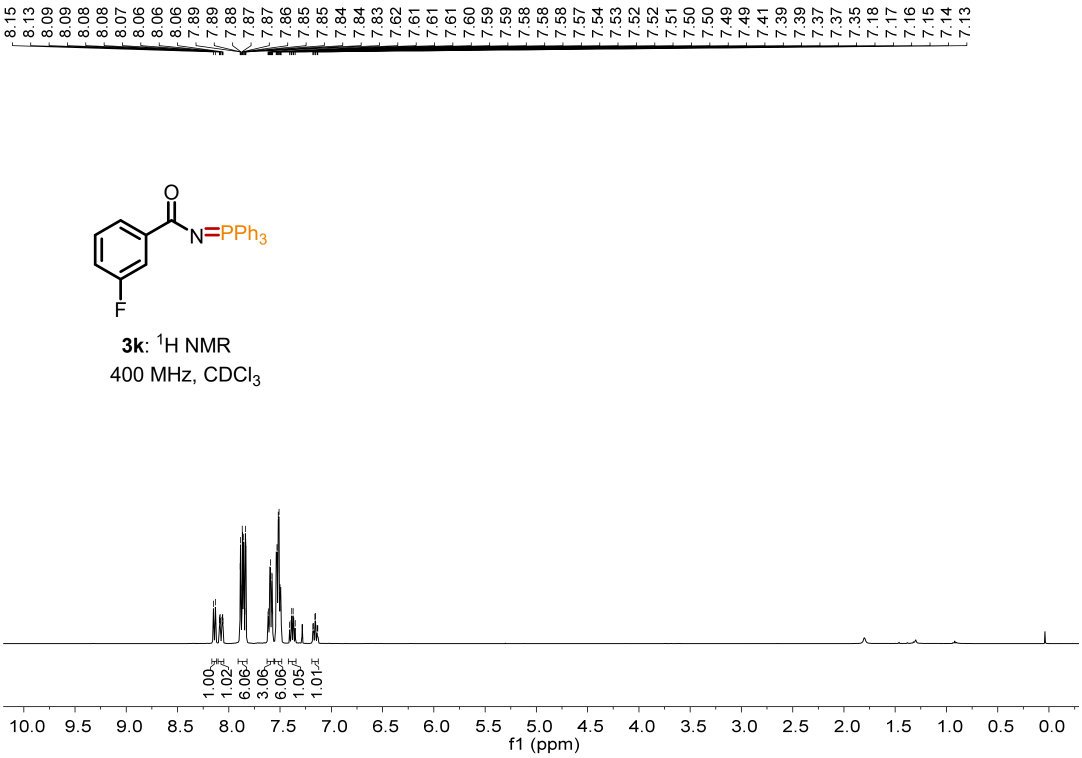

3k: ^{1}H NMR
400 MHz, $CDCl_3$

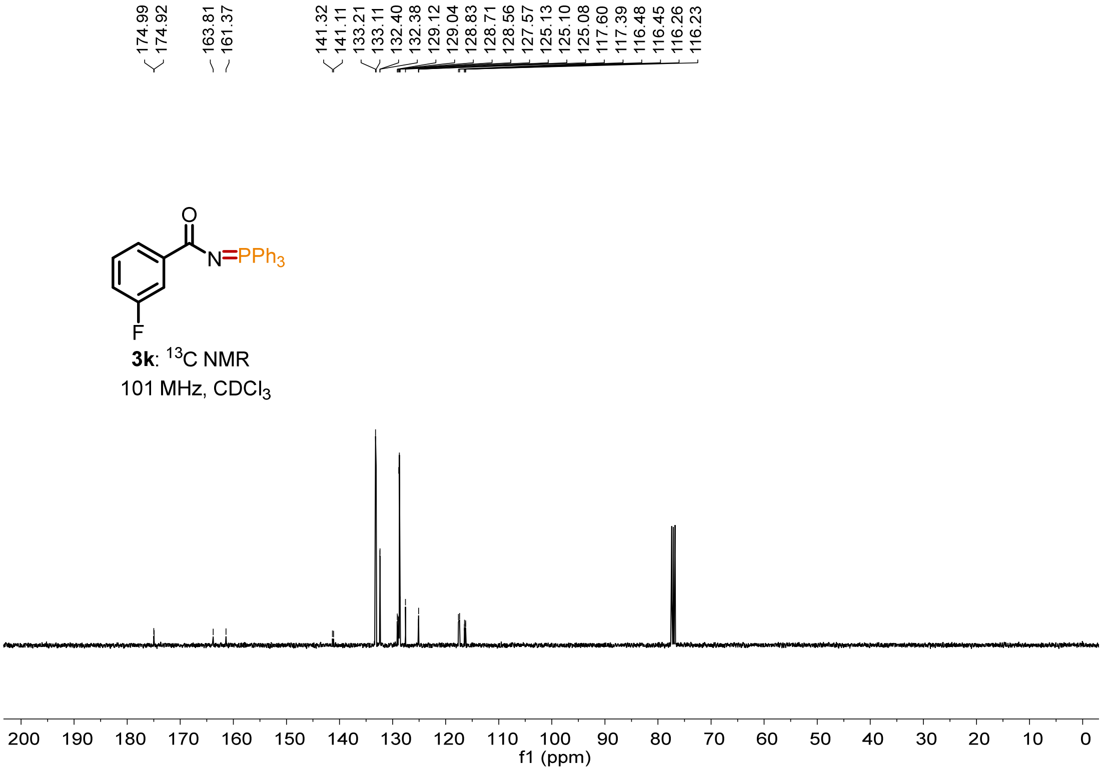
174.99
174.92
163.81
161.37
141.32
141.11
133.21
133.11
132.40
132.38
129.12
129.04
128.83
128.71
128.56
127.57
125.13
125.10
125.08
117.60
117.39
116.48
116.45
116.26
116.23
O
N=PPh3
F
3k: 13C NMR
101 MHz, CDCl3
200 190 180 170 160 150 140 130 120 110 100 90 80 70 60 50 40 30 20 10 0
f1 (ppm)

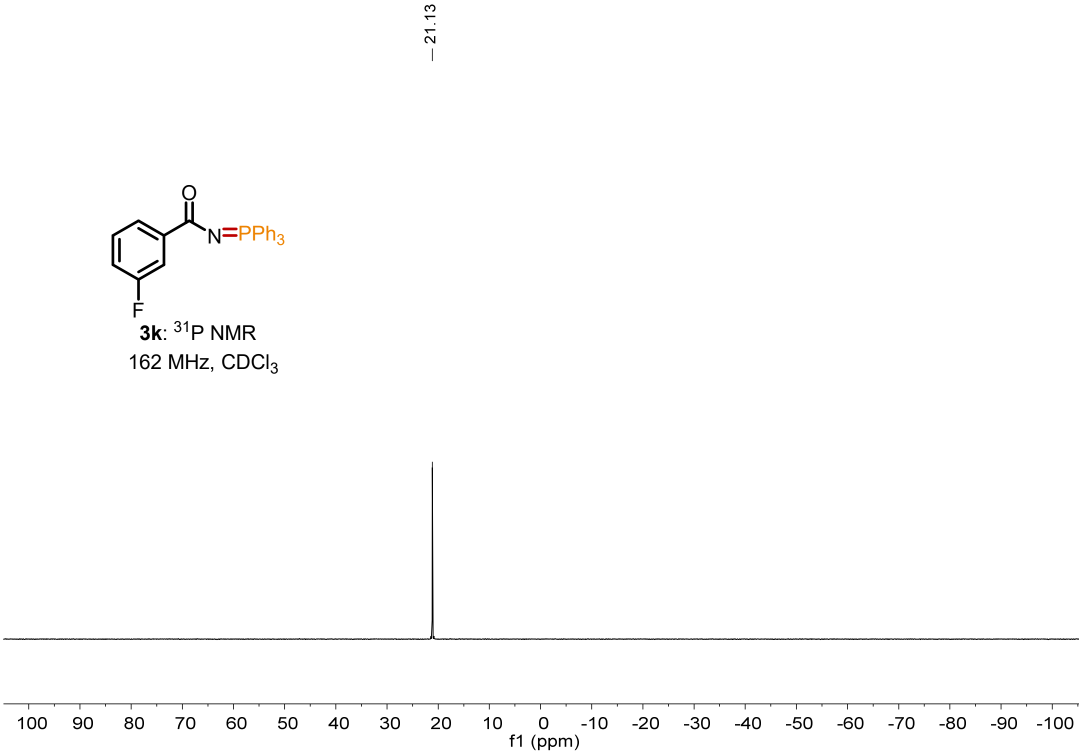
21.13
O
N=PPh3
F
3k: 31P NMR
162 MHz, CDCl3
100 90 80 70 60 50 40 30 20 10 0 -10 -20 -30 -40 -50 -60 -70 -80 -90 -100
f1 (ppm)

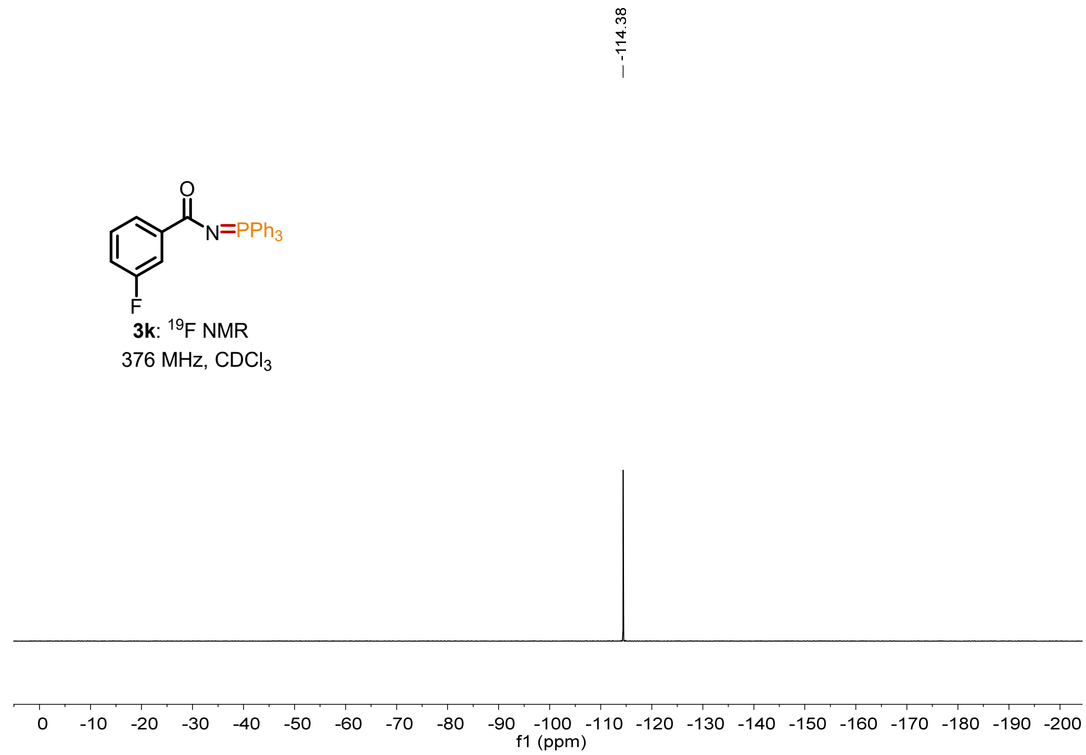
-114.38
N=PPh3
O
F
3k: 19F NMR
376 MHz, CDCl3
0 -10 -20 -30 -40 -50 -60 -70 -80 -90 -100 -110 -120 -130 -140 -150 -160 -170 -180 -190 -200
f1 (ppm)

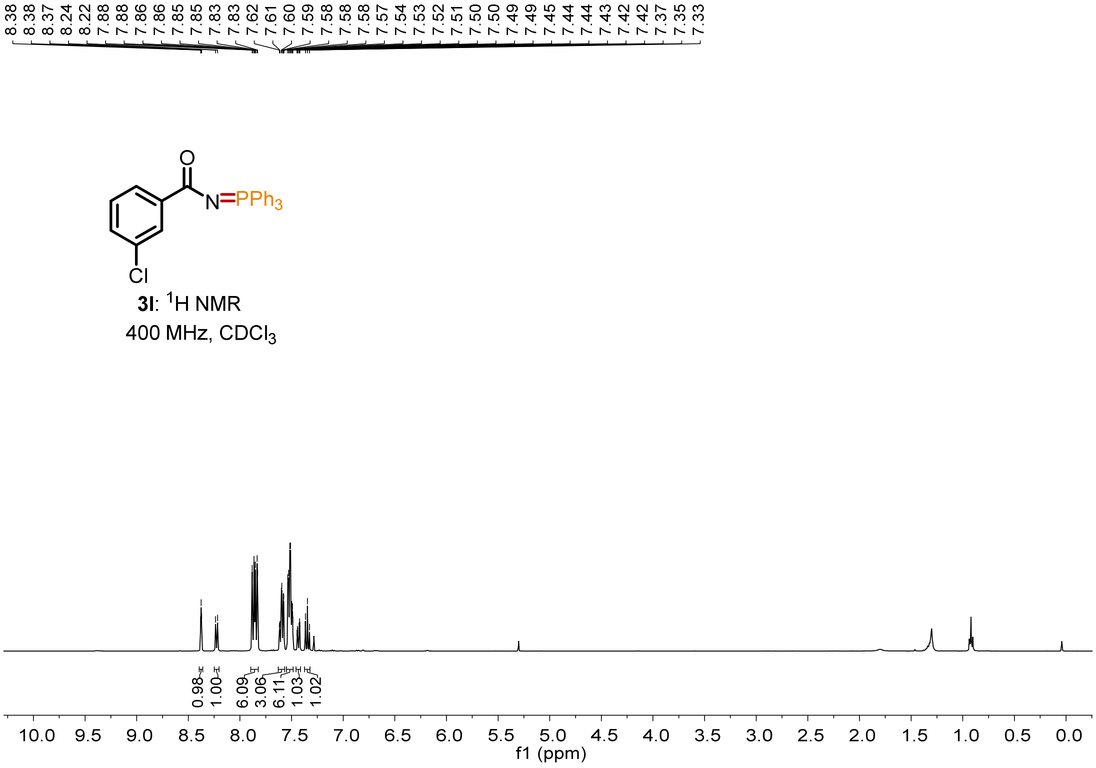
8.38 8.38 8.37 8.24 8.22 7.88 7.88 7.86 7.86 7.85 7.85 7.83 7.83 7.62 7.61 7.60 7.59 7.58 7.58 7.58 7.57 7.54 7.53 7.52 7.51 7.50 7.50 7.49 7.49 7.45 7.44 7.44 7.43 7.42 7.42 7.37 7.35 7.33
N=PPh3
O
Cl
3l: 1H NMR
400 MHz, CDCl3
0.98 1.00 6.09 3.06 6.11 1.03 1.02
10.0 9.5 9.0 8.5 8.0 7.5 7.0 6.5 6.0 5.5 5.0 4.5 4.0 3.5 3.0 2.5 2.0 1.5 1.0 0.5 0.0
f1 (ppm)

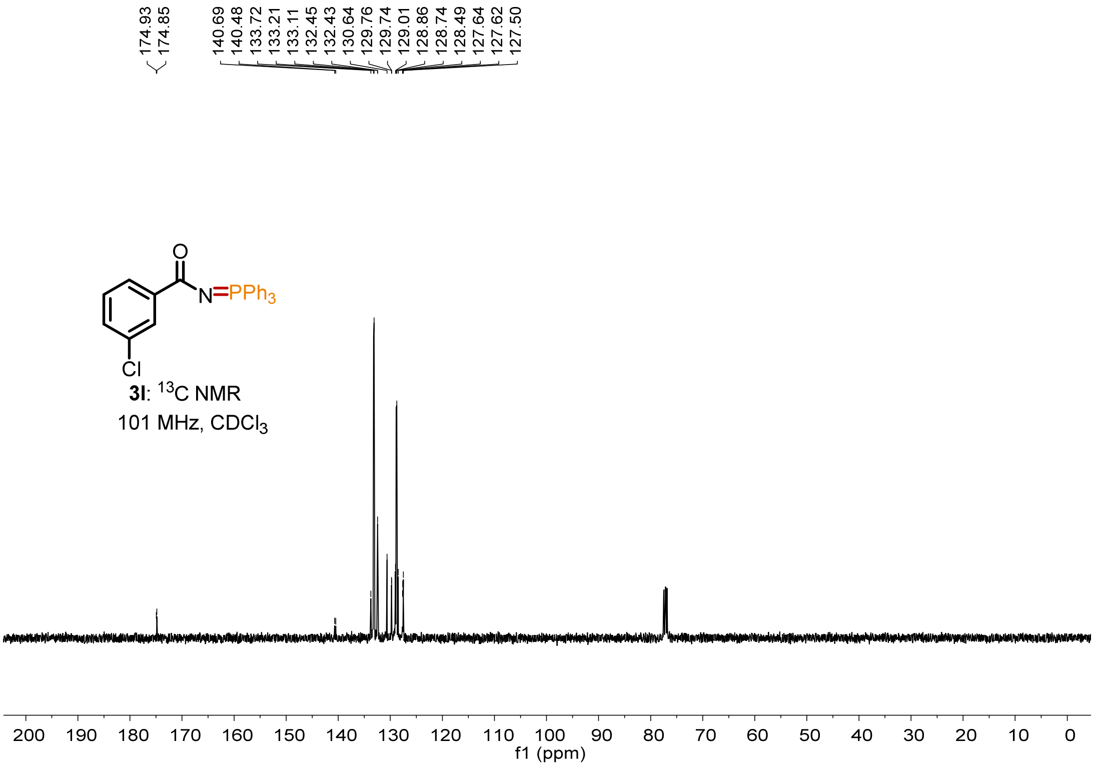
174.93
174.85
140.69
140.48
133.72
133.21
133.11
132.45
132.43
130.64
129.76
129.74
129.01
128.86
128.74
128.49
127.64
127.62
127.50
O
N=PPh3
Cl
3l: 13C NMR
101 MHz, CDCl3
200 190 180 170 160 150 140 130 120 110 100 90 80 70 60 50 40 30 20 10 0
f1 (ppm)

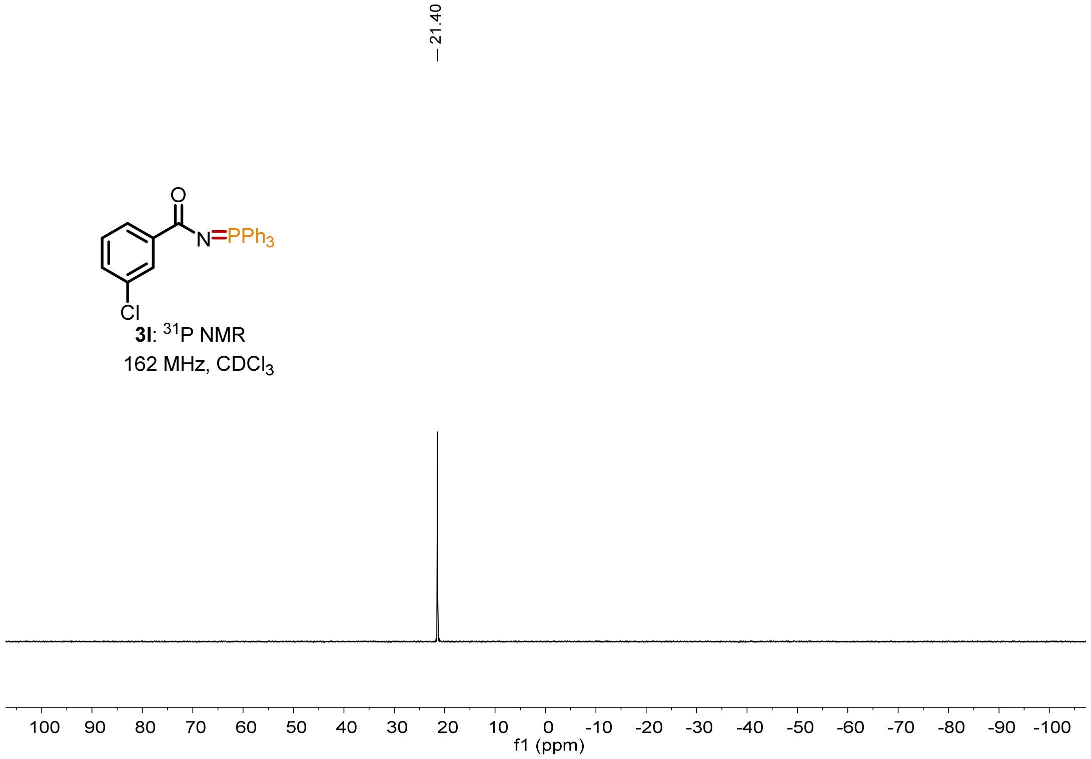
21.40
O
N=PPh3
Cl
3l: 31P NMR
162 MHz, CDCl3
100 90 80 70 60 50 40 30 20 10 0 -10 -20 -30 -40 -50 -60 -70 -80 -90 -100
f1 (ppm)

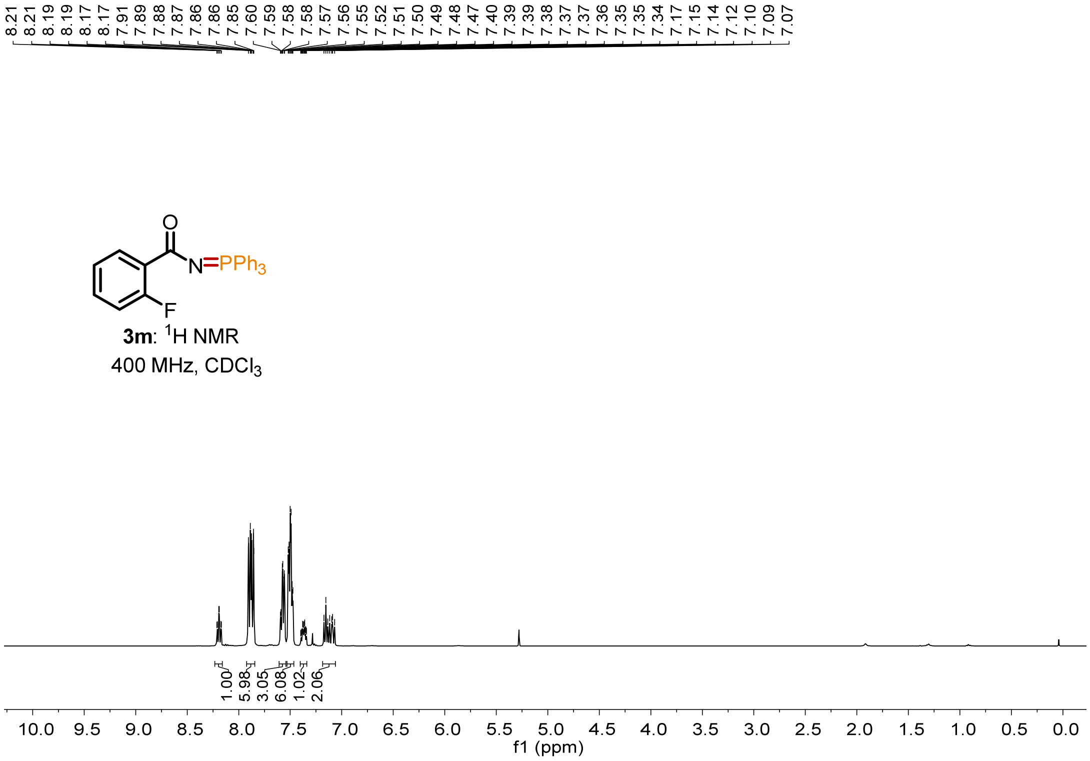
8.21 8.21 8.19 8.19 8.17 8.17 7.91 7.89 7.88 7.87 7.86 7.86 7.85 7.60 7.59 7.58 7.58 7.57 7.56 7.55 7.52 7.51 7.50 7.49 7.48 7.47 7.40 7.39 7.39 7.38 7.37 7.37 7.36 7.35 7.35 7.34 7.17 7.15 7.14 7.12 7.10 7.09 7.07
O
N
PPh3
F
3m: 1H NMR
400 MHz, CDCl3
1.00 5.98 3.05 6.08 1.02 2.06
f1 (ppm)

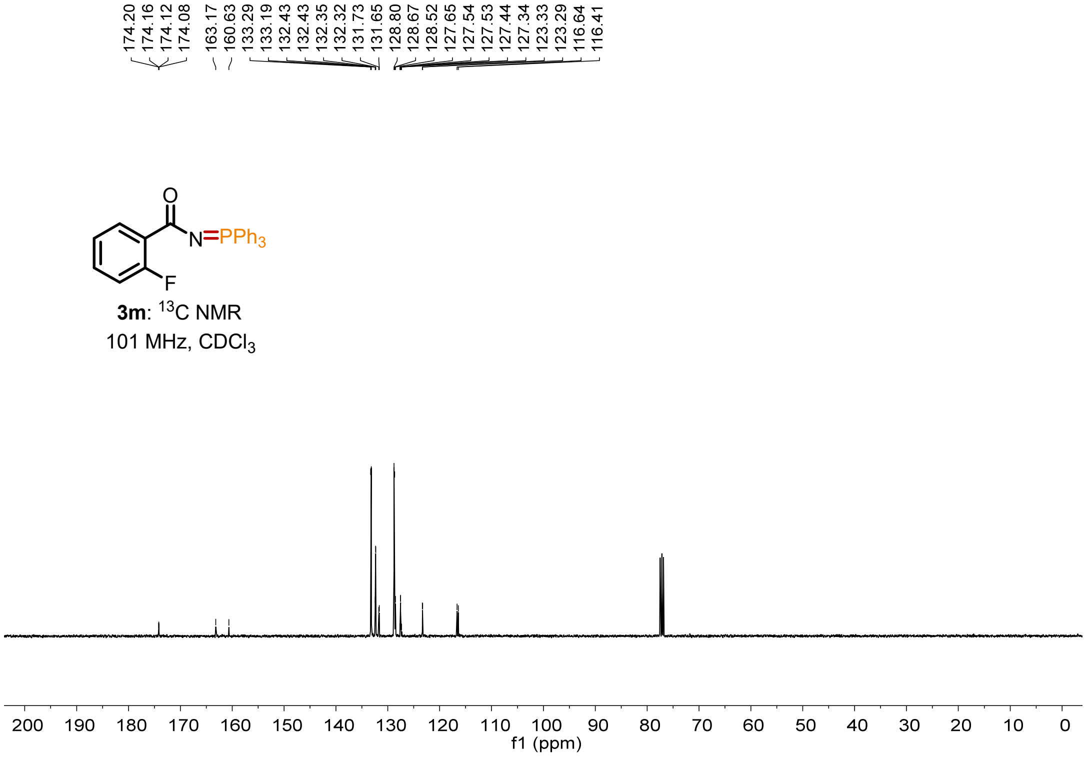
174.20 174.16 174.12 174.08 163.17 160.63 133.29 133.19 132.43 132.43 132.35 132.32 131.73 131.65 128.80 128.67 128.52 127.65 127.54 127.53 127.44 127.34 123.33 123.29 116.64 116.41
O
N
PPh3
F
3m: 13C NMR
101 MHz, CDCl3
f1 (ppm)

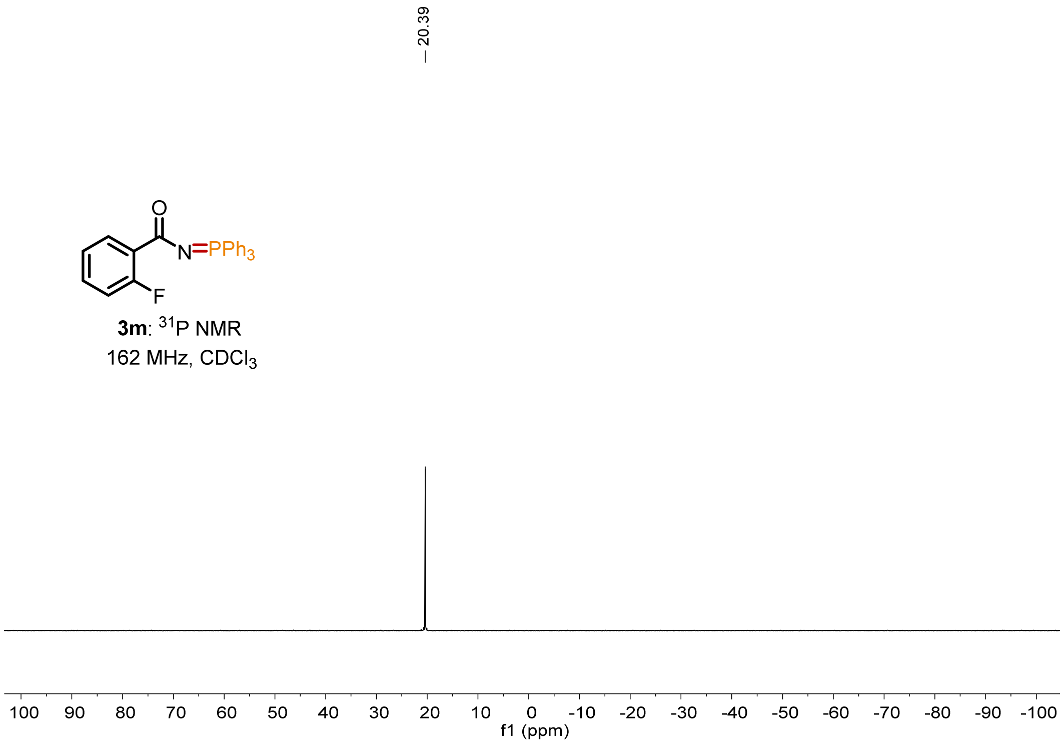
20.39
O
N=PPh3
F
3m: 31P NMR
162 MHz, CDCl3
100 90 80 70 60 50 40 30 20 10 0 -10 -20 -30 -40 -50 -60 -70 -80 -90 -100
f1 (ppm)

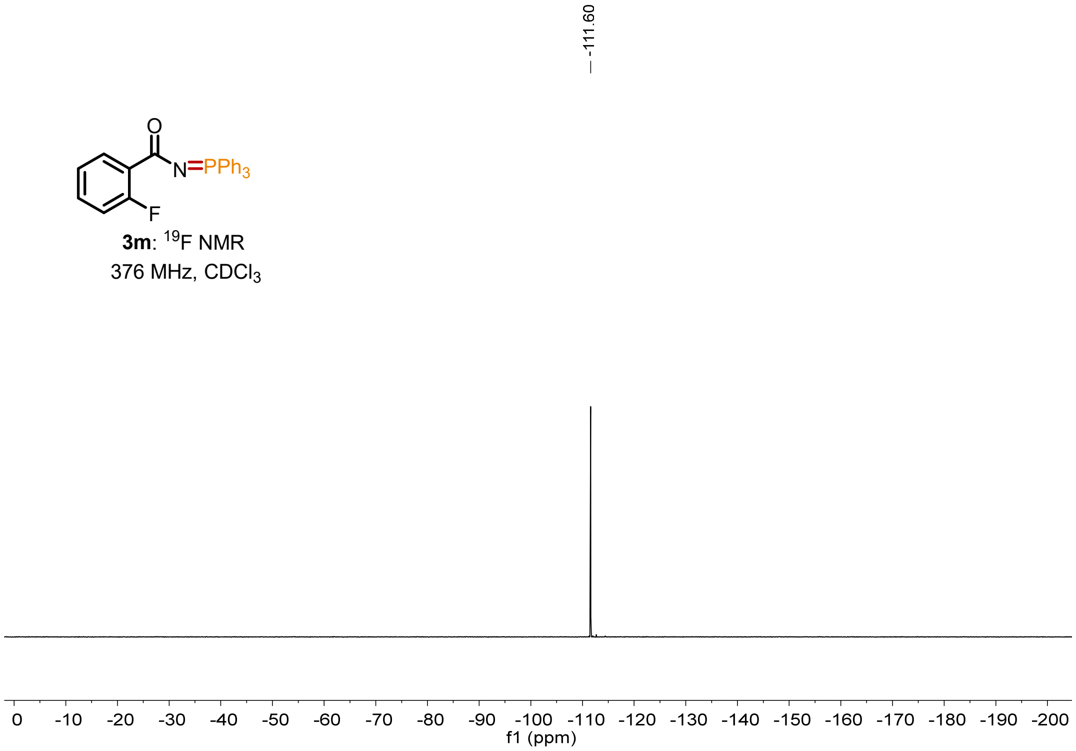
-111.60
O
N=PPh3
F
3m: 19F NMR
376 MHz, CDCl3
0 -10 -20 -30 -40 -50 -60 -70 -80 -90 -100 -110 -120 -130 -140 -150 -160 -170 -180 -190 -200
f1 (ppm)

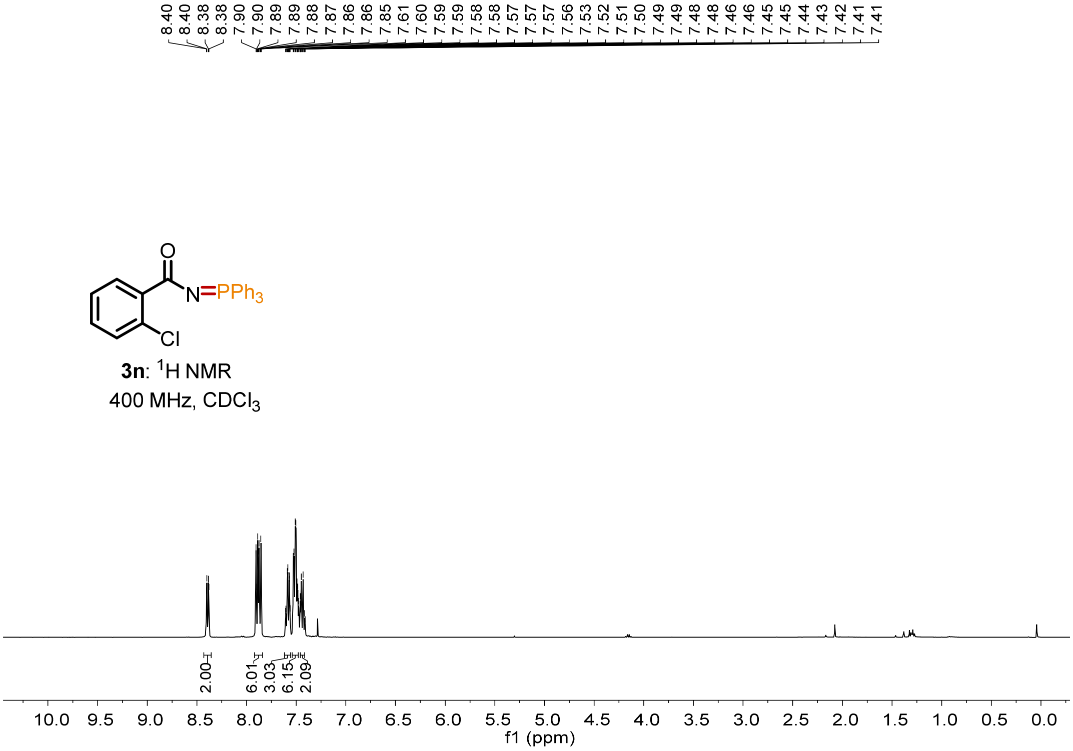
8.40
8.40
8.38
8.38
7.90
7.90
7.89
7.89
7.88
7.87
7.86
7.86
7.85
7.61
7.60
7.59
7.59
7.58
7.58
7.57
7.57
7.57
7.56
7.53
7.52
7.51
7.50
7.49
7.49
7.48
7.48
7.46
7.46
7.45
7.45
7.44
7.43
7.42
7.41
7.41
O
N=PPh3
Cl
3n: 1H NMR
400 MHz, CDCl3
2.00
6.01
3.03
6.15
2.09
10.0 9.5 9.0 8.5 8.0 7.5 7.0 6.5 6.0 5.5 5.0 4.5 4.0 3.5 3.0 2.5 2.0 1.5 1.0 0.5 0.0
f1 (ppm)

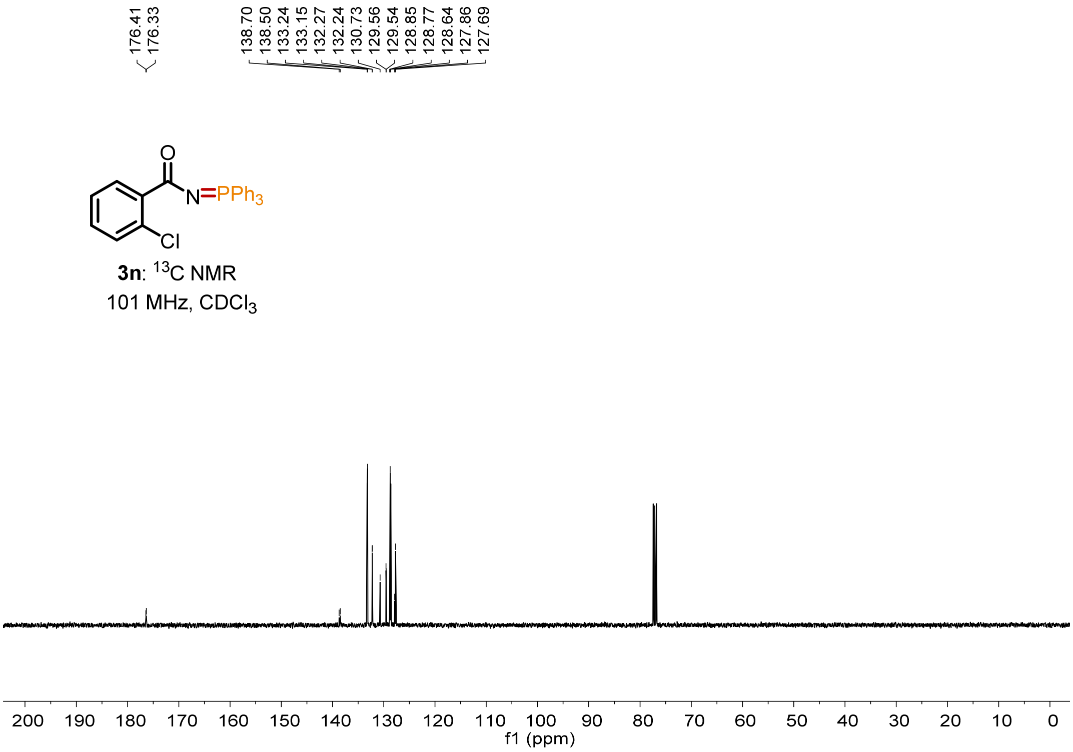
176.41
176.33
138.70
138.50
133.24
133.15
132.27
132.24
130.73
129.56
129.54
128.85
128.77
128.64
127.86
127.69
O
N=PPh3
Cl
3n: 13C NMR
101 MHz, CDCl3
200 190 180 170 160 150 140 130 120 110 100 90 80 70 60 50 40 30 20 10 0
f1 (ppm)

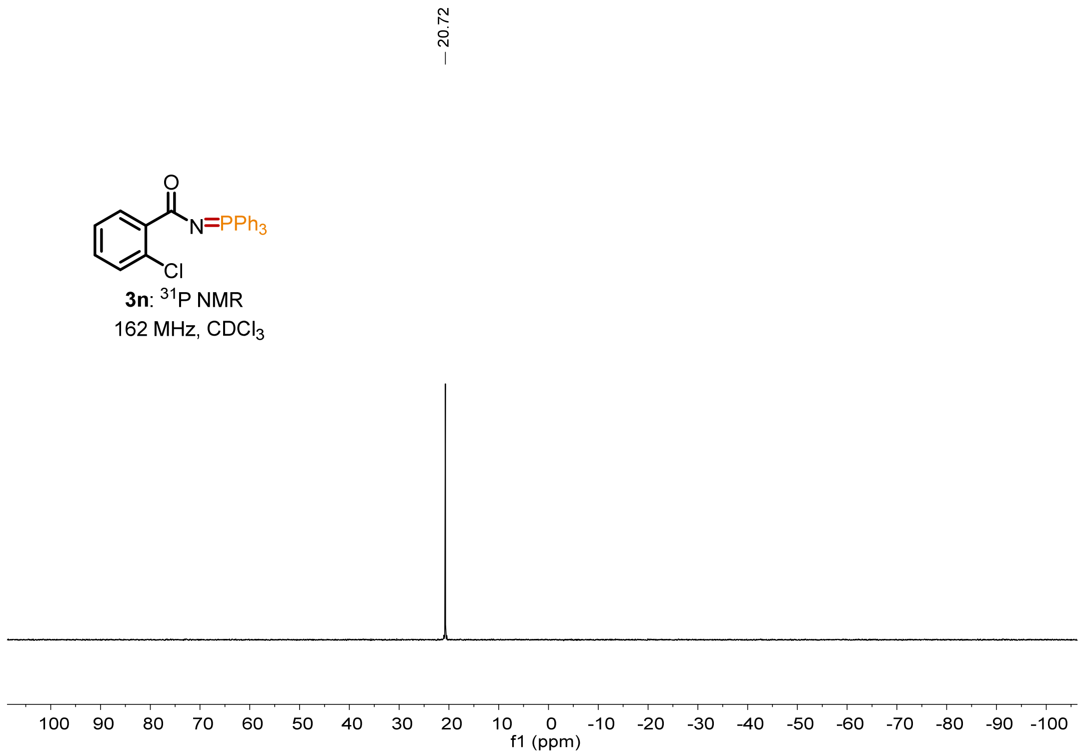
20.72
3n: ³¹P NMR
162 MHz, CDCl3
f1 (ppm)

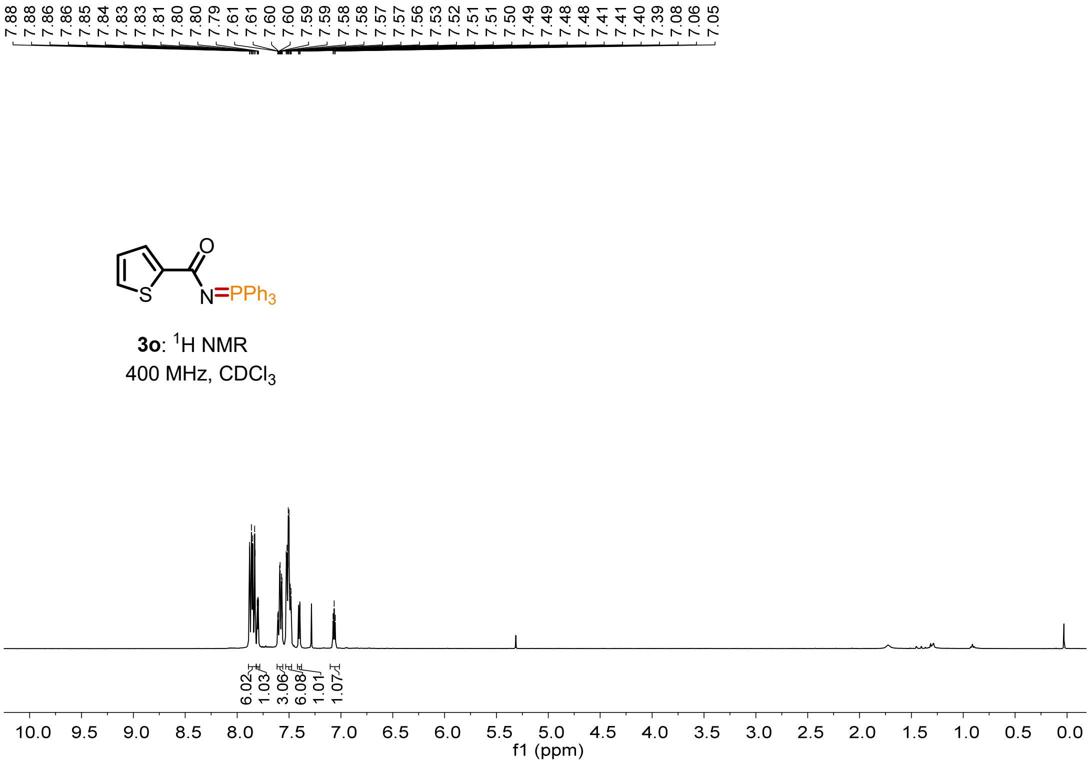
7.88 7.88 7.86 7.86 7.85 7.84 7.83 7.81 7.80 7.80 7.79 7.61 7.61 7.60 7.60 7.59 7.59 7.58 7.58 7.57 7.57 7.56 7.53 7.52 7.51 7.51 7.50 7.49 7.49 7.48 7.48 7.41 7.40 7.39 7.08 7.06 7.05
3o: ¹H NMR
400 MHz, CDCl3
6.02 1.03 3.06 6.08 1.01 1.07
f1 (ppm)

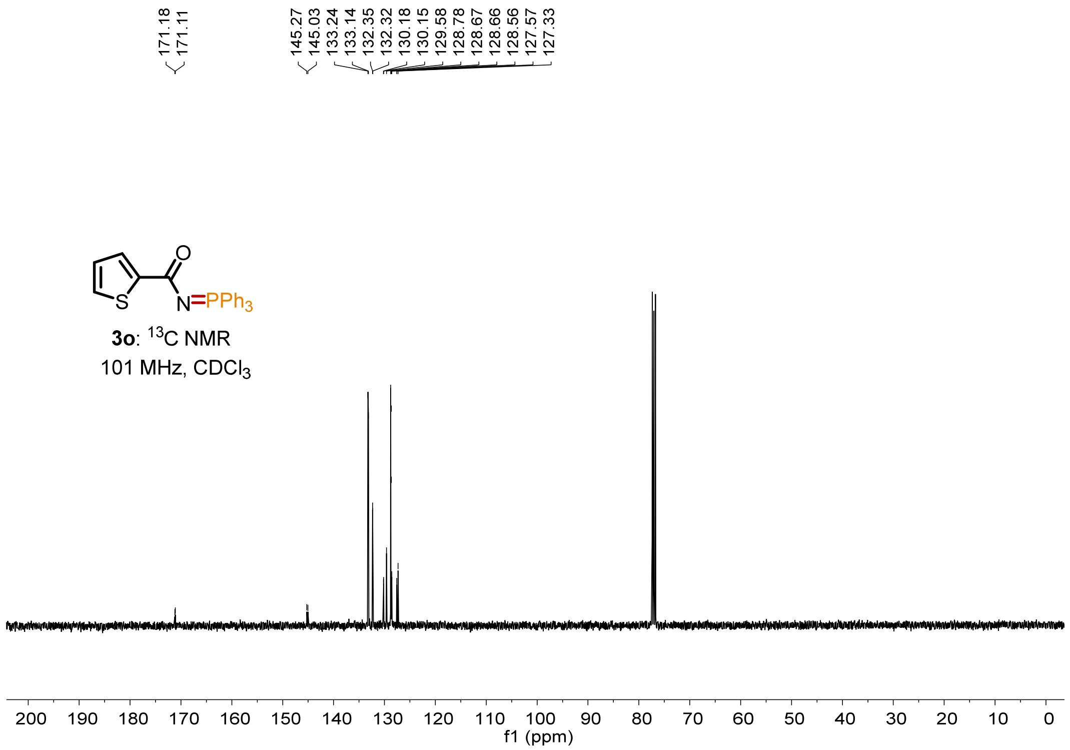
171.18
171.11
145.27
145.03
133.24
133.14
132.35
132.32
130.18
130.15
129.58
128.78
128.67
128.66
128.56
127.57
127.33
N=PPh3
3o: 13C NMR
101 MHz, CDCl3
200 190 180 170 160 150 140 130 120 110 100 90 80 70 60 50 40 30 20 10 0
f1 (ppm)

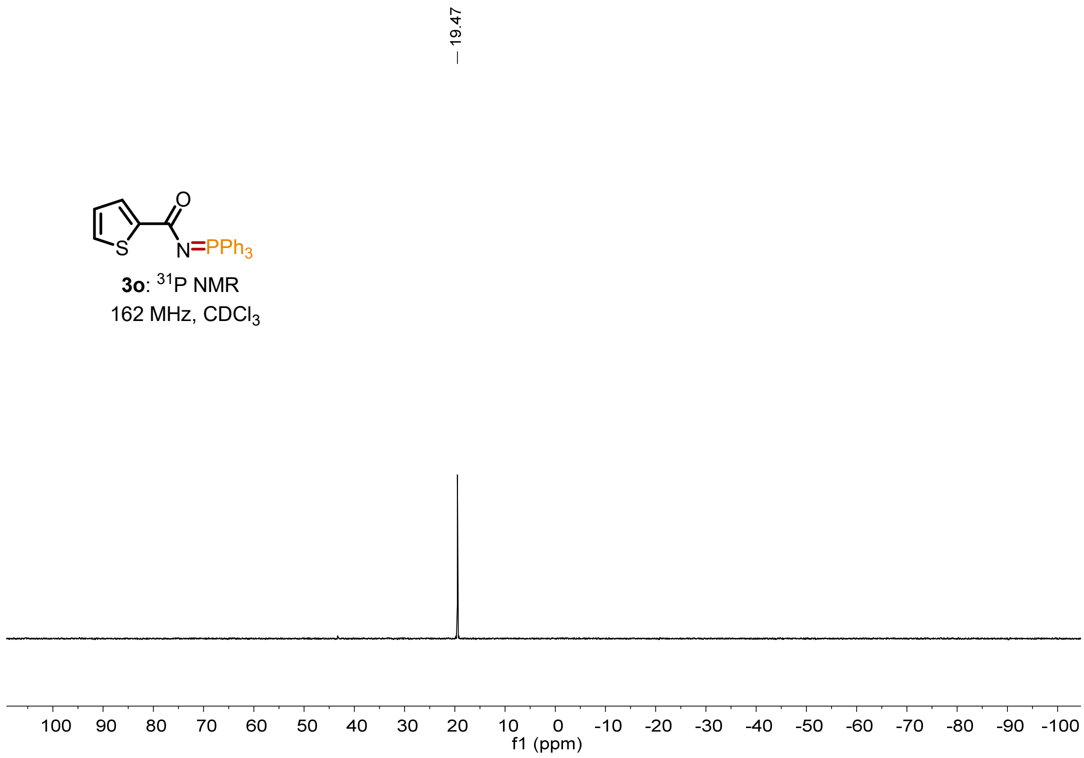
19.47
N=PPh3
3o: 31P NMR
162 MHz, CDCl3
100 90 80 70 60 50 40 30 20 10 0 -10 -20 -30 -40 -50 -60 -70 -80 -90 -100
f1 (ppm)

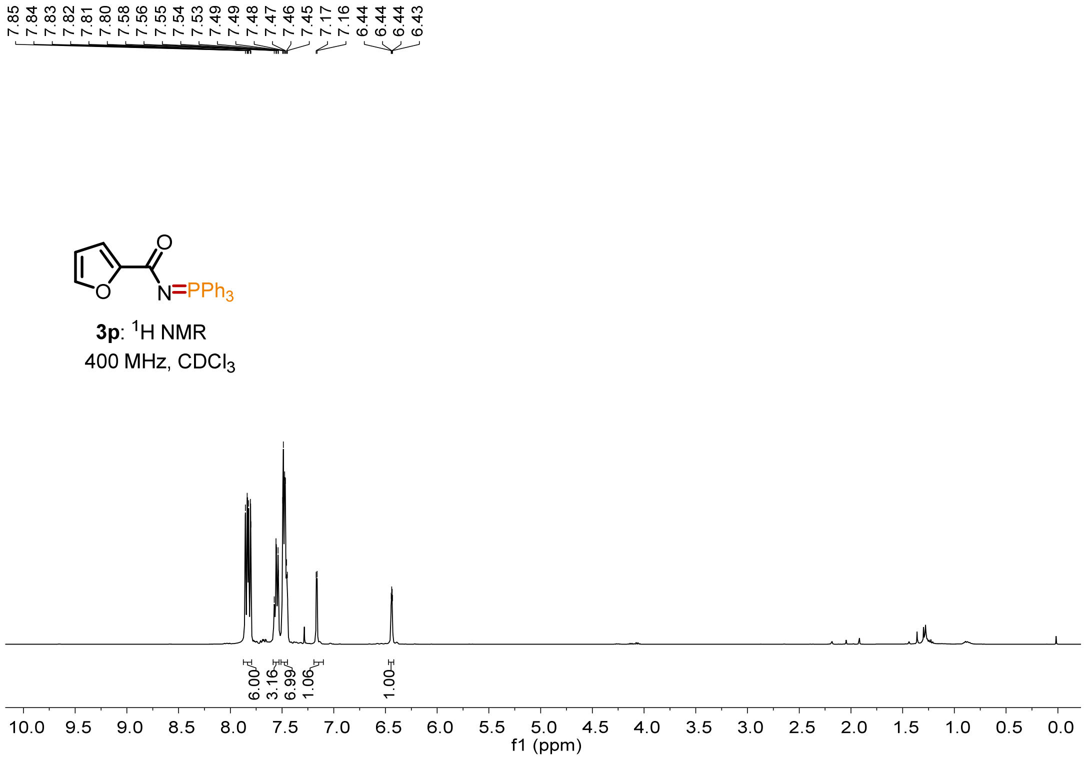
7.85 7.84 7.83 7.82 7.81 7.80 7.58 7.56 7.55 7.54 7.53 7.49 7.48 7.47 7.46 7.45 7.17 7.16 6.44 6.44 6.44 6.43
N=PPh3
3p: 1H NMR
400 MHz, CDCl3
6.00 3.16 6.99 1.06 1.00
10.0 9.5 9.0 8.5 8.0 7.5 7.0 6.5 6.0 5.5 5.0 4.5 4.0 3.5 3.0 2.5 2.0 1.5 1.0 0.5 0.0
f1 (ppm)

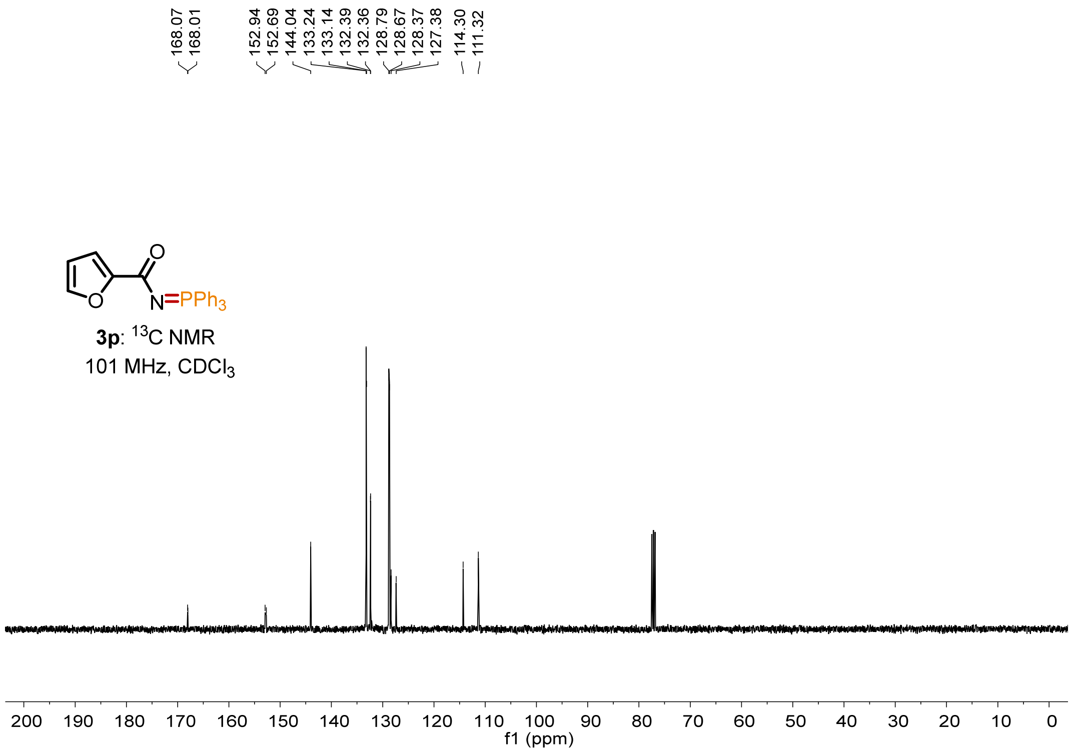
168.07 168.01 152.94 152.69 144.04 133.24 133.14 132.39 132.36 128.79 128.67 128.37 127.38 114.30 111.32
N=PPh3
3p: 13C NMR
101 MHz, CDCl3
200 190 180 170 160 150 140 130 120 110 100 90 80 70 60 50 40 30 20 10 0
f1 (ppm)

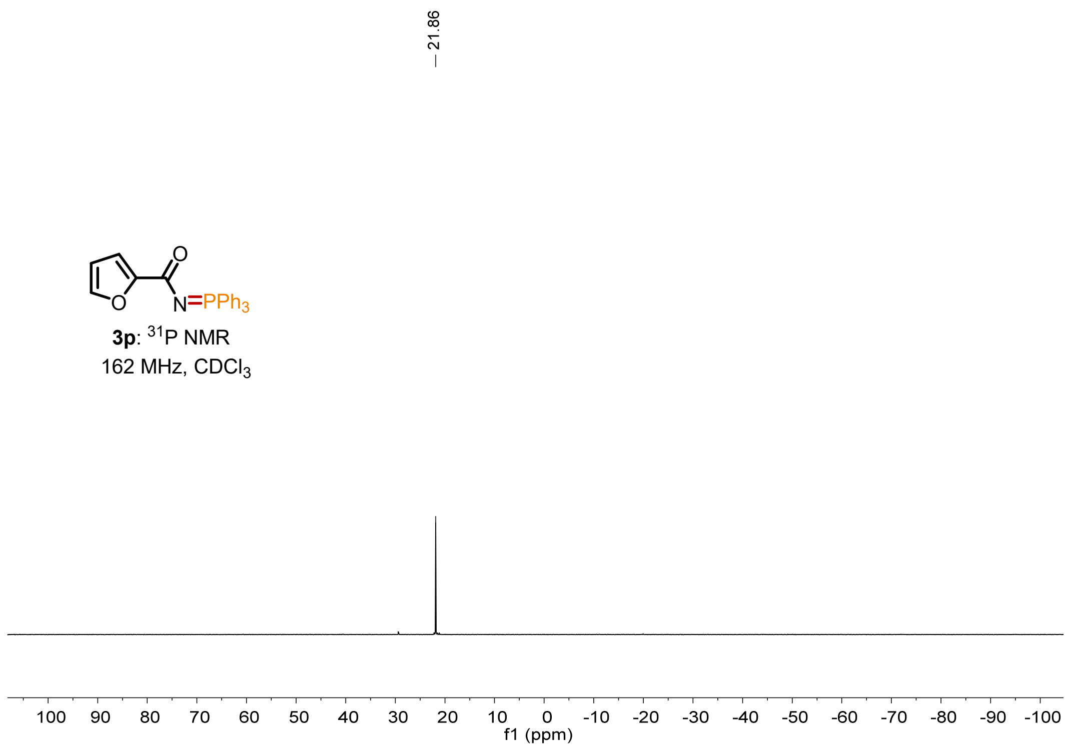
O
N=PPh3
3p: 31P NMR
162 MHz, CDCl3
21.86
100 90 80 70 60 50 40 30 20 10 0 -10 -20 -30 -40 -50 -60 -70 -80 -90 -100
f1 (ppm)

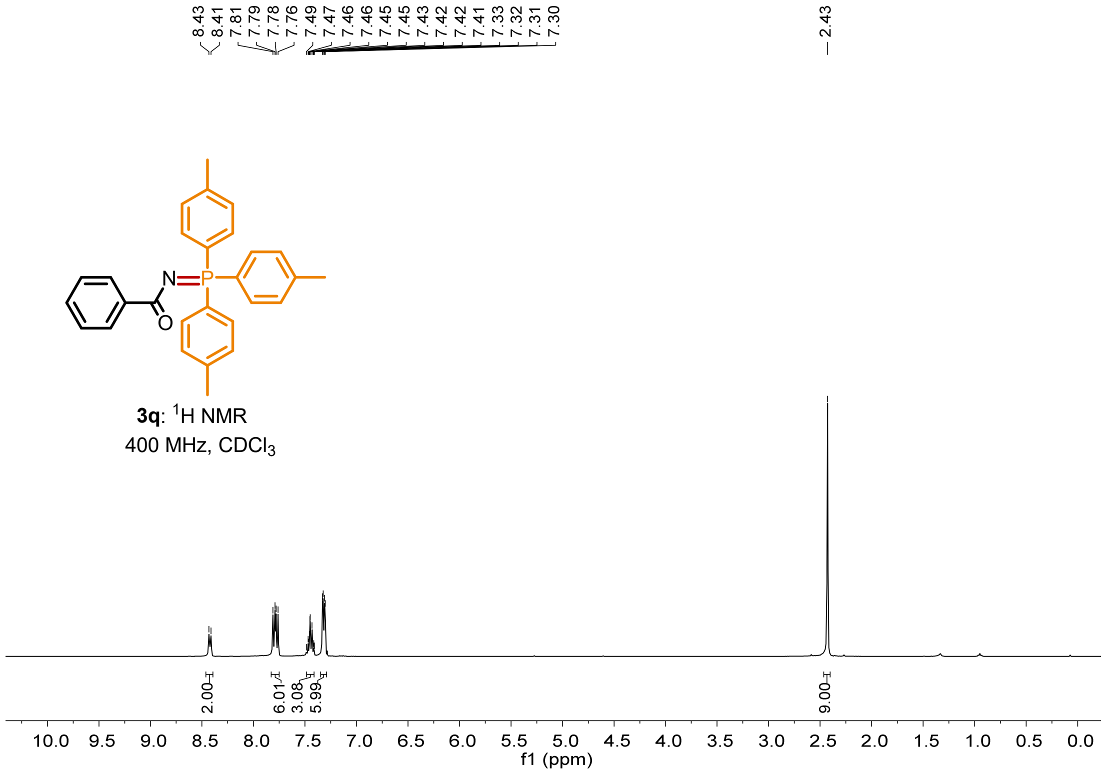
8.43
8.41
7.81
7.79
7.78
7.76
7.49
7.47
7.46
7.46
7.45
7.45
7.43
7.42
7.42
7.41
7.33
7.32
7.31
7.30
2.43
N=P
O
3q: 1H NMR
400 MHz, CDCl3
2.00
6.01
3.08
5.99
9.00
10.0 9.5 9.0 8.5 8.0 7.5 7.0 6.5 6.0 5.5 5.0 4.5 4.0 3.5 3.0 2.5 2.0 1.5 1.0 0.5 0.0
f1 (ppm)

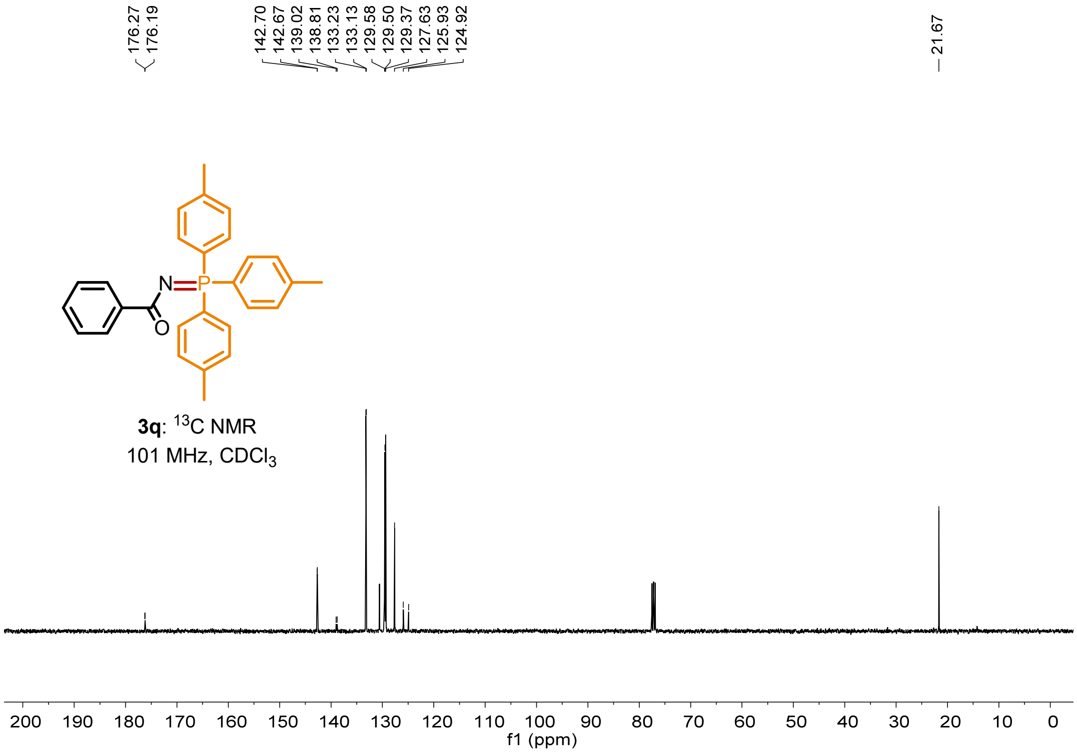

176.27
176.19
142.70
142.67
139.02
138.81
133.23
133.13
129.58
129.50
129.37
127.63
125.93
124.92
21.67
3q: 13C NMR
101 MHz, CDCl3
200 190 180 170 160 150 140 130 120 110 100 90 80 70 60 50 40 30 20 10 0
f1 (ppm)

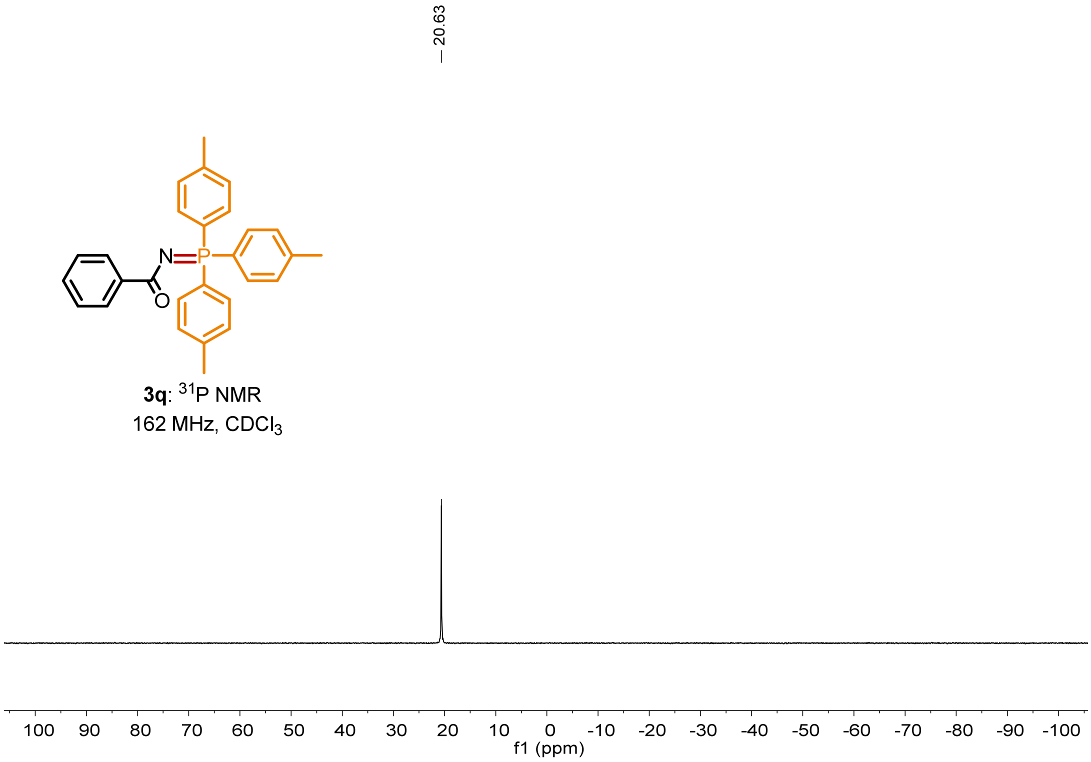

20.63
3q: 31P NMR
162 MHz, CDCl3
100 90 80 70 60 50 40 30 20 10 0 -10 -20 -30 -40 -50 -60 -70 -80 -90 -100
f1 (ppm)

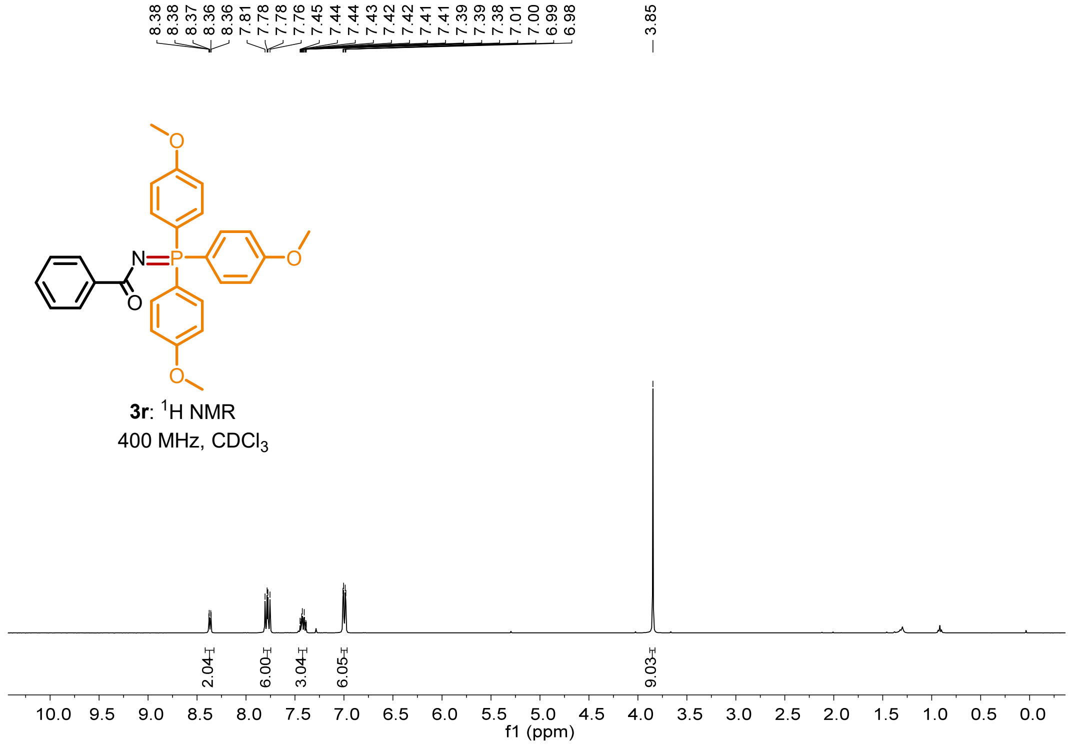
8.38
8.38
8.37
8.36
8.36
7.81
7.78
7.78
7.76
7.45
7.44
7.44
7.43
7.42
7.42
7.41
7.41
7.39
7.39
7.38
7.01
7.00
6.99
6.98
3.85
3r: 1H NMR
400 MHz, CDCl3
2.04
6.00
3.04
6.05
9.03
f1 (ppm)

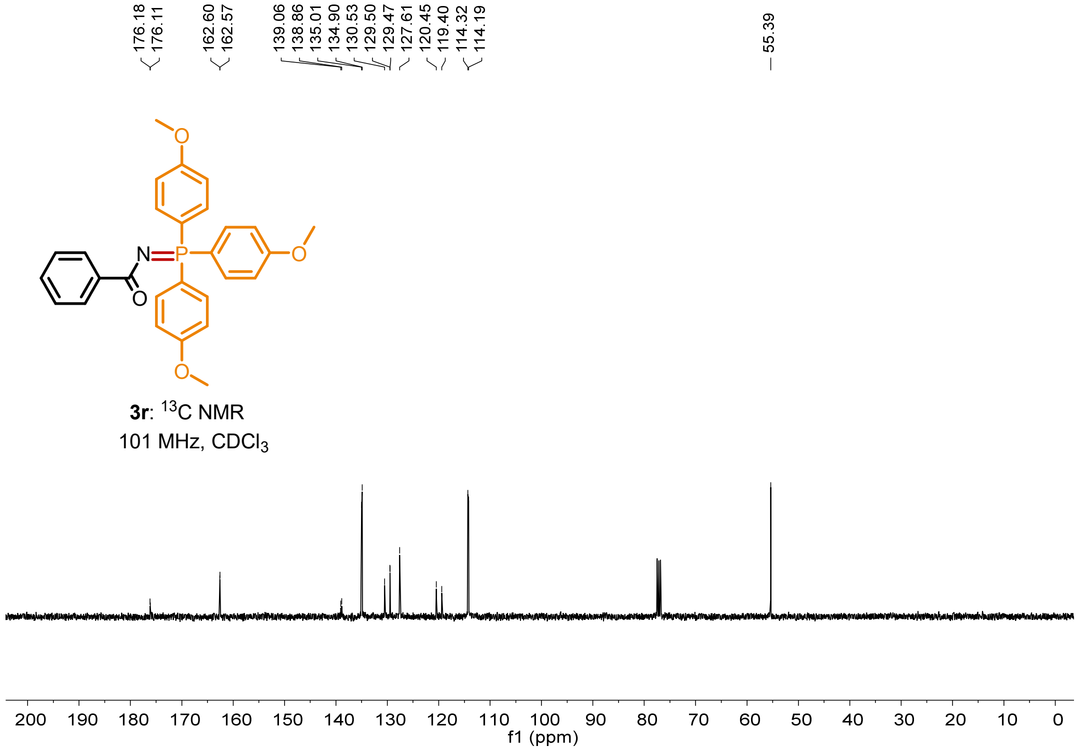
176.18
176.11
162.60
162.57
139.06
138.86
135.01
134.90
130.53
129.50
129.47
127.61
120.45
119.40
114.32
114.19
55.39
3r: 13C NMR
101 MHz, CDCl3
f1 (ppm)

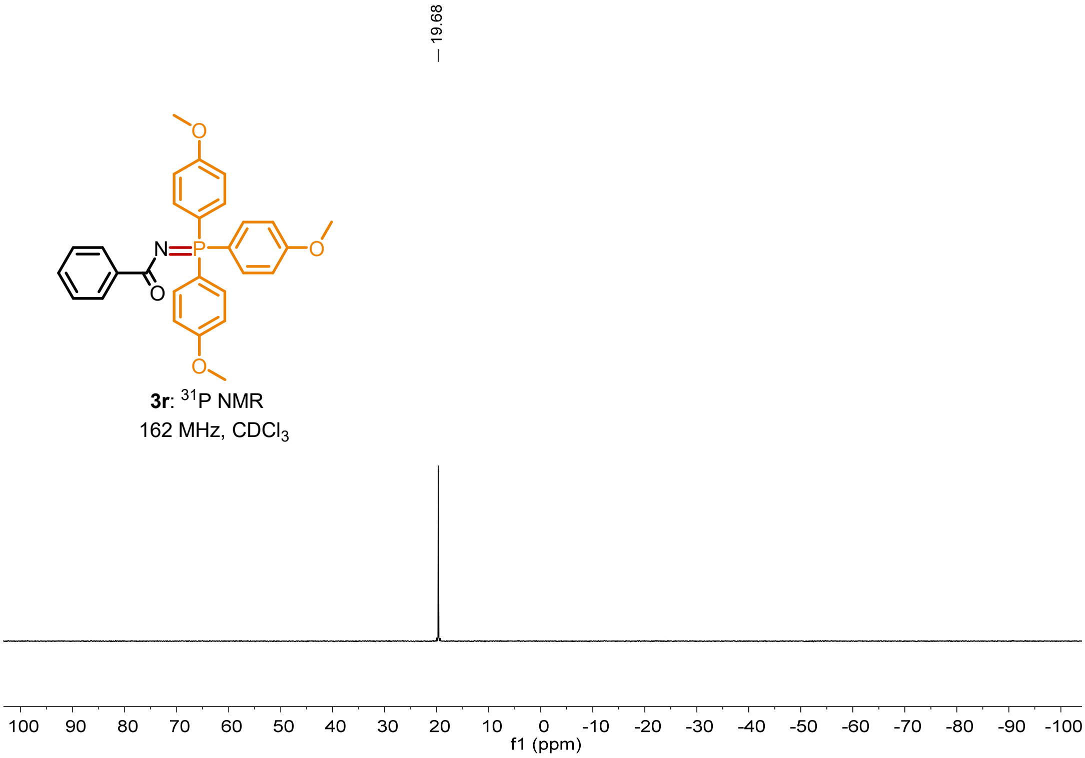

19.68
3r: ^{31}P NMR
162 MHz, $CDCl_3$
100 90 80 70 60 50 40 30 20 10 0 -10 -20 -30 -40 -50 -60 -70 -80 -90 -100
f1 (ppm)

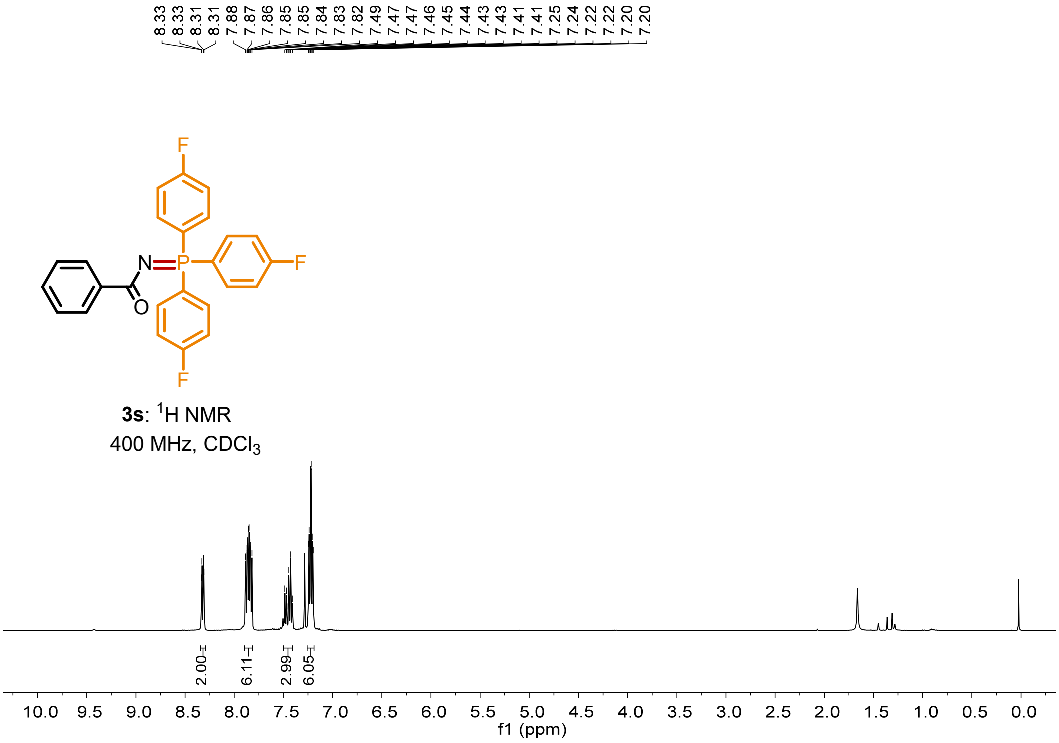

8.33 8.33 8.31 8.31 7.88 7.87 7.86 7.85 7.85 7.84 7.83 7.82 7.49 7.47 7.47 7.46 7.45 7.44 7.43 7.43 7.41 7.41 7.25 7.24 7.22 7.22 7.20 7.20
3s: ^{1}H NMR
400 MHz, $CDCl_3$
2.00 6.11 2.99 6.05
10.0 9.5 9.0 8.5 8.0 7.5 7.0 6.5 6.0 5.5 5.0 4.5 4.0 3.5 3.0 2.5 2.0 1.5 1.0 0.5 0.0
f1 (ppm)

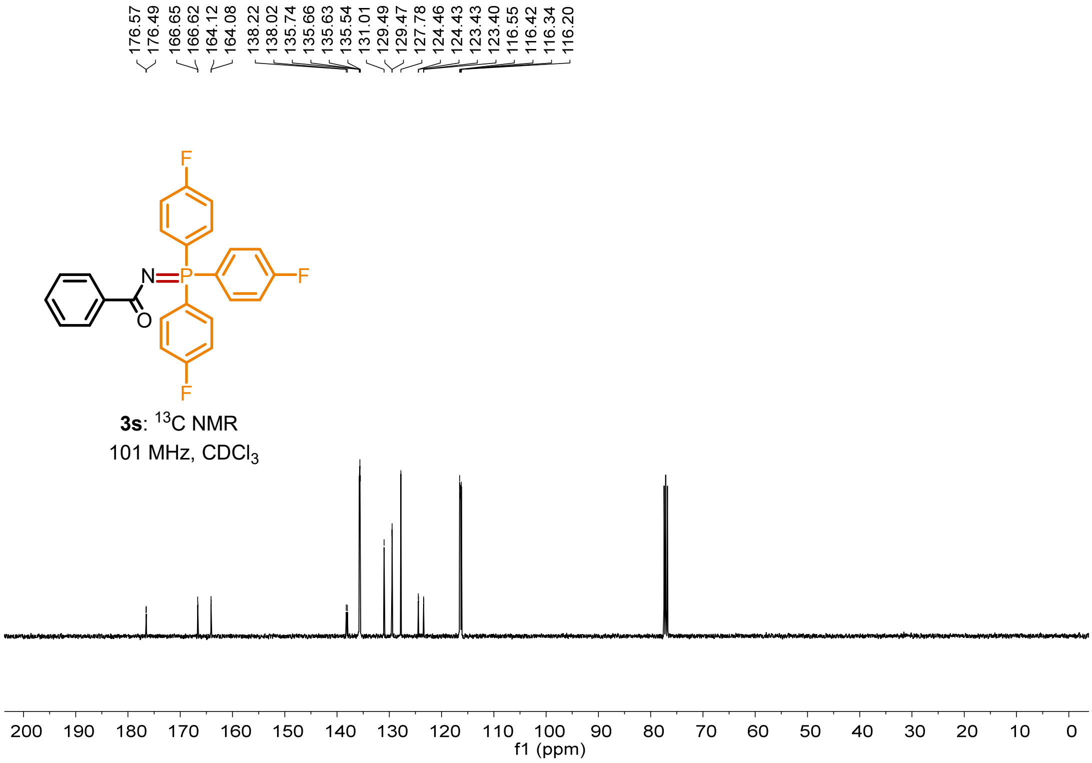
176.57
176.49
166.65
166.62
164.12
164.08
138.22
138.02
135.74
135.66
135.63
135.54
131.01
129.49
129.47
127.78
124.46
124.43
123.43
123.40
116.55
116.42
116.34
116.20
N
P
O
F
F
F
3s: 13C NMR
101 MHz, CDCl3
200 190 180 170 160 150 140 130 120 110 100 90 80 70 60 50 40 30 20 10 0
f1 (ppm)

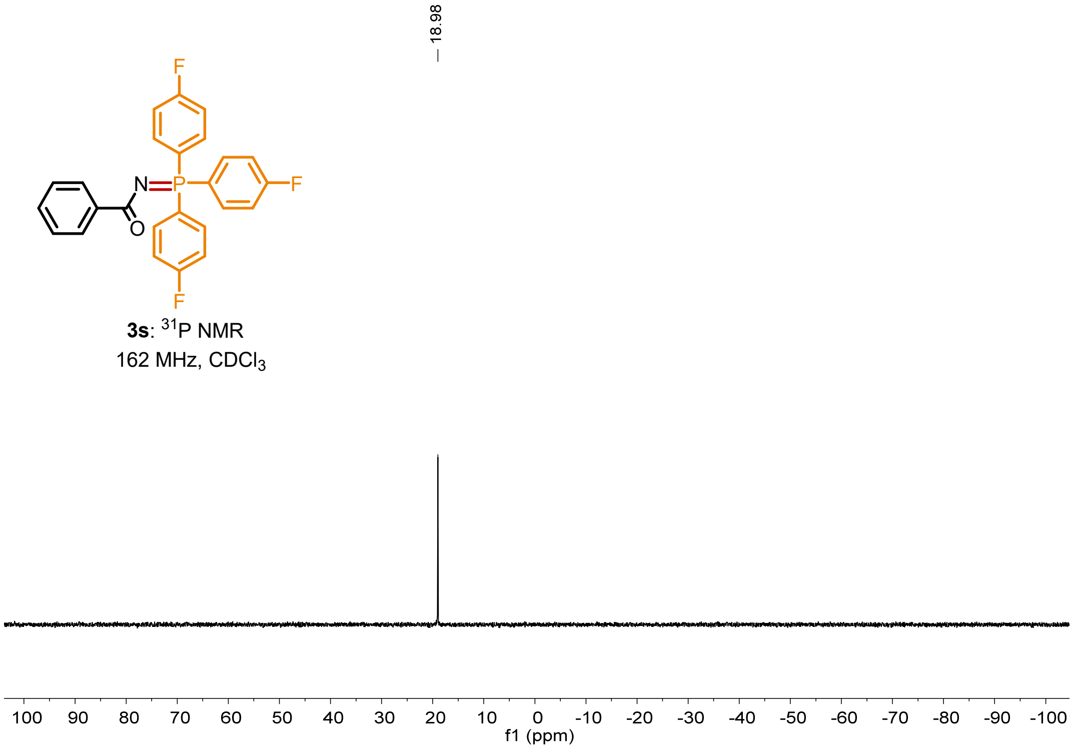
18.98
N
P
O
F
F
F
3s: 31P NMR
162 MHz, CDCl3
100 90 80 70 60 50 40 30 20 10 0 -10 -20 -30 -40 -50 -60 -70 -80 -90 -100
f1 (ppm)

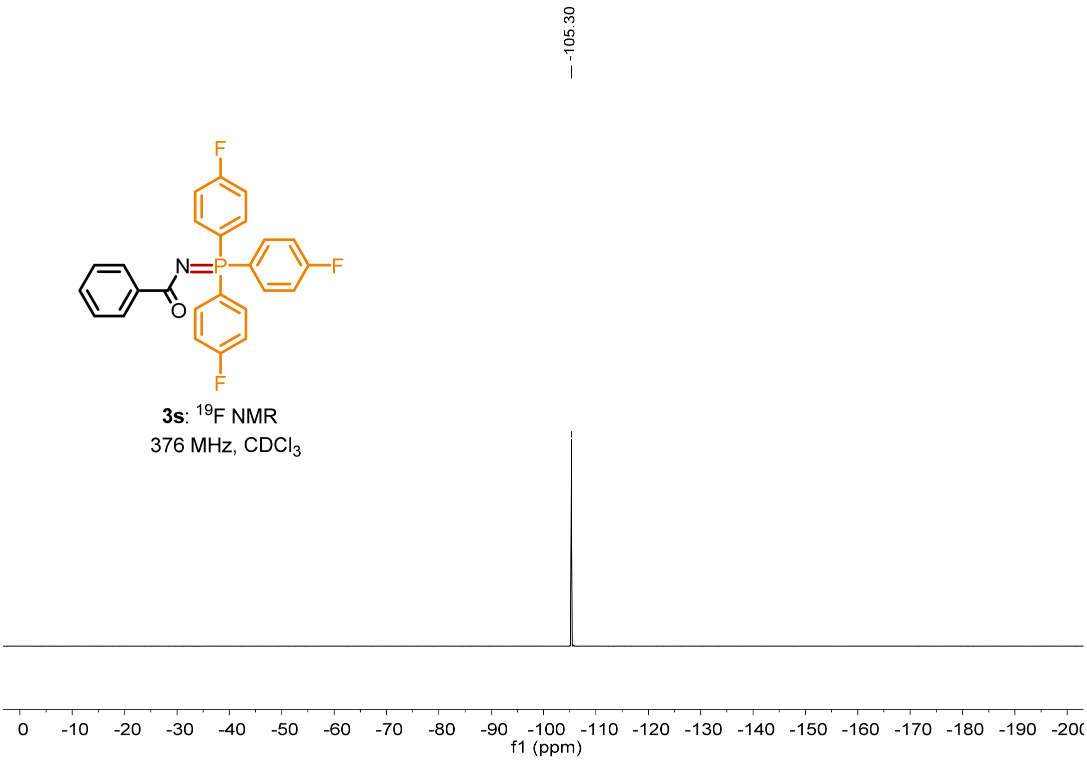
-105.30
F
N
P
O
3s: 19F NMR
376 MHz, CDCl3
0 -10 -20 -30 -40 -50 -60 -70 -80 -90 -100 -110 -120 -130 -140 -150 -160 -170 -180 -190 -200
f1 (ppm)

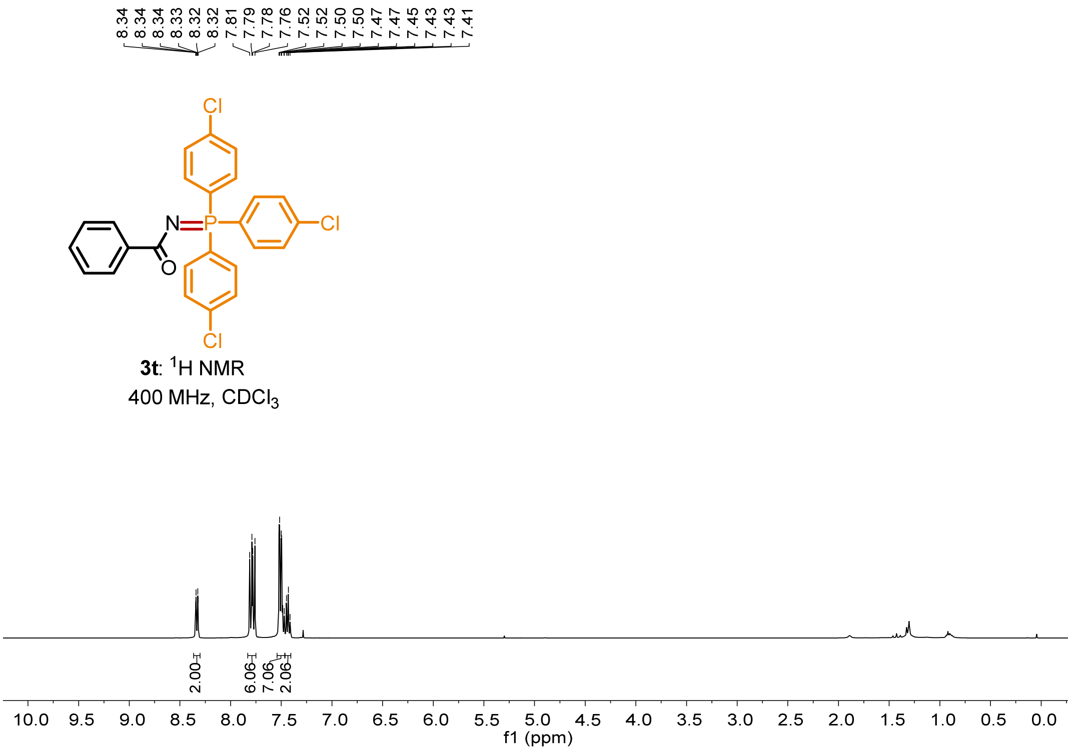
8.34 8.34 8.34 8.33 8.32 8.32 7.81 7.79 7.78 7.76 7.52 7.52 7.50 7.50 7.47 7.47 7.45 7.43 7.43 7.41
Cl
N
P
O
3t: 1H NMR
400 MHz, CDCl3
2.00
6.06
7.06
2.06
10.0 9.5 9.0 8.5 8.0 7.5 7.0 6.5 6.0 5.5 5.0 4.5 4.0 3.5 3.0 2.5 2.0 1.5 1.0 0.5 0.0
f1 (ppm)

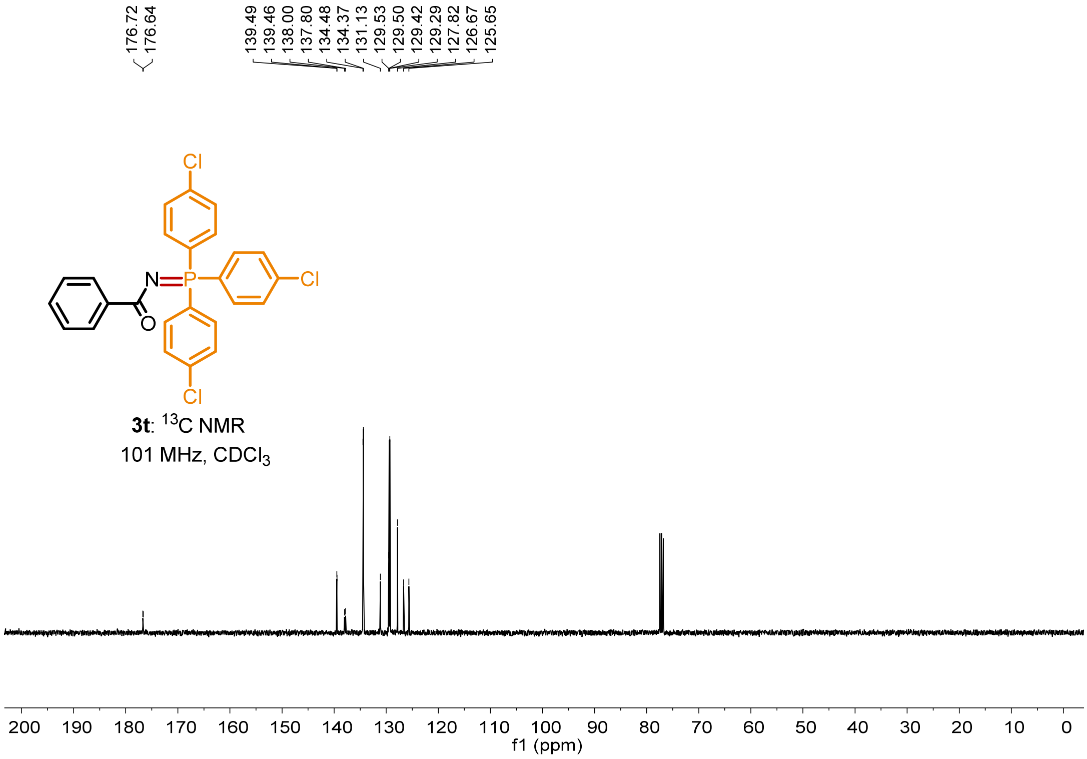
176.72
176.64
139.49
139.46
138.00
137.80
134.48
134.37
131.13
129.53
129.50
129.42
129.29
127.82
126.67
125.65
Cl
N
P
Cl
O
Cl
3t: ^{13}C NMR
101 MHz, $CDCl_3$
200 190 180 170 160 150 140 130 120 110 100 90 80 70 60 50 40 30 20 10 0
f1 (ppm)

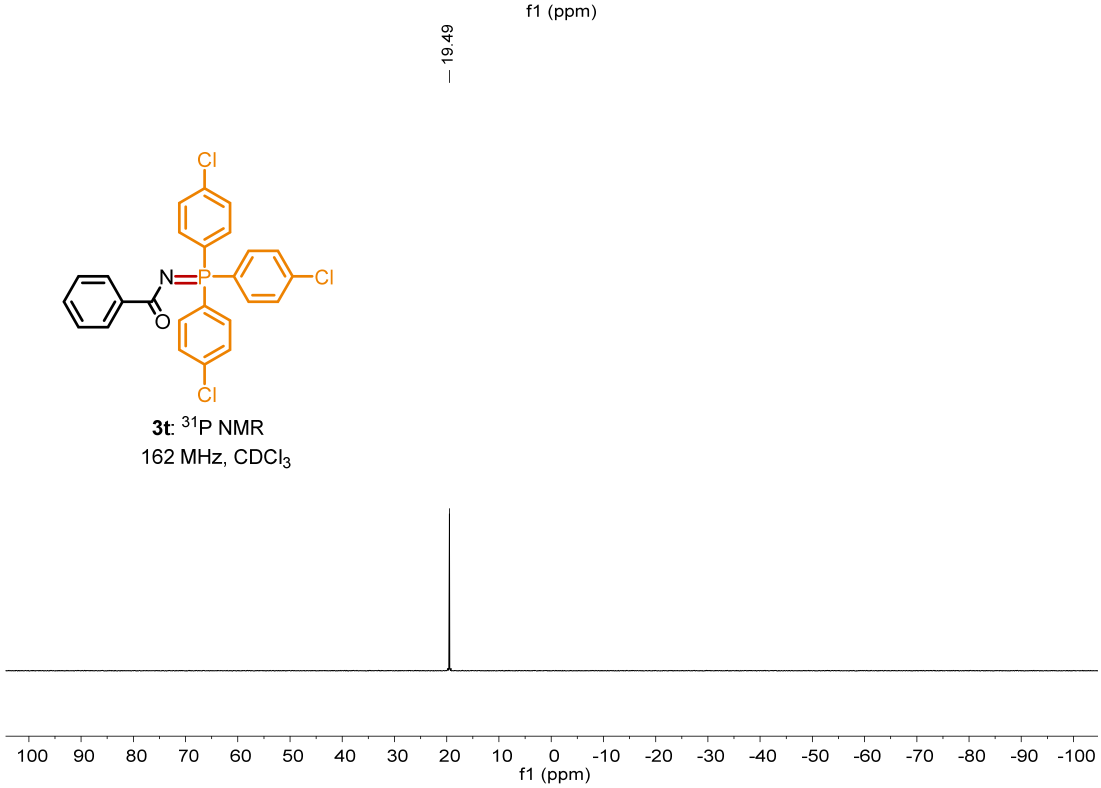
19.49
Cl
N
P
Cl
O
Cl
3t: ^{31}P NMR
162 MHz, $CDCl_3$
100 90 80 70 60 50 40 30 20 10 0 -10 -20 -30 -40 -50 -60 -70 -80 -90 -100
f1 (ppm)

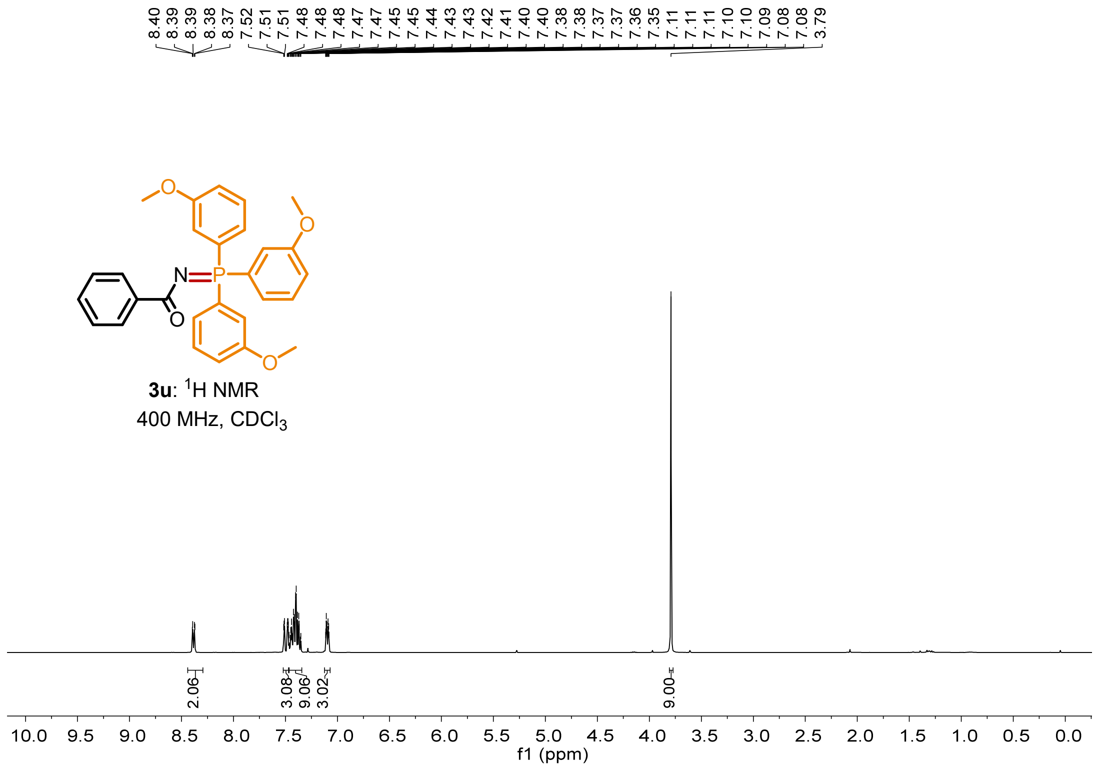
8.40 8.39 8.39 8.38 8.37 7.52 7.51 7.51 7.48 7.48 7.48 7.47 7.47 7.45 7.45 7.44 7.43 7.43 7.42 7.41 7.40 7.40 7.38 7.38 7.37 7.37 7.36 7.35 7.11 7.11 7.11 7.10 7.10 7.09 7.08 7.08 3.79
N
P
O
3u: 1H NMR
400 MHz, CDCl3
2.06
3.08
9.06
3.02
9.00
f1 (ppm)

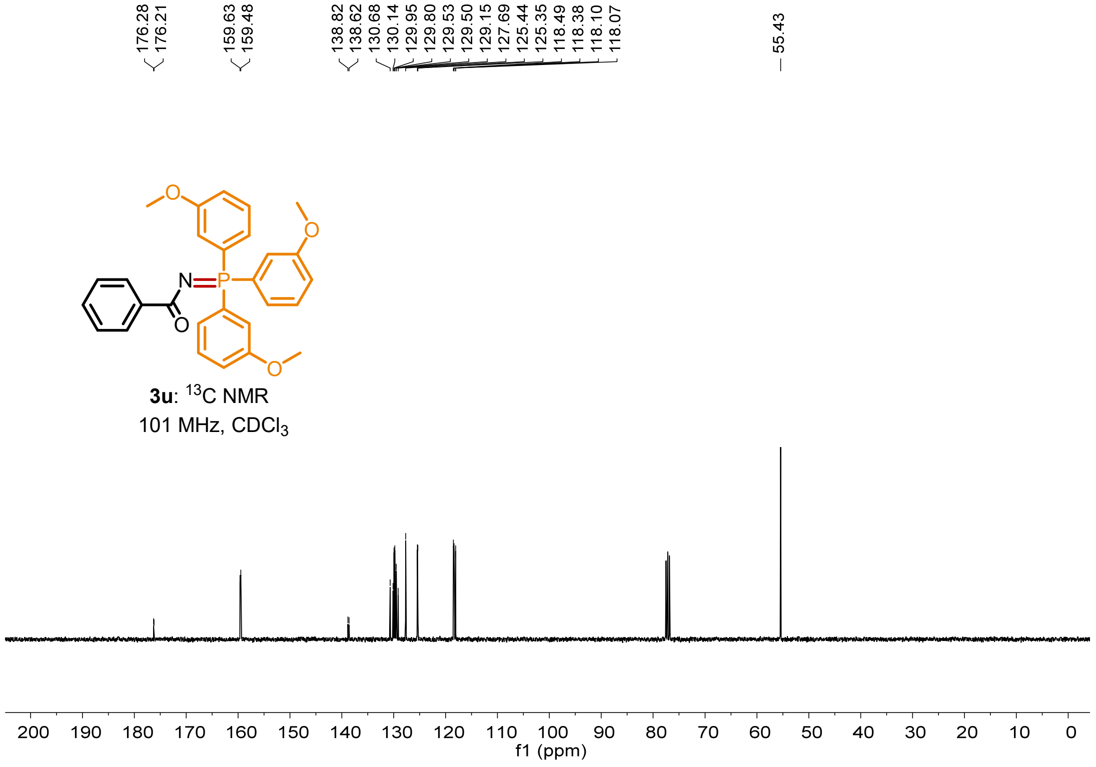
176.28 176.21
159.63 159.48
138.82 138.62 130.68 130.14 129.95 129.80 129.53 129.50 129.15 127.69 125.44 125.35 118.49 118.38 118.10 118.07
55.43
N
P
O
3u: 13C NMR
101 MHz, CDCl3
f1 (ppm)

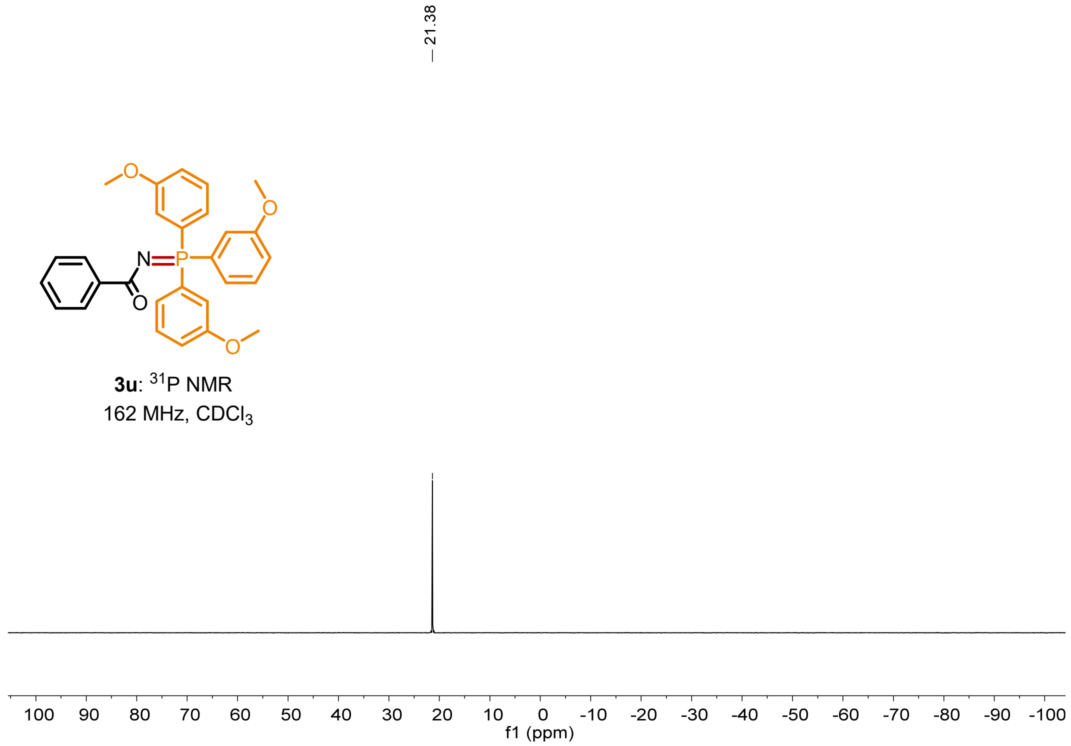

3u: ^{31}P NMR
162 MHz, $CDCl_3$

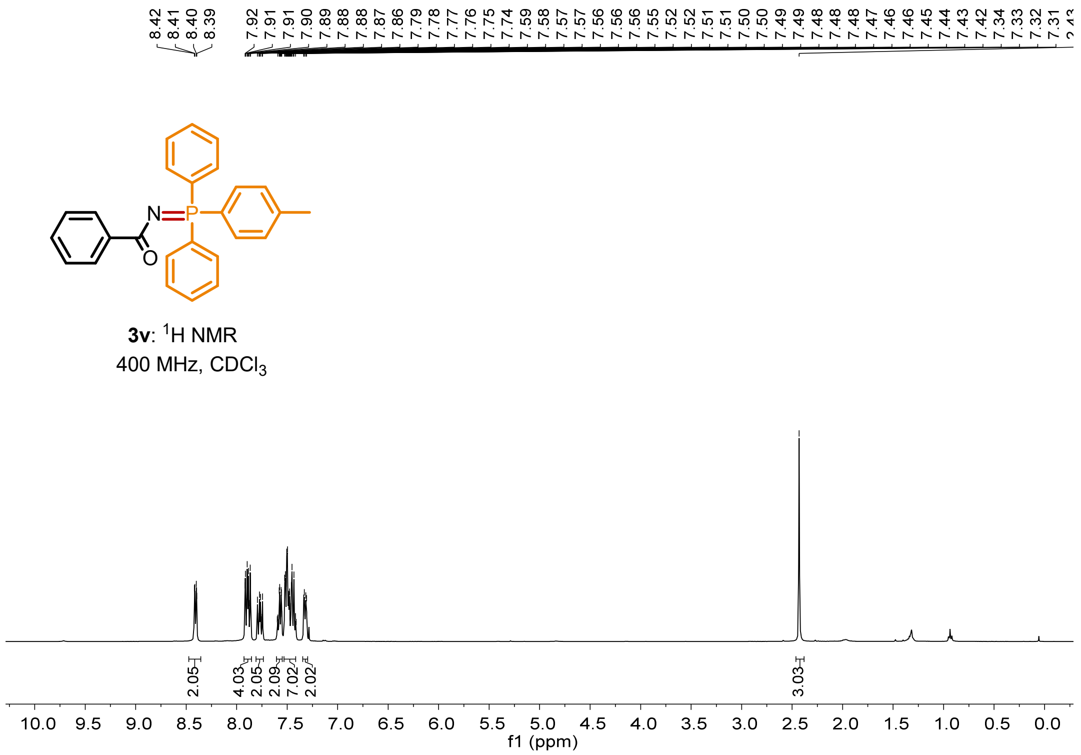

3v: ^{1}H NMR
400 MHz, $CDCl_3$

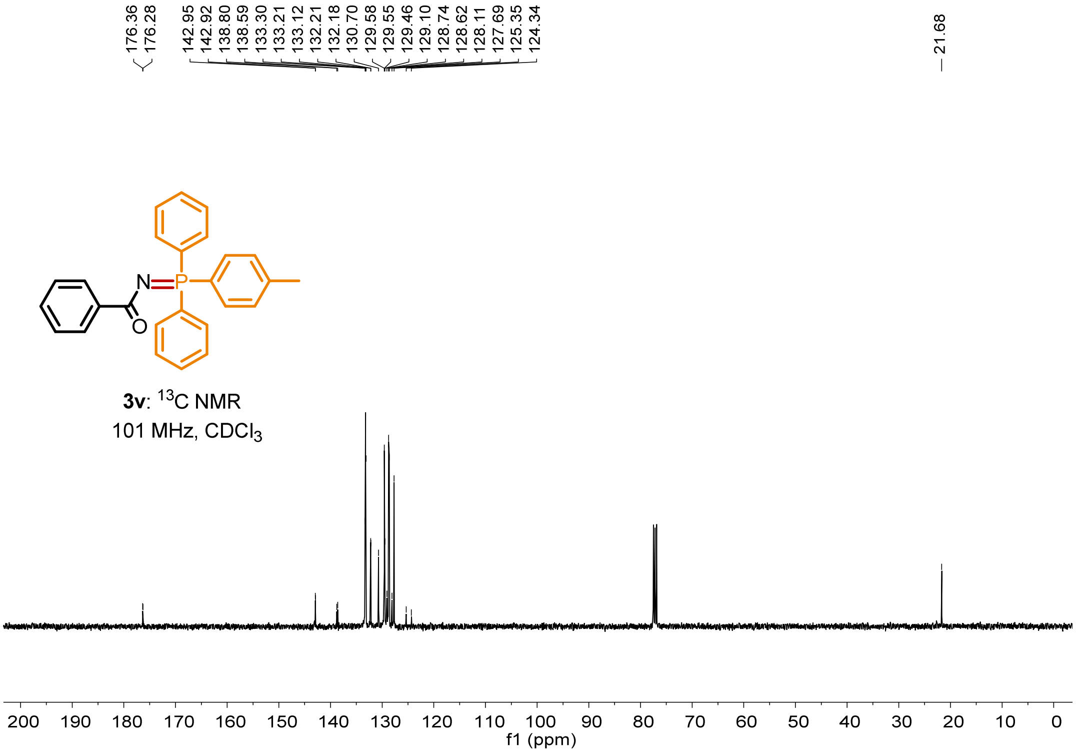
176.36
176.28
142.95
142.92
138.80
138.59
133.30
133.21
133.12
132.21
132.18
130.70
129.58
129.55
129.46
129.10
128.74
128.62
128.11
127.69
125.35
124.34
21.68
N
P
O
3v: ^{13}C NMR
101 MHz, $CDCl_3$
200 190 180 170 160 150 140 130 120 110 100 90 80 70 60 50 40 30 20 10 0
f1 (ppm)

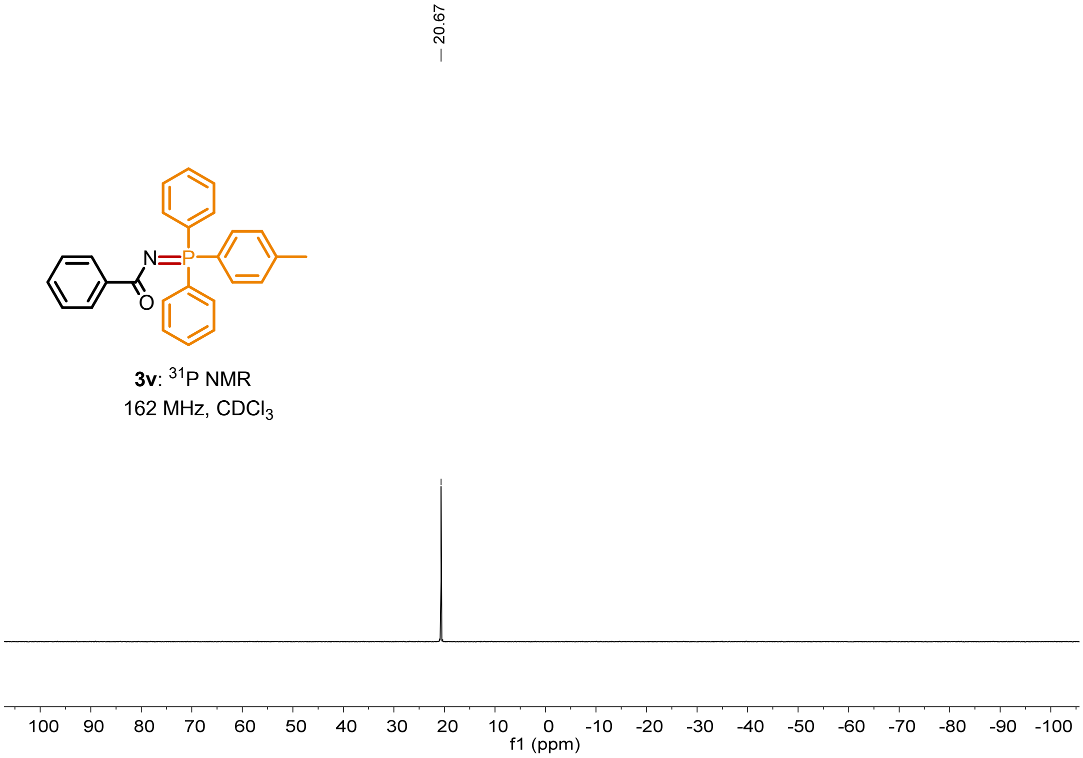
20.67
N
P
O
3v: ^{31}P NMR
162 MHz, $CDCl_3$
100 90 80 70 60 50 40 30 20 10 0 -10 -20 -30 -40 -50 -60 -70 -80 -90 -100
f1 (ppm)

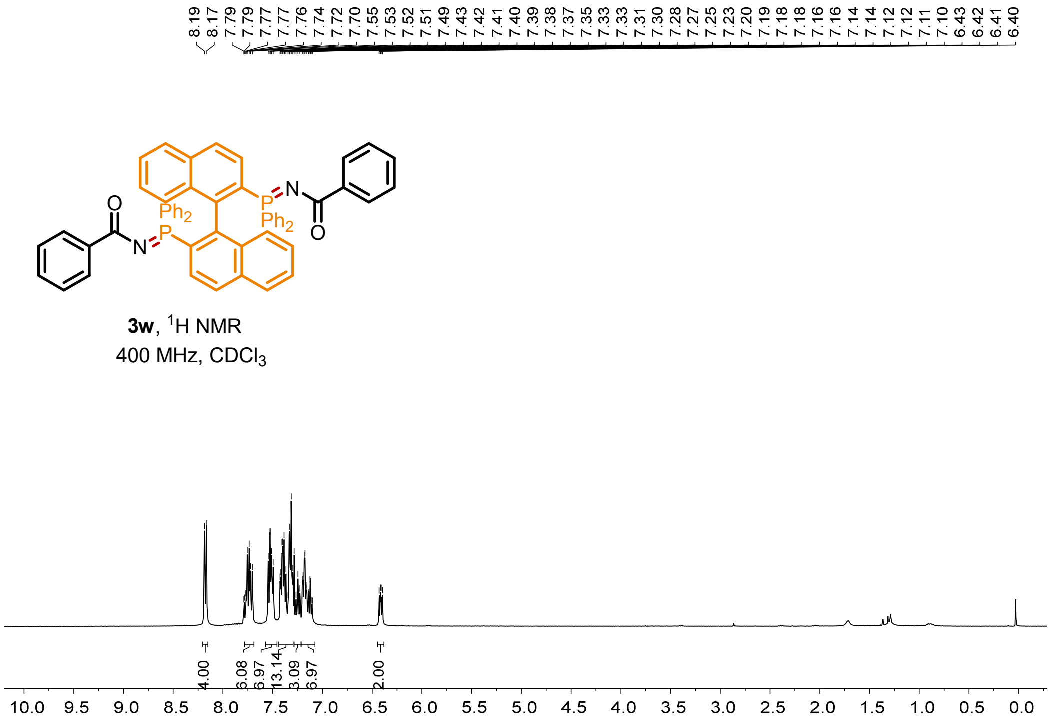
8.19 8.17 7.79 7.79 7.77 7.77 7.76 7.74 7.72 7.70 7.55 7.53 7.52 7.51 7.49 7.43 7.42 7.41 7.40 7.39 7.38 7.37 7.35 7.33 7.33 7.31 7.30 7.28 7.27 7.25 7.23 7.20 7.19 7.18 7.18 7.16 7.16 7.14 7.14 7.12 7.12 7.11 7.10 6.43 6.42 6.41 6.40
Ph2
Ph2
P
P
N
N
O
O
3w, 1H NMR
400 MHz, CDCl3
4.00 6.08 6.97 13.14 3.09 6.97 2.00
10.0 9.5 9.0 8.5 8.0 7.5 7.0 6.5 6.0 5.5 5.0 4.5 4.0 3.5 3.0 2.5 2.0 1.5 1.0 0.5 0.0

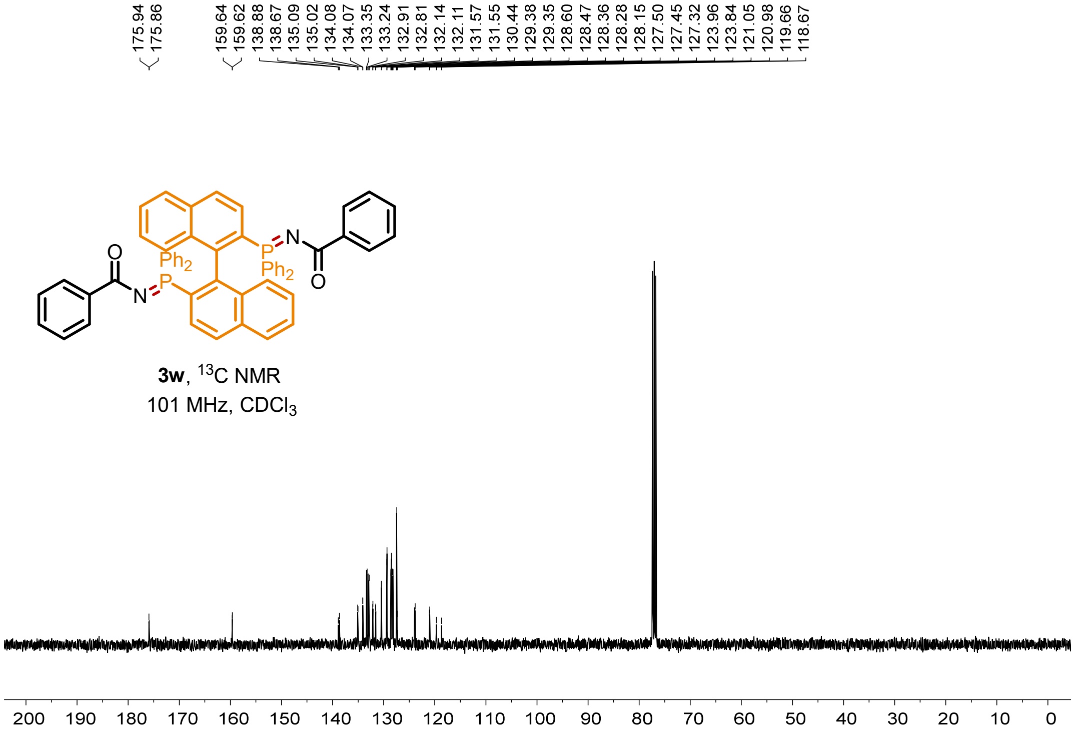
175.94 175.86 159.64 159.62 138.88 138.67 135.09 135.02 134.08 134.07 133.35 133.24 132.91 132.81 132.14 132.11 131.57 131.55 130.44 129.38 129.35 128.60 128.47 128.36 128.28 128.15 127.50 127.45 127.32 123.96 123.84 121.05 120.98 119.66 118.67
Ph2
Ph2
P
P
N
N
O
O
3w, 13C NMR
101 MHz, CDCl3
200 190 180 170 160 150 140 130 120 110 100 90 80 70 60 50 40 30 20 10 0

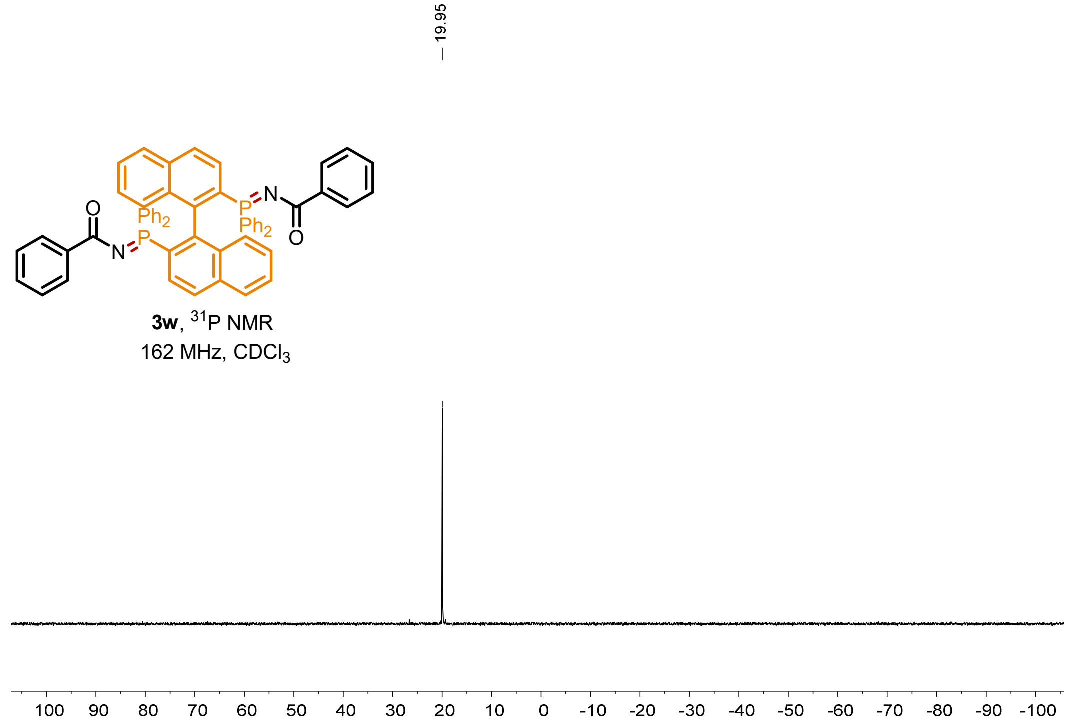
19.95
Ph2
Ph2
N
P
O
3w, 31P NMR
162 MHz, CDCl3
100 90 80 70 60 50 40 30 20 10 0 -10 -20 -30 -40 -50 -60 -70 -80 -90 -100

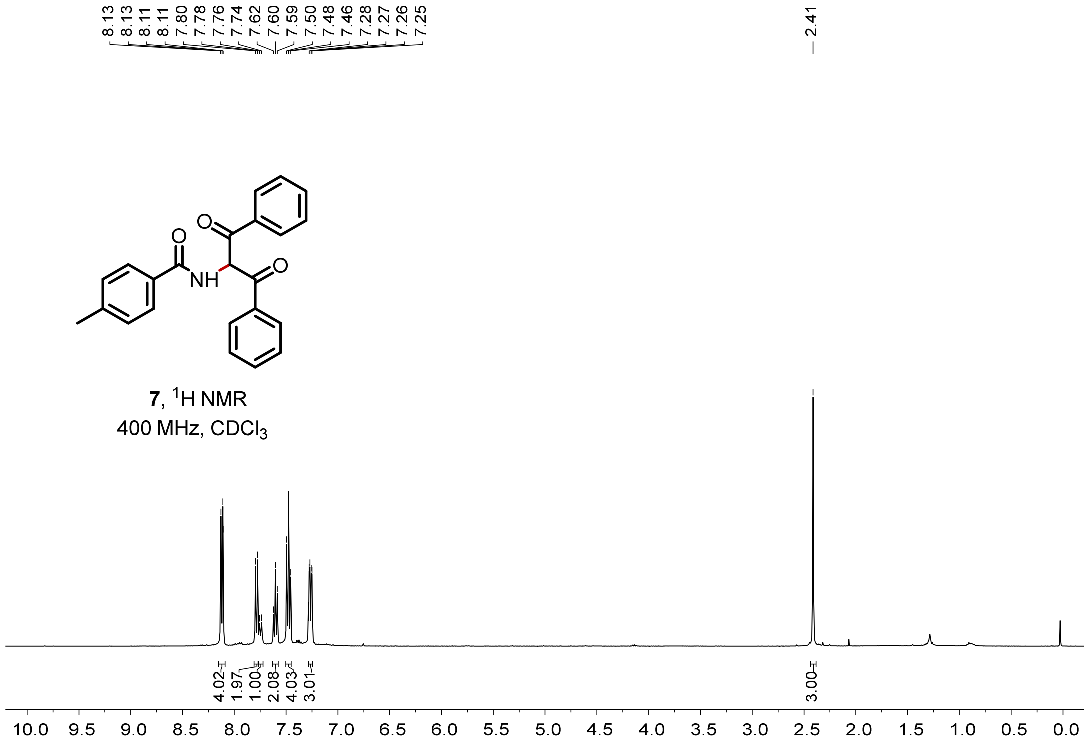
8.13 8.13 8.11 8.11 7.80 7.78 7.76 7.74 7.62 7.60 7.59 7.50 7.48 7.46 7.28 7.27 7.26 7.25
2.41
O
NH
7, 1H NMR
400 MHz, CDCl3
4.02 1.97 1.00 2.08 4.03 3.01
3.00
10.0 9.5 9.0 8.5 8.0 7.5 7.0 6.5 6.0 5.5 5.0 4.5 4.0 3.5 3.0 2.5 2.0 1.5 1.0 0.5 0.0

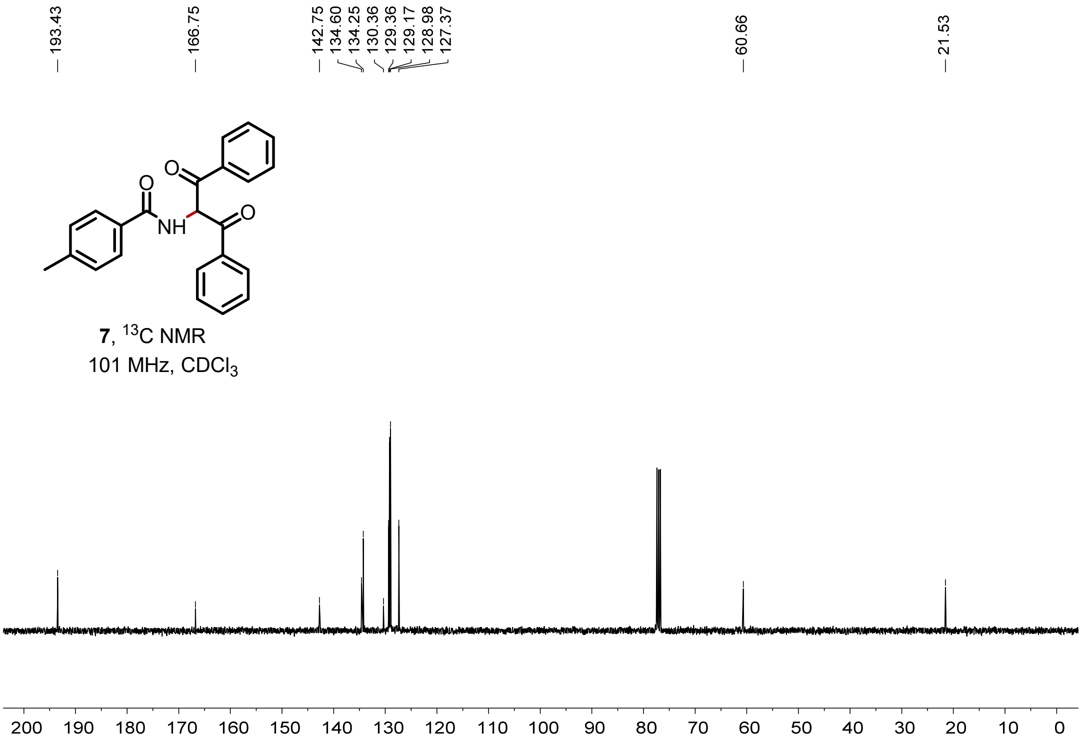
193.43
166.75
142.75
134.60
134.25
130.36
129.36
129.17
128.98
127.37
60.66
21.53
NH
O
O
O
7, 13C NMR
101 MHz, CDCl3
200 190 180 170 160 150 140 130 120 110 100 90 80 70 60 50 40 30 20 10 0

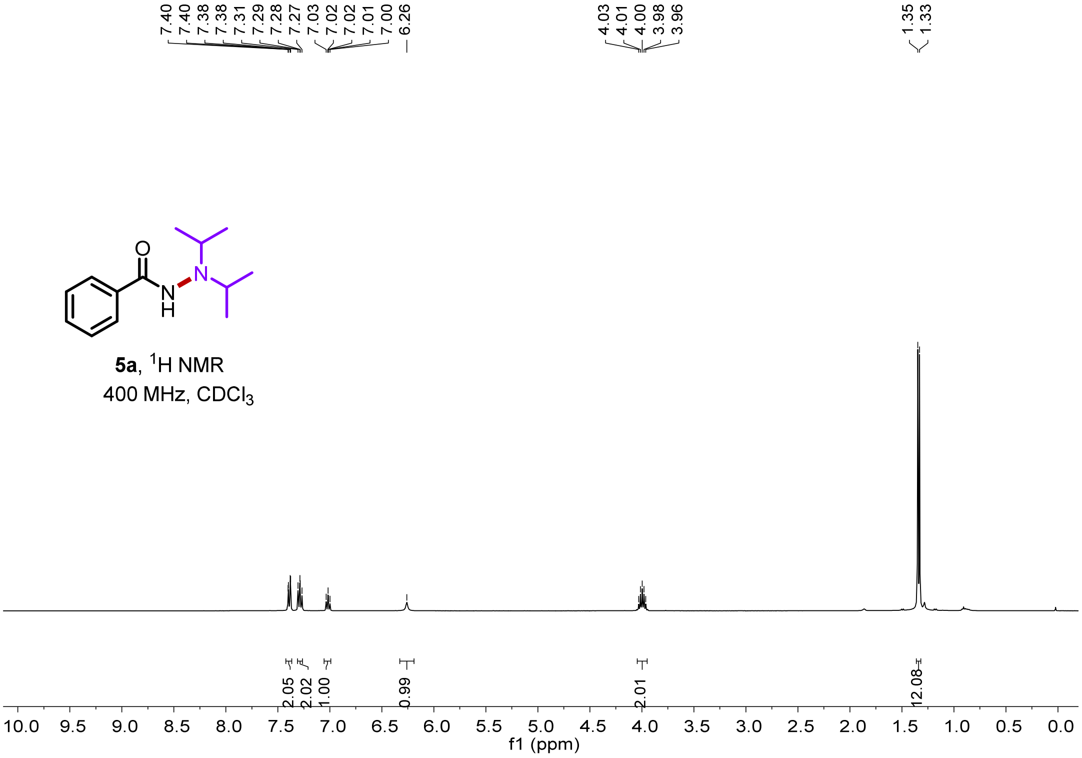
7.40
7.40
7.38
7.38
7.31
7.29
7.28
7.27
7.03
7.02
7.02
7.01
7.00
6.26
4.03
4.01
4.00
3.98
3.96
1.35
1.33
O
N
N
H
5a, 1H NMR
400 MHz, CDCl3
2.05
2.02
1.00
0.99
2.01
12.08
10.0 9.5 9.0 8.5 8.0 7.5 7.0 6.5 6.0 5.5 5.0 4.5 4.0 3.5 3.0 2.5 2.0 1.5 1.0 0.5 0.0
f1 (ppm)

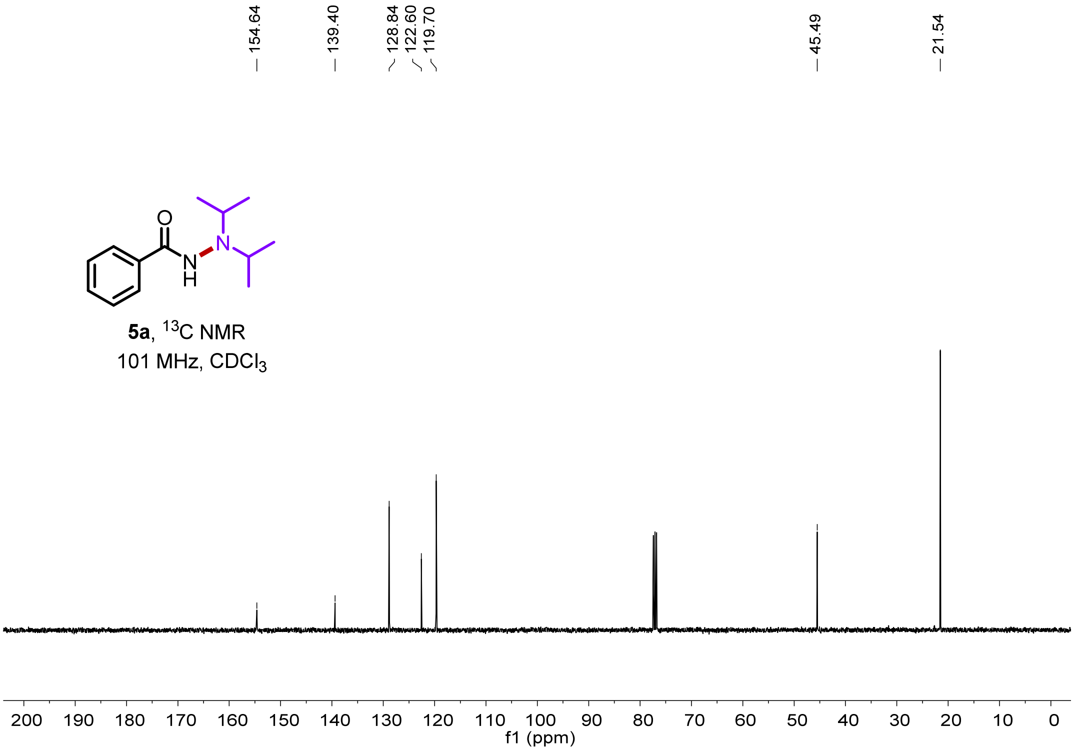
154.64
139.40
128.84
122.60
119.70
45.49
21.54
5a, 13C NMR
101 MHz, CDCl3
200 190 180 170 160 150 140 130 120 110 100 90 80 70 60 50 40 30 20 10 0
f1 (ppm)

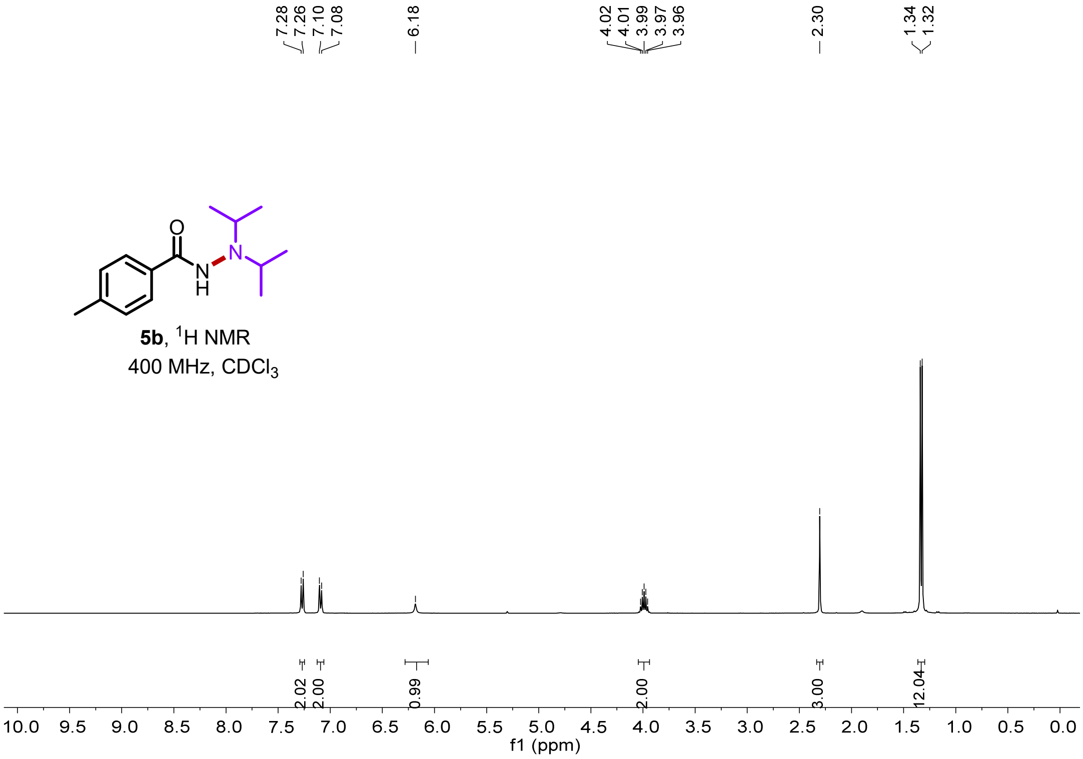
7.28
7.26
7.10
7.08
6.18
4.02
4.01
3.99
3.97
3.96
2.30
1.34
1.32
5b, 1H NMR
400 MHz, CDCl3
2.02
2.00
0.99
2.00
3.00
12.04
10.0 9.5 9.0 8.5 8.0 7.5 7.0 6.5 6.0 5.5 5.0 4.5 4.0 3.5 3.0 2.5 2.0 1.5 1.0 0.5 0.0
f1 (ppm)

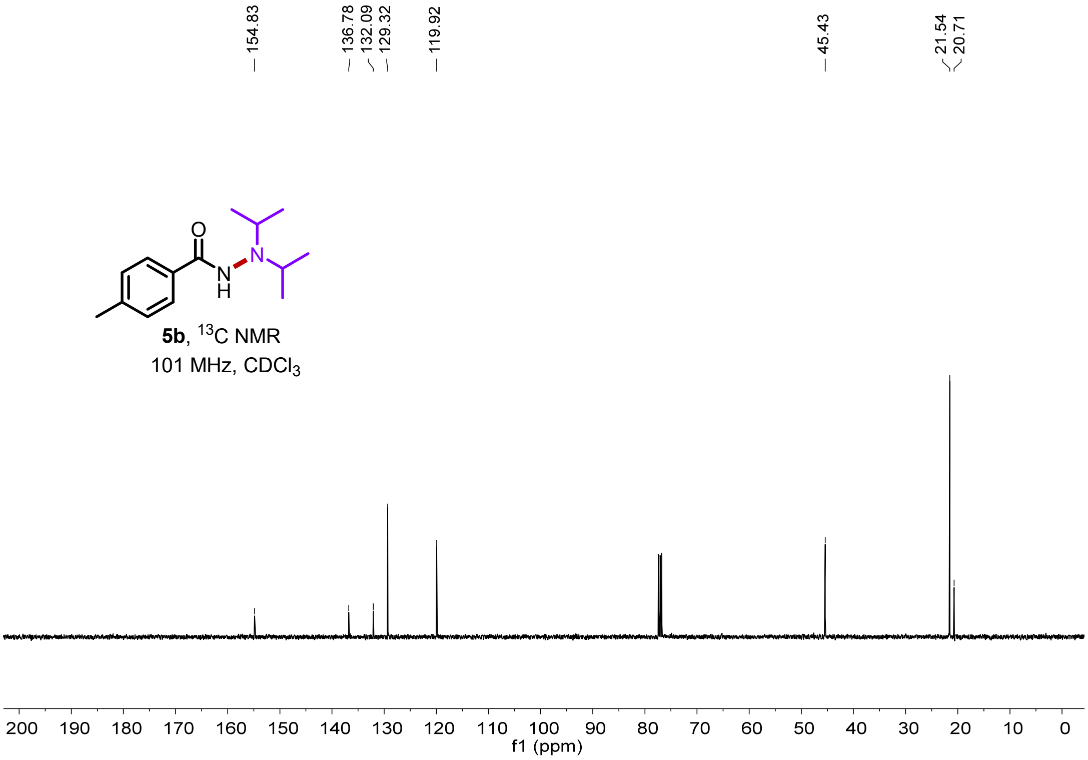
154.83
136.78
132.09
129.32
119.92
45.43
21.54
20.71
5b, ^{13}C NMR
101 MHz, $CDCl_3$
200 190 180 170 160 150 140 130 120 110 100 90 80 70 60 50 40 30 20 10 0
f1 (ppm)

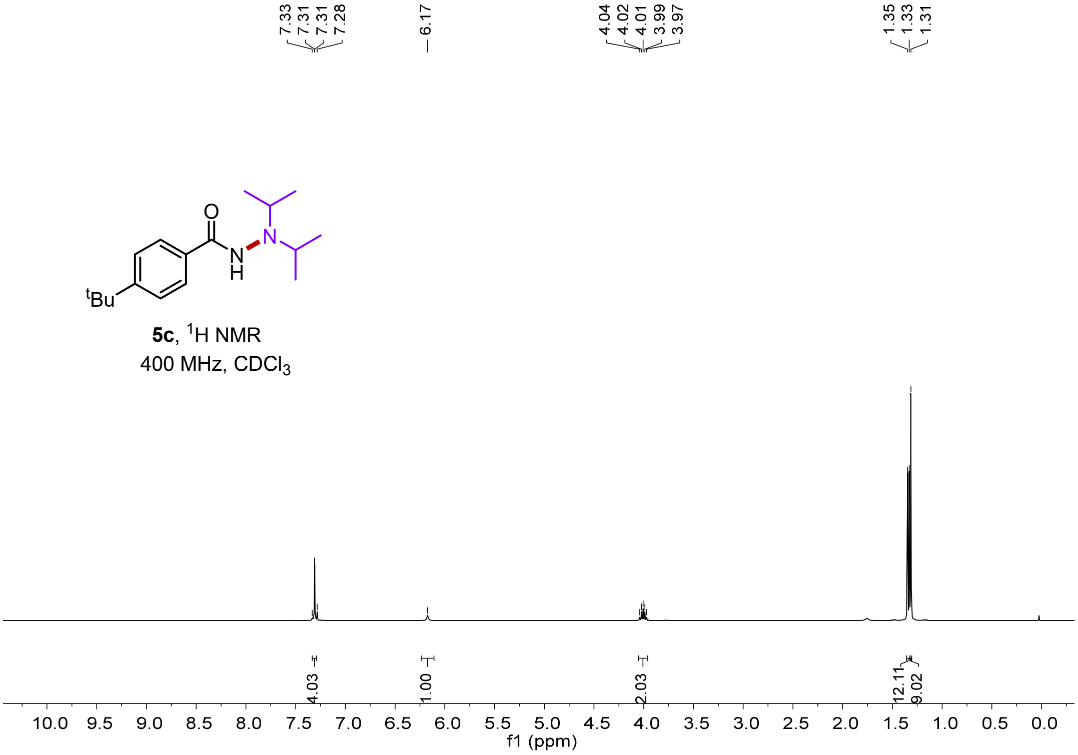
7.33
7.31
7.31
7.28
6.17
4.04
4.02
4.01
3.99
3.97
1.35
1.33
1.31
^{t}Bu
5c, ^{1}H NMR
400 MHz, $CDCl_3$
4.03
1.00
2.03
12.11
9.02
10.0 9.5 9.0 8.5 8.0 7.5 7.0 6.5 6.0 5.5 5.0 4.5 4.0 3.5 3.0 2.5 2.0 1.5 1.0 0.5 0.0
f1 (ppm)

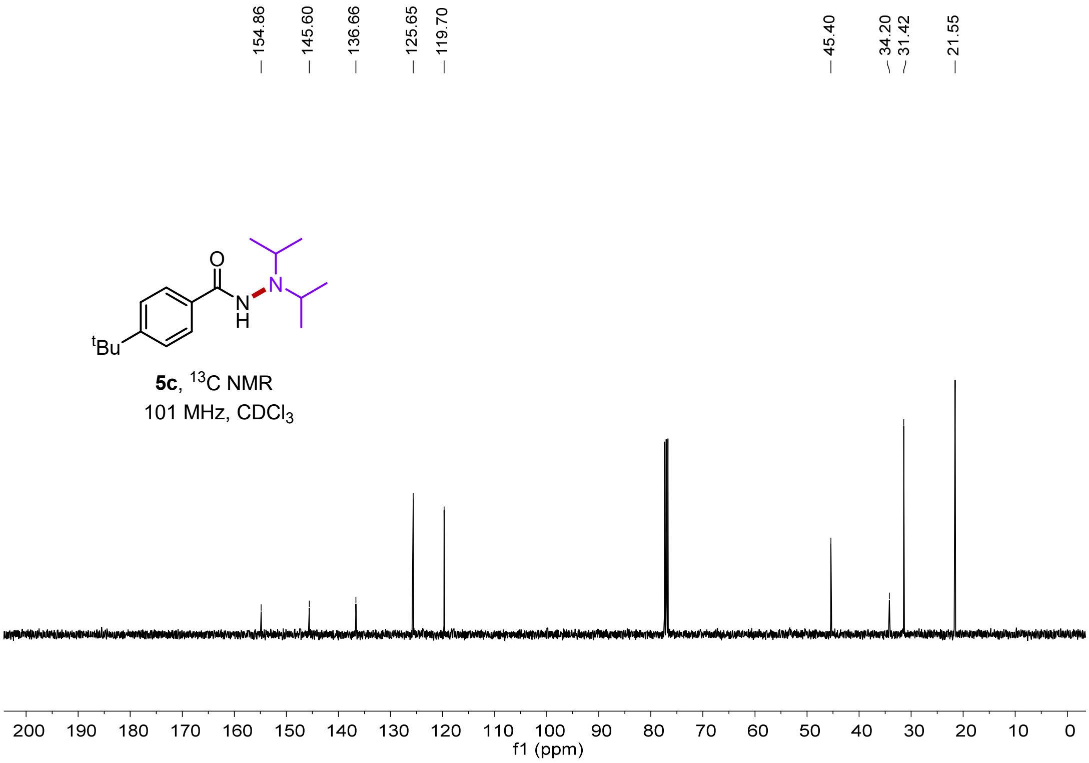
154.86
145.60
136.66
125.65
119.70
45.40
34.20
31.42
21.55
tBu
O
N
H
N
5c, 13C NMR
101 MHz, CDCl3
200 190 180 170 160 150 140 130 120 110 100 90 80 70 60 50 40 30 20 10 0
f1 (ppm)

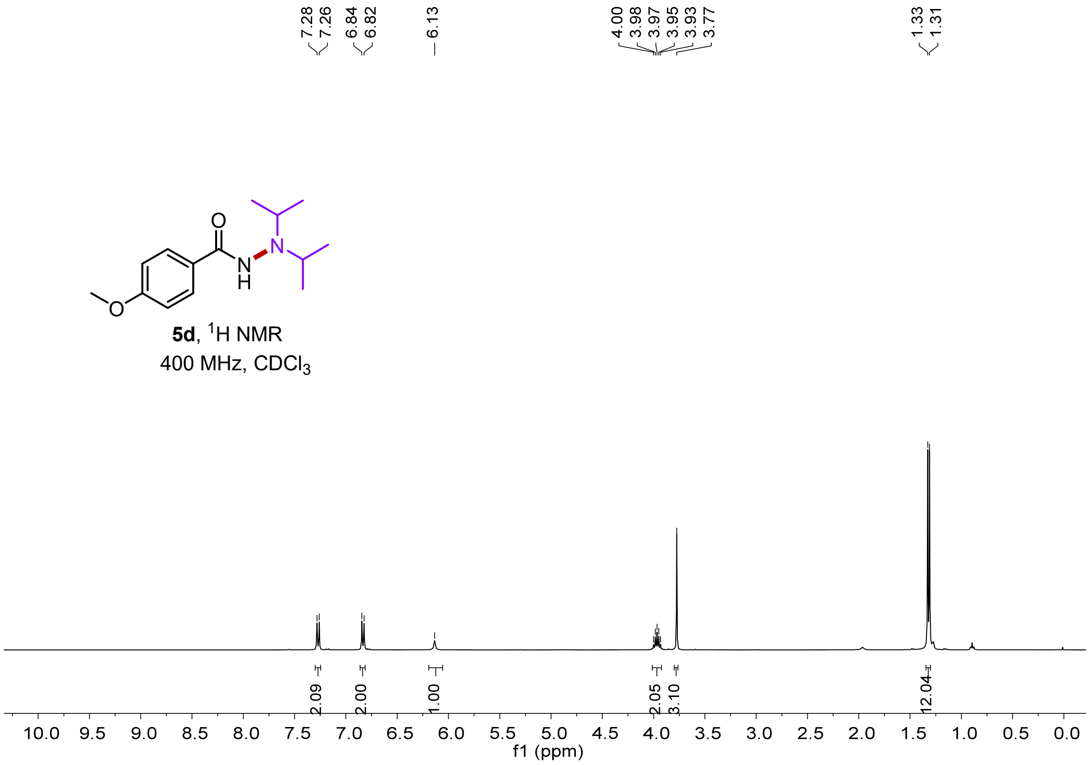
7.28
7.26
6.84
6.82
6.13
4.00
3.98
3.97
3.95
3.93
3.77
1.33
1.31
O
N
H
N
O
5d, 1H NMR
400 MHz, CDCl3
2.09
2.00
1.00
2.05
3.10
12.04
10.0 9.5 9.0 8.5 8.0 7.5 7.0 6.5 6.0 5.5 5.0 4.5 4.0 3.5 3.0 2.5 2.0 1.5 1.0 0.5 0.0
f1 (ppm)

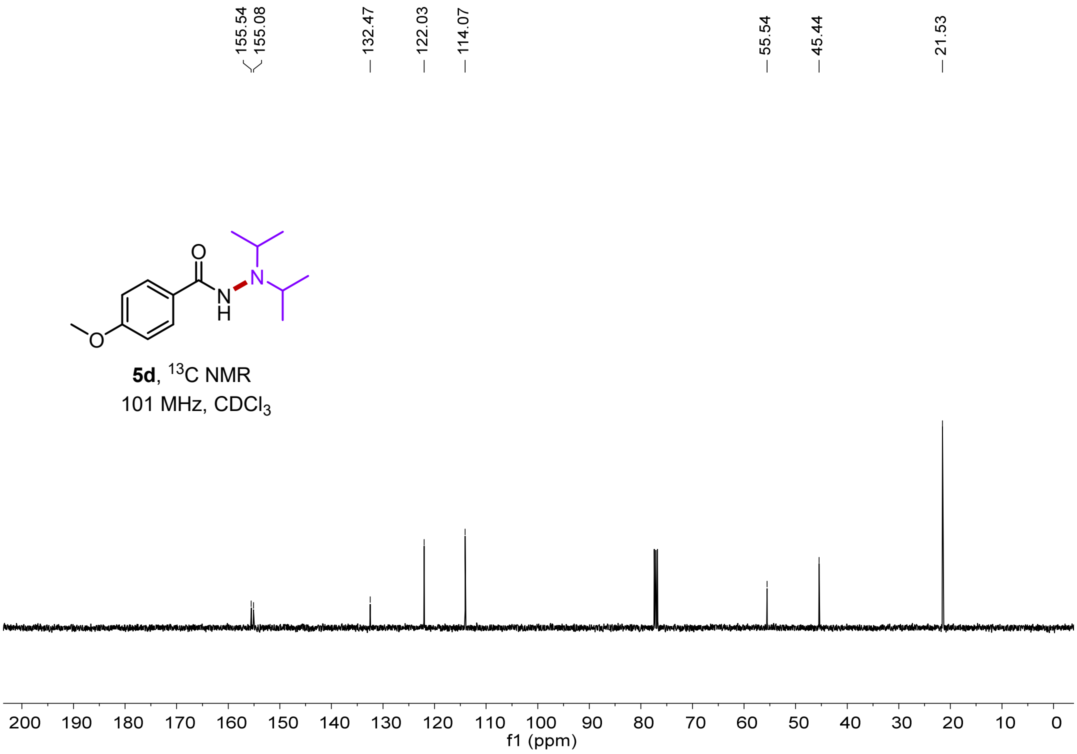
155.54
155.08
132.47
122.03
114.07
55.54
45.44
21.53
5d, 13C NMR
101 MHz, CDCl3
200 190 180 170 160 150 140 130 120 110 100 90 80 70 60 50 40 30 20 10 0
f1 (ppm)

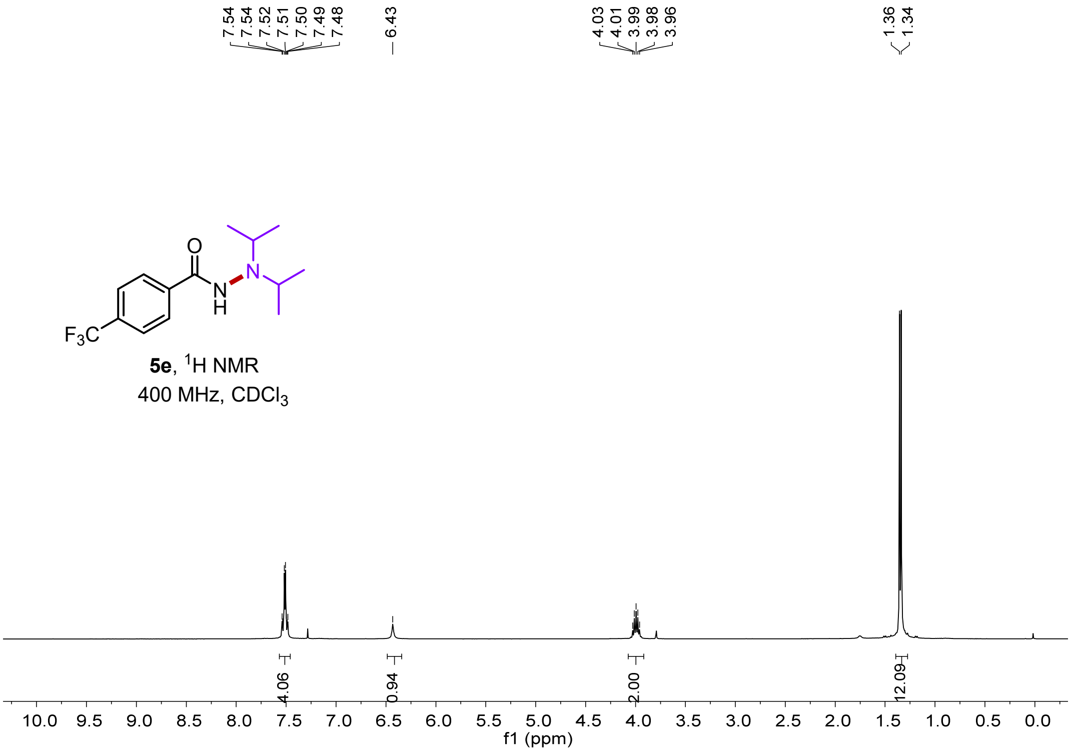
7.54
7.54
7.52
7.51
7.50
7.49
7.48
6.43
4.03
4.01
3.99
3.98
3.96
1.36
1.34
5e, 1H NMR
400 MHz, CDCl3
4.06
0.94
2.00
12.09
10.0 9.5 9.0 8.5 8.0 7.5 7.0 6.5 6.0 5.5 5.0 4.5 4.0 3.5 3.0 2.5 2.0 1.5 1.0 0.5 0.0
f1 (ppm)

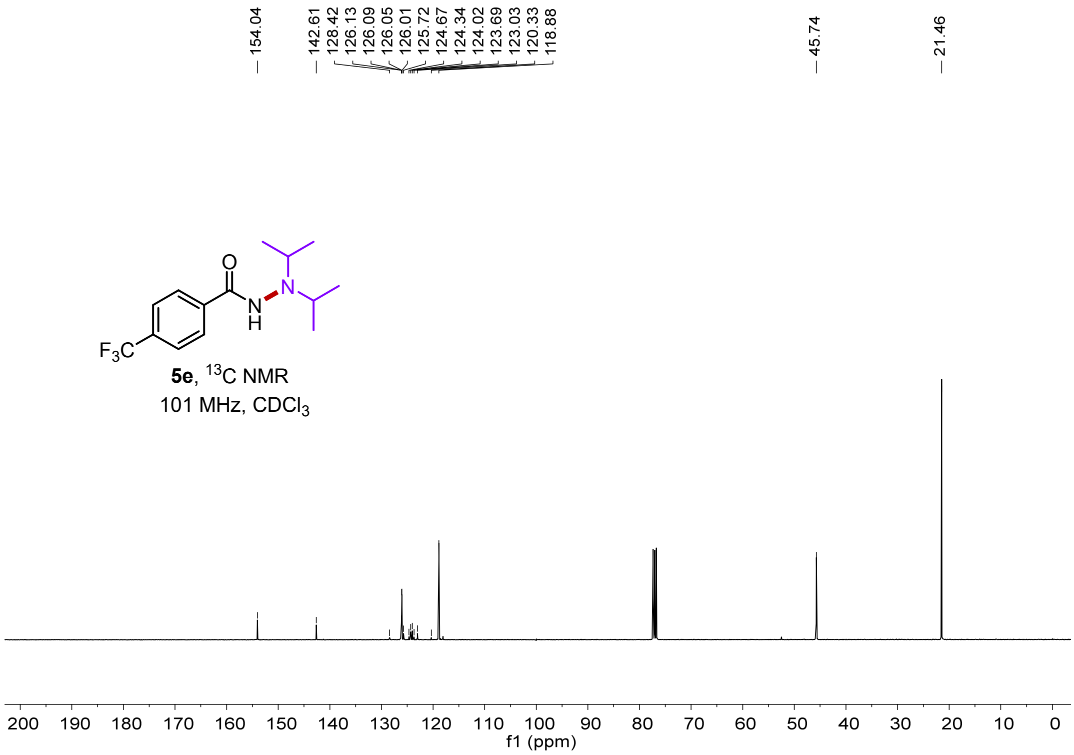
154.04
142.61
128.42
126.13
126.09
126.05
126.01
125.72
124.67
124.34
124.02
123.69
123.03
120.33
118.88
45.74
21.46
O
N
N
H
F3C
5e, 13C NMR
101 MHz, CDCl3
200 190 180 170 160 150 140 130 120 110 100 90 80 70 60 50 40 30 20 10 0
f1 (ppm)

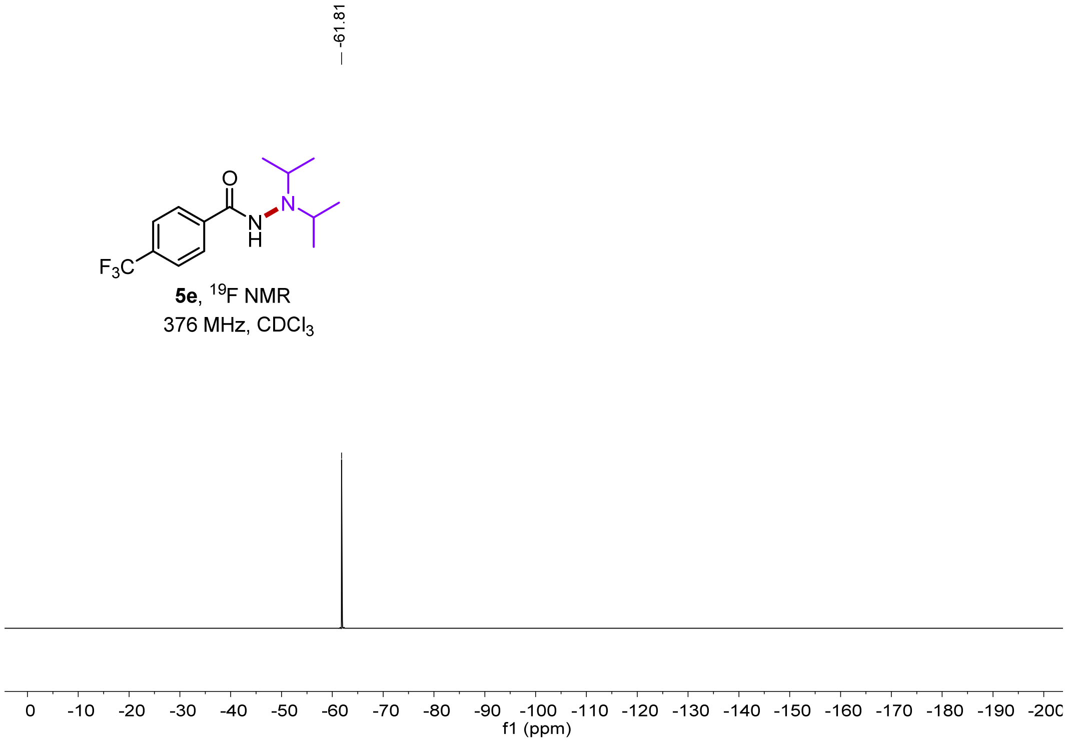
-61.81
O
N
N
H
F3C
5e, 19F NMR
376 MHz, CDCl3
0 -10 -20 -30 -40 -50 -60 -70 -80 -90 -100 -110 -120 -130 -140 -150 -160 -170 -180 -190 -200
f1 (ppm)

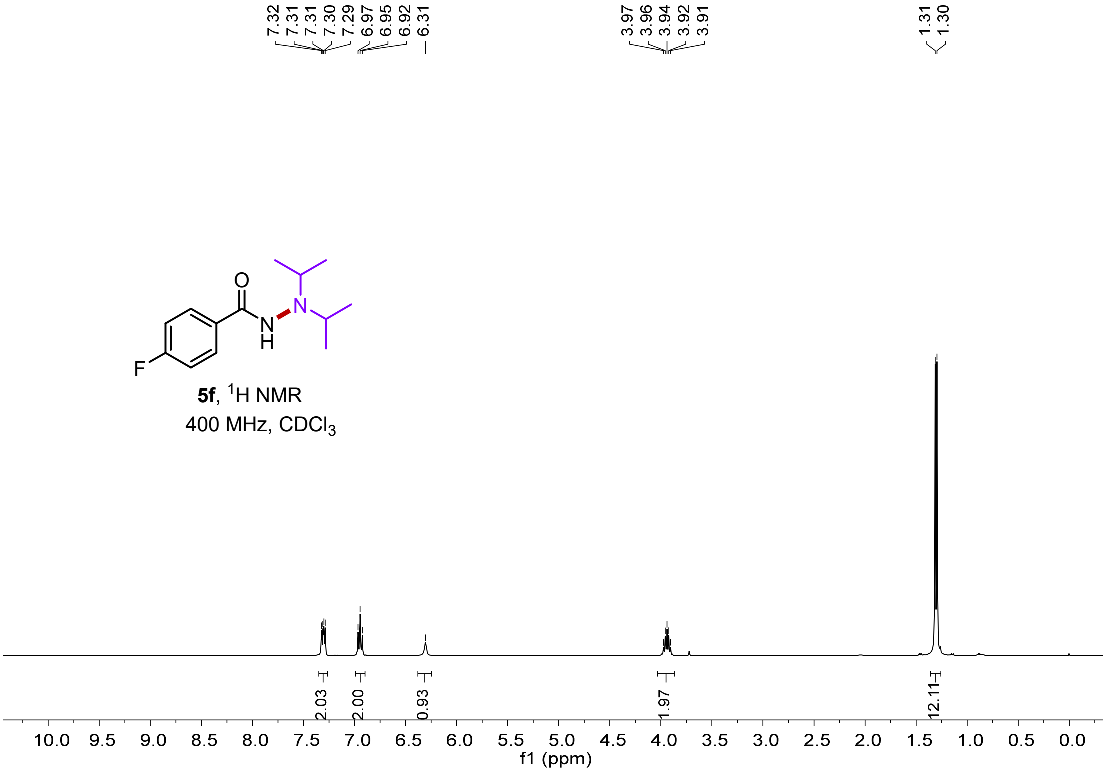
7.32
7.31
7.31
7.30
7.29
6.97
6.95
6.92
6.31
3.97
3.96
3.94
3.92
3.91
1.31
1.30
O
N
H
N
F
5f, ^{1}H NMR
400 MHz, $CDCl_3$
2.03
2.00
0.93
1.97
12.11
f1 (ppm)

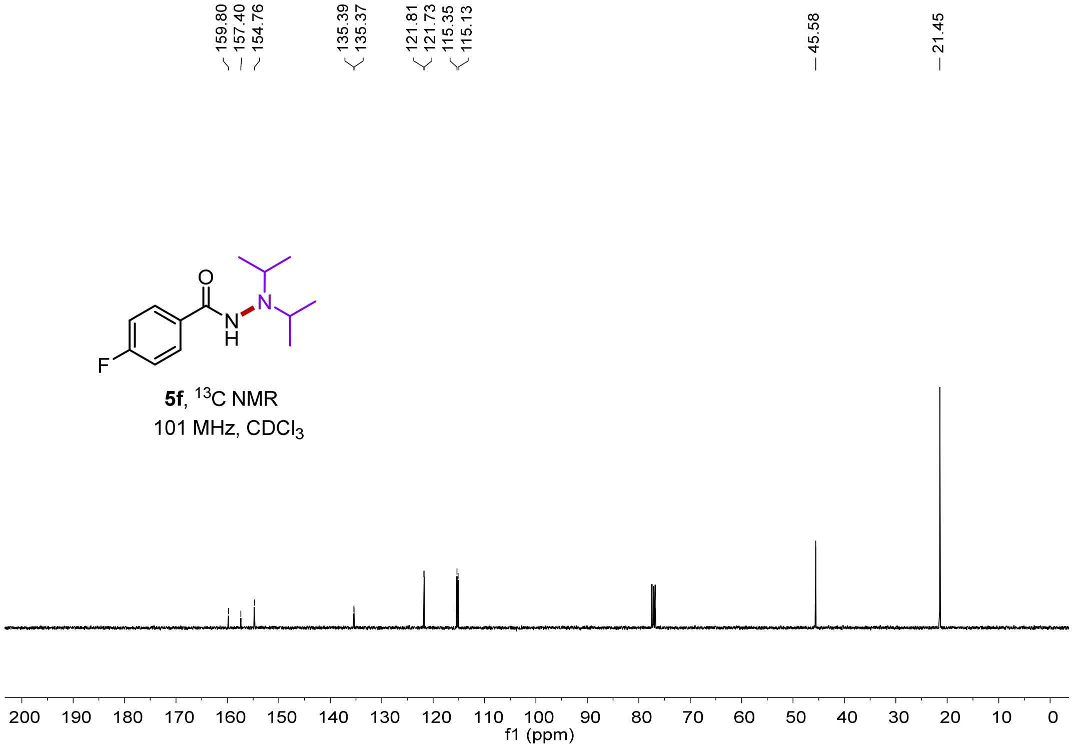
159.80
157.40
154.76
135.39
135.37
121.81
121.73
115.35
115.13
45.58
21.45
O
N
H
N
F
5f, ^{13}C NMR
101 MHz, $CDCl_3$
f1 (ppm)

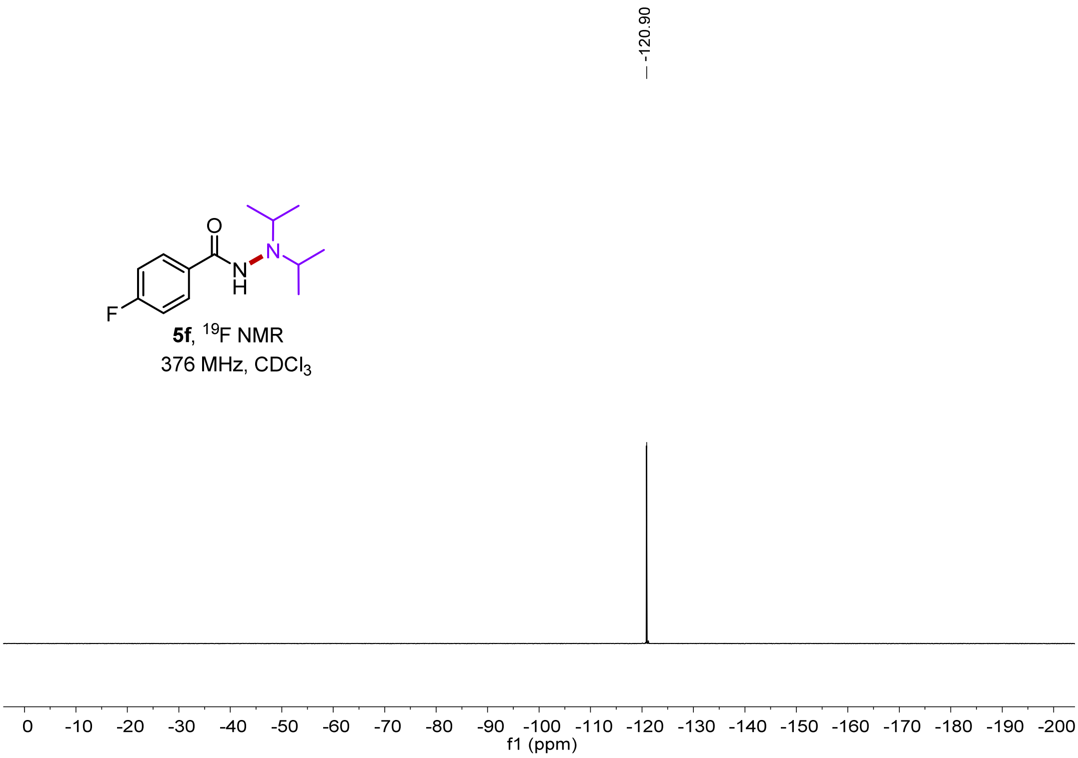
-120.90
O
N
N
H
F
5f, ^{19}F NMR
376 MHz, CDCl$_3$
0 -10 -20 -30 -40 -50 -60 -70 -80 -90 -100 -110 -120 -130 -140 -150 -160 -170 -180 -190 -200
f1 (ppm)

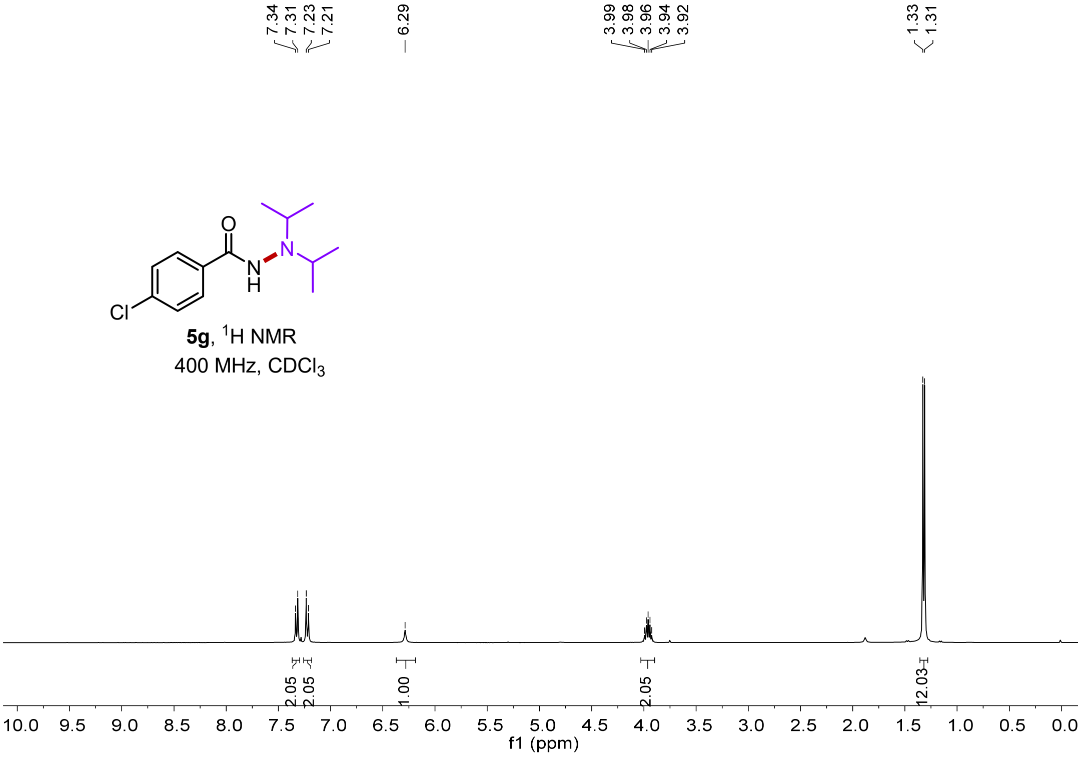
7.34
7.31
7.23
7.21
6.29
3.99
3.98
3.96
3.94
3.92
1.33
1.31
O
N
N
H
Cl
5g, ^{1}H NMR
400 MHz, CDCl$_3$
2.05
2.05
1.00
2.05
12.03
10.0 9.5 9.0 8.5 8.0 7.5 7.0 6.5 6.0 5.5 5.0 4.5 4.0 3.5 3.0 2.5 2.0 1.5 1.0 0.5 0.0
f1 (ppm)

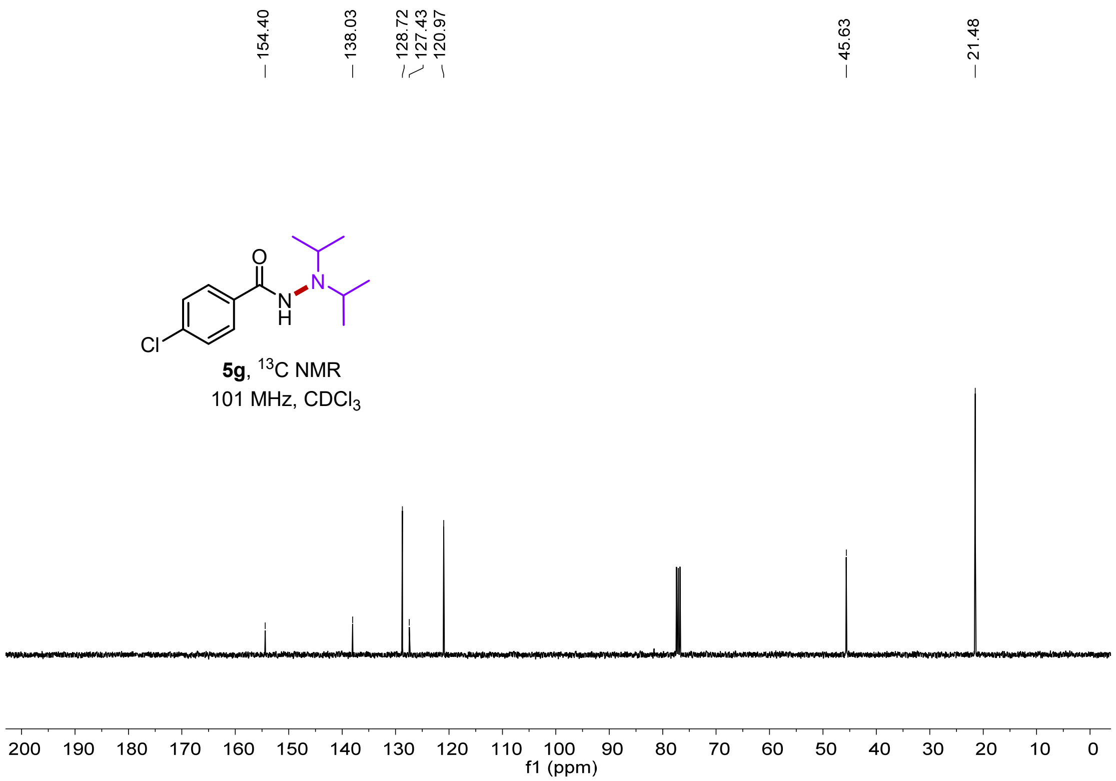
154.40
138.03
128.72
127.43
120.97
45.63
21.48
Cl
O
N
H
N
5g, 13C NMR
101 MHz, CDCl3
f1 (ppm)

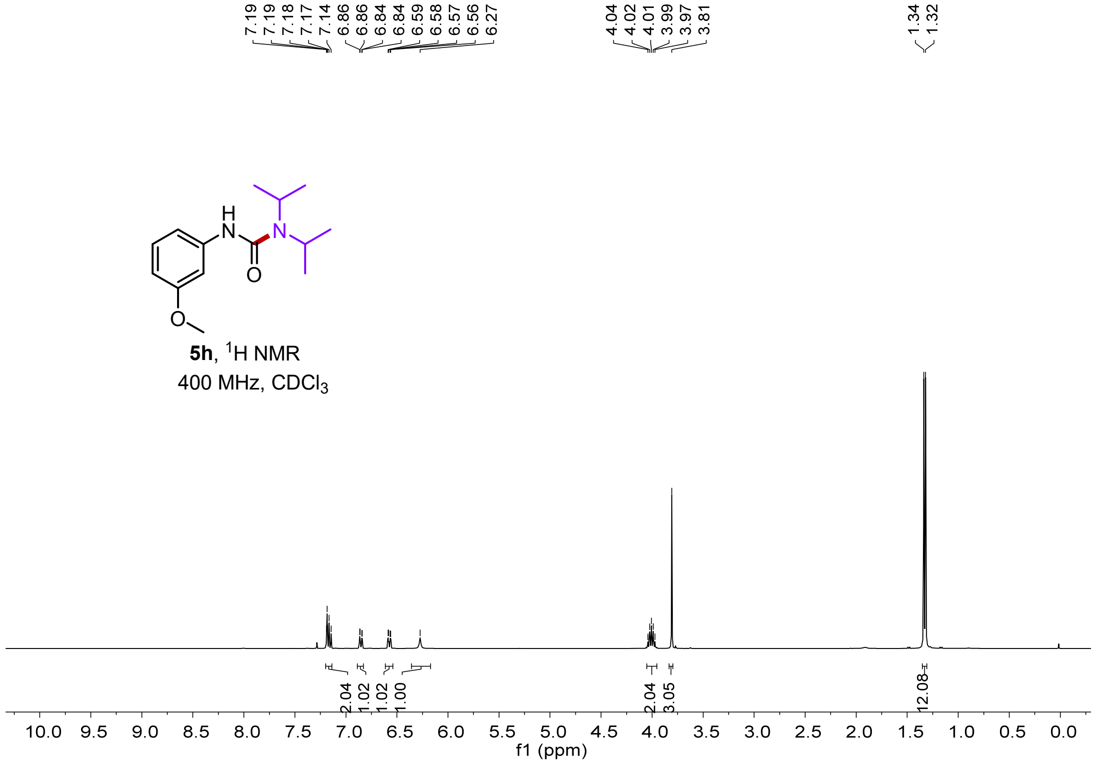
7.19
7.19
7.18
7.17
7.14
6.86
6.86
6.84
6.84
6.59
6.58
6.57
6.56
6.27
4.04
4.02
4.01
3.99
3.97
3.81
1.34
1.32
H
N
N
O
O
5h, 1H NMR
400 MHz, CDCl3
2.04
1.02
1.02
1.00
2.04
3.05
12.08
f1 (ppm)

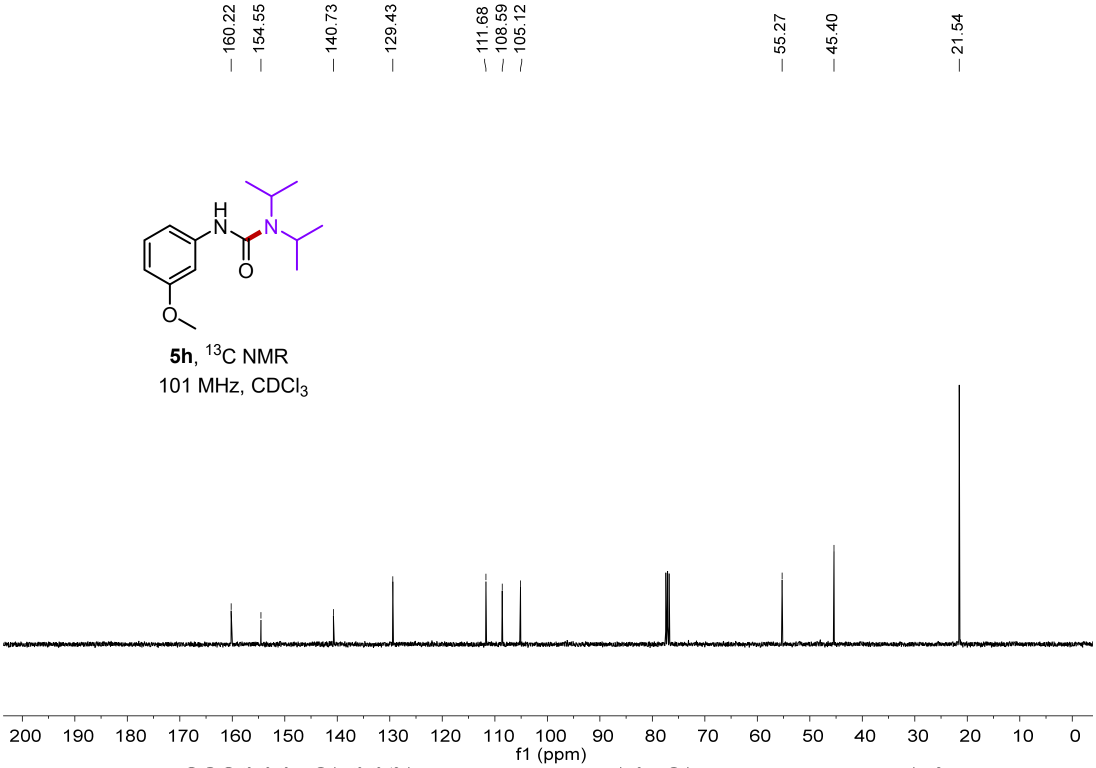
160.22
154.55
140.73
129.43
111.68
108.59
105.12
55.27
45.40
21.54
H
N
N
O
O
5h, 13C NMR
101 MHz, CDCl3
200 190 180 170 160 150 140 130 120 110 100 90 80 70 60 50 40 30 20 10 0
f1 (ppm)

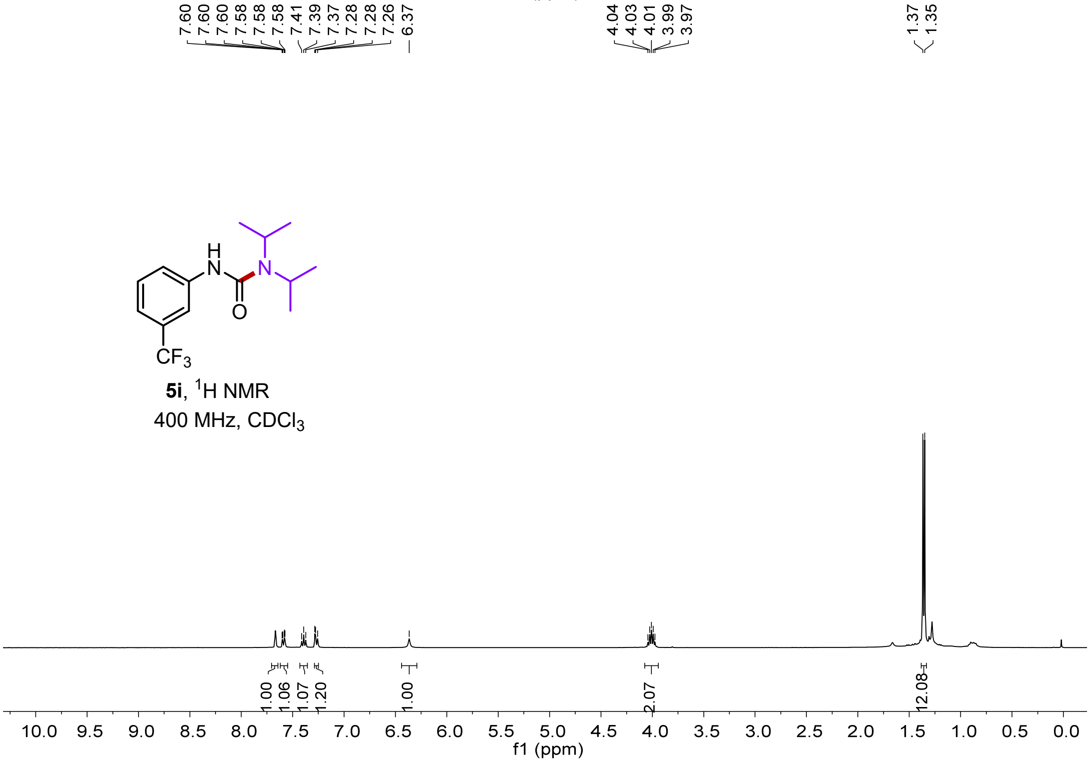
7.60
7.60
7.60
7.58
7.58
7.58
7.41
7.39
7.37
7.28
7.28
7.26
6.37
4.04
4.03
4.01
3.99
3.97
1.37
1.35
H
N
N
O
CF3
5i, 1H NMR
400 MHz, CDCl3
1.00
1.06
1.07
1.20
1.00
2.07
12.08
10.0 9.5 9.0 8.5 8.0 7.5 7.0 6.5 6.0 5.5 5.0 4.5 4.0 3.5 3.0 2.5 2.0 1.5 1.0 0.5 0.0
f1 (ppm)

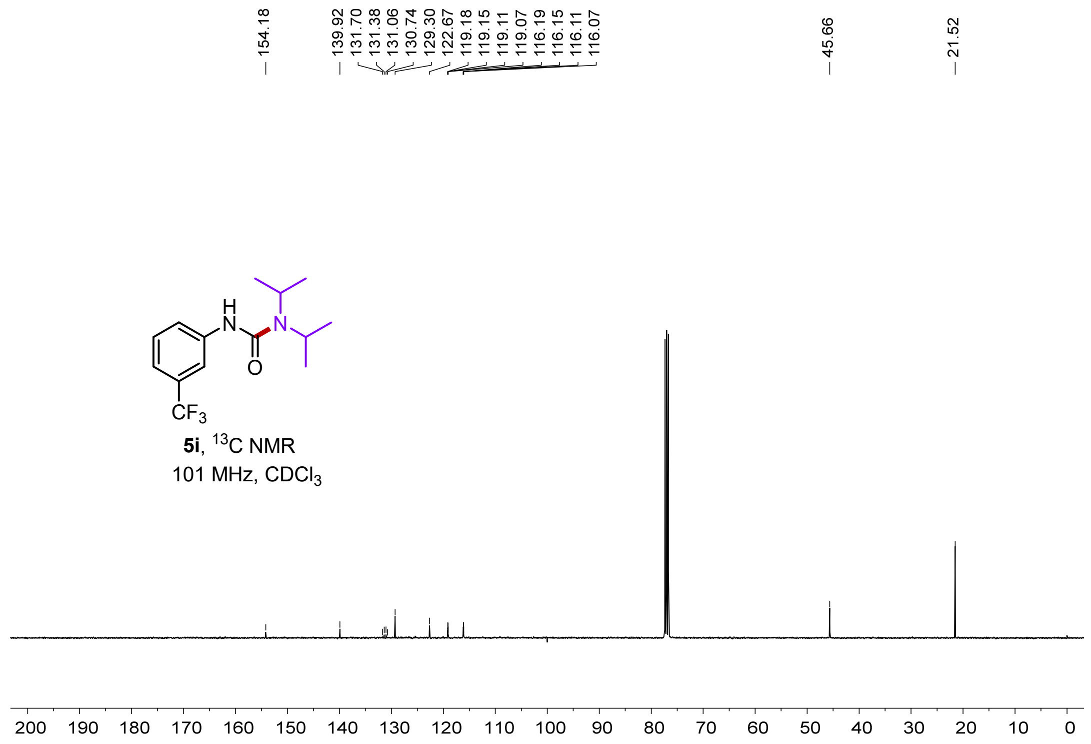
154.18
139.92
131.70
131.38
131.06
130.74
129.30
122.67
119.18
119.15
119.11
119.07
116.19
116.15
116.11
116.07
45.66
21.52
H
N
N
O
CF3
5i, 13C NMR
101 MHz, CDCl3
200 190 180 170 160 150 140 130 120 110 100 90 80 70 60 50 40 30 20 10 0

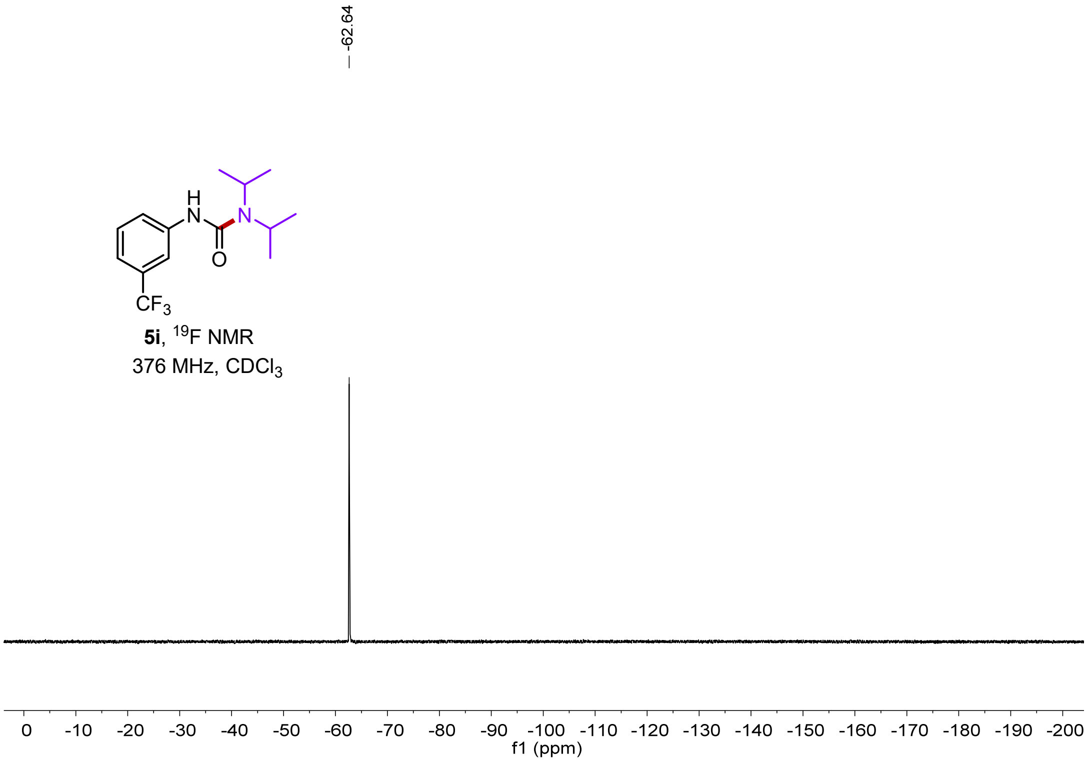
-62.64
H
N
N
O
CF3
5i, 19F NMR
376 MHz, CDCl3
0 -10 -20 -30 -40 -50 -60 -70 -80 -90 -100 -110 -120 -130 -140 -150 -160 -170 -180 -190 -200
f1 (ppm)

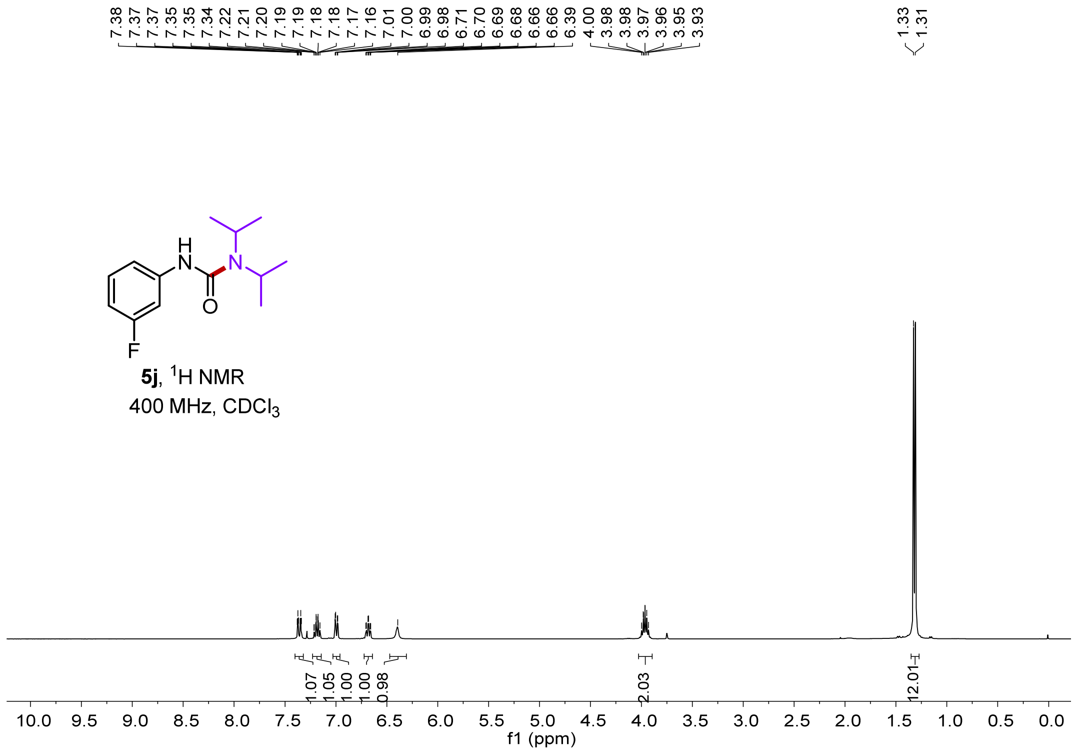
7.38
7.37
7.37
7.35
7.35
7.34
7.22
7.21
7.20
7.19
7.19
7.18
7.18
7.17
7.16
7.01
7.00
6.99
6.98
6.71
6.70
6.69
6.68
6.66
6.66
6.39
4.00
3.98
3.98
3.97
3.96
3.95
3.93
1.33
1.31
H
N
N
O
F
5j, 1H NMR
400 MHz, CDCl3
1.07
1.05
1.00
1.00
0.98
2.03
12.01
f1 (ppm)

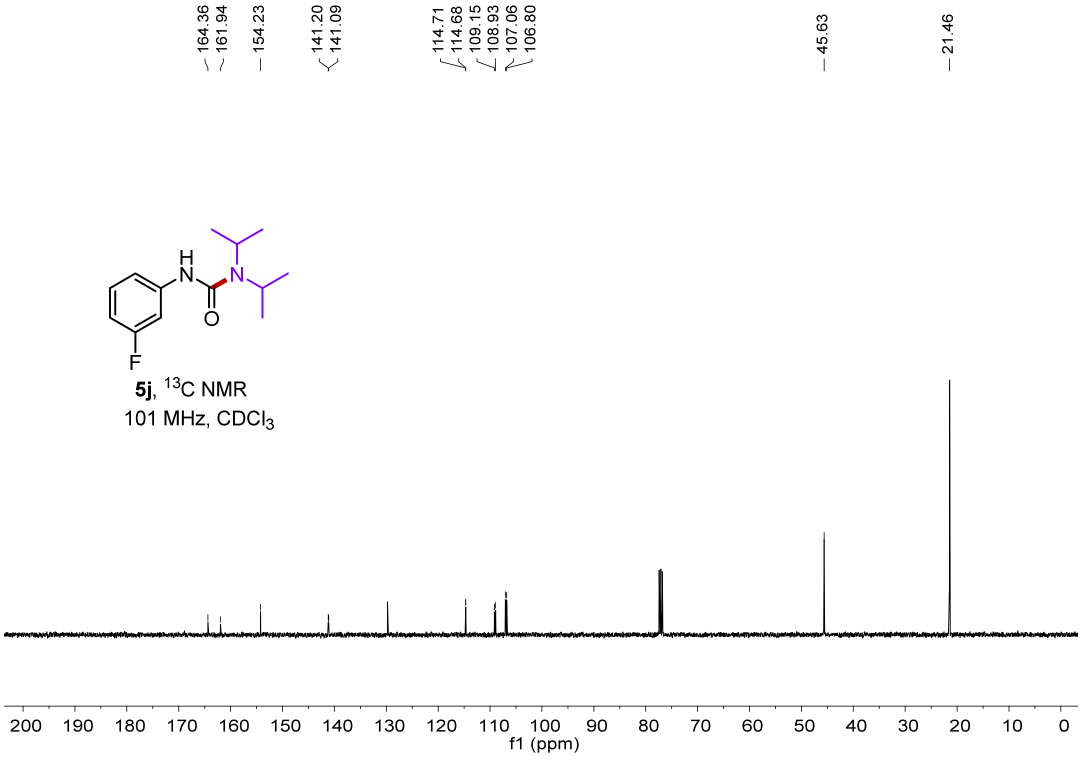
164.36
161.94
154.23
141.20
141.09
114.71
114.68
109.15
108.93
107.06
106.80
45.63
21.46
H
N
N
O
F
5j, 13C NMR
101 MHz, CDCl3
f1 (ppm)

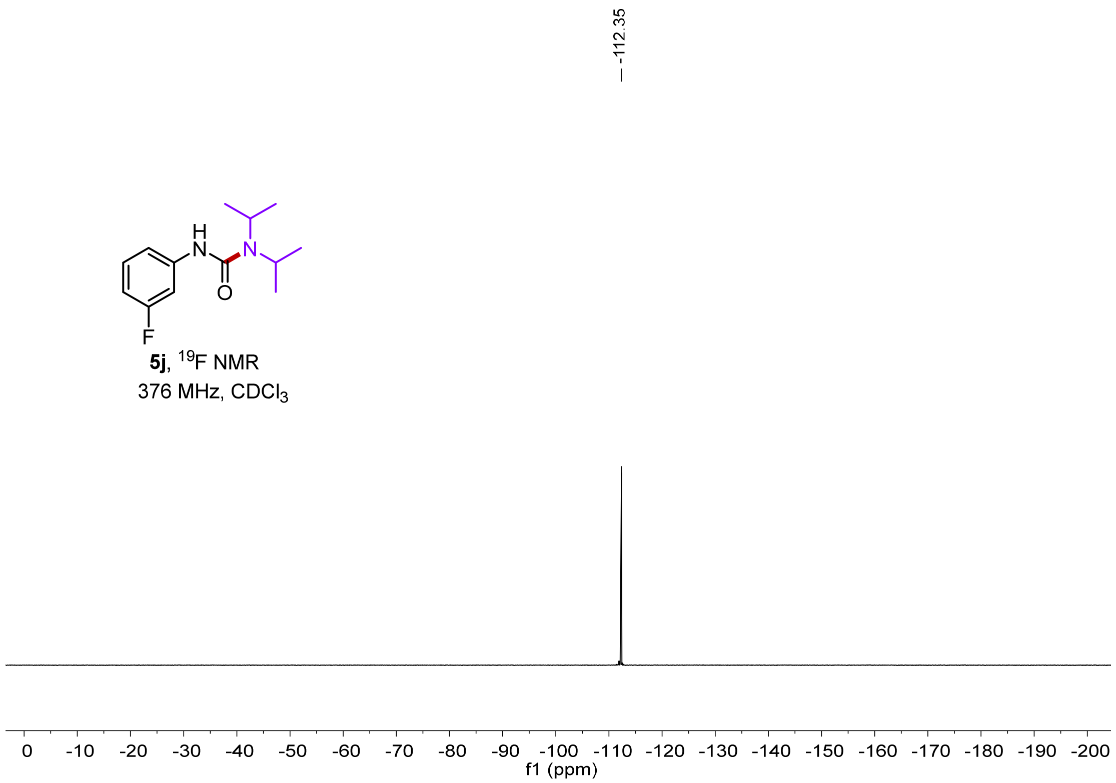
-112.35
5j, 19F NMR
376 MHz, CDCl3
0 -10 -20 -30 -40 -50 -60 -70 -80 -90 -100 -110 -120 -130 -140 -150 -160 -170 -180 -190 -200
f1 (ppm)

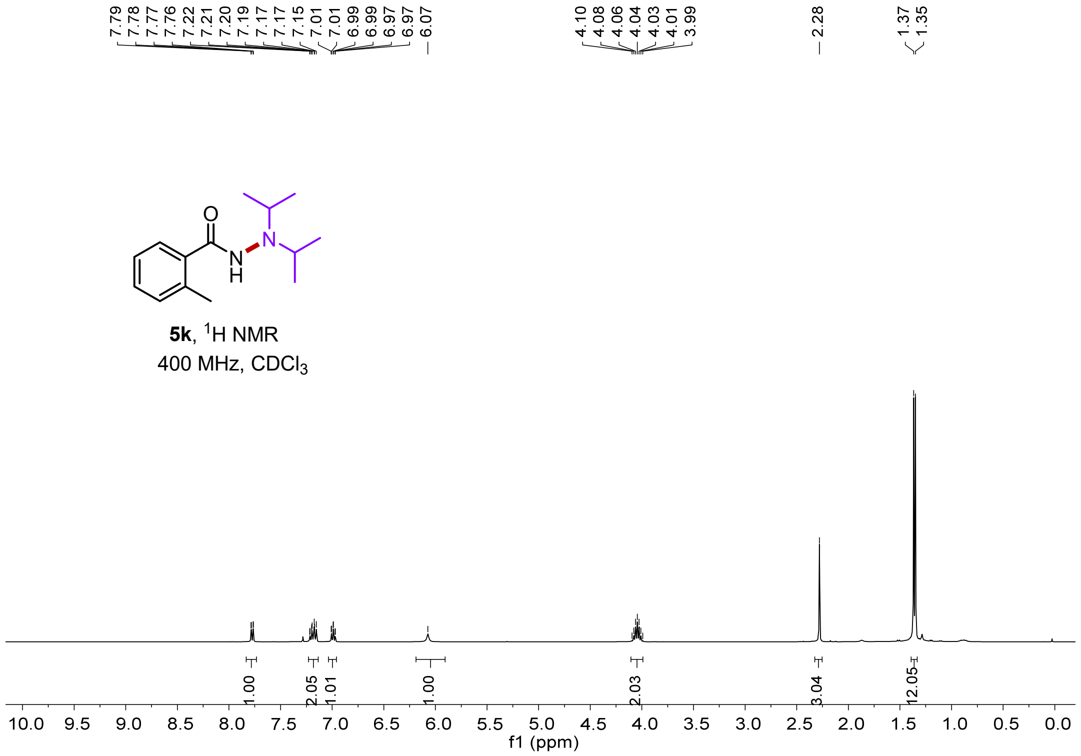
7.79 7.78 7.77 7.76 7.22 7.21 7.20 7.19 7.17 7.17 7.15 7.01 7.01 6.99 6.99 6.97 6.97 6.07
4.10 4.08 4.06 4.04 4.03 4.01 3.99
2.28
1.37 1.35
5k, 1H NMR
400 MHz, CDCl3
1.00 2.05 1.01 1.00 2.03 3.04 12.05
10.0 9.5 9.0 8.5 8.0 7.5 7.0 6.5 6.0 5.5 5.0 4.5 4.0 3.5 3.0 2.5 2.0 1.5 1.0 0.5 0.0
f1 (ppm)

— 154.82
— 137.59
130.26
127.64
126.72
123.23
122.28
— 45.44
— 21.50
— 18.28

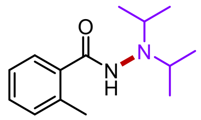

5k, ^{13}C NMR
101 MHz, CDCl$_3$

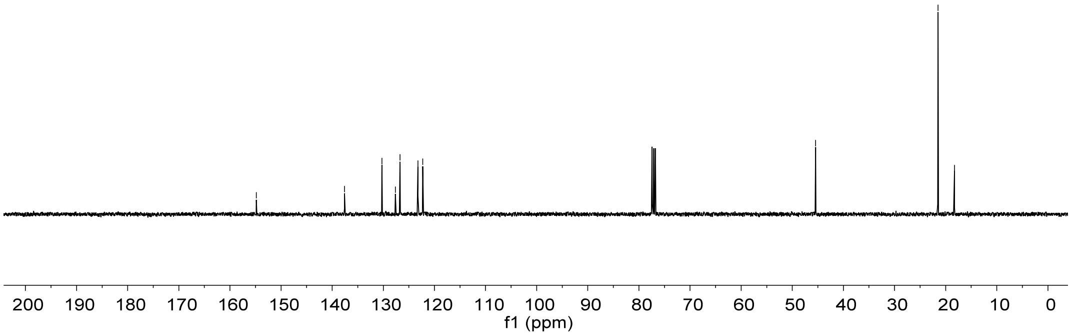

8.22 8.21 8.20 8.19 8.18 8.17 7.12 7.12 7.10 7.09 7.08 7.08 7.07 7.06 7.06 7.06 7.04 7.03 6.96 6.96 6.95 6.94 6.94 6.94 6.93 6.92 6.92 6.92 6.91 6.90 6.58 4.12 4.11 4.09 4.07 4.05 4.04 4.02
1.36
1.34

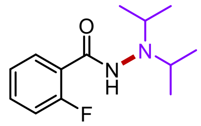

5l, ^{1}H NMR
400 MHz, CDCl$_3$

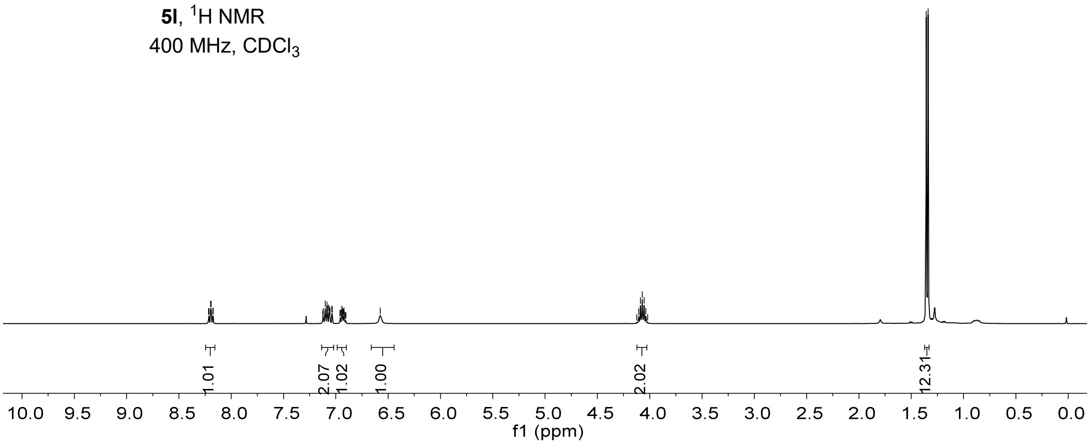

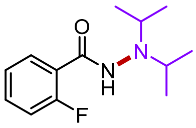

5l, ^{13}C NMR
101 MHz, $CDCl_3$

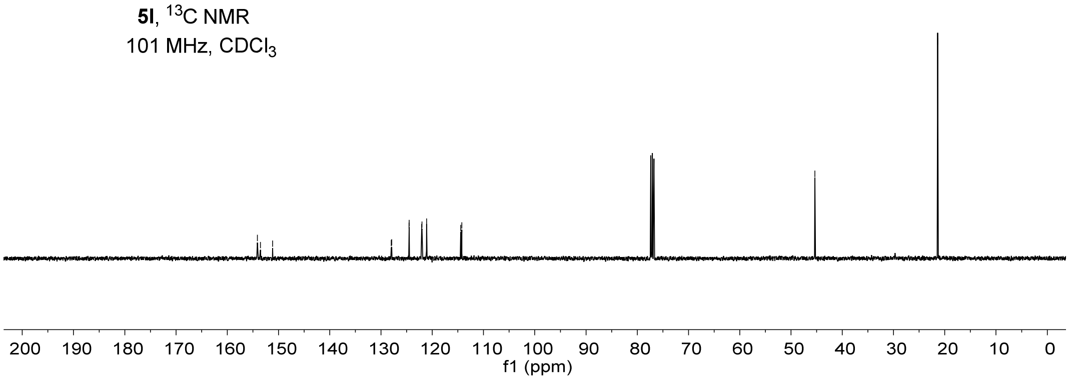

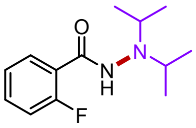

5l, ^{19}F NMR
376 MHz, $CDCl_3$

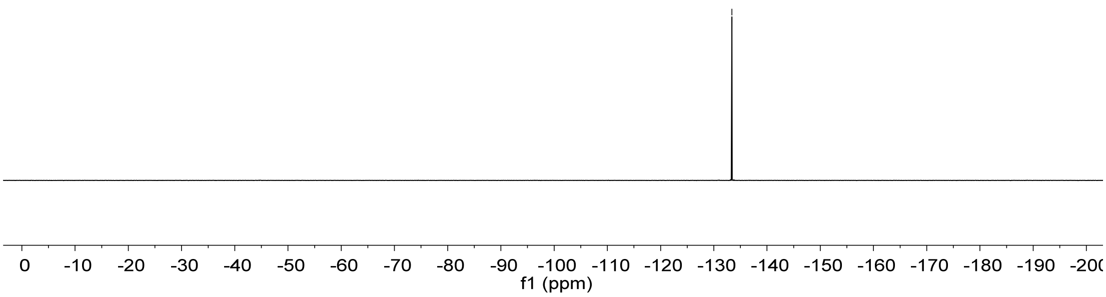

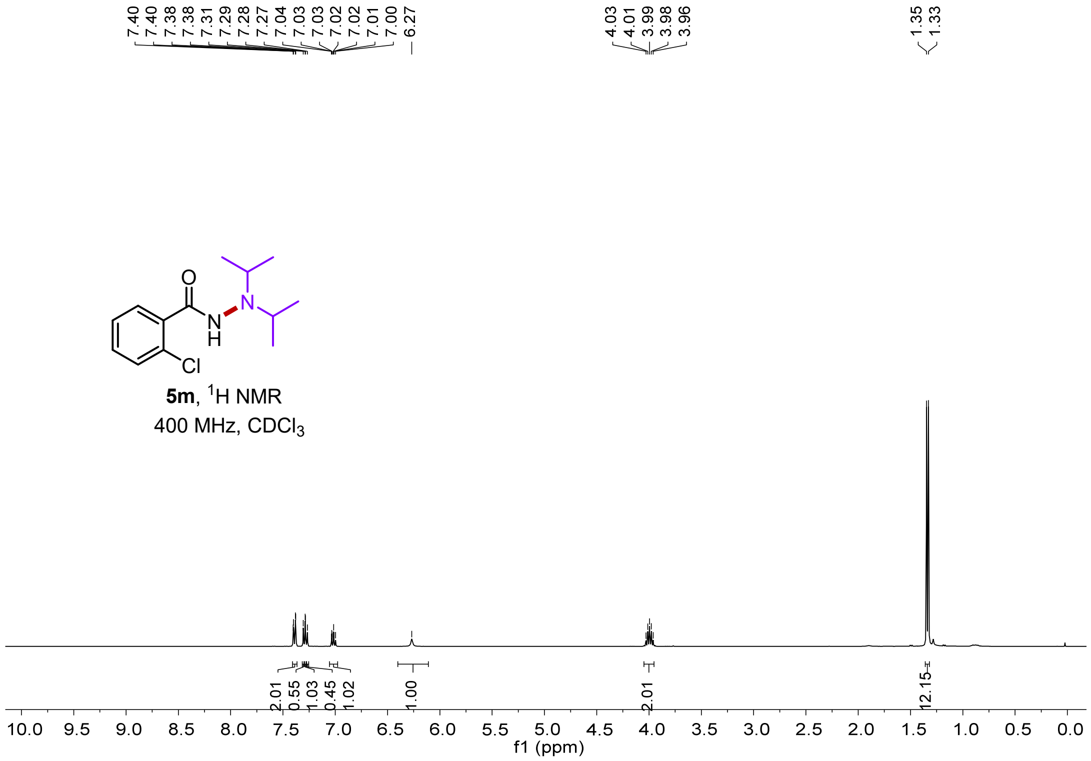
7.40
7.40
7.38
7.38
7.31
7.29
7.28
7.27
7.04
7.03
7.03
7.02
7.02
7.01
7.00
6.27
4.03
4.01
3.99
3.98
3.96
1.35
1.33
O
N
H
N
Cl
5m, ^{1}H NMR
400 MHz, $CDCl_3$
2.01
0.55
1.03
0.45
1.02
1.00
2.01
12.15
10.0 9.5 9.0 8.5 8.0 7.5 7.0 6.5 6.0 5.5 5.0 4.5 4.0 3.5 3.0 2.5 2.0 1.5 1.0 0.5 0.0
f1 (ppm)

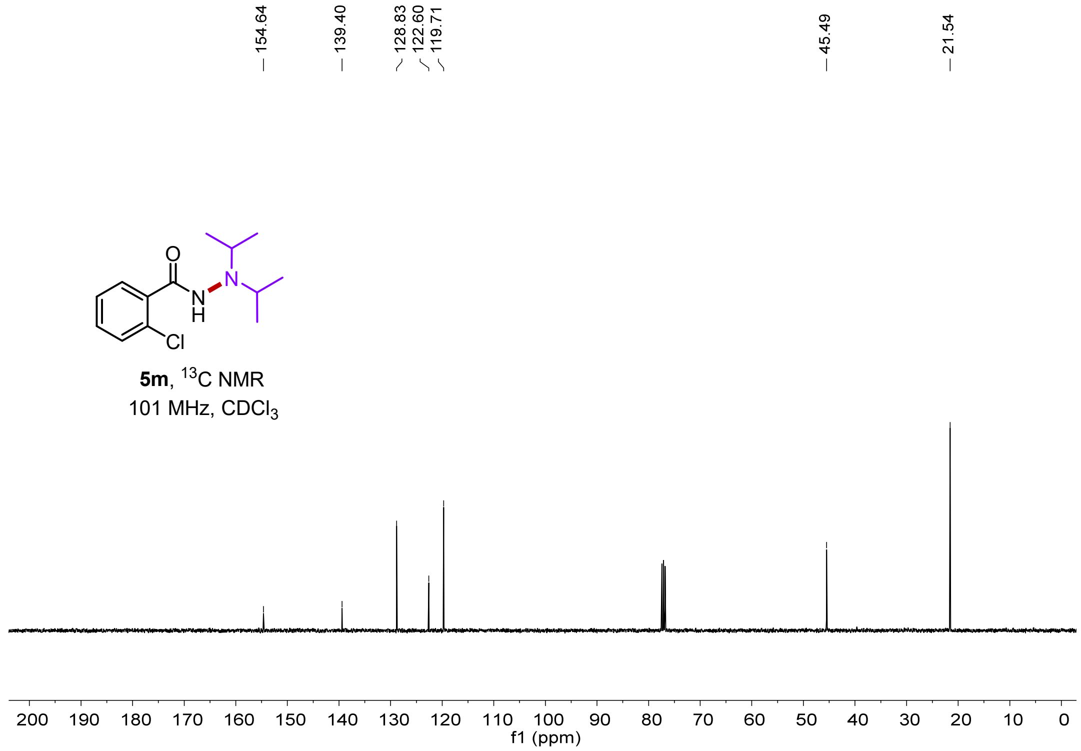
154.64
139.40
128.83
122.60
119.71
45.49
21.54
O
N
H
N
Cl
5m, ^{13}C NMR
101 MHz, $CDCl_3$
200 190 180 170 160 150 140 130 120 110 100 90 80 70 60 50 40 30 20 10 0
f1 (ppm)

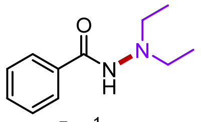

5n, ^{1}H NMR
400 MHz, $CDCl_3$

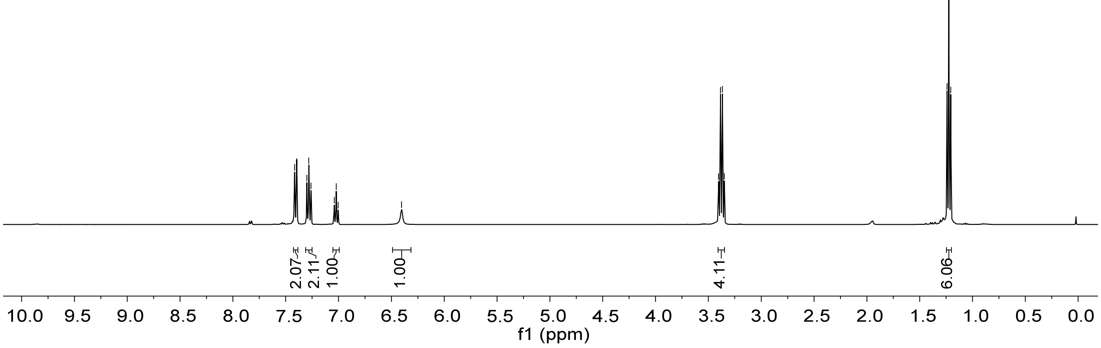

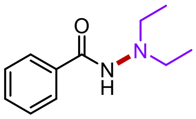

5n, ^{13}C NMR
101 MHz, $CDCl_3$

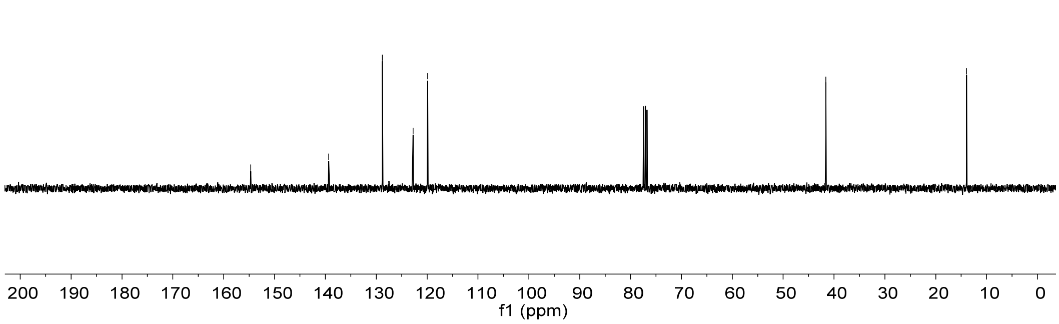

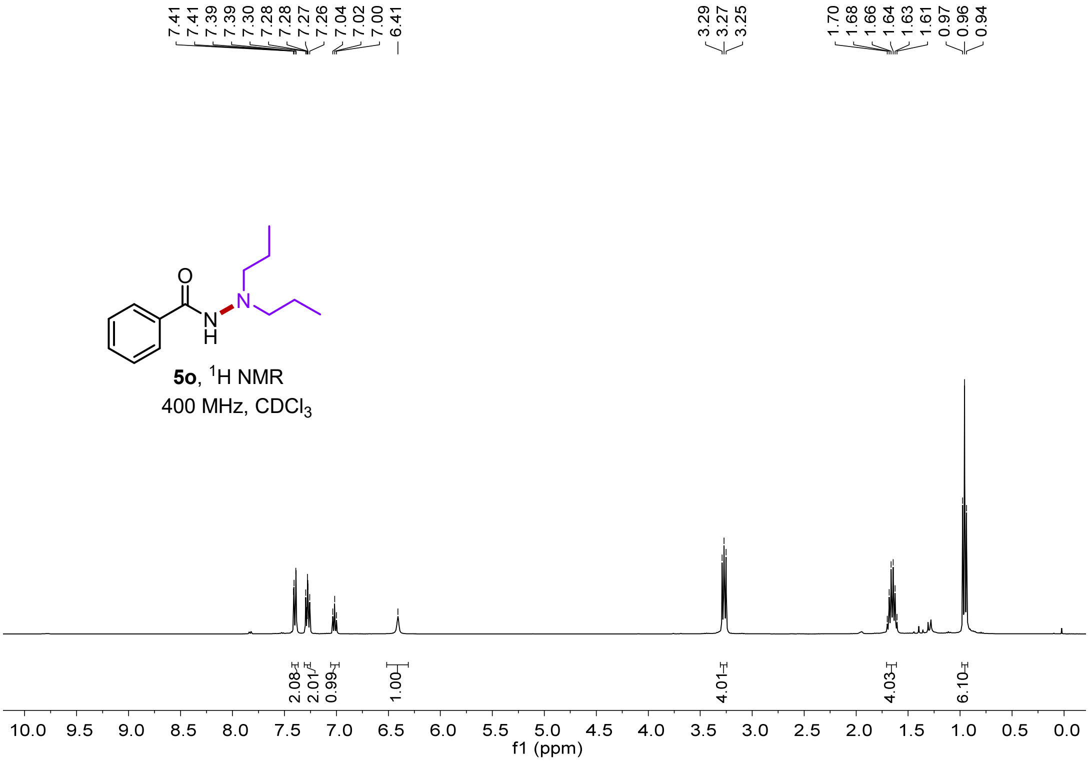
7.41
7.41
7.39
7.39
7.30
7.28
7.28
7.27
7.26
7.04
7.02
7.00
6.41
3.29
3.27
3.25
1.70
1.68
1.66
1.64
1.63
1.61
0.97
0.96
0.94
O
N
H
N
5o, 1H NMR
400 MHz, $CDCl_3$
2.08
2.01
0.99
1.00
4.01
4.03
6.10
10.0 9.5 9.0 8.5 8.0 7.5 7.0 6.5 6.0 5.5 5.0 4.5 4.0 3.5 3.0 2.5 2.0 1.5 1.0 0.5 0.0
f1 (ppm)

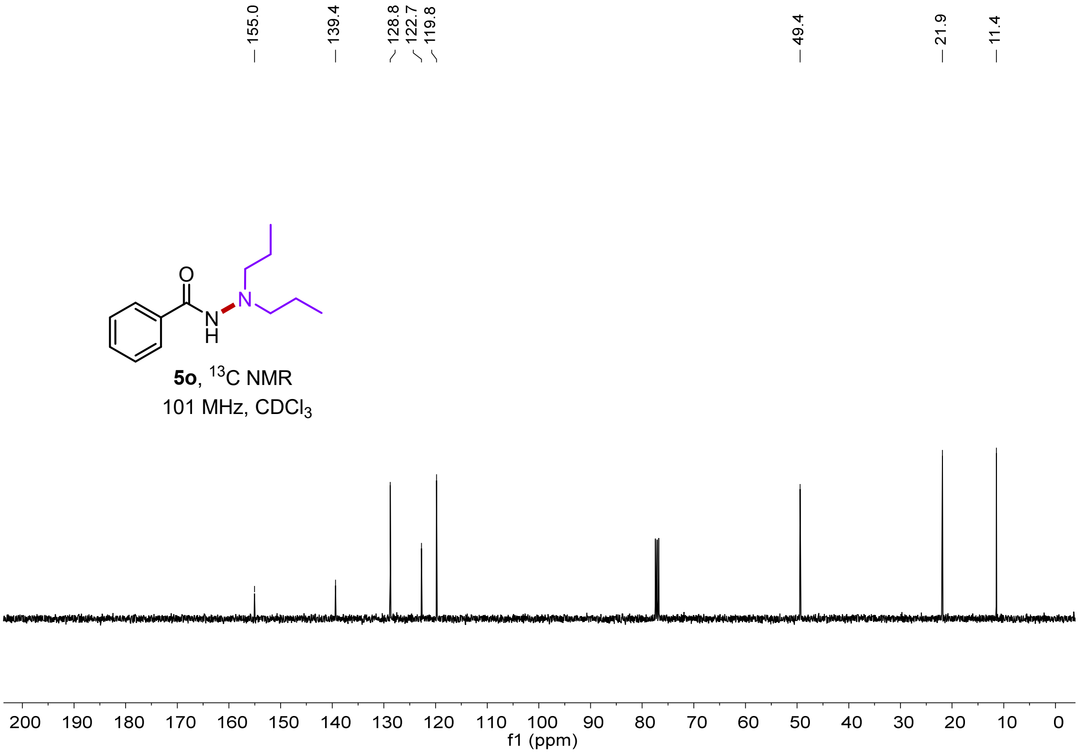
155.0
139.4
128.8
122.7
119.8
49.4
21.9
11.4
O
N
H
N
5o, 13C NMR
101 MHz, $CDCl_3$
200 190 180 170 160 150 140 130 120 110 100 90 80 70 60 50 40 30 20 10 0
f1 (ppm)

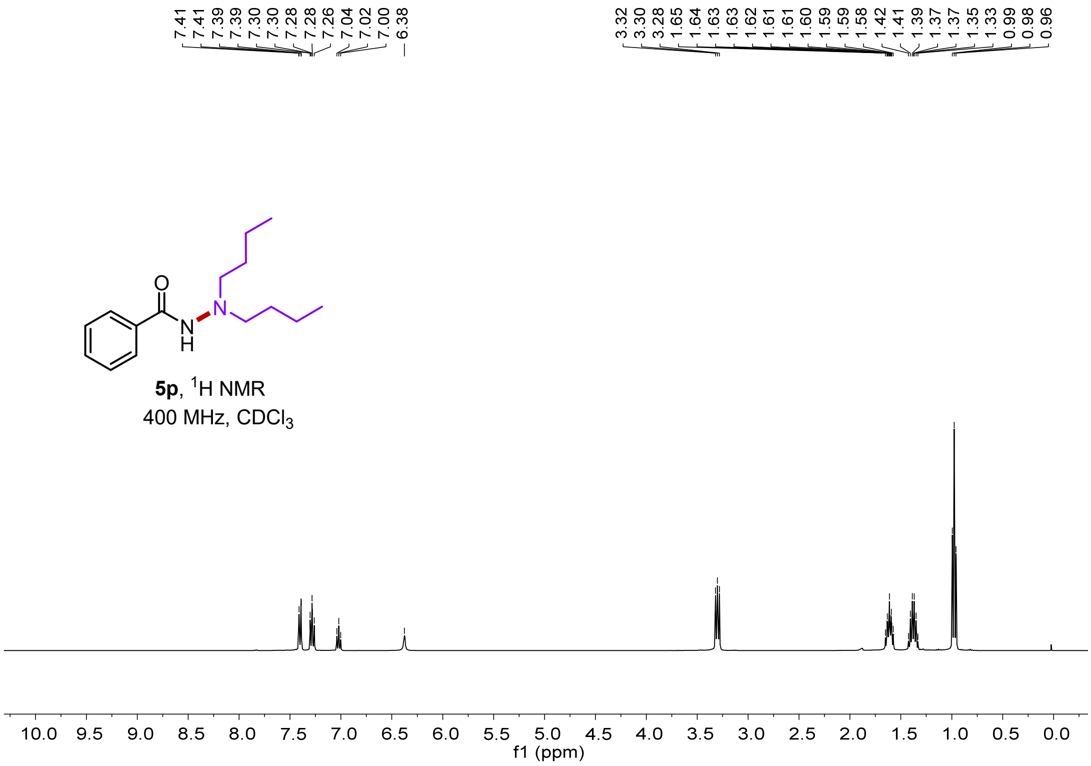
7.41 7.41 7.39 7.39 7.30 7.30 7.28 7.28 7.26 7.04 7.02 7.00 6.38
3.32 3.30 3.28 1.65 1.64 1.63 1.63 1.62 1.61 1.61 1.60 1.59 1.59 1.58 1.42 1.41 1.39 1.37 1.37 1.35 1.33 0.99 0.98 0.96
O
N
H
N
5p, 1H NMR
400 MHz, CDCl3
10.0 9.5 9.0 8.5 8.0 7.5 7.0 6.5 6.0 5.5 5.0 4.5 4.0 3.5 3.0 2.5 2.0 1.5 1.0 0.5 0.0
f1 (ppm)

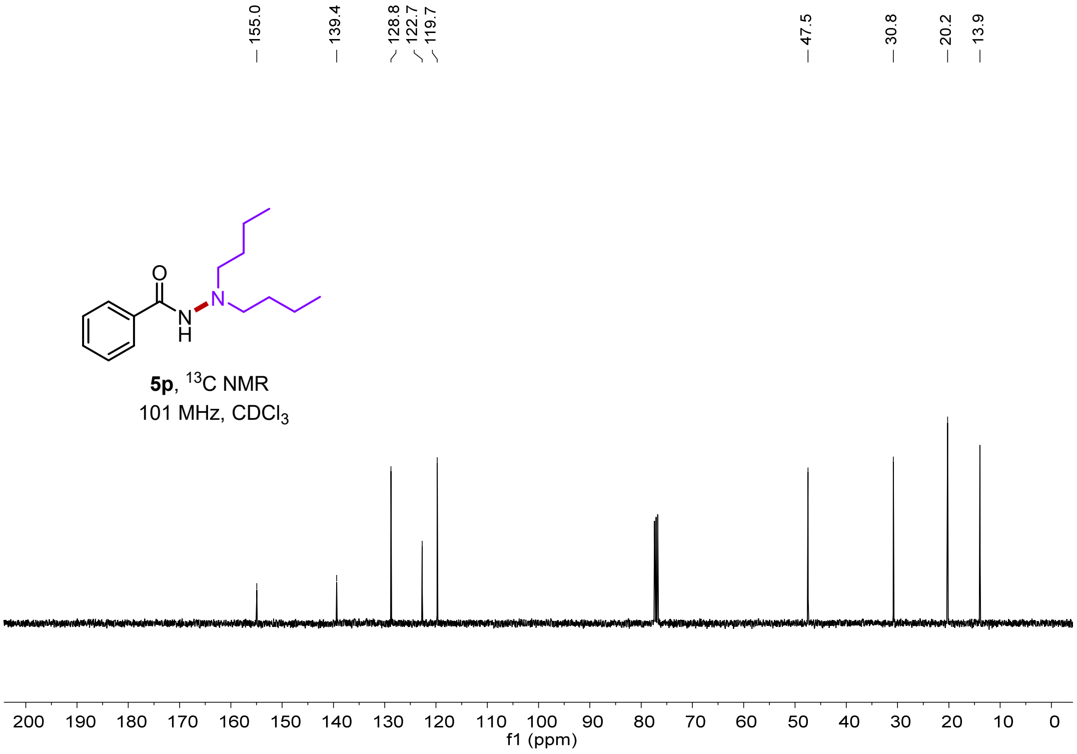
155.0
139.4
128.8 122.7 119.7
47.5
30.8
20.2
13.9
O
N
H
N
5p, 13C NMR
101 MHz, CDCl3
200 190 180 170 160 150 140 130 120 110 100 90 80 70 60 50 40 30 20 10 0
f1 (ppm)

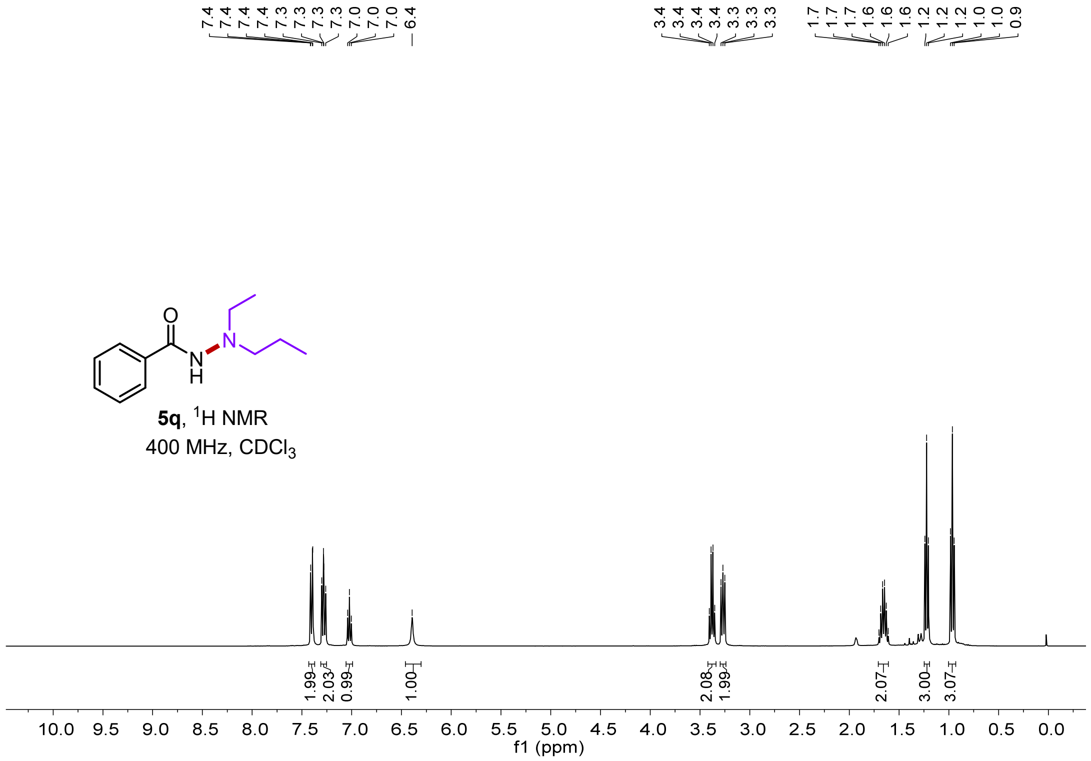
5q, 1H NMR
400 MHz, CDCl3
f1 (ppm)

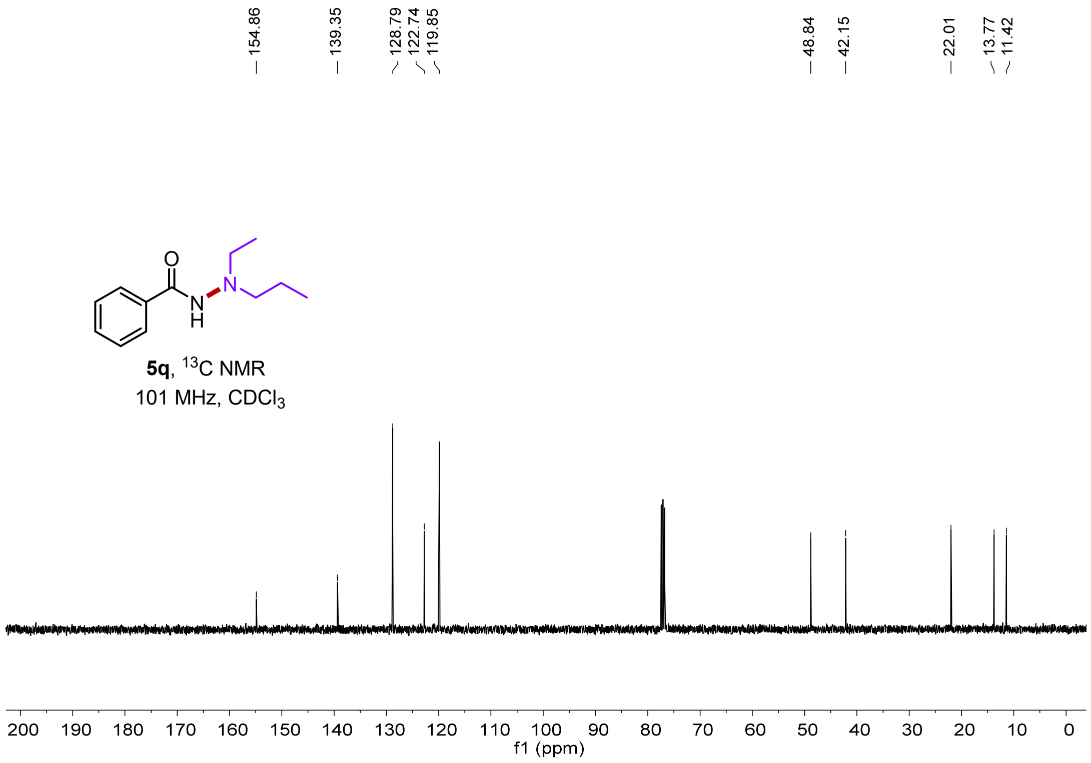
5q, 13C NMR
101 MHz, CDCl3
154.86
139.35
128.79
122.74
119.85
48.84
42.15
22.01
13.77
11.42
f1 (ppm)

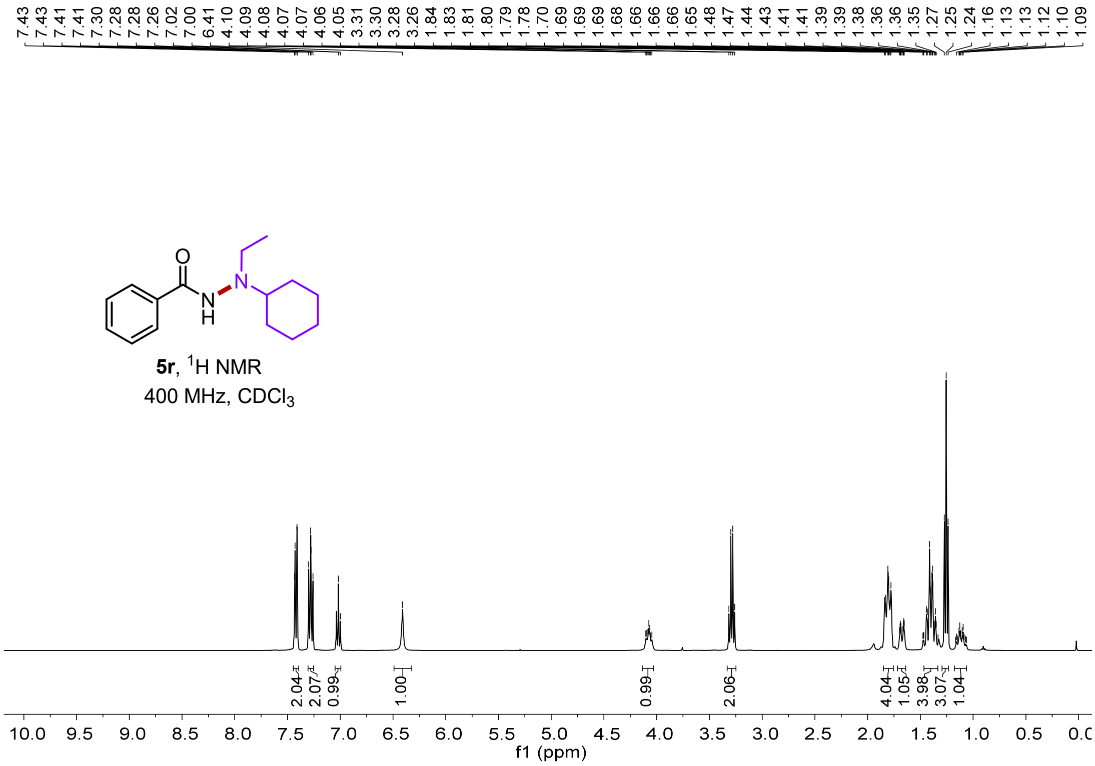

7.43
7.43
7.41
7.41
7.30
7.28
7.28
7.26
7.02
7.00
6.41
4.10
4.09
4.08
4.07
4.07
4.06
4.05
3.31
3.30
3.28
3.26
1.84
1.83
1.81
1.80
1.79
1.78
1.70
1.69
1.69
1.69
1.68
1.66
1.66
1.66
1.65
1.48
1.47
1.44
1.43
1.41
1.41
1.39
1.39
1.38
1.36
1.36
1.35
1.27
1.25
1.24
1.16
1.13
1.13
1.12
1.10
1.09
5r, 1H NMR
400 MHz, CDCl3
2.04
2.07
0.99
1.00
0.99
2.06
4.04
1.05
3.98
3.07
1.04
10.0 9.5 9.0 8.5 8.0 7.5 7.0 6.5 6.0 5.5 5.0 4.5 4.0 3.5 3.0 2.5 2.0 1.5 1.0 0.5 0.0
f1 (ppm)

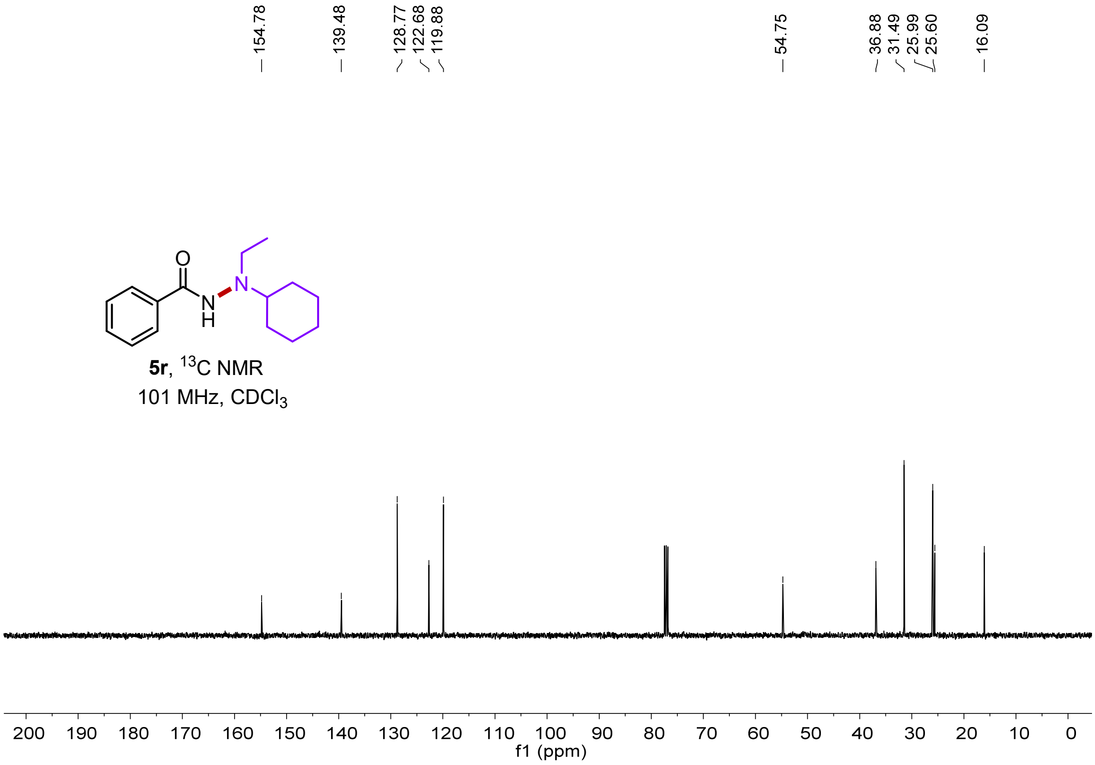

154.78
139.48
128.77
122.68
119.88
54.75
36.88
31.49
25.99
25.60
16.09
5r, 13C NMR
101 MHz, CDCl3
200 190 180 170 160 150 140 130 120 110 100 90 80 70 60 50 40 30 20 10 0
f1 (ppm)

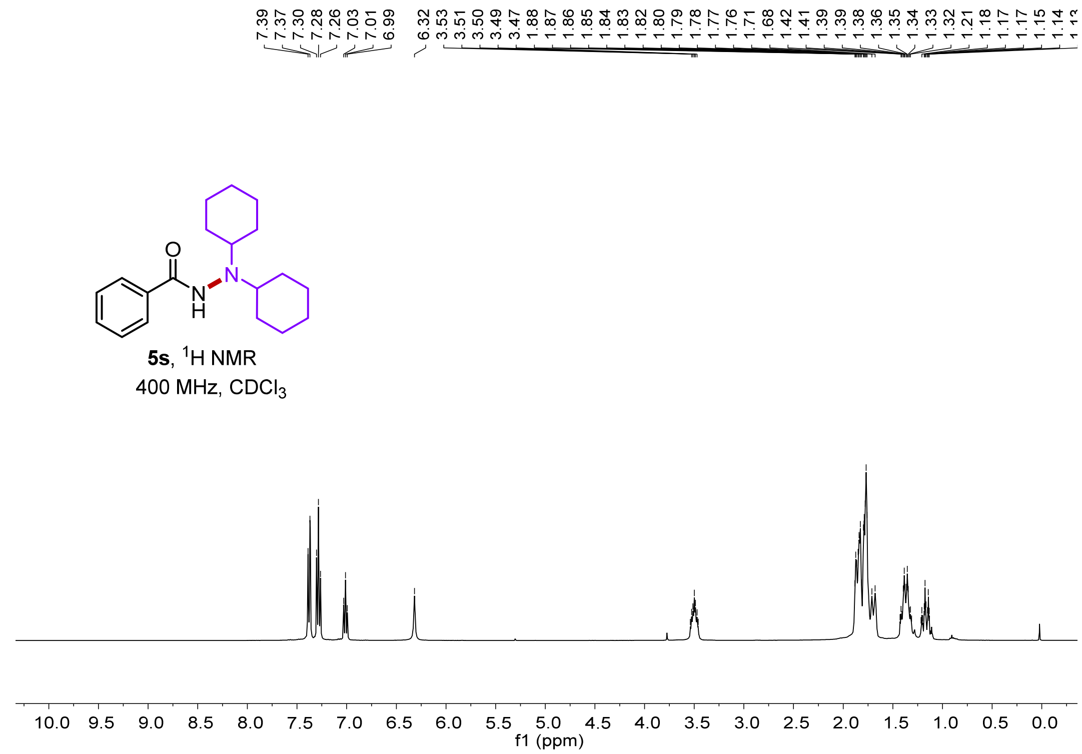
7.39 7.37 7.30 7.28 7.26 7.03 7.01 6.99 6.32 3.53 3.51 3.50 3.49 3.47 1.88 1.87 1.86 1.85 1.84 1.83 1.82 1.80 1.79 1.78 1.77 1.76 1.71 1.68 1.42 1.41 1.39 1.38 1.36 1.35 1.34 1.33 1.32 1.21 1.18 1.17 1.15 1.14
O
N
H
N
5s, ^{1}H NMR
400 MHz, $CDCl_3$
10.0 9.5 9.0 8.5 8.0 7.5 7.0 6.5 6.0 5.5 5.0 4.5 4.0 3.5 3.0 2.5 2.0 1.5 1.0 0.5 0.0
f1 (ppm)

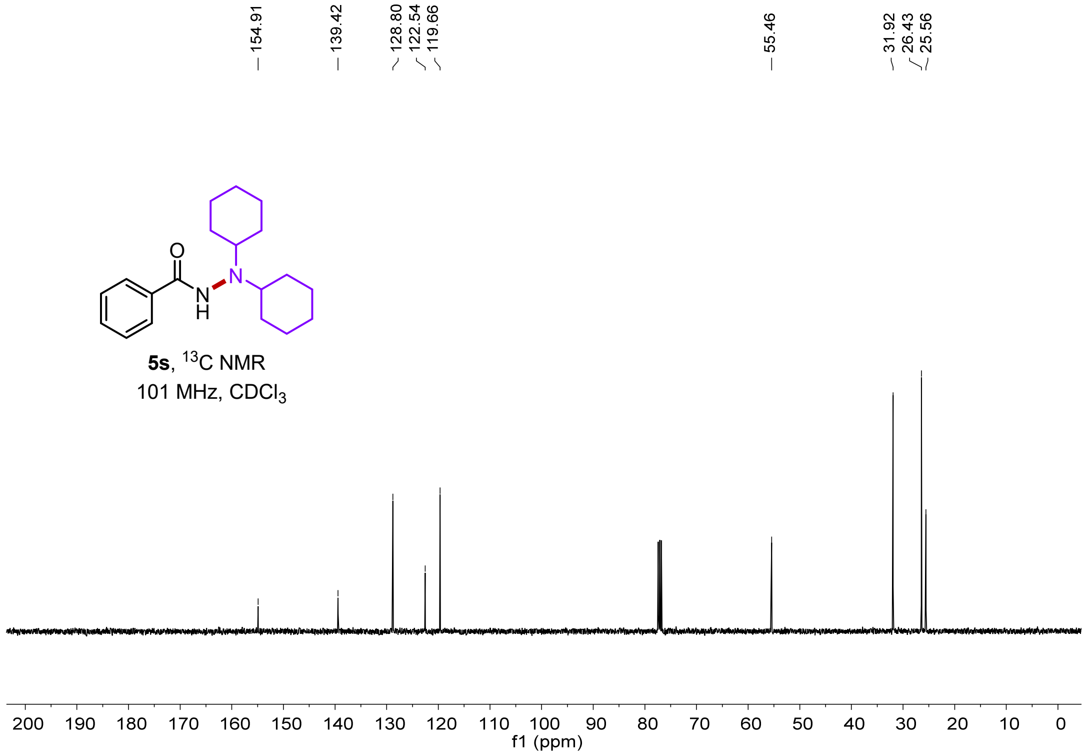
154.91 139.42 128.80 122.54 119.66 55.46 31.92 26.43 25.56
O
N
H
N
5s, ^{13}C NMR
101 MHz, $CDCl_3$
200 190 180 170 160 150 140 130 120 110 100 90 80 70 60 50 40 30 20 10 0
f1 (ppm)

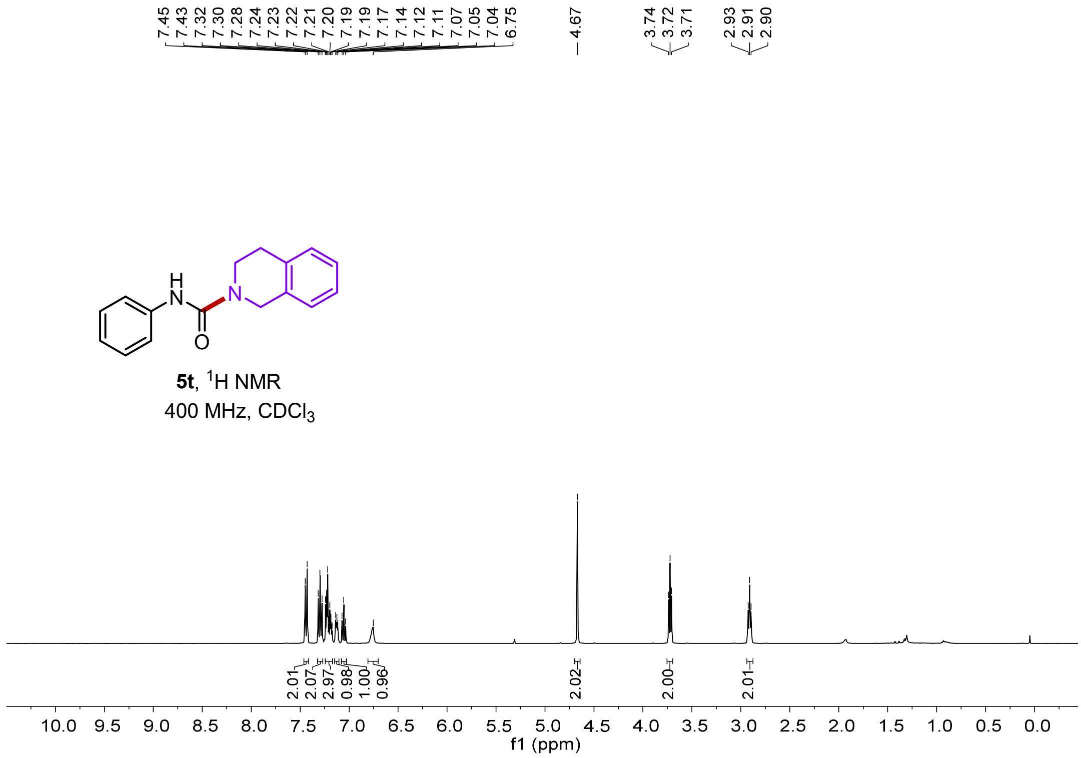

5t, ^{1}H NMR
400 MHz, $CDCl_3$

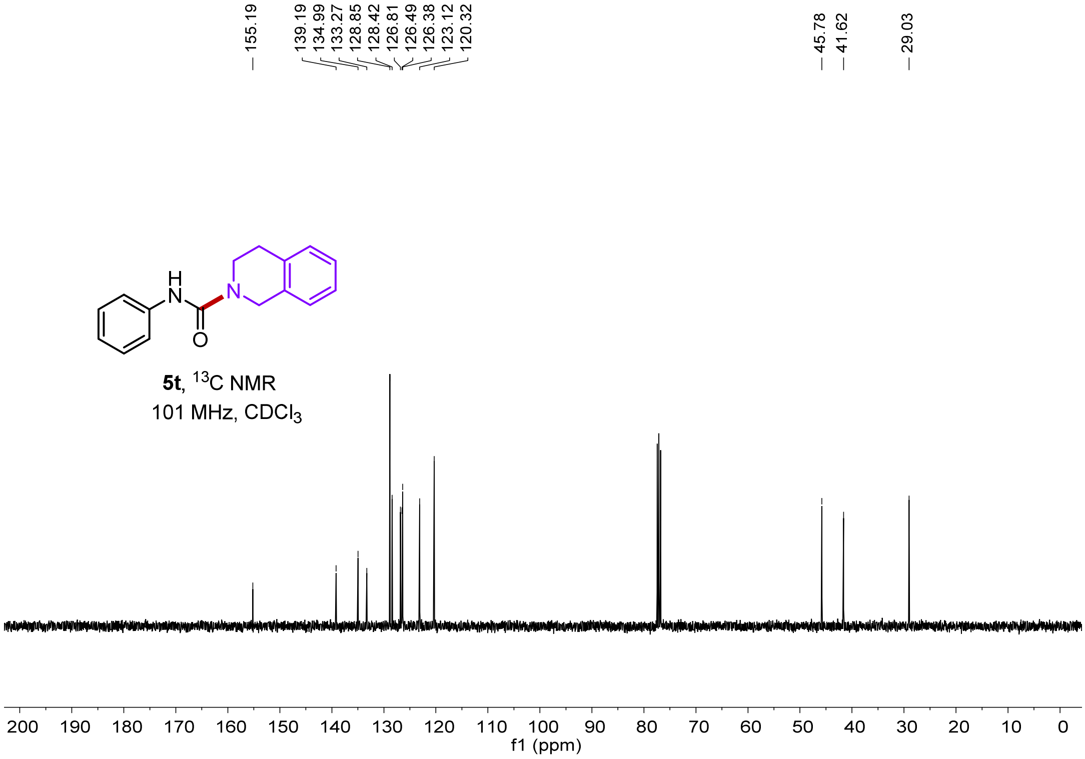

5t, ^{13}C NMR
101 MHz, $CDCl_3$

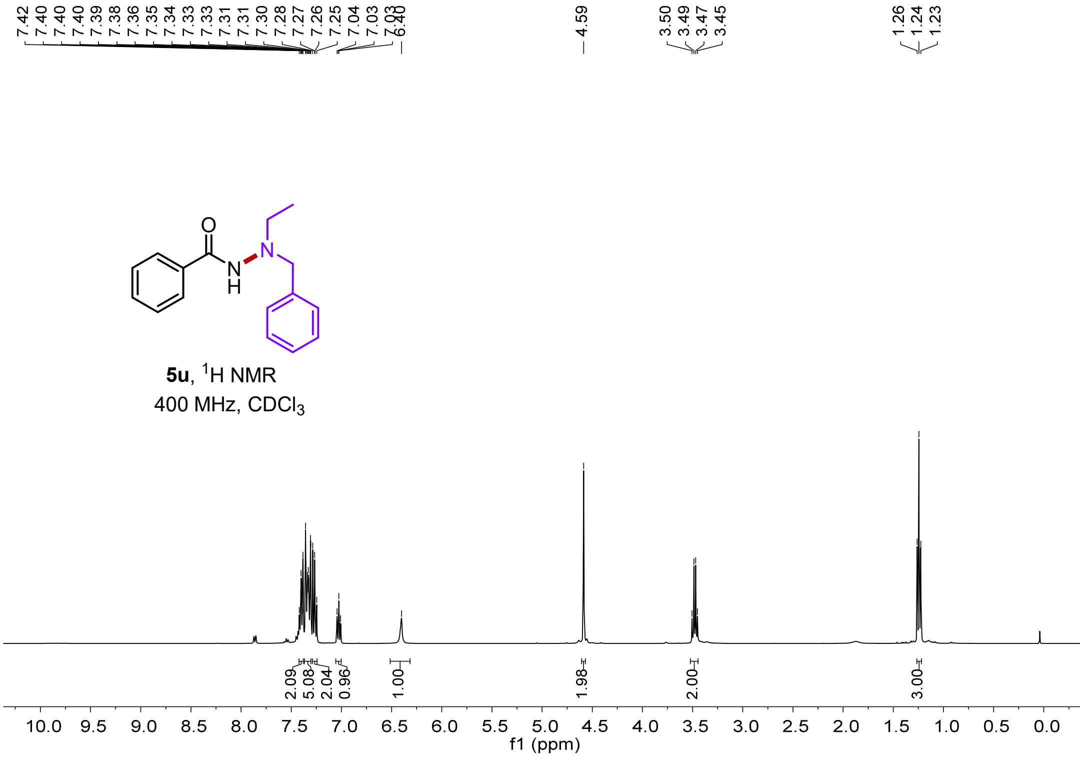
7.42 7.40 7.40 7.40 7.39 7.38 7.36 7.35 7.34 7.33 7.33 7.31 7.31 7.30 7.28 7.27 7.26 7.25 7.04 7.03 7.03 6.40
4.59
3.50 3.49 3.47 3.45
1.26 1.24 1.23
O
N
H
N
5u, ^{1}H NMR
400 MHz, $CDCl_3$
2.09 5.08 2.04 0.96 1.00 1.98 2.00 3.00
10.0 9.5 9.0 8.5 8.0 7.5 7.0 6.5 6.0 5.5 5.0 4.5 4.0 3.5 3.0 2.5 2.0 1.5 1.0 0.5 0.0
f1 (ppm)

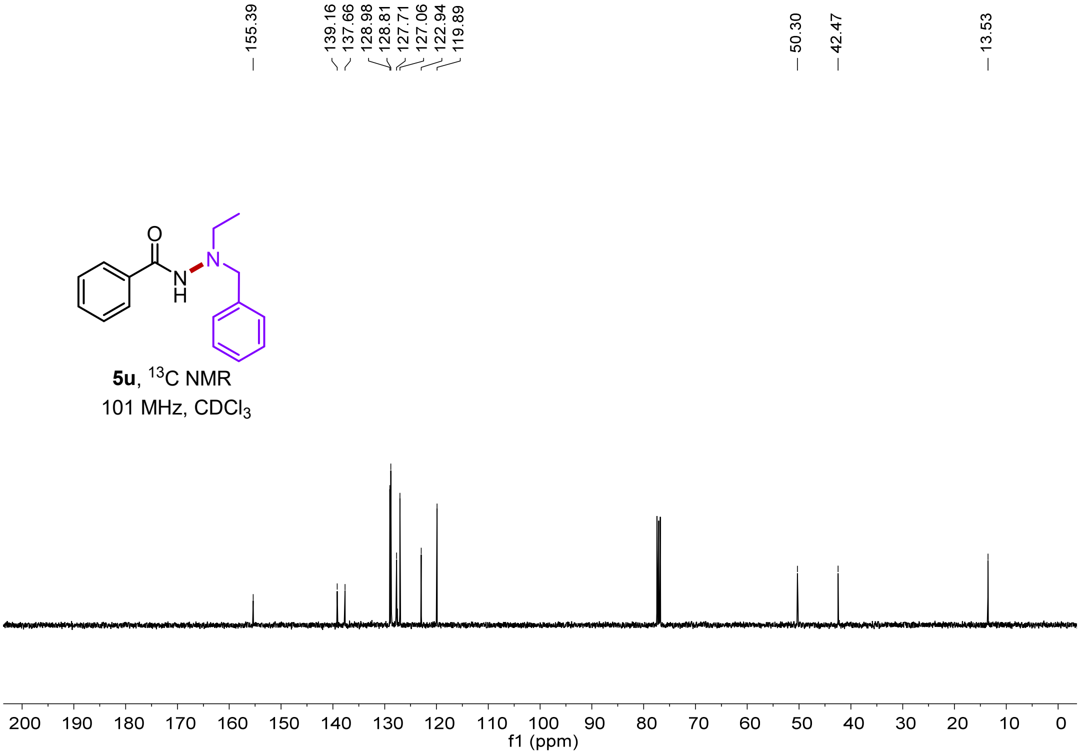
155.39
139.16 137.66 128.98 128.81 127.71 127.06 122.94 119.89
50.30 42.47
13.53
O
N
H
N
5u, ^{13}C NMR
101 MHz, $CDCl_3$
200 190 180 170 160 150 140 130 120 110 100 90 80 70 60 50 40 30 20 10 0
f1 (ppm)

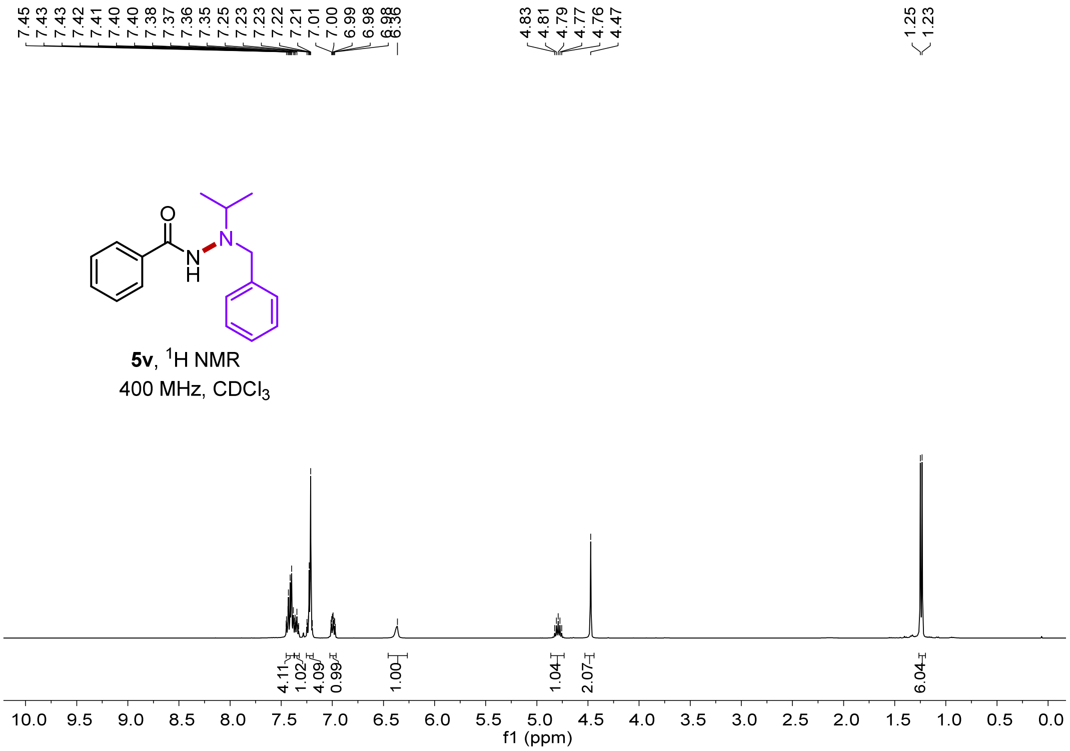
7.45
7.43
7.43
7.42
7.41
7.40
7.40
7.38
7.37
7.36
7.35
7.25
7.23
7.23
7.22
7.21
7.01
7.00
6.99
6.98
6.36
4.83
4.81
4.79
4.77
4.76
4.47
1.25
1.23
O
N
N
H
5v, 1H NMR
400 MHz, CDCl3
4.11
1.02
4.09
0.99
1.00
1.04
2.07
6.04
10.0 9.5 9.0 8.5 8.0 7.5 7.0 6.5 6.0 5.5 5.0 4.5 4.0 3.5 3.0 2.5 2.0 1.5 1.0 0.5 0.0
f1 (ppm)

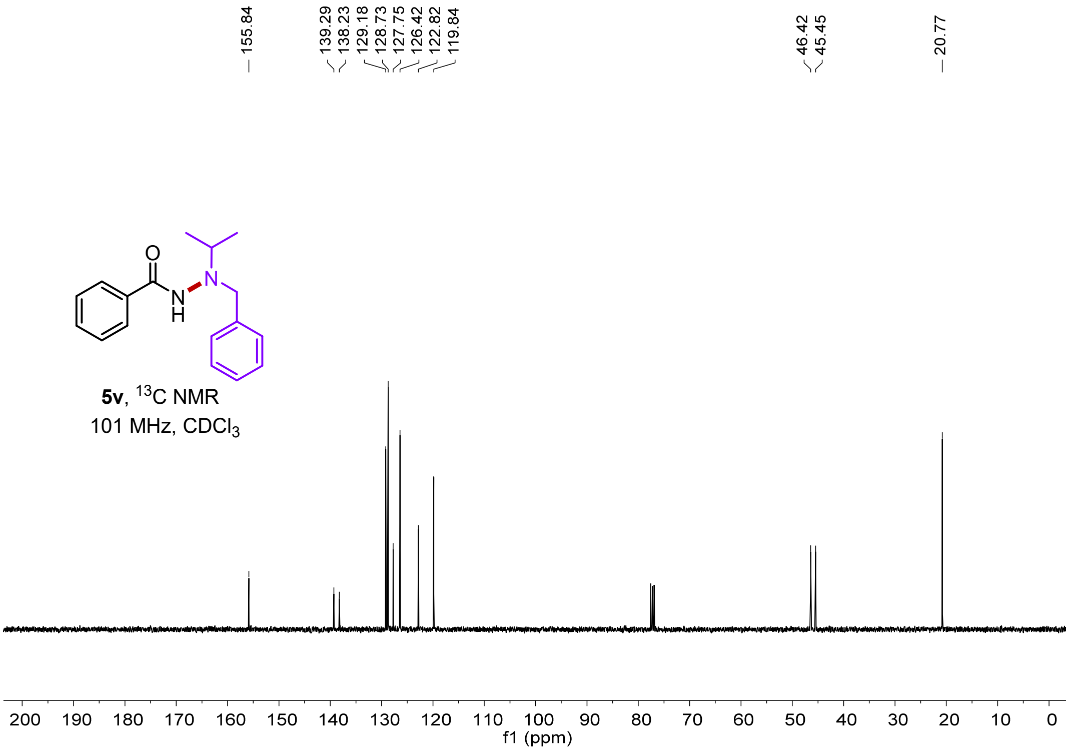
155.84
139.29
138.23
129.18
128.73
127.75
126.42
122.82
119.84
46.42
45.45
20.77
O
N
N
H
5v, 13C NMR
101 MHz, CDCl3
200 190 180 170 160 150 140 130 120 110 100 90 80 70 60 50 40 30 20 10 0
f1 (ppm)

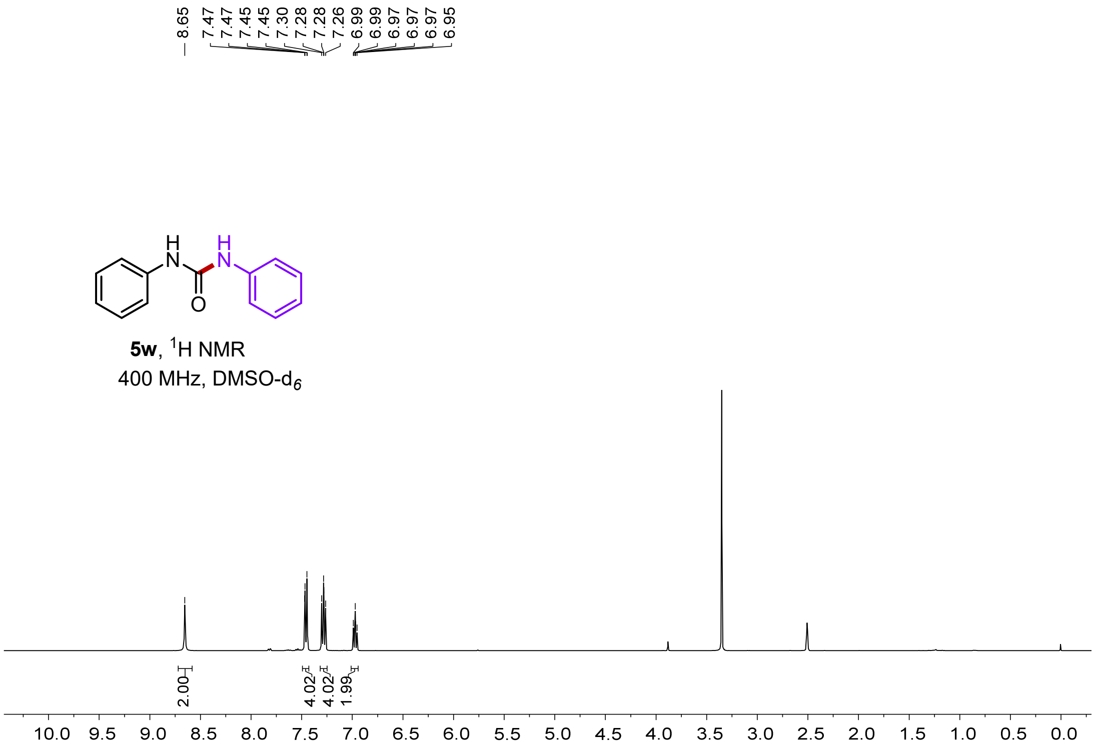
8.65
7.47
7.47
7.45
7.45
7.30
7.28
7.28
7.26
6.99
6.99
6.97
6.97
6.97
6.95
5w, ^{1}H NMR
400 MHz, DMSO-d$_6$
2.00
4.02
4.02
1.99
10.0 9.5 9.0 8.5 8.0 7.5 7.0 6.5 6.0 5.5 5.0 4.5 4.0 3.5 3.0 2.5 2.0 1.5 1.0 0.5 0.0

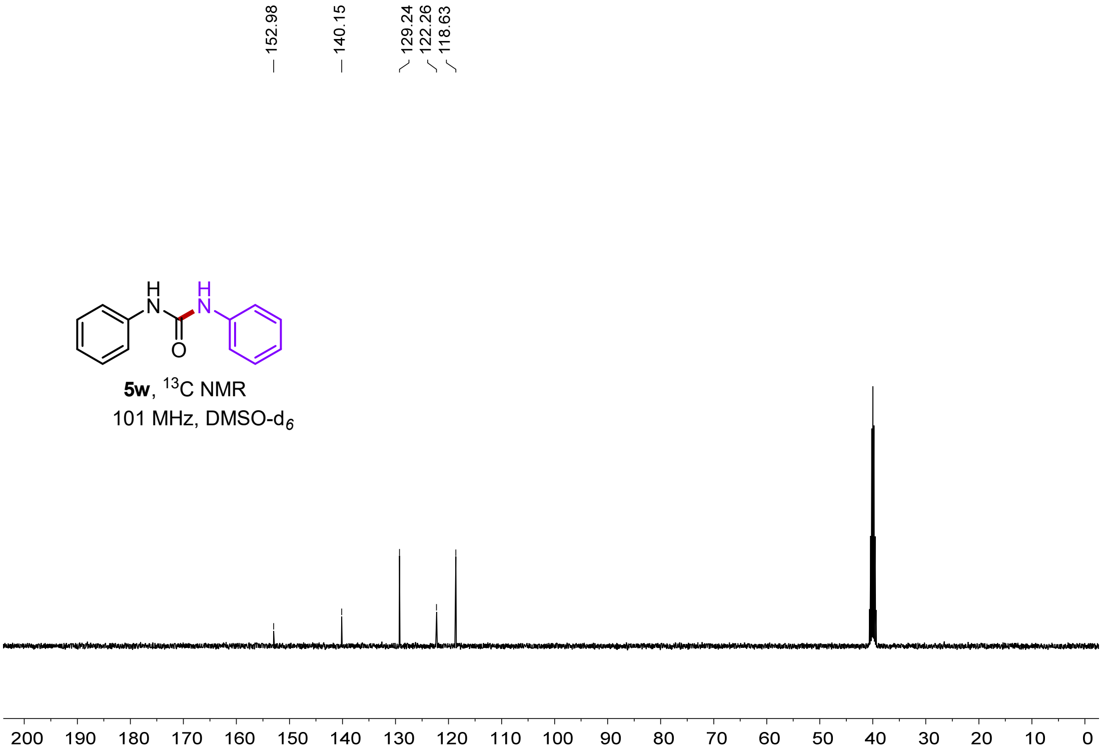
152.98
140.15
129.24
122.26
118.63
5w, ^{13}C NMR
101 MHz, DMSO-d$_6$
200 190 180 170 160 150 140 130 120 110 100 90 80 70 60 50 40 30 20 10 0
